# Supplementary material for: Multi-component reactions via copper(I) difluorocarbene as carbonyl source for constructing α—aminoamide derivatives
Source: Nat Commun. 2025 Jul 18;16:6643. doi: 10.1038/s41467-025-61947-z (PMC12274583; doi:10.1038/s41467-025-61947-z)
Supplement: Supplementary file 1 — Supplementary Information [file 41467_2025_61947_MOESM1_ESM.pdf]

Supplementary Information

Multi-Component Reactions *via* Copper(I) Difluorocarbene as Carbonyl Source for Constructing *α*-Aminoamide Derivatives

Jiuling Li* †, Baofan Wang †, Taichen Liu †, Qinhong Wen, Tongfei Jing, Xiang Fu, Yingming Pan, Kai Wei, Xiaoyu Zhou*, Wenhao Hu, Zhenghui Kang*

School of Medical Sciences, Pingdingshan University, Pingdingshan 467000, China
Zhongshan Institute for Drug Discovery, Shanghai Institute of Materia Medica, Chinese Academy of Sciences, Zhongshan 528400, China.
School of Chemistry and Pharmaceutical Sciences, Guangxi Normal University, Guilin 541004, China.
School of Pharmaceutical Sciences, Sun Yat-sen University, Guangzhou 510006, China
School of Pharmaceutical and Chemical Engineering, Taizhou University, Taizhou, China

E-mail: kangzhenghui@simm.ac.cn; orgchem90@163.com; zhouxiaoyu@tzc.edu.cn

1. General information & materials 1

2. Experimental procedures..2

3. Complete data for optimization of the reaction conditions 4

4. Control experiments..8

5. DFT computations for the formation of intermediate IV 15

6. Single crystal X-ray diffraction data 18

7. Analytical data of products 20

8. NMR spectra of products 44

9. Reference 126

1. General information & materials

General: All ^{1}H NMR (500 MHz, 600MHz) and ^{13}C NMR (125 MHz, 150 MHz) and ^{19}F NMR (471MHz) spectra were recorded on 500 or 600 MHz spectrometers in in $CDCl_3$, DMSO-d_6 and Methanol-d_4. Chemical shifts were reported in ppm with the solvent signal as reference, and coupling constants (J) were given in Hertz. The peak information was described as: s = singlet, d = doublet, t = triplet, q = quartet, m = multiplet, br = broad. High-resolution mass spectrometry (HRMS) was recorded on a commercial apparatus (ESI Source). Single crystal X-ray diffraction data were recorded on Bruker-AXS SMART APEX II single crystal X-ray diffractometer.

Materials: Unless otherwise noted below, commercially available reagents were used throughout without further purification, and all reactions were performed using standard Schlenk techniques under an atmosphere of argon. Solvents were dried and distilled followed the standard methods before using. Aldehydes **3ab** and **3ac** derived from Gemfibrozil and Ciprofibrate were synthesized using the following literature[1]. Analytical thin-layer chromatography was performed using glass plates pre-coated with 200-300 mesh silica gel impregnated with a fluorescent indicator (254 nm). Flash column chromatography was performed using silica gel (300-400 mesh). Materials were prepared according to the known procedure and had physical and spectral properties identical to those earlier reported.

2. Experimental procedures

2.1 General procedure for multi-component reaction

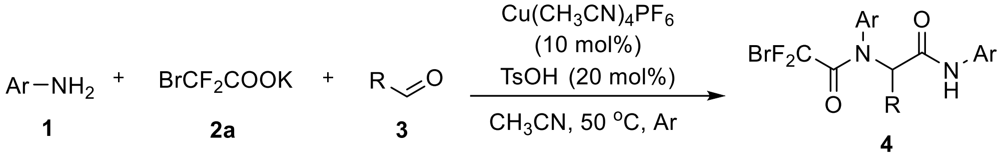

To an oven-dried 10 mL Schlenk tube equipped with a stir bar was added $Cu(CH_3CN)_4PF_6$ (7.5mg, 0.02mmol, 10.0 mol%), TsOH (6.9mg, 0.04mmol, 20.0 mol%) , aromatic amines **1** (0.5 mmol, 2.5 equiv), $BrCF_2COOK$ **2a** (128mg, 0.6 mmol, 3.0 equiv) , and aldehydes **3** (0.2 mmol, 1.0 equiv), and suspended in CH_3CN (3.0 mL) under dry argon atmosphere. The resulting mixture was stirred at 50°C for 12 hours. The progress of the reaction was monitored by TLC. After the reaction was complete, the reaction was cooled to room temperature and concentrated under reduced pressure. The residue was purified by flash column chromatography (eluent: EA:PE = 1/20~1/5) to give the pure product **4**.

2.2 Procedure for the product derivatizations

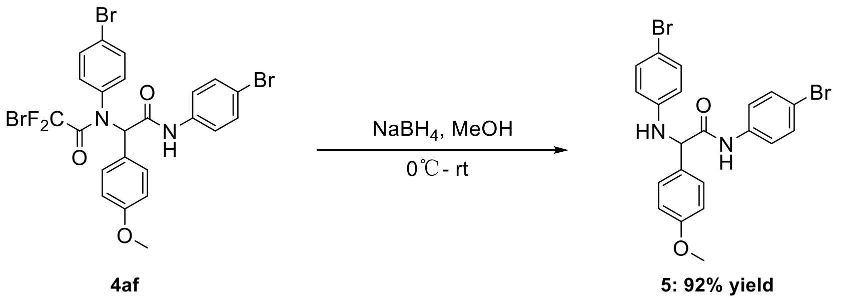

Synthesis of 5**:** To a round bottom flask with a stir bar was added **4af** (0.2 mmol, 1.0 equiv) and MeOH (2.0 mL), and cooled to 0°C. Then $NaBH_4$ (0.4 mmol, 2.0 equiv) was added dropwise at 0℃, and the resulting mixture was stirred at room temperature for 2 h. The reaction was quenched by the addition of a saturated aqueous NH_4Cl (2.0 mL). The layers were separated and the aqueous layer was extracted with EtOAc (2 x 5 mL), then dried over Na_2SO_4, filtered and concentrated *in vacuo*. The mixture was purified by column chromatography on silica gel (eluent: EA:PE = 1/8) to give the pure products **5** (92% yield).

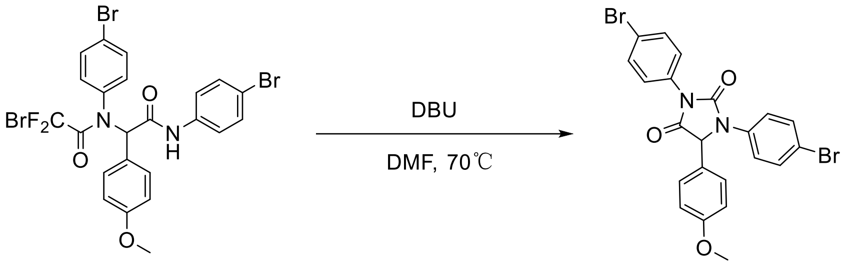

4af **6: 90% yield**

***Synthesis of 6*:** To an oven-dried round bottom flask equipped with a stir bar was added **4af** (0.2 mmol, 1.0 equiv), and dissolved in DMF (2.0 mL). The solution was then heated to 70°C. Dissolved in DMF (2.0 mL), DBU (0.44 mmol, 2.2 equiv) was added dropwise to the mixture and stirred for 10 h. The solution was poured into water (2.0 mL) to quench the reaction and the mixture was extracted with EtOAc (2 x 5.0 mL). The combined organic layers were washed with brine, dried over anhydrous $NaSO_4$, filtered, and concentrated under reduced pressure. The residue was purified by column chromatography on silica gel (eluent: EA:PE = 1/8) to afford the pure product **6** (90% yield).

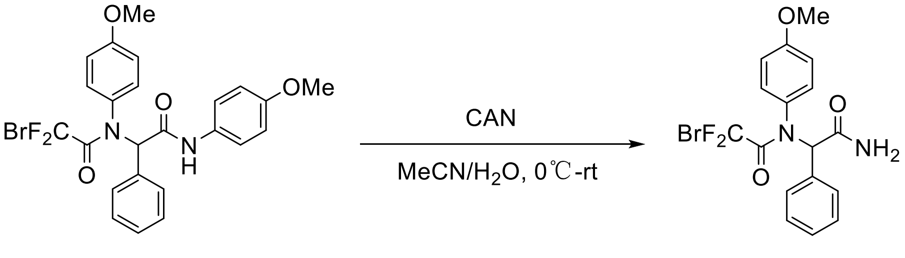

4g **7: 55% yield**

***Synthesis of 7*:** To a round bottom flask with a stir bar was added CAN (0.8 mmol, 4.0 equiv) and H_2O (4.0 mL), and cooled to 0°C. Dissolved in CH_3CN (2.0 mL), **4g** (0.2 mmol, 1.0 equiv) was added dropwise to the mixture, followed by stirring the reaction at room temperature for 8 h. The reaction was then quenched by the addition of a saturated aqueous Na_2CO_3 solution (2.0 mL). The layers were separated and the aqueous layer was extracted with EtOAc (2 x 5 mL), then dried over Na_2SO_4, filtered and concentrated *in vacuo*. The mixture was purified by column chromatography on silica gel (eluent: EA:PE = 1/5) to give the pure product **7** (55% yield).

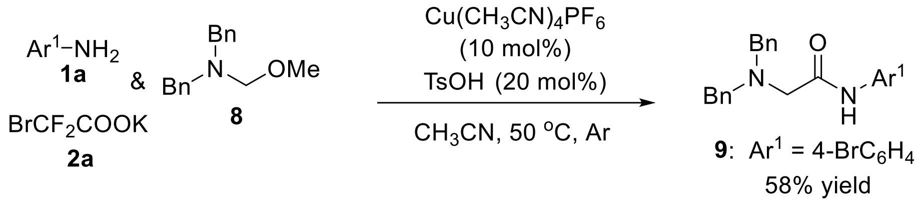

Synthesis of 9: To an oven-dried 10 mL Schlenk tube equipped with a stir bar was added $Cu(CH_3CN)_4PF_6$ (7.5mg, 0.02mmol, 10.0 mol%), TsOH (6.9mg, 0.04mmol, 20.0 mol%) , aromatic amine **1a** (0.3 mmol, 1.5 equiv), $BrCF_2COOK$ **2a** (128mg, 0.6 mmol, 3.0 equiv) , and *N*,*N*-dibenzyl-1-methoxymethanamine **8** (0.2 mmol, 1.0 equiv), and suspended in CH_3CN (2.0 mL) under dry argon atmosphere. The resulting mixture was stirred at 50℃ for 12 hours. The progress of the reaction was monitored by TLC. After the reaction was complete, the reaction was cooled to room temperature and concentrated under reduced pressure. The residue was purified by flash column chromatography (eluent: EA:PE = 1/20~1/10) to give the pure product **9** (58% yield).

3. Complete data for optimization of the reaction conditions

Table S1. *Optimization of the reaction conditions* [a]

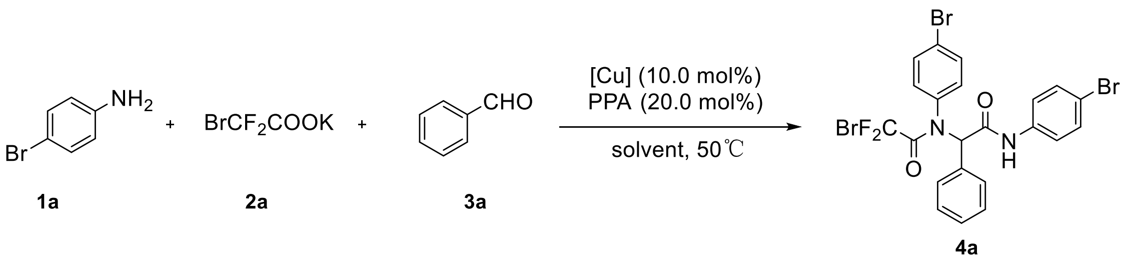

Entry	[Cu]	solvent	yield[b](%)
1	CuCl	CH_3CN	57
2	CuI	CH_3CN	55
3	CuBr	CH_3CN	58
4	$CuCl_2$	CH_3CN	50
5	$CuBr_2$	CH_3CN	63
6	$Cu(OAc)_2$	CH_3CN	60
7	$CuSO_4$	CH_3CN	54
8	$Cu(OTf)_2$	CH_3CN	62
9	CuOTf	CH_3CN	61
10	$Cu(CH_3CN)_4PF_6$	CH_3CN	78
11	$Cu(CH_3CN)_4BF_4$	CH_3CN	65
12	$Cu(CH_3CN)_4PF_6$	THF	N.D.
13	$Cu(CH_3CN)_4PF_6$	1,4-dioxane	N.D.

14	$Cu(CH_3CN)_4PF_6$	DMF	N.D.
15	$Cu(CH_3CN)_4PF_6$	DMSO	N.D.
16	$Cu(CH_3CN)_4PF_6$	DCE	N.D.
17	$Cu(CH_3CN)_4PF_6$	MTBE	N.D.
18	$Cu(CH_3CN)_4PF_6$	Xylenes	N.D.

[a] The reactions were conducted on a 0.2 mmol scale: **1**: **2**: **3** = 2.5: 3: 1, copper catalyst (10.0 mol %), PPA (20.0 mol %), the corresponding solvent (3 mL). [b] Isolated yield. N.D. = not detected

Table S2. *Optimization of the reaction conditions* [a]

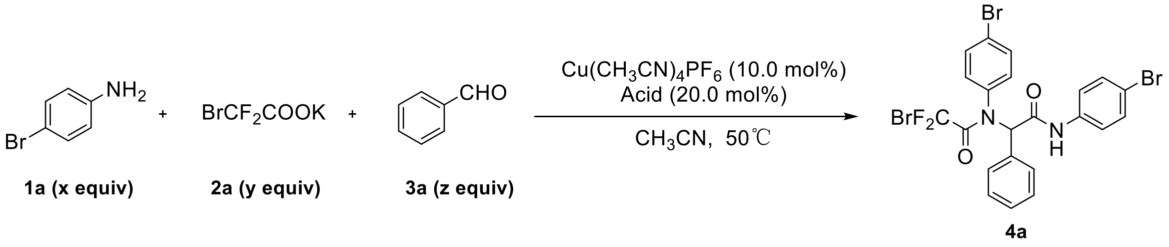

1a (x equiv) **2a (y equiv)** **3a (z equiv)** **4a**

Entry	x : y : z	Acid	yield[b](%)
1	2: 3: 1	HCOOH	58
2	2: 3: 1	AcOH	58
3	2: 3: 1	$MeSO_3H$	63
4	2: 3: 1	Isobutyric acid	50
5	2: 3: 1	PPA	66
6	2.2: 3: 1	$MeSO_3H$	68
7	2.2: 3: 1	Isonipecotic acid	51
8	2.2: 3: 1	H_3PO_4(85% in water)	67
9	2.2: 3: 1	1-adamantanic acid	58
10	2.2: 3: 1	PPA	72
11	2.2: 3: 1	TsOH	75
12	2.5: 3: 1	PPA	78
13	2.5: 3: 1	$MeSO_3H$	80
14	2.5: 3: 1	HCOOH	68
15	2.5: 3: 1	AcOH	68
16	2.5: 3: 1	H_3PO_4(85% in water)	72
17	2.5: 3: 1	1-adamantanic acid	65
18	2.5: 3: 1	TsOH	83
19	3: 3: 1	TsOH	83

[a] The reactions were conducted on a 0.2 mmol scale: **1**: **2**: **3** = x: y: z, $Cu(CH_3CN)_4PF_6$(10.0 mol %), Acid (20.0 mol %), CH_3CN (3 mL). [b] Isolated yield.

Table S3. *Attemption of the chiral phosphoric acid for enantioselective control of the reaction of* ***1a****,* ***2a*** *and* ***3a*** [a]

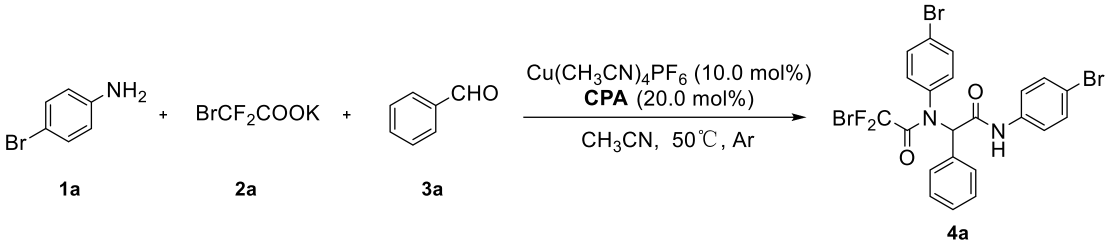

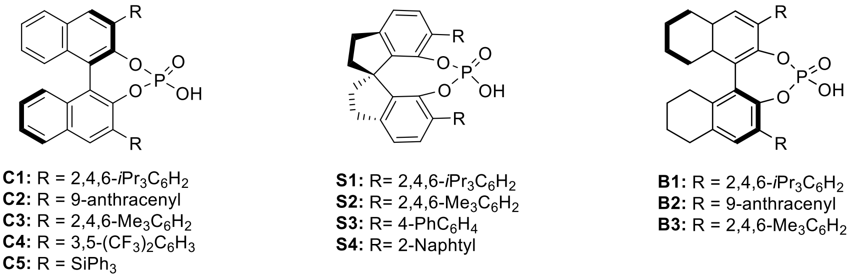

Entry	CPA	yield[b](%)	ee[c](%)
1	**C1**	63	0
2	**C2**	55	0
3	**C3**	58	0
4	**C4**	50	0
5	**C5**	48	0
6	**S1**	56	0
7	**S2**	60	0
8	**S3**	50	0
9	**S4**	62	0
10	**B1**	51	0
11	**B2**	48	0
12	**B3**	47	0

[a] The reactions were conducted on a 0.2 mmol scale: **1**: **2**: **3** = 2.5: 3: 1, $Cu(CH_3CN)_4PF_6$(10.0 mol %), CPA (20.0 mol %), CH_3CN (3 mL). [b] Yields were determined by ^{1}H NMR spectroscopy with 1,3,5-Trimethoxybenzene as an internal standard. [c] Determined by chiral HPLC analysis.

Table S4. *Attemption of the different ligands for enantioselective control of the reaction of* ***1a, 2a*** *and* ***3a*** [a]

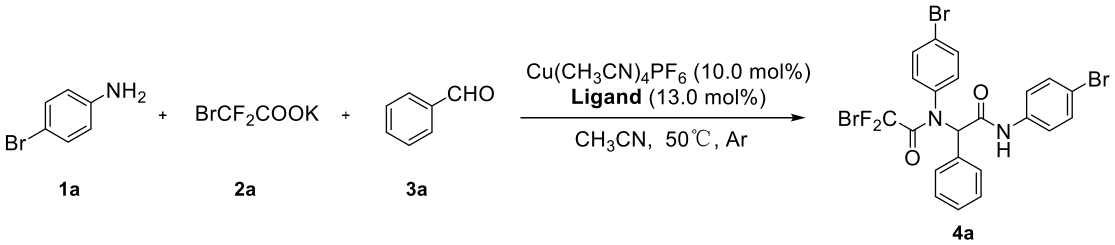

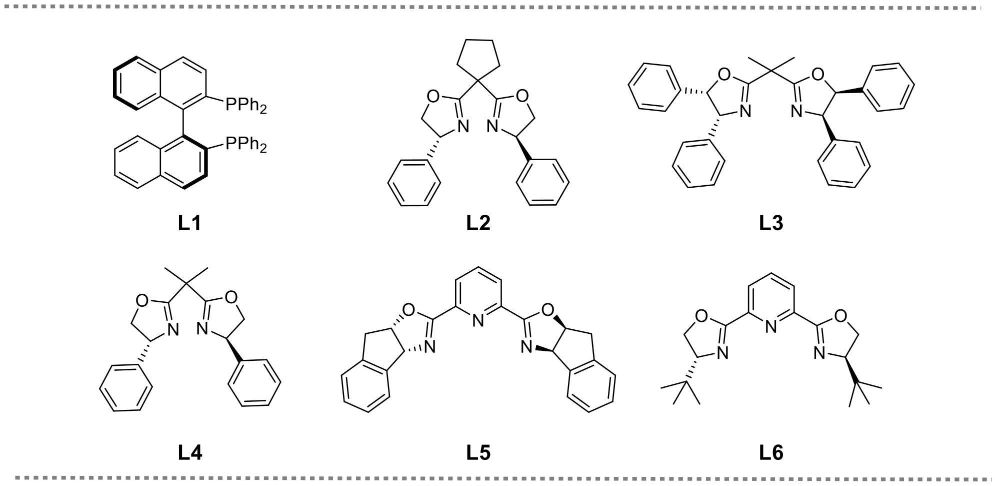

Entry	Ligand	yield[b](%)	ee[c](%)
1	**L1**	40	0
2	**L2**	43	0
3	**L3**	38	0
4	**L4**	45	0
5	**L5**	42	0
6	**L6**	46	0

[a] The reactions were conducted on a 0.2 mmol scale: **1**: **2**: **3** = 2.5: 3: 1, $Cu(CH_3CN)_4PF_6$ (10.0 mol%), Ligand (13.0 mol%) were stirred in CH_3CN Solution (3 mL) under argon at room temperature for 20 min, then the above mixture was stirred at 50 ℃ for 12 h. [b] Yields were determined by ^1H NMR spectroscopy with 1,3,5-Trimethoxybenzene as an internal standard. [c] Determined by chiral HPLC analysis.

4. Control experiments

4.1 Control reaction with/without H_2O and ^{18}O-isotope labeling.

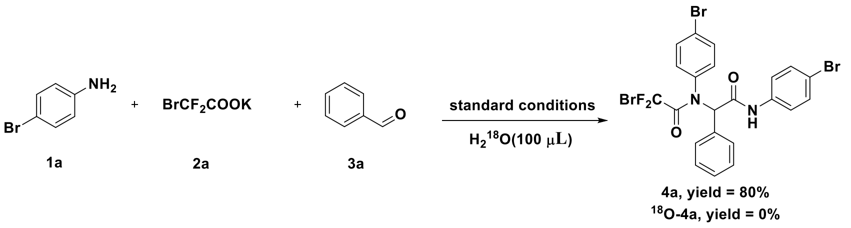

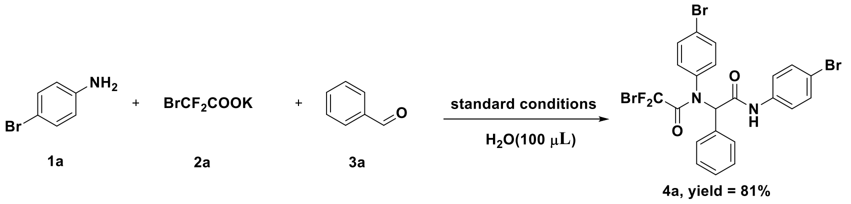

To an oven-dried 10 mL Schlenk tube equipped with a stir bar was added $Cu(CH_3CN)_4PF_6$ (7.5mg, 0.02mmol, 10.0 mol%), TsOH (6.9mg, 0.04mmol, 20.0 mol%), aromatic amines **1a** (0.5 mmol, 2.5 equiv), $BrCF_2COOK$ **2a** (128mg, 0.6 mmol, 3.0 equiv) , and aldehyde **3a** (0.2 mmol, 1.0 equiv), and suspended in CH_3CN (3.0 mL) under dry argon atmosphere. Then either 100 μL oxygen-18 water or water was added to the add to the above mixture, and the resulting mixture was stirred at 50°C for 12 hours. After the reaction was complete, the yield of multi-component products was not affected, and the HRMS results of Figure S1a and Figure S1b were consistent (Figure S1), which indicated that the water molecules generated in situ were not the oxygen source of the product in multi-component reactions.

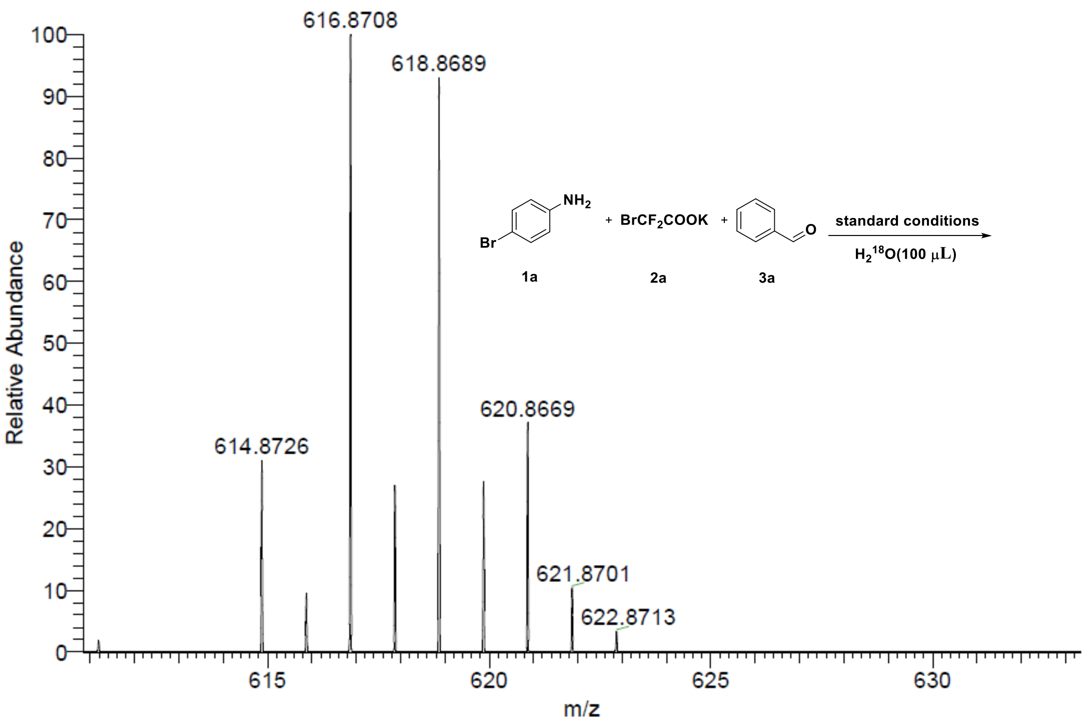

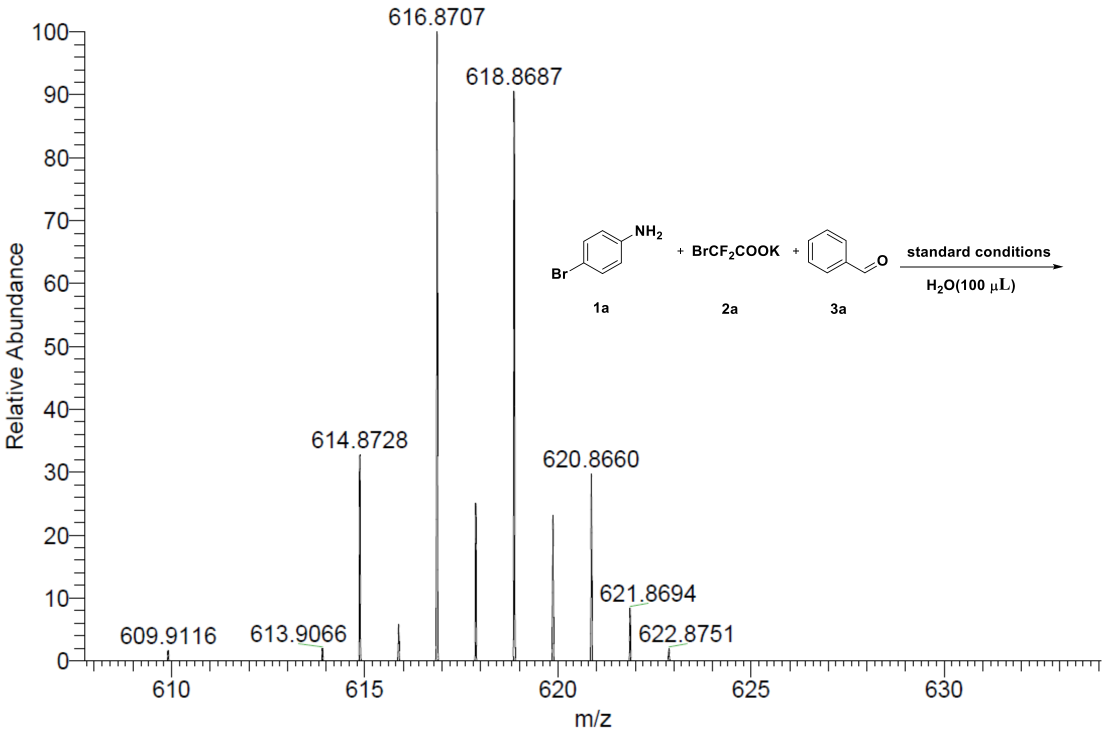

Figure S1 HRMS results of ^{18}O-isotope labeling reactions

4.2 Control reaction without aldehyde

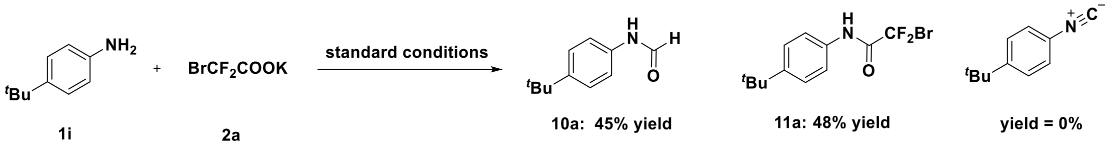

When benzaldehyde was absent in multi-component reactions under standard conditions, *p*-tert butylaniline reacted with $BrCF_2COOK$ to obtain insertion product **10a** and amidation product **11a** with a yield of nearly 1:1 ratio, and the isonitrile was not detected by LC-MS from beginning to end.

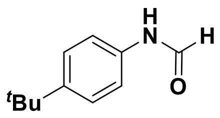

10a: 45% yield

^{1}H NMR (500 MHz, $CDCl_3$) δ 8.68 (d, *J* = 11.4 Hz, 0.56 H), 8.48 (d, *J* = 11.5 Hz, 0.53H), 8.37 (d, *J* = 1.8 Hz, 0.47 H), 7.67 (s, 0.46 H), 7.55 – 7.46 (m, 1H), 7.43 – 7.34 (m, 2H), 7.12 – 7.02 (m, 1H), 1.34 (s, 5H), 1.32 (s, 4H).

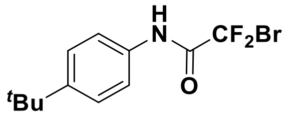

11a: 48% yield

^{1}H NMR (500 MHz, $CDCl_3$) δ 7.79 (s, 1H), 7.52 – 7.46 (m, 2H), 7.45 – 7.38 (m, 2H), 1.32 (s, 9H).

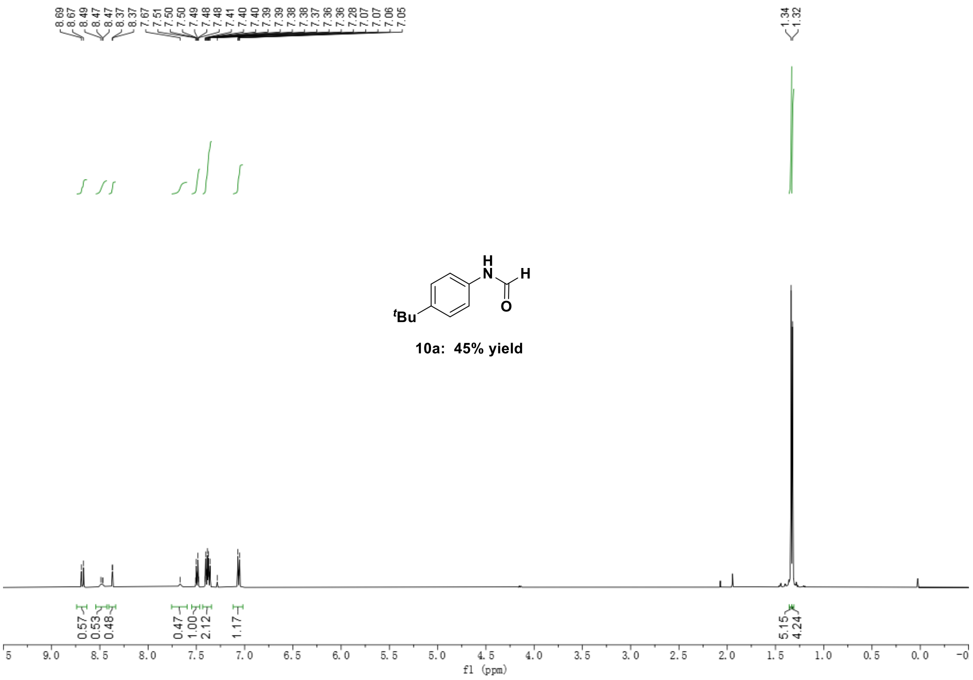

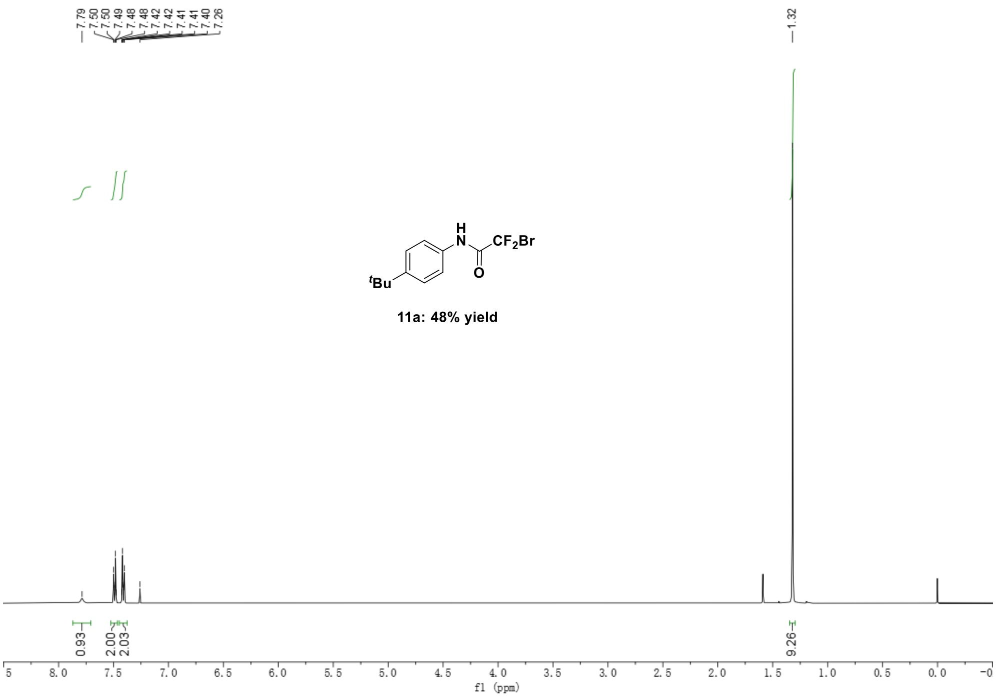

Figure S2 ^{1}H NMR spectra of compound **10a** and compound **11a**

4.3 Competitive experiments of BrCF$_2$CO$_2$K, p-bromoaniline and silyl enol ether

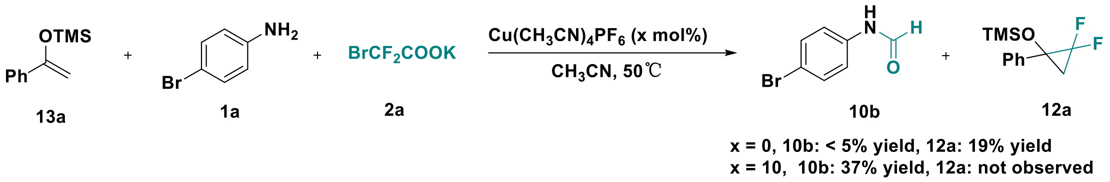

To an oven-dried 10 mL Schlenk tube equipped with a stir bar was added 4-bromoaniline **1a** (17.2mg, 0.1 mmol, 1.0 equiv), BrCF$_2$CO$_2$K (21.3 mg, 0.1 mmol, 1.0 equiv) and Cu(CH$_3$CN)$_4$PF$_6$ (3.5mg, 0.01mmol, 10.0 mol%) under dry argon atmosphere. The solvent of silyl enol ether **13a** (38.4mg, 0.2 mmol, 2.0 equiv) in CH$_3$CN (4.0 mL) was added dropwise to the mixture. The resulting mixture was stirred at 50 °C for 6 hours. Fluorobenzene (19 μL, 0.2 mmol, 1.0 equiv) was added as the internal standard, and the resulting mixture was detected by LC-MS and ^{19}F-NMR. The yield of **10b** and **12a** was determined by LC-MS and ^{19}F-NMR respectively.

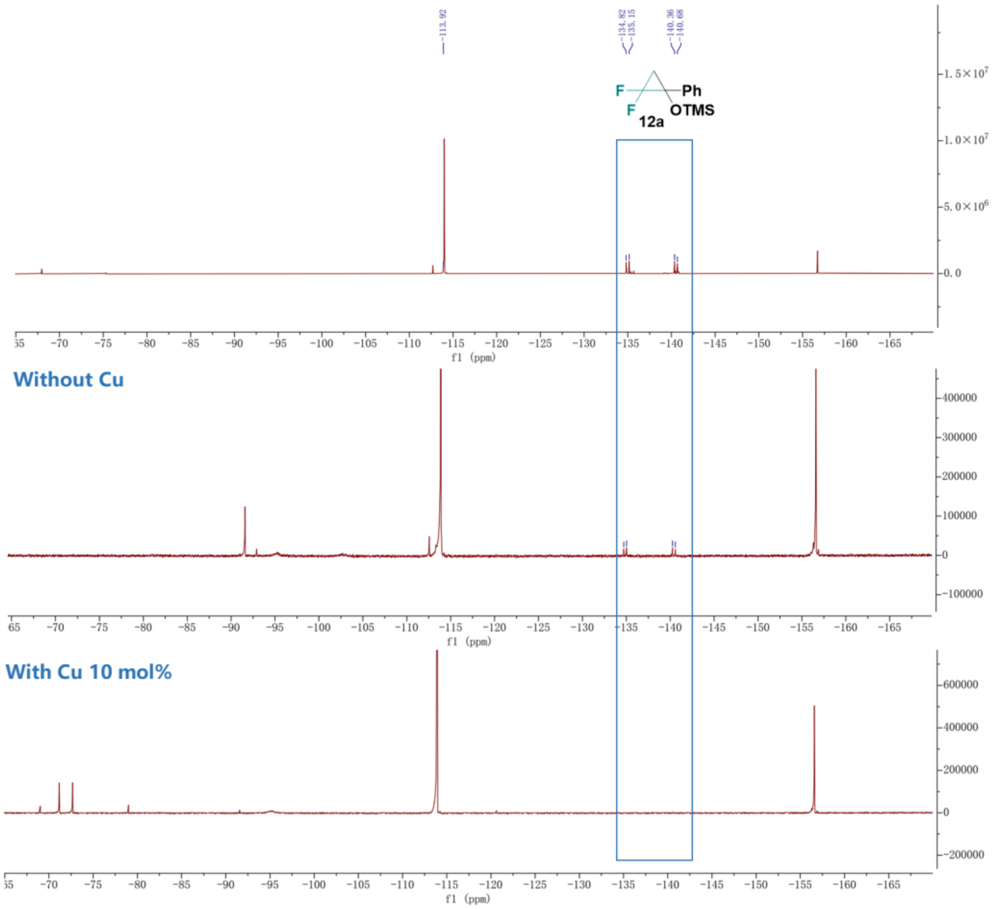

Figure S3 ^{19}F NMR spectrums of difluorocyclopropane and reaction mixture

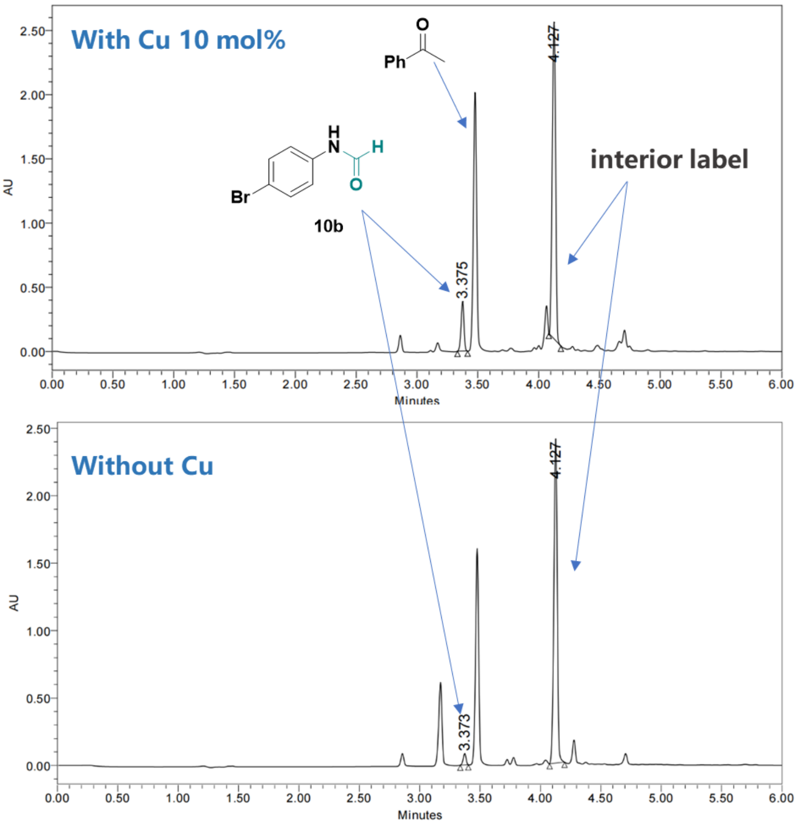

Figure S4 HPLC of reaction mixture

4.4 The reaction of $BrCF_2CO_2K$, p-bromoaniline and benzaldehyde under the conditions of CuCl, 2,9-diMe-1,10-phen, CH_3CN, 50 ℃.

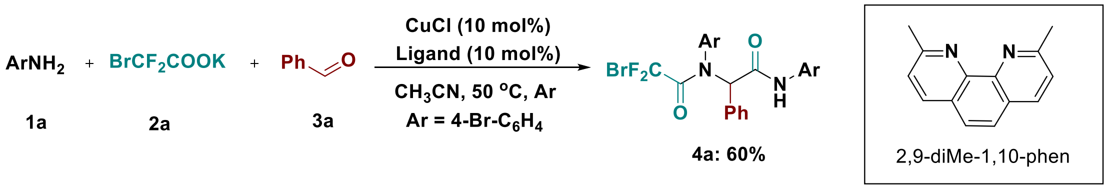

Figure S5 Reaction under the conditions of CuCl, 2,9-diMe-1,10-phen, CH3CN, 50 °C

To an oven-dried 10 mL Schlenk tube equipped with a stir bar was added CuCl (2 mg, 0.02 mmol, 10 mol%), 2,9-diMe-1,10-phen (4.2 mg, 0.02 mmol, 10 mol%), aromatic amines **1** (0.5 mmol, 2.5 equiv), $BrCF_2COOK$ **2a** (128mg, 0.6 mmol, 3.0 equiv), and aldehydes **3a** (0.2 mmol, 1.0 equiv). Anhydrous MeCN (4 mL) was added, resulting suspension liquid. The mixture was stirred at 50℃ for 12 hours. The progress of the reaction was monitored by TLC. After the reaction was complete, the reaction was cooled to room temperature and concentrated under reduced pressure. The residue

was purified by flash column chromatography (eluent: EA:PE = 1/20~1/5) to give the pure product **4a** in 60% yield.

4.5 The template reactions were conducted with different Lewis acid

To an oven-dried 10 mL Schlenk tube equipped with a stir bar was added Lewis acid (0.02mmol, 10.0 mol%), TsOH (6.9mg, 0.04mmol, 20.0 mol%), aromatic amines **1a** (0.5 mmol, 2.5 equiv), $BrCF_2COOK$ **2a** (128mg, 0.6 mmol, 3.0 equiv), and aldehydes **3a** (0.2 mmol, 1.0 equiv). Anhydrous MeCN (4 mL) was added, resulting suspension liquid. The mixture was stirred at 50°C for 12 hours. The progress of the reaction was monitored by TLC. Fluorobenzene (19 µL, 0.2 mmol, 1.0 equiv) was added as the internal standard, and the resulting mixture was detected by LC-MS and ^{19}F-NMR. The yield was determined by LC-MS and ^{19}F-NMR.

Table S5. *Attemption of the* different Lewis acid

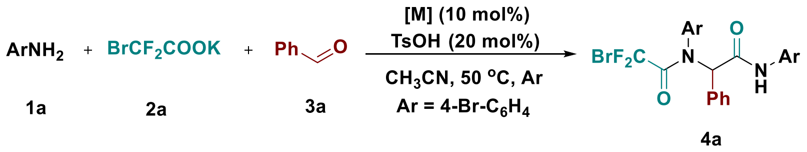

entry	[M]	Yield of 4a
1	$Sc(OTf)_3$	N.D.
2	$Yb(OTf)_3$	N.D.
3	$Zn(OTf)_2$	trace
4	$AgSbF_6$	trace
5	$Ni(OTf)_2$	N.D.

5. DFT computations for the formation of intermediate IV

All the calculations were performed with Gaussian 16 package[2]. Geometries were optimized using DFT with the B3LY[3-5] functional with D3BJ[6,7] dispersion correction and the basis set of def2-SVP[6] at 298.15 K and 1 atm. Frequency computations for all the optimized geometries were given to verified by the minima (zero imaginary frequencies) or transition structures (a single imaginary frequency) point. Intrinsic reaction coordinate (IRC)[7] calculations were performed for all the transition states. Further refining of single point energies with solvent effect was calculated at the B3LYP-D3BJ and def2-TZVP[6,8] level of theory. The solvent effects were performed with SMD[9] model in acetonitrile solvent. The 3D optimized structures were displayed by CYLview visualization program[10].

The two different copper configurations for the formation of ammonium ylide intermediate IV were separately considered for application in our calculation process[11,12], and the results indicated that the Gibbs free energies of copper difluorocarbene $[Cu^{I}]=CF_2$ II and ammonium ylide intermediate IV for four-coordinated configuration are higher than that for two-coordinated configuration (-11.0 *vs* -15.3 kcal/mol; -11.2 *vs* -24.1 kcal/mol). However, we could not obtain the transition state of C-N bond formation for four-coordinated copper configuration. To verify this transition state, potential energy surface scans were performed at 100 different points (Figure S6). The energy fluctuations were found at points 25, 26, 81 and 82, the C-N distances of corresponding the structures are 2.102 Å, 2.123 Å, 3.222 Å and 3.242 Å respectively, which indicate the absence of a C-N bond in the structures at points 81 and 82. For the structures at points 25 and 26, the energy fluctuation are caused by the variation of the dihedral angle between the amine and carbene groups, as the different perspectives were shown below. Thus, the transition state of C-N bond formation via four-coordinated copper configuration could not located, and two-coordinated copper configuration were used in the DFT calculations.

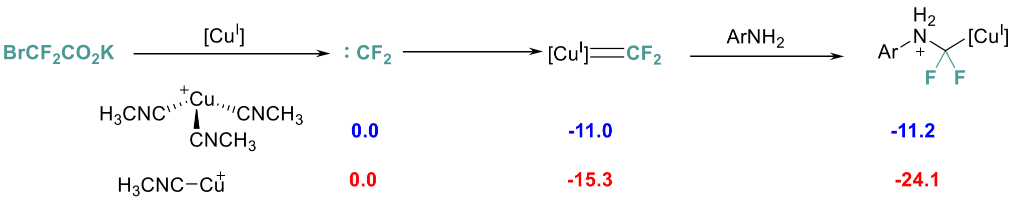

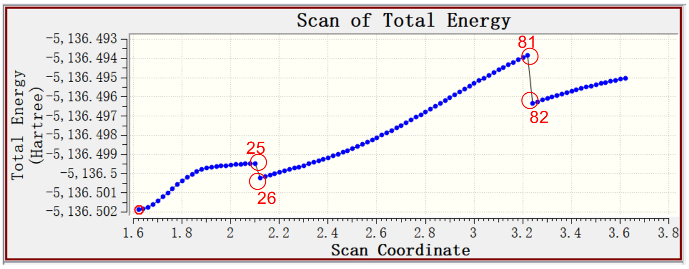

Different perspectives of structures at 25 and 26 points :

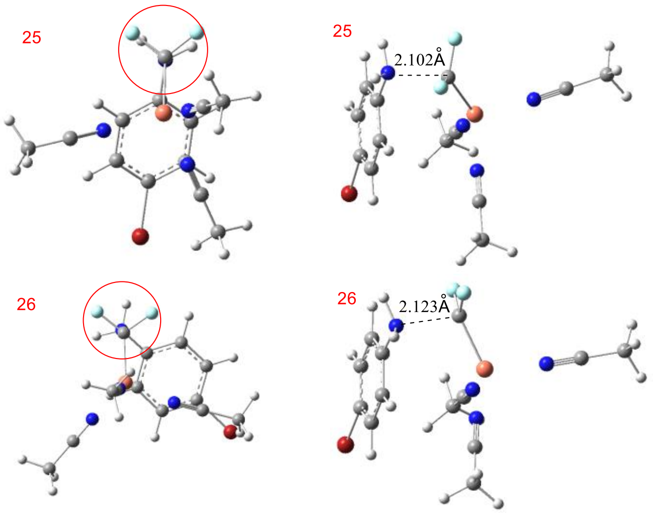

Figure S6. DFT computations for different copper configurations

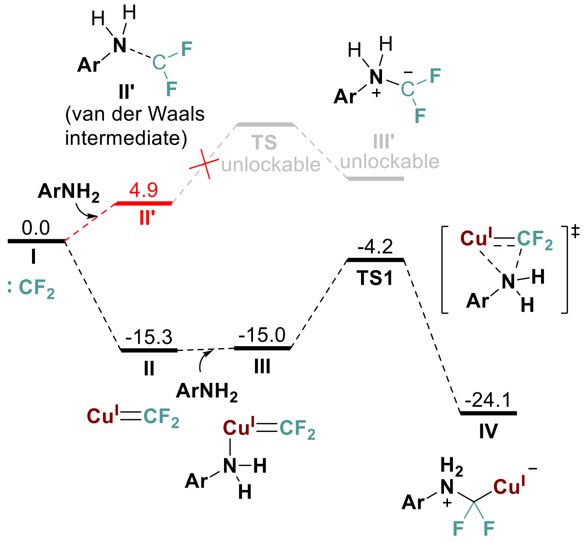

Figure S7. DFT computations for the formation of intermediate **IV**

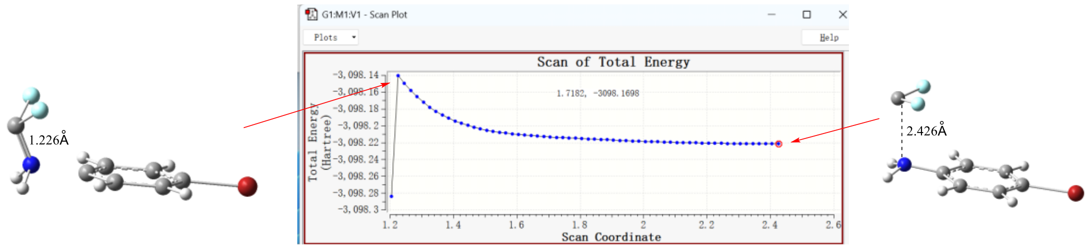

Figure S8. Energy surface scans of **II'**

Density functional theory (DFT) computations were performed to insight into the reaction mechanism of intermediate **IV** formation (Figure. S7). The free difluorocarbene **I** complexed with $[Cu^{I}]^{+}$ to form the copper difluorocarbene **II** (-15.3 kcal/mol), which is more favored than that directly interactions with $ArNH_2$ to form a weakly bound van der Waals intermediate **II'** (4.9 kcal/mol). The aromatic amine ($ArNH_2$) then coordinates to the copper difluorocarbene **II**, providing the intermediate **III** with a lower free energy about -15.0 kcal/mol. Subsequently, the intermediate **III** undergoes the C-N formation via transition state **TS1** (-4.2 kcal/mol), resulting in the stable intermediate **IV** (-24.1 kcal/mol). The DFT calculations suggest that the overall process for formation the intermediate **IV** via copper difluorocarbene is thermodynamically favorable, with an energy release of -24.1 kcal/mol, indicating a thermodynamically favorable pathway. In the absence of copper catalyst, formation of a weakly bound van der Waals intermediate **II'** from free difluorocarbene with $ArNH_2$ was more slowly (4.9 kcal/mol versus -15.3 kcal/mol). Furthermore, attempts to locate a transition state for C–N bond formation from **II'** to generate the free ylide intermediate **III'** were unsuccessful, likely due to a high-energy barrier rendering the process thermodynamically unfavorable. We have performed the C-N bond scan on intermediate III' at 62 different points, and found no energy fluctuations (Figure. S8). Thus, these results rule out the possibility of intermediate **IV** forming directly from free difluorocarbene. The DFT calculations highlight the crucial role of copper(I) in facilitating the transformation, wherein coordination with difluorocarbene generates the copper-difluorocarbene intermediate, followed by C–N bond formation to yield **IV**.

6. Single crystal X-ray diffraction data

Single crystal X-ray diffraction data of 4e (CCDC NO.: 2393043)
(Ellipsoid contour probability levels: 50%)

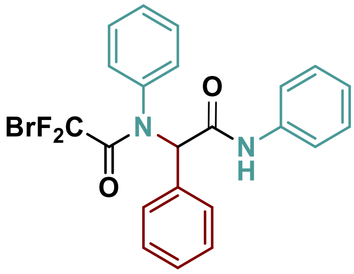

4e

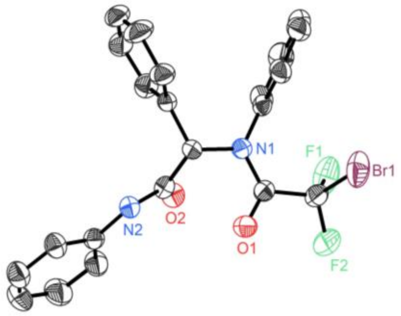

X-ray crystal structure of **4e**

Bond precision: C-C = 0.0104 A Wavelength=0.71073

Cell:	a=14.0485(7)	b=14.1313(7)	c=41.337(2)
	alpha=90	beta=91.792(2)	gamma=90
Temperature:	193 K		

	Calculated	Reported
Volume	8202.4(7)	8202.4(7)
Space group	C c	C 1 c 1
Hall group	C -2yc	C -2yc
Moiety formula	C22 H17 Br F2 N2 O2	C22 H17 Br F2 N2 O2
Sum formula	C22 H17 Br F2 N2 O2	C22 H17 Br F2 N2 O2
Mr	459.28	459.28
Dx,g cm-3	1.488	1.488
Z	16	16
Mu (mm-1)	2.042	2.042
F000	3712.0	3712.0
F000'	3709.21	
h,k,lmax	18,18,54	18,18,53
Nref	19155[9582]	18453
Tmin,Tmax	0.767,0.815	0.610,0.746
Tmin'	0.767	

Correction method= # Reported T Limits: Tmin=0.610 Tmax=0.746
AbsCorr = MULTI-SCAN

Data completeness= 1.93/0.96 Theta(max)= 27.690

R(reflections)= 0.0543(12368) wR2(reflections)= 0.1473(18453)

S = 1.039 Npar= 1083

Figure S9 Crystallographic data of **4e**

Single crystal X-ray diffraction data of 4ap (CCDC NO.: 2393055)
(Ellipsoid contour probability levels: 50%)

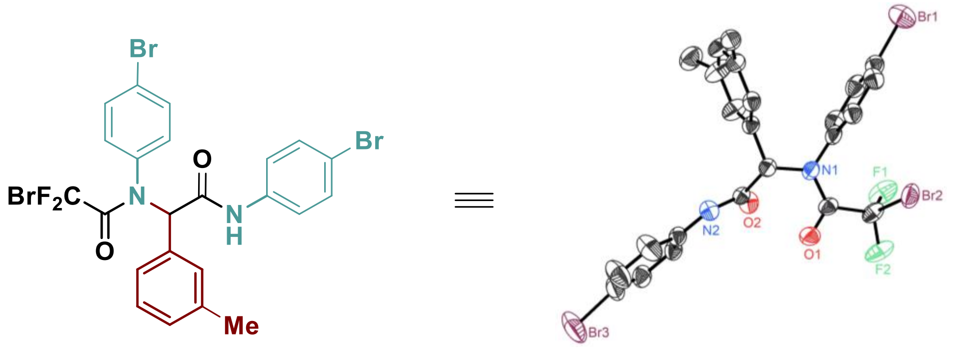

4ap X-ray crystal structure of **4ap**

Bond precision:	C-C = 0.0054 A		Wavelength=0.71073
Cell:	a=21.5503(8)	b=11.4940(3)	c=9.5528(3)
	alpha=90	beta=97.858(1)	gamma=90
Temperature:	200 K		

	Calculated	Reported
Volume	2344.00(13)	2344.00(13)
Space group	P 21/c	P 1 21/c 1
Hall group	-P 2ybc	-P 2ybc
Moiety formula	C23 H17 Br3 F2 N2 O2	C23 H17 Br3 F2 N2 O2
Sum formula	C23 H17 Br3 F2 N2 O2	C23 H17 Br3 F2 N2 O2
Mr	631.09	631.11
Dx,g cm-3	1.788	1.788
Z	4	4
Mu (mm-1)	5.202	5.202
F000	1232.0	1232.0
F000'	1229.24	
h,k,lmax	28,14,12	27,14,12
Nref	5398	5390
Tmin,Tmax	0.514,0.594	0.495,0.746
Tmin'	0.503	

Correction method= # Reported T Limits: Tmin=0.495 Tmax=0.746
AbsCorr = MULTI-SCAN

Data completeness= 0.999 Theta(max)= 27.527

R(reflections)= 0.0518(4218) wR2(reflections)= 0.1204(5390)

S = 1.077 Npar= 355

Figure S10 Crystallographic data of **4ap**

7. Analytical data of products

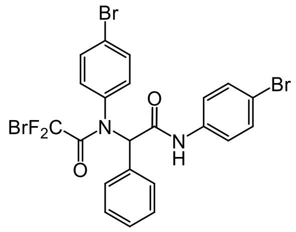

2-bromo-*N*-(4-bromophenyl)-*N*-(2-((4-bromophenyl)amino)-2-oxo-1-phenylethyl)-2,2-difluoroacetamide

4a: 102 mg, white solid, 83% yield.

^{1}H NMR (500 MHz, DMSO) δ 10.55 (s, 1H), 7.82 (dd, *J* = 8.6, 2.6 Hz, 1H), 7.60 (d, *J* = 8.6 Hz, 2H), 7.54 (dd, *J* = 8.5, 2.3 Hz, 1H), 7.51 (d, *J* = 8.6 Hz, 2H), 7.23 (dd, *J* = 8.5, 2.4 Hz, 1H), 7.21 – 7.11 (m, 5H), 6.78 (dd, *J* = 8.3, 2.5 Hz, 1H), 6.15 (s, 1H).

^{13}C NMR (150 MHz, DMSO) δ 167.9, 158.8 (t, J_{CF} = 25.8 Hz), 138.5, 136.0, 134.2, 133.8, 132.3, 132.2, 131.5, 131.2, 131.2, 129.3, 128.8, 122.4, 121.6, 115.7, 111.4 (t, J_{CF} = 314.9 Hz), 67.1.

^{19}F NMR (471 MHz, DMSO) δ -50.6 – -56.2 (m, 2F).

HRMS-ESI: calcd. for $C_{22}H_{16}Br_3F_2N_2O_2$ $[M + H]^+$ 616.8705, found 616.8705.

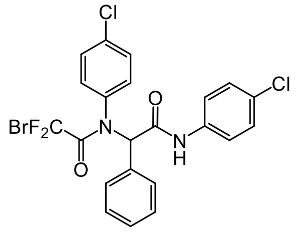

2-bromo-*N*-(4-chlorophenyl)-*N*-(2-((4-chlorophenyl)amino)-2-oxo-1-phenylethyl)-2,2-difluoroacetamide

4b: 82 mg, white solid, 78% yield.

^{1}H NMR (500 MHz, DMSO) δ 10.56 (s, 1H), 7.89 (dd, *J* = 8.6, 2.6 Hz, 1H), 7.72 – 7.60 (m, 2H), 7.46 – 7.32 (m, 3H), 7.17 (tt, *J* = 8.4, 3.6 Hz, 5H), 7.10 (dd, *J* = 8.5, 2.6 Hz, 1H), 6.85 (dd, *J* = 8.8, 2.5 Hz, 1H), 6.16 (s, 1H).

^{13}C NMR (125 MHz, DMSO) δ 167.9, 158.9 (t, J_{CF} = 25.8 Hz), 138.1, 135.5, 133.9, 133.8, 133.5, 132.4, 131.2, 129.2, 128.8, 128.5, 128.2, 127.7, 111.4 (t, J_{CF} = 313.8 Hz), 67.1.

^{19}F NMR (471 MHz, DMSO) δ -45.6 – -61.9 (m, 2F).

HRMS-ESI: calcd. for $C_{22}H_{16}BrCl_2F_2N_2O_2$ $[M + H]^+$ 526.9735, found 526.9731.

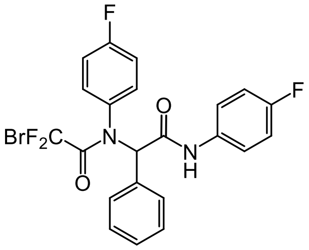

2-bromo-2,2-difluoro-*N*-(4-fluorophenyl)-*N*-(2-((4-fluorophenyl)amino)-2-oxo-1-phenylethyl)acetamide

4c: 74 mg, light yellow solid, 75% yield.

^{1}H NMR (500 MHz, DMSO-d_6) δ 10.46 (s, 1H), 7.93 (td, *J* = 6.2, 5.7, 3.2 Hz, 1H), 7.64 (dd, *J* = 9.0, 4.9 Hz, 2H), 7.17 (ddt, *J* = 8.8, 5.3, 3.0 Hz, 8H), 6.86 (d, *J* = 6.4 Hz, 2H), 6.14 (s, 1H).

^{13}C NMR (125 MHz, DMSO) δ 167.7, 161.9 (d, J_{CF} = 245.0 Hz), 159.0 (t, J_{CF} = 25.7 Hz), 158.6 (d, J_{CF} = 237.5 Hz), 135.5 (d, J_{CF} = 2.5 Hz), 134.4 (d, J_{CF} = 8.8 Hz), 133.9 (d, J_{CF} = 8.8 Hz), 132.9 (d, J_{CF} = 2.5 Hz), 132.7, 131.2, 129.1, 128.7, 121.4 (d, J_{CF} = 8.8 Hz), 115.9 (d, J_{CF} = 21.3 Hz), 115.2 (d, J_{CF} = 22.5 Hz), 115.0 (d, J_{CF} = 22.5 Hz), 111.5 (d, J_{CF} = 312.5 Hz), 67.1.

^{19}F NMR (471 MHz, DMSO-d_6): δ -51.9 – -54.7 (m, 2F), -112.6 (s, 1F), -118.9 (s, 1F).

HRMS-ESI: calcd. for $C_{22}H_{16}BrF_4N_2O_2$ $[M + H]^+$ 495.0326, found 495.0323.

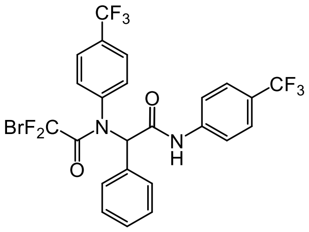

2-bromo-2,2-difluoro-*N*-(2-oxo-1-phenyl-2-((4-(trifluoromethyl)phenyl)amino)ethyl)-*N*-(4-(trifluoromethyl)phenyl)acetamide

4d: 95 mg, white solid, 80% yield.

^{1}H NMR (500 MHz, DMSO) δ 10.83 (s, 1H), 8.09 (d, *J* = 8.4 Hz, 1H), 7.85 (d, *J* = 8.5 Hz, 2H), 7.74 (d, *J* = 8.4 Hz, 1H), 7.70 (d, *J* = 8.6 Hz, 2H), 7.42 (d, *J* = 8.4 Hz, 1H), 7.17 (d, *J* = 2.5 Hz, 5H), 7.10 (d, *J* = 8.4 Hz, 1H), 6.23 (s, 1H).

^{13}C NMR (150 MHz, DMSO): δ 168.3, 158.7 (t, J_{CF} = 25.7 Hz), 142.6, 140.4, 133.2, 132.68, 132.0, 131.2, 129.5 (q, J_{CF} = 33.2 Hz), 129.4, 128.8, 126.7 (q, J_{CF} = 3.0 Hz), 125.7, 125.3, 124.8 (q, J_{CF} = 271.8 Hz), 124.2 (q, J_{CF} = 33.2 Hz), 124.2 (t, J_{CF} = 273.3 Hz), 119.7, 111.4 (t, J_{CF} = 314.8 Hz), 67.2.

^{19}F NMR (471 MHz, DMSO-d_6): δ -52.8 – -54.4 (m, 2F), -60.5 (s, 3F), -61.2 (s, 3F).

HRMS-ESI: calcd. for $C_{24}H_{16}BrF_8N_2O_2$ $[M + H]^+$ 595.0262, found 595.0253.

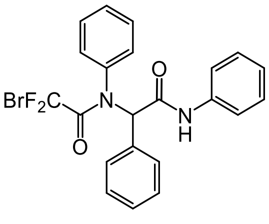

2-bromo-2,2-difluoro-*N*-(2-oxo-1-phenyl-2-(phenylamino)ethyl)-*N*-phenylacetamide

4e: 73 mg, light yellow solid, 80% yield.

^{1}H NMR (500 MHz, DMSO) δ 10.37 (s, 1H), 7.90 (d, *J* = 8.0 Hz, 1H), 7.63 (d, *J* = 8.0 Hz, 2H), 7.32 (t, *J* = 7.8 Hz, 3H), 7.22 – 7.11 (m, 6H), 7.07 (t, *J* = 7.4 Hz, 1H), 7.00 (t, *J* = 7.7 Hz, 1H), 6.80 (d, *J* = 8.0 Hz, 1H), 6.17 (s, 1H);

^{13}C NMR (125 MHz, DMSO) δ 167.8, 159.0 (t, J_{CF} = 25.8 Hz), 139.3, 136.6, 132.8, 132.0, 131.7, 131.2, 129.3, 129.1, 129.0, 128.6, 128.4, 128.1, 124.0, 119.6, 111.6 (t, J_{CF} = 316.3 Hz), 67.2.

^{19}F NMR (471 MHz, DMSO) δ -51.9 – -54.2 (m, 2F).

HRMS-ESI: calcd. for $C_{22}H_{18}BrF_2N_2O_2$ $[M + H]^+$ 459.0515, found 459.0508.

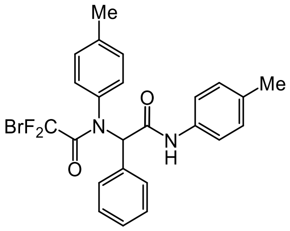

2-bromo-2,2-difluoro-*N*-(2-oxo-1-phenyl-2-(*p*-tolylamino)ethyl)-*N*-(*p*-tolyl)acetamide

4f: 68 mg, light yellow solid, 70% yield.

^{1}H NMR (500 MHz, DMSO) δ 10.25 (s, 1H), 7.77 (dd, *J* = 8.2, 2.2 Hz, 1H), 7.54 – 7.45 (m, 2H), 7.21 – 7.06 (m, 8H), 6.86 – 6.76 (m, 1H), 6.72 – 6.59 (m, 1H), 6.13 (s, 1H), 2.25 (s, 3H), 2.17 (s, 3H).

^{13}C NMR (150 MHz, DMSO) δ 167.6, 159.3, 159.1, 158.9, 138.4, 136.8, 134.0, 133.0, 132.9, 131.7, 131.5, 131.2, 129.6, 128.9, 128.9, 128.6, 128.5, 119.6, 113.7, 111.6, 109.6, 67.2, 21.1, 20.9.

^{19}F NMR (471 MHz, DMSO) δ -48.9 – -57.0 (m, 2F).

HRMS-ESI: calcd. for $C_{24}H_{22}BrF_2N_2O_2$ $[M + H]^+$ 487.0828, found 487.0823.

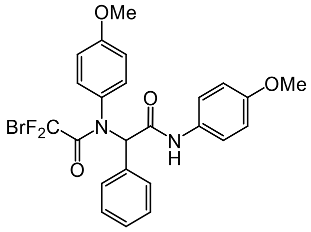

2-bromo-2,2-difluoro-*N*-(4-methoxyphenyl)-*N*-(2-((4-methoxyphenyl)amino)-2-oxo-1-phenylethyl)acetamide

4g: 71 mg, white solid, 68% yield.

^{1}H NMR (500 MHz, $CDCl_3$) δ 7.77 (d, *J* = 8.8 Hz, 1H), 7.51 (s, 1H), 7.32 (d, *J* = 8.8 Hz, 2H), 7.29 – 7.25 (m, 1H), 7.22 (t, *J* = 7.5 Hz, 2H), 7.16 (d, *J* = 7.2 Hz, 2H), 6.78 (d, *J* = 8.7 Hz, 3H), 6.50 (s, 2H), 6.13 (s, 1H), 3.75 (s, 3H), 3.74 (s, 3H).

^{13}C NMR (125 MHz, $CDCl_3$) δ 166.5, 160.1 (t, J_{CF} = 25.8 Hz), 159.6, 156.7, 132.4, 132.1, 130.8, 130.4, 129.3, 129.1, 128.8, 122.1, 114.1, 113.3, 113.2, 111.3 (t, J_{CF} = 317.5 Hz), 68.0, 55.5, 55.3.

^{19}F NMR (471 MHz, $CDCl_3$) δ -50.1 – -54.9 (m, 2F).

HRMS-ESI: calcd. for $C_{24}H_{22}BrF_2N_2O_4$ $[M + H]^+$ 519.0726, found 519.0728.

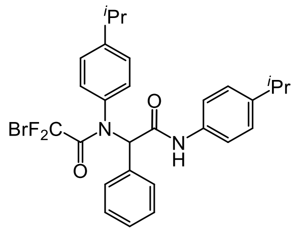

2-bromo-2,2-difluoro-*N*-(4-isopropylphenyl)-*N*-(2-((4-isopropylphenyl)amino)-2-oxo-1-phenylethyl)acetamide

4h: 72 mg, light yellow solid, 66% yield.

^{1}H NMR (500 MHz, DMSO) δ 10.25 (s, 1H), 7.79 (d, J = 7.8 Hz, 1H), 7.57 – 7.48 (m, 2H), 7.21 – 7.15 (m, 3H), 7.12 (dtd, J = 10.5, 5.5, 3.0 Hz, 5H), 6.83 (d, J = 8.4 Hz, 1H), 6.62 (d, J = 8.2 Hz, 1H), 6.11 (s, 1H), 2.84 (p, J = 6.9 Hz, 1H), 2.76 (p, J = 6.9 Hz, 1H), 1.17 (d, J = 6.9 Hz, 6H), 1.07 (dd, J = 7.0, 1.7 Hz, 6H).

^{13}C NMR (125 MHz, DMSO) δ 167.5, 159.0 (t, J_{CF} = 25.6 Hz), 149.2, 144.1, 137.0, 134.3, 133.0, 131.6, 131.5, 131.1, 128.8, 128.4, 127.0, 126.1, 125.8, 119.6, 111.7 (t, J_{CF} = 317.5 Hz), 67.2, 33.3, 24.4, 24.4, 24.1, 24.1.

^{19}F NMR (471 MHz, DMSO-d_6) δ -48.3 – -56.3 (m, 2F).

HRMS-ESI: calcd. for $C_{28}H_{30}BrF_2N_2O_2$ $[M + H]^+$ 543.1454, found 543.1450.

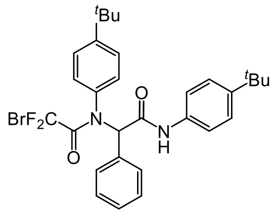

2-bromo-*N*-(4-(*tert*-butyl)phenyl)-*N*-(2-((4-(*tert*-butyl)phenyl)amino)-2-oxo-1-phenylethyl)-2,2-difluoroacetamide

4i: 78 mg, light yellow solid, 68% yield.

^{1}H NMR (500 MHz, DMSO) δ 10.25 (s, 1H), 7.78 (d, J = 8.5 Hz, 1H), 7.52 (d, J = 8.6 Hz, 2H), 7.32 (d, J = 8.5 Hz, 3H), 7.21 – 7.04 (m, 5H), 7.02 – 6.90 (m, 1H), 6.62 (d, J = 8.7 Hz, 1H), 6.11 (s, 1H), 1.26 (s, 9H), 1.16 (s, 9H).

^{13}C NMR (125 MHz, DMSO) δ 167.5, 159.0 (t, J_{CF} = 25.9 Hz), 151.5, 146.3, 136.7, 134.0, 133.0, 131.3, 131.1, 128.7, 128.4, 125.9, 124.9, 124.7, 119.3, 111.7 (t, J_{CF} = 315.6 Hz), 67.2, 34.7, 34.5, 31.7, 31.4.

^{19}F NMR (471 MHz, DMSO) δ -52.1 – -53.9 (m, 2F).

HRMS-ESI: calcd. for $C_{30}H_{34}BrF_2N_2O_2$ $[M + H]^+$ 571.1767, found 571.1763.

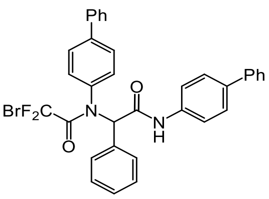

N-([1,1'-biphenyl]-4-yl)-N-(2-([1,1'-biphenyl]-4-ylamino)-2-oxo-1-phenylethyl)-2-bromo-2,2-difluoroacetamide

4j: 100 mg, white solid, 82% yield.

^{1}H NMR (500 MHz, DMSO) δ 10.52 (s, 1H), 8.00 (d, J = 8.3 Hz, 1H), 7.78 – 7.72 (m, 2H), 7.72 – 7.63 (m, 5H), 7.62 – 7.56 (m, 2H), 7.44 (dt, J = 11.0, 7.6 Hz, 4H), 7.35 (q, J = 7.6 Hz, 3H), 7.27 – 7.20 (m, 2H), 7.17 (q, J = 3.1, 2.5 Hz, 3H), 6.88 (d, J = 8.3 Hz, 1H), 6.24 (s, 1H).

^{13}C NMR (125 MHz, DMSO) δ 167.9, 159.1 (t, J_{CF} = 25.6 Hz), 140.3, 140.1, 139.0, 138.7, 136.0, 135.8, 132.8, 132.5, 132.3, 131.3, 129.4, 129.4, 129.1, 128.6, 128.4, 127.6, 127.5, 127.0, 126.8, 126.3, 126.0, 120.0, 111.7 (t, J_{CF} = 315.0 Hz), 67.3.

^{19}F NMR (471 MHz, DMSO) δ -51.9 – -54.1 (m, 2F).

HRMS-ESI: calcd. for $C_{34}H_{26}BrF_2N_2O_2$ $[M + H]^+$ 611.1141, found 611.1135.

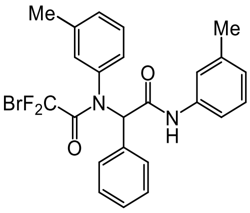

2-bromo-2,2-difluoro-N-(2-oxo-1-phenyl-2-(m-tolylamino)ethyl)-N-(m-tolyl)acetamide

4k: 56 mg, white solid, 57% yield.

^{1}H NMR (500 MHz, DMSO) δ 10.28 (s, 1H), 7.79 – 7.63 (m, 1H), 7.53 (s, 1H), 7.35 (d, J = 8.1 Hz, 1H), 7.21 – 7.15 (m, 6.5H), 6.97 (d, J = 7.5 Hz, 1H), 6.90 – 6.86 (m, 1.5H), 6.65 – 6.49 (m, 1H), 6.13 (s, 1H), 2.29 (s, 3H), 2.27 (s, 1.5H), 1.98 (s, 1.5H).

^{13}C NMR (150 MHz, DMSO) δ 167.7 (167.7), 158.9 (t, J_{CF} = 25.7 Hz), 139.2, 138.6, 137.6 (137.3), 136.5, 132.9 (132.8), 132.5 (132.2), 131.3 (131.2), 129.5, 129.1, 128.9, 128.7, 128.5 (128.4), 128.1 (127.8), 124.7, 120.1, 116.7, 111.7 (t, J_{CF} = 314.1 Hz), 67.3, 21.6, 21.3 (20.8).

^{19}F NMR (471 MHz, DMSO) δ -52.1 – -53.6 (m, 2F).

HRMS-ESI: calcd. for $C_{24}H_{22}BrF_2N_2O_2$ $[M + H]^+$ 487.0828, found 487.0823.

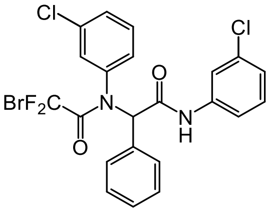

2-bromo-N-(3-chlorophenyl)-N-(2-((3-chlorophenyl)amino)-2-oxo-1-phenylethyl)-2,2-difluoroacetamide

4l: 74 mg, white solid, 70% yield.

^{1}H NMR (500 MHz, DMSO-d_6) δ 10.65 (d, J = 16.9 Hz, 1H), 8.00 – 7.86 (m, 1.5H), 7.82 (d, J = 8.0 Hz,

0.5H), 7.48 – 7.39 (m, 1H), 7.35 (ddt, J = 8.1, 5.6, 2.7 Hz, 1.5H), 7.26 (ddd, J = 10.6, 7.9, 2.1 Hz, 1H), 7.22 – 7.12 (m, 6H), 7.06 (t, J = 8.0 Hz, 0.5H), 6.97 (s, 0.5H), 6.84 (d, J = 8.0 Hz, 0.5H), 6.15 (d, J = 15.3 Hz, 1H).

^{13}C NMR (125 MHz, DMSO) δ 168.2 (168.1), 158.8 (t, J_{CF} = 25.2 Hz) (158.7 (t, J_{CF} = 25.2 Hz)), 140.6 (140.5), 137.9 (137.8), 133.7, 132.5 (132.3), 132.2 (132.1), 132.1, 131.5, 131.2 (131.2), 131.1, 130.5, 129.9 (129.7), 129.3, 129.3 (129.2), 128.8 (128.7), 123.9 (123.8), 119.2 (119.1), 118.1 (118.0), 111.4 (t, J_{CF} = 316.3 Hz) (111.3 (t, J_{CF} = 316.3 Hz)), 67.1 (67.10).

^{19}F NMR (471 MHz, DMSO) δ -50.4 – -56.1 (m, 2F).

HRMS-ESI: calcd. for $C_{22}H_{16}BrCl_2F_2N_2O_2$ [M + H]$^+$ 526.9735, found 526.9735.

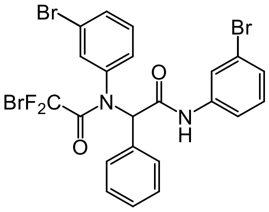

2-bromo-*N*-(3-bromophenyl)-*N*-(2-((3-bromophenyl)amino)-2-oxo-1-phenylethyl)-2,2-difluoroacetamide

4m: 86 mg, light yellow solid, 70% yield.

^{1}H NMR (500 MHz, $CDCl_3$) δ 8.15 (d, J = 7.7 Hz, 0.5H), 7.95 – 7.68 (m, 2.5H), 7.37 (d, J = 8.1 Hz, 1H), 7.31 – 7.13 (m, 7.5H), 7.08 (q, J = 7.6 Hz, 1H), 6.88 (t, J = 8.1 Hz, 0.5H), 6.76 (s, 0.5H), 6.57 (d, J = 8.0 Hz, 0.5H), 6.17 (d, J = 9.1 Hz, 1H).

^{13}C NMR (150 MHz, DMSO) δ 168.2 (168.0), 158.8 (t, J_{CF} = 25.7 Hz) (158.7 (t, J_{CF} = 25.7 Hz)), 140.7 (140.6), 138.0 (137.9), 135.1, 134.4, 132.2 (132.1), 132.1, 131.4 (131.4), 131.2 (131.2), 130.8, 130.1 (130.0), 129.3, 128.8 (128.7), 126.8 (126.7), 122.2, 122.0 (122.0), 120.7 (120.5), 118.5 (118.4), 111.4 (t, J_{CF} = 314.8 Hz) (111.3 (t, J_{CF} = 314.8 Hz)), 67.1.

^{19}F NMR (471 MHz, DMSO) δ -49.9 – -56.3 (m, 2F).

HRMS-ESI: calcd. for $C_{22}H_{16}Br_3F_2N_2O_2$ [M + H]$^+$ 616.8705, found 616.8711.

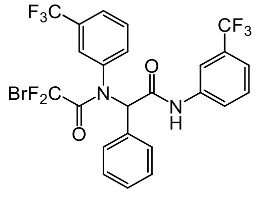

2-bromo-2,2-difluoro-*N*-(2-oxo-1-phenyl-2-((3-(trifluoromethyl)phenyl)amino)ethyl)-*N*-(3-(trifluoromethyl)phenyl)acetamide

4n: 77 mg, light yellow solid, 65% yield.

^{1}H NMR (500 MHz, DMSO) δ 10.84 (d, J = 10.9 Hz, 1H), 8.24 – 8.05 (m, 2H), 7.85 (d, J = 8.3 Hz, 0.5H), 7.78 (d, J = 8.3 Hz, 0.5H), 7.57 (tt, J = 11.8, 5.7 Hz, 2.5H), 7.45 (d, J = 7.8 Hz, 1H), 7.29 (t, J = 7.9 Hz, 0.5H), 7.25 – 7.09 (m, 6H), 6.22 (s, 1H).

^{13}C NMR (125 MHz, DMSO) δ 168.4 (168.3), 158.8 (t, J_{CF} = 27.7) (158.8 (t, J_{CF} = 27.7)), 139.9 (139.78), 137.3, 136.5, 135.9, 132.1 (132.0), 131.2 (131.1), 130.7 (130.7), 130.5-121.0 (Within this range, carbons appear as broad multiplets due to the influence of rotational isomerism and trifluoromethyl, which are not been reported), 120.6 (q, J_{CF} = 3.8), 115.6, 111.3 (t, J_{CF} = 315.0 Hz) (111.2 (t, J_{CF} = 315.0 Hz)), 67.2

(67.1).

^{19}F NMR (471 MHz, DMSO-d_6): δ -52.9 – -54.6 (m, 2F), -61.4 (d, J = 7.9 Hz, 3F), -61.6 (dd, J = 73.9, 6.6 Hz, 3F).

HRMS-ESI: calcd. for $C_{24}H_{16}BrF_8N_2O_2$ $[M + H]^+$ 595.0262, found 595.0258.

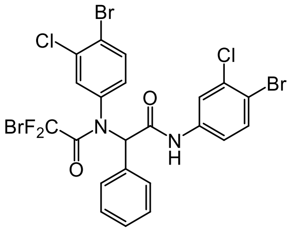

2-bromo-*N*-(4-bromo-3-chlorophenyl)-*N*-(2-((4-bromo-3-chlorophenyl)amino)-2-oxo-1-phenylethyl)-2,2-difluoroacetamide

4q: 55 mg, white solid, 40% yield.

^{1}H NMR (500 MHz, DMSO) δ 10.78 (d, J = 13.5 Hz, 1H), 8.12 – 8.01 (m, 2H), 7.72 (dd, J = 14.0, 8.3 Hz, 2H), 7.48 (d, J = 8.6 Hz, 1H), 7.41 (ddd, J = 15.9, 8.7, 2.5 Hz, 1H), 7.27 – 7.21 (m, 3H), 7.19 (dd, J = 6.6, 3.3 Hz, 3H), 6.85 – 6.76 (m, 1H), 6.14 (d, J = 13.0 Hz, 1H);

^{13}C NMR (150 MHz, DMSO): δ 168.3 (168.1), 158.7 (t, J_{CF} = 25.7 Hz) (158.6 (t, J_{CF} = 25.7 Hz)), 139.7 (139.6), 137.1, 134.5, 134.1, 133.7, 133.5, 133.4 (133.3), 132.8 (132.7), 132.6, 132.1, 131.8 (131.8), 131.2 (131.2), 129.5, 129.0 (128.9), 122.6 (122.5), 120.9 (120.8), 120.0 (119.9), 115.5 (115.4), 111.2 (t, J_{CF} = 314.8 Hz) (111.1 (t, J_{CF} = 314.8 Hz)), 67.0 (67.0).

^{19}F NMR (471 MHz, DMSO-d_6) δ -52.9 – -55.1 (m, 2F).

HRMS-ESI: calcd. for $C_{22}H_{14}Br_3Cl_2F_2N_2O_2$ $[M + H]^+$ 684.7925, found 684.7919.

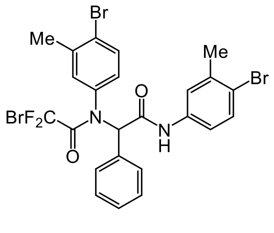

2-bromo-*N*-(4-bromo-3-methylphenyl)-*N*-(2-((4-bromo-3-methylphenyl)amino)-2-oxo-1-phenylethyl)-2,2-difluoroacetamide

4r: 99 mg, light yellow solid, 77% yield.

^{1}H NMR (500 MHz, DMSO-d_6) δ 10.46 (s, 1H), 7.82 (s, 1H), 7.67 (d, J = 2.7 Hz, 1H), 7.52 (dd, J = 14.0, 8.5 Hz, 2H), 7.40 – 7.32 (m, 1H), 7.17 (td, J = 9.8, 8.7, 3.7 Hz, 6H), 6.77 (s, 1H), 6.58 (dd, J = 8.6, 2.5 Hz, 1H), 6.12 (d, J = 6.5 Hz, 1H), 2.32 (s, 3H), 2.30 (s, 1H), 2.02 (s, 1H);

^{13}C NMR (125 MHz, DMSO) δ 167.9 (167.8), 158.8 (t, J_{CF} = 25.2 Hz), 138.7, 138.1, 137.4 (137.1), 136.0, 134.5 (134.1), 132.8, 132.4 (132.4), 132.0 (131.8), 131.3 (131.0), 131.2 (131.2), 129.2, 128.7 (128.6), 124.7 (124.6), 121.9, 119.1 (119.1), 118.2, 111.4 (t, J_{CF} = 315.0 Hz), 67.1, 23.1, 22.8 (22.3).

^{19}F NMR (471 MHz, DMSO-d_6) δ -52.0 – -54.8 (m, 2F).

HRMS-ESI: calcd. for $C_{24}H_{20}Br_3F_2N_2O_2$ $[M + H]^+$ 644.9018, found 644.9014.

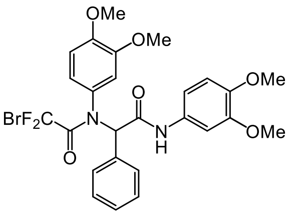

2-bromo-*N*-(3,4-dimethoxyphenyl)-*N*-(2-((3,4-dimethoxyphenyl)amino)-2-oxo-1-phenylethyl)-2,2-difluoroacetamide

4s: 58 mg, white solid, 50% yield.

^{1}H NMR (500 MHz, DMSO) δ 10.19 (d, *J* = 18.1 Hz, 1H), 7.48 (d, *J* = 2.4 Hz, 0.4H), 7.41 (dd, *J* = 8.6, 2.4 Hz, 0.6H), 7.29 (d, *J* = 2.4 Hz, 0.6H), 7.26 – 7.12 (m, 6.4H), 6.93 – 6.84 (m, 1.6H), 6.57 (d, *J* = 8.6 Hz, 0.4H), 6.38 (dd, *J* = 8.6, 2.3 Hz, 0.4H), 6.30 (d, *J* = 2.4 Hz, 0.6H), 6.08 (d, *J* = 2.5 Hz, 1H), 3.77 – 3.73 (m, 4.3H), 3.72 (s, 3H), 3.69 (s, 1.8H), 3.63 (s, 1.2H), 3.37 (s, 1.7H).

^{13}C NMR (125 MHz, DMSO) δ 167.4 (167.3), 159.2 (t, J_{CF} = 25.2 Hz), 149.1, 149.0, 147.8 (147.6), 145.5 (145.4), 133.2 (133.1), 133.0 (132.9), 131.4 (131.1), 129.0, 129.0, 128.9, 128.9, 128.5, 128.5, 124.6 (124.4), 116.0 (115.7), 111.7 (t, J_{CF} = 315.6 Hz) (111.6 (t, J_{CF} = 315.6 Hz)), 112.6, 111.6 (111.3), 110.3, 104.6 (104.5), 67.5 (67.1), 56.2 (55.8), 56.1 (55.9), 55.9 (55.8).

^{19}F NMR (471 MHz, DMSO) δ -50.2 – -57.0 (m, 2F).

HRMS-ESI: calcd. for $C_{26}H_{26}BrF_2N_2O_6$ $[M + H]^+$ 579.0937, found 579.0936.

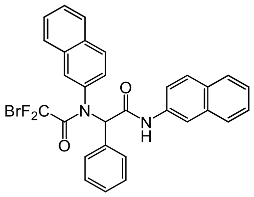

2-bromo-2,2-difluoro-*N*-(naphthalen-2-yl)-*N*-(2-(naphthalen-2-ylamino)-2-oxo-1-phenylethyl)acetamide

4t: 73 mg, white solid, 65% yield.

^{1}H NMR (500 MHz, CD_3OD) δ 11.46 (d, *J* = 21.1 Hz, 1H), 9.45 – 9.18 (m, 1.6H), 8.90 – 8.77 (m, 1H), 8.68 (dt, *J* = 21.6, 7.3 Hz, 4H), 8.62 – 8.58 (m, 0.5H), 8.45 (d, *J* = 8.2 Hz, 0.5H), 8.40 (dd, *J* = 8.8, 2.1 Hz, 1H), 8.38 – 8.27 (m, 3.6H), 8.24 (q, *J* = 7.4, 6.0 Hz, 1.5H), 8.13 – 8.01 (m, 2H), 7.88 (dd, *J* = 8.2, 4.6 Hz, 2.5H), 7.82 (d, *J* = 7.3 Hz, 0.5H), 7.72 (d, *J* = 8.7 Hz, 0.5H), 7.13 (d, *J* = 25.0 Hz, 1H);

^{13}C NMR (125 MHz, DMSO) δ 168.2 (168.1), 159.2 (t, J_{CF} = 25.8 Hz) (159.2 (t, J_{CF} = 25.8 Hz)), 136.8 (136.8), 134.3 (134.0), 133.9, 132.8 (132.6), 132.6 (132.5), 132.3 (132.2), 131.4, 131.2 (131.0), 130.4, 129.4 (129.1), 129.0, 128.7 (128.5), 128.6, 128.3, 128.0, 127.9, 127.9, 127.6, 127.5, 127.0, 126.9, 125.3, 120.2, 115.9 (115.9), 111.6 (t, J_{CF} = 315.0 Hz) (111.54 (t, J_{CF} = 315.0 Hz)), 67.8 (67.2);

^{19}F NMR (471 MHz, DMSO-d_6) δ -48.9 – -58.3 (m, 2F).

HRMS-ESI: calcd. for $C_{30}H_{22}BrF_2N_2O_2$ $[M + H]^+$ 559.0828, found 559.0828.

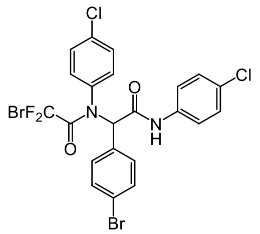

2-bromo-*N*-(1-(4-bromophenyl)-2-((4-chlorophenyl)amino)-2-oxoethyl)-*N*-(4-chlorophenyl)-2,2-difluoroacetamide

4u: 91 mg, white solid, 75% yield.

^{1}H NMR (500 MHz, DMSO) δ 10.57 (s, 1H), 7.96 – 7.83 (m, 1H), 7.76 – 7.59 (m, 2H), 7.51 – 7.33 (m, 5H), 7.18 (dd, *J* = 8.5, 2.6 Hz, 1H), 7.13 (d, *J* = 8.2 Hz, 2H), 6.98 – 6.84 (m, 1H), 6.14 (s, 1H).

^{13}C NMR (125 MHz, DMSO) δ 167.6, 158.9 (t, J_{CF} = 25.8 Hz), 137.9, 135.5, 134.0, 133.5, 133.3, 131.9, 131.8, 129.3, 128.7, 128.4, 127.9, 122.8, 121.3, 111.4 (t, J_{CF} = 315.0 Hz), 66.3.

^{19}F NMR (471 MHz, DMSO) δ -51.5 – -55.2 (m, 2F).

HRMS-ESI: calcd. for $C_{22}H_{15}Br_2Cl_2F_2N_2O_2$ [M + H]$^+$ 606.8820, found 606.8810.

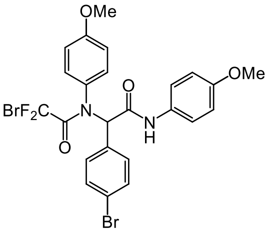

2-bromo-*N*-(1-(4-bromophenyl)-2-((4-methoxyphenyl)amino)-2-oxoethyl)-2,2-difluoro-*N*-(4-methoxyphenyl)acetamide

4v: 72 mg, light yellow solid, 60% yield.

^{1}H NMR (500 MHz, DMSO) δ 10.20 (s, 1H), 7.78 (dd, *J* = 8.9, 2.5 Hz, 1H), 7.56 – 7.47 (m, 2H), 7.42 – 7.34 (m, 2H), 7.15 – 7.07 (m, 2H), 6.95 – 6.83 (m, 3H), 6.73 (dd, *J* = 8.9, 2.6 Hz, 1H), 6.61 (dd, *J* = 8.8, 3.0 Hz, 1H), 6.07 (s, 1H), 3.72 (s, 3H), 3.68 (s, 3H).

^{13}C NMR (150 MHz, DMSO): δ 167.0, 159.4, 159.2 (t, J_{CF} = 25.7 Hz), 155.9, 133.3, 133.2, 132.9, 132.7, 132.3, 131.6, 129.0, 122.4, 121.2, 114.4, 113.5, 113.4, 111.6 (t, J_{CF} = 314.8 Hz), 66.37, 55.70, 55.66.

^{19}F NMR (471 MHz, DMSO-*d*$_6$): δ -52.13 – -53.85 (m, 2F).

HRMS-ESI: calcd. for $C_{24}H_{21}Br_2F_2N_2O_4$ [M + H]$^+$ 598.9811, found 598.9804.

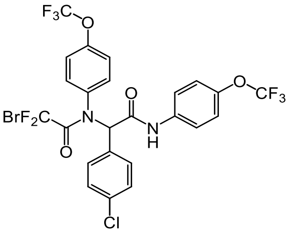

2-bromo-*N*-(1-(4-chlorophenyl)-2-oxo-2-((4-(trifluoromethoxy)phenyl)amino)ethyl)-2,2-difluoro-*N*-(4-(trifluoromethoxy)phenyl)acetamide

4w: 103 mg, white solid, 78% yield.

^{1}H NMR (500 MHz, DMSO) δ 10.66 (s, 1H), 7.97 (dd, *J* = 8.7, 2.6 Hz, 1H), 7.73 (d, *J* = 9.1 Hz, 2H), 7.35 (t, *J* = 9.4 Hz, 3H), 7.24 (d, *J* = 8.6 Hz, 2H), 7.18 (d, *J* = 8.6 Hz, 2H), 7.10 (dd, *J* = 9.1, 2.8 Hz, 1H), 7.01 (dd, *J* = 8.9, 2.5 Hz, 1H), 6.16 (s, 1H).

^{13}**C NMR** (125 MHz, DMSO) δ 167.7, 159.0, 158.8, 158.6, 148.5, 144.4, 138.2, 135.6, 134.3, 134.1, 133.7, 133.0, 131.4, 128.7, 121.6 (q, J_{CF} = 258.8 Hz,), 121.3 (q, J_{CF} = 257.0 Hz,), 122.2, 121.1, 120.9, 111.4 (t, J_{CF} = 314.4 Hz,), 66.3.
^{19}**F NMR** (471 MHz, DMSO) δ -52.5 – -54.5 (m, 2F), -57.1 – -57.2 (m, 3F), -57.2 – -57.2 (m, 3F).
HRMS-ESI: calcd. for $C_{24}H_{15}BrClF_8N_2O_4$ $[M + H]^+$ 660.9771, found 660.9766.

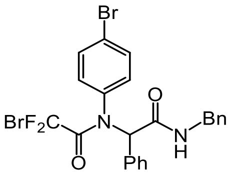

N-(2-(benzylamino)-2-oxo-1-phenylethyl)-2-bromo-N-(4-bromophenyl)-2,2-difluoroacetamide
4x: 40 mg, white solid, 36% yield.
^{1}**H NMR** (600 MHz, DMSO) δ 10.58 (s, 0.5H), 10.43 (s, 0.5H), 7.53 (td, *J* = 27.4, 26.9, 8.6 Hz, 4H), 7.43 – 7.17 (m, 4H), 7.17 – 7.01 (m, 4H), 6.94 (d, *J* = 7.3 Hz, 1H), 6.73 (d, *J* = 6.1 Hz, 1H), 6.30 (s, 0.5H), 5.94 (s, 0.5H), 5.02 (d, *J* = 15.9 Hz, 0.5H), 4.95 (d, *J* = 18.2 Hz, 0.5H), 4.69 (d, *J* = 18.0 Hz, 0.5H), 4.35 (d, *J* = 16.0 Hz, 0.5H).
^{13}**C NMR** (150 MHz, DMSO) δ 167.82 (167.64), 160.83 (t, J_{CF} = 26.0 Hz) (159.95 (t, J_{CF} = 26.6 Hz)), 138.46 (138.12), 137.39 (137.03), 133.79, 132.24 (132.09), 130.98 (130.16), 129.52 (129.23), 128.59 (128.08), 128.08, 126.91 (126.33), 126.83, 121.88 (121.62), 116.07 (115.67), 111.13 (t, J_{CF} = 314.1 Hz) (110.98 (t, J_{CF} = 311.1 Hz)), 64.90 (64.29), 50.34 (49.97).
^{19}**F NMR** (471 MHz, DMSO) δ -54.45 (dd, *J* = 161.7, 48.8 Hz, 1F), -56.78 (dd, *J* = 161.7, 117.6 Hz, 1F).
HRMS-ESI: calcd. for $C_{23}H_{18}Br_2F_2N_2O_2$ $[M + H]^+$ 550.9776, found 550.9785.

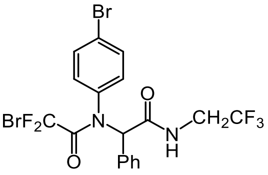

2-bromo-N-(4-bromophenyl)-2,2-difluoro-N-(2-oxo-1-phenyl-2-((2,2,2-trifluoroethyl)amino)ethyl)acetamide
4y: 35 mg, white solid, 32% yield.
^{1}**H NMR** (600 MHz, DMSO) δ 10.62 (s, 1H), 7.54 (q, *J* = 9.0 Hz, 4H), 7.49 (d, *J* = 7.8 Hz, 3H), 7.46 (d, *J* = 7.3 Hz, 2H), 6.28 (s, 1H), 4.55 (dq, *J* = 19.2, 10.1, 9.5 Hz, 1H), 4.22 – 3.97 (m, 1H).
^{13}**C NMR** (150 MHz, DMSO) δ 167.4, 160.7 (t, J_{CF} = 27.5 Hz), 137.9, 133.6, 132.3, 130.1, 129.7, 123.8 (q, J_{CF} = 282.4 Hz), 121.9, 116.3, 110.3 (t, J_{CF} = 310.2 Hz), 64.2, 47.6 (q, J_{CF} = 34.9 Hz).
^{19}**F NMR** (471 MHz, DMSO) δ -55.88 (d, *J* = 167.7 Hz, 1F), -57.14 (d, *J* = 167.7 Hz, 1F), -65.53 (s, 3F).
HRMS-ESI: calcd. for $C_{18}H_{13}Br_2F_5N_2O_2$ $[M + H]^+$ 542.9337, found 542.9346.

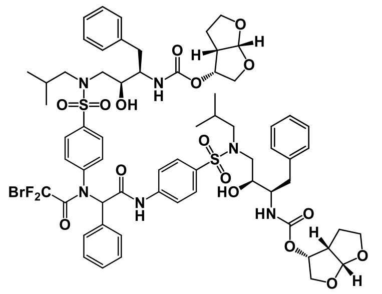

(3*R*,3a*S*,6a*R*)-hexahydrofuro[2,3-*b*]furan-3-yl ((2*R*,3*S*)-4-((4-(2-bromo-2,2-difluoro-N-(2-((4-(N-((2*S*,3*R*)-3-(((((3*R*,3a*S*,6a*R*)-hexahydrofuro[2,3-*b*]furan-3-yl)oxy)carbonyl)amino)-2-hydroxy-4-phenylbutyl)-*N*-isobutylsulfamoyl)phenyl)amino)-2-oxo-1-phenylethyl)acetamido)-*N*-isobutylphenyl)sulfonamido)-3-hydroxy-1-phenylbutan-2-yl)carbamate

4z: 151 mg, light yellow solid, 55% yield.

^{1}H NMR (600 MHz, $CDCl_3$) δ 8.63 (s, 1H), 8.19 (s, 1H), 7.87 – 7.58 (m, 5H), 7.41 (t, J = 10.6 Hz, 1H), 7.28 (q, J = 9.2 Hz, 4H), 7.23 – 7.13 (m, 7H), 7.08 (dd, J = 17.3, 7.5 Hz, 2H), 6.73 (s, 1H), 6.26 (d, J = 8.3 Hz, 1H), 5.63 (dd, J = 11.5, 5.6 Hz, 2H), 5.28 (q, J = 8.6, 6.4 Hz, 2H), 5.09 – 4.79 (m, 2H), 4.03 – 3.79 (m, 8H), 3.78 – 3.48 (m, 6H), 3.09 (d, J = 13.2 Hz, 6H), 2.93 – 2.65 (m, 8H), 2.20 – 1.77 (m, 4H), 1.62 (h, J = 10.9 Hz, 2H), 1.44 – 1.28 (m, 2H), 0.90 (d, J = 6.9 Hz, 6H), 0.84 (t, J = 9.1 Hz, 6H).

^{13}C NMR (150 MHz, $CDCl_3$): δ 167.2 (167.2), 159.5 (t, J_{CF} = 26.4 Hz) (159.5 (t, J_{CF} = 26.4 Hz)), 155.7 (155.6), 155.6 (155.6), 141.8, 140.2 (140.1), 139.1, 138.8, 137.8, 137.7, 133.2 (133.1), 132.7, 132.1, 130.9, 130.7, 129.8, 129.4 (129.3), 129.1 (129.0), 128.6 (128.5), 127.5, 127.0, 126.7 (126.6), 126.6, 119.7 (119.6), 110.8 (t, J_{CF} = 315.6 Hz), 109.4 (109.3), 73.5, 73.5 (73.4), 72.7, 72.7 (72.6), 71.1 (71.0), 70.9 (70.9), 69.7, 69.6, 67.6, 58.6 (58.5), 58.1, 57.8, 55.4, 53.5 (53.5), 53.0 (52.8), 45.6 (45.5), 35.8, 35.7 (35.4), 29.3, 27.2, 27.1 (27.1), 26.9, 25.9 (25.8), 25.9, 20.1 (20.1), 20.0 (19.9);

^{19}F NMR (471 MHz, $CDCl_3$): δ -50.4 – -55.3 (m, 2F).

HRMS-ESI: calcd. for $C_{64}H_{78}BrF_2N_6O_{16}S_2$ $[M + H]^+$ 1367.4062, found 1367.4084.

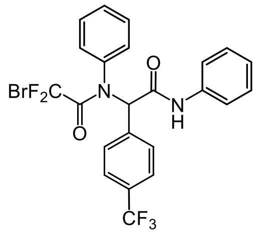

2-bromo-2,2-difluoro-*N*-(2-oxo-2-(phenylamino)-1-(4-(trifluoromethyl)phenyl)ethyl)-*N*-phenylacetamide

4aa: 76 mg, light yellow solid, 72% yield.

^{1}H NMR (500 MHz, DMSO) δ 10.43 (s, 1H), 7.89 (d, J = 8.0 Hz, 1H), 7.65 – 7.58 (m, 2H), 7.53 (d, J = 8.1 Hz, 2H), 7.41 (d, J = 8.1 Hz, 2H), 7.33 (td, J = 7.8, 3.8 Hz, 3H), 7.20 (tt, J = 7.5, 1.3 Hz, 1H), 7.12 – 7.07 (m, 1H), 7.04 (t, J = 7.8 Hz, 1H), 6.86 (d, J = 7.9 Hz, 1H), 6.25 (s, 1H);

^{13}C NMR (150 MHz, DMSO) δ 167.1, 159.0 (t, J_{CF} = 25.7 Hz), 139.0, 137.6, 136.5, 132.1, 132.0, 131.6, 129.3 (q, J_{CF} = 31.7 Hz), 129.3, 129.3, 128.6, 128.3, 124.3 (q, J_{CF} = 271.8 Hz), 125.4 (q, J_{CF} = 3.0 Hz), 124.2, 119.7, 111.5 (t, J_{CF} = 314.8 Hz), 66.6;

^{19}F NMR (471 MHz, DMSO) δ -51.9 – -54.4 (m, 2F), -61.3 (s, 3F).

HRMS-ESI: calcd. for $C_{23}H_{17}BrF_5N_2O_2$ $[M + H]^+$ 527.0389, found 527.0381.

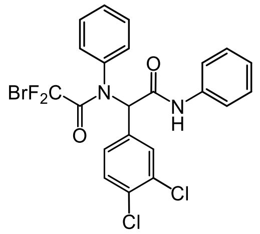

2-bromo-*N*-(1-(3,4-dichlorophenyl)-2-oxo-2-(phenylamino)ethyl)-2,2-difluoro-*N*-phenylacetamide

4ab: 63 mg, white solid, 60% yield.

^{1}H NMR (500 MHz, DMSO) δ 10.42 (s, 1H), 7.86 (d, *J* = 8.0 Hz, 1H), 7.64 – 7.56 (m, 2H), 7.51 (d, *J* = 2.2 Hz, 1H), 7.43 – 7.30 (m, 4H), 7.24 (tt, *J* = 7.4, 1.3 Hz, 1H), 7.17 – 7.05 (m, 3H), 6.92 (d, *J* = 8.0 Hz, 1H), 6.14 (s, 1H).

^{13}C NMR (150 MHz, DMSO) δ 167.0, 158.8 (t, J_{CF} = 26.4 Hz), 139.0, 136.5, 133.8, 133.4, 132.0, 131.9, 131.7, 131.1, 130.7, 129.5, 129.4, 128.7, 128.5, 124.3, 119.7, 111.5 (t, J_{CF} = 314.8 Hz), 65.9.

^{19}F NMR (471 MHz, DMSO) δ -52.5 – -53.9 (m, 2F).

HRMS-ESI: calcd. for $C_{22}H_{16}BrCl_2F_2N_2O_2$ $[M + H]^+$526.9735, found 526.9729.

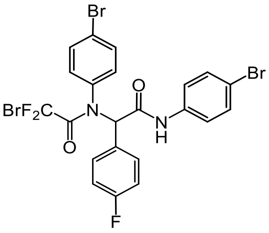

2-bromo-*N*-(4-bromophenyl)-*N*-(2-((4-bromophenyl)amino)-1-(4-fluorophenyl)-2-oxoethyl)-2,2-difluoroacetamide

4ac: 83 mg, light yellow solid, 65% yield.

^{1}H NMR (500 MHz, DMSO) δ 10.56 (s, 1H), 7.80 (dd, *J* = 8.7, 2.6 Hz, 1H), 7.63 – 7.54 (m, 3H), 7.54 – 7.49 (m, 2H), 7.29 (dd, *J* = 8.5, 2.4 Hz, 1H), 7.25 – 7.17 (m, 2H), 7.03 (t, *J* = 8.8 Hz, 2H), 6.80 (dd, *J* = 8.5, 2.6 Hz, 1H), 6.15 (s, 1H).

^{13}C NMR (125 MHz, DMSO) δ 167.8, 162.5 (d, J_{CF} = 247.0 Hz), 158.8 (t, J_{CF} = 25.8 Hz), 138.4, 135.9, 134.2, 133.8, 133.4 (d, J_{CF} = 8.8 Hz), 132.2, 131.6, 131.3, 128.7 (d, J_{CF} = 3.8 Hz), 122.6, 121.7, 115.7 (d, J_{CF} = 21.4 Hz), 111.4 (t, J_{CF} = 315.0 Hz), 66.2.

^{19}F NMR (471 MHz, DMSO) δ -52.3 – -54.4 (m, 2F), -112.4 (s, 1F).

HRMS-ESI: calcd. for $C_{22}H_{15}Br_3F_3N_2O_2$ $[M + H]^+$ 634.8610, found 634.8607.

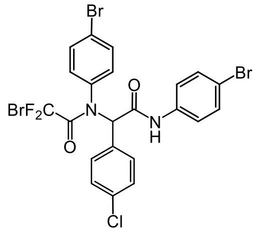

2-bromo-*N*-(4-bromophenyl)-*N*-(2-((4-bromophenyl)amino)-1-(4-chlorophenyl)-2-oxoethyl)-2,2-difluoroacetamide

4ad: 87 mg, white solid, 67% yield.

^{1}H NMR (500 MHz, DMSO) δ 10.57 (s, 1H), 7.80 (dd, *J* = 8.5, 2.5 Hz, 1H), 7.61 – 7.54 (m, 3H), 7.51 (d, *J* = 8.8 Hz, 2H), 7.32 (dd, *J* = 8.5, 2.4 Hz, 1H), 7.27 (d, *J* = 8.3 Hz, 2H), 7.19 (d, *J* = 8.3 Hz, 2H), 6.84 (dd, *J* = 8.3, 2.5 Hz, 1H), 6.14 (s, 1H).

^{13}C NMR (150 MHz, DMSO) δ 167.61, 158.78 (t, J_{CF} = 25.7 Hz), 138.35, 135.89, 134.23, 134.07, 133.78, 133.03, 132.17, 131.69, 131.42, 131.39, 128.84, 122.63, 121.67, 115.86, 111.34 (t, J_{CF} = 314.1 Hz), 66.21.

^{19}F NMR (471 MHz, DMSO) δ -52.24 – -54.87 (m, 2F).

HRMS-ESI: calcd. for $C_{22}H_{15}Br_3ClF_2N_2O_2$ [M + H]$^+$ 650.8315, found 650.8308

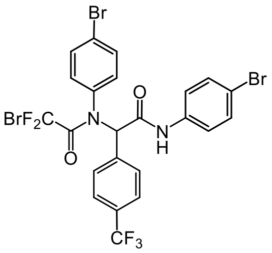

2-bromo-*N*-(4-bromophenyl)-*N*-(2-((4-bromophenyl)amino)-2-oxo-1-(4-(trifluoromethyl)phenyl)ethyl)-2,2-difluoroacetamide

4ae: 96 mg, white solid, 70% yield.

^{1}H NMR (500 MHz, DMSO) δ 10.62 (s, 1H), 7.81 (d, *J* = 8.7 Hz, 1H), 7.58 (dd, *J* = 8.5, 6.5 Hz, 5H), 7.51 (d, *J* = 8.7 Hz, 2H), 7.42 (d, *J* = 8.0 Hz, 2H), 7.33 – 7.22 (m, 1H), 6.85 (d, *J* = 8.5 Hz, 1H), 6.25 (s, 1H).

^{13}C NMR (125 MHz, DMSO) δ 167.3, 158.8 (t, J_{CF} = 26.0 Hz), 138.3, 137.1, 135.9, 134.2, 133.7, 132.2, 132.1, 131.7, 131.4, 129.6 (q, J_{CF} = 32.1 Hz), 125.6 (q, J_{CF} = 3.7 Hz), 124.3 (q, J_{CF} = 273.4 Hz), 122.7, 121.7, 116.0, 111.3 (t, J_{CF} = 315.0 Hz), 66.4.

^{19}F NMR (471 MHz, DMSO-d_6) δ -52.8 – -54.3 (m, 2F), -61.3 (s, 3F).

HRMS-ESI: calcd. for $C_{23}H_{15}Br_3F_5N_2O_2$ [M + H]$^+$ 684.8578, found 684.8575.

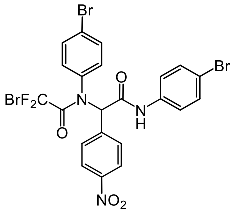

2-bromo-*N*-(4-bromophenyl)-*N*-(2-((4-bromophenyl)amino)-1-(4-nitrophenyl)-2-oxoethyl)-2,2-difluoroacetamide

4af: 60 mg, white solid, 45% yield.

^{1}H NMR (500 MHz, DMSO) δ 10.66 (s, 1H), 8.07 (d, *J* = 8.5 Hz, 2H), 7.82 (d, *J* = 8.6 Hz, 1H), 7.58 (d, *J* = 8.7 Hz, 3H), 7.50 (dd, *J* = 13.4, 8.6 Hz, 4H), 7.30 (d, *J* = 8.7 Hz, 1H), 6.88 (d, *J* = 8.5 Hz, 1H), 6.28 (s, 1H).

^{13}C NMR (150 MHz, DMSO) δ 167.0, 158.9 (t, J_{CF} = 25.7 Hz), 147.9, 139.8, 138.2, 135.8, 134.1, 133.7, 132.7, 132.2, 131.9, 131.6, 123.8, 122.9, 121.8, 116.1, 111.3 (t, J_{CF} = 314.1 Hz), 66.1.

^{19}F NMR (471 MHz, DMSO) δ -52.5 – -54.6 (m, 2F).

HRMS-ESI: calcd. for $C_{22}H_{15}Br_3F_2N_3O_4$ [M + H]$^+$ 661.8555, found 661.8561.

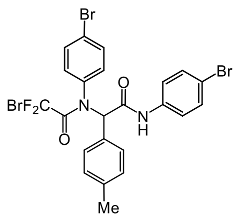

2-bromo-*N*-(4-bromophenyl)-*N*-(2-((4-bromophenyl)amino)-2-oxo-1-(*p*-tolyl)ethyl)-2,2-difluoroacetamide

4ag: 101 mg, light yellow solid, 80% yield.

^{1}H NMR (500 MHz, DMSO) δ 10.50 (s, 1H), 7.83 (dd, J = 8.7, 2.6 Hz, 1H), 7.62 – 7.57 (m, 2H), 7.55 (dd, J = 8.5, 2.4 Hz, 1H), 7.53 – 7.47 (m, 2H), 7.26 (dd, J = 8.5, 2.4 Hz, 1H), 7.04 (d, J = 8.1 Hz, 2H), 6.99 (d, J = 8.0 Hz, 2H), 6.79 (dd, J = 8.7, 2.6 Hz, 1H), 6.10 (s, 1H), 2.17 (s, 3H).

^{13}C NMR (150 MHz, DMSO) δ 168.1, 158.8 (t, J_{CF} = 25.7 Hz), 138.6, 138.5, 136.0, 134.2, 133.8, 132.1, 131.5, 131.2, 131.1, 129.4, 129.3, 122.5, 121.6, 115.7, 111.4 (t, J_{CF} = 314.8 Hz), 66.8, 21.1.

^{19}F NMR (471 MHz, DMSO) δ -45.1 – -59.2 (m, 2F).

HRMS-ESI: calcd. for $C_{23}H_{18}Br_3F_2N_2O_2$ [M + H]$^+$ 630.8861, found 630.8856.

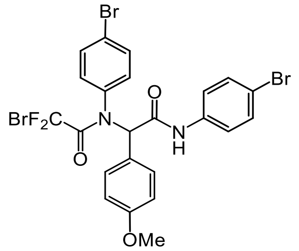

2-bromo-*N*-(4-bromophenyl)-*N*-(2-((4-bromophenyl)amino)-1-(4-methoxyphenyl)-2-oxoethyl)-2,2-difluoroacetamide

4ah: 110 mg, light yellow solid, 85% yield.

^{1}H NMR (500 MHz, DMSO) δ 10.49 (s, 1H), 7.81 (dd, J = 8.6, 2.6 Hz, 1H), 7.63 – 7.54 (m, 3H), 7.53 – 7.48 (m, 2H), 7.28 (dd, J = 8.5, 2.4 Hz, 1H), 7.12 – 7.01 (m, 2H), 6.77 (dd, J = 8.4, 2.6 Hz, 1H), 6.76 – 6.70 (m, 2H), 6.09 (s, 1H), 3.65 (s, 3H).

^{13}C NMR (125 MHz, DMSO) δ 168.2, 159.8, 158.7 (t, J_{CF} = 25.8 Hz), 138.5, 136.1, 134.2, 133.8, 132.5, 132.1, 131.5, 131.2, 124.1, 122.4, 121.6, 115.7, 114.2, 111.4 (t, J_{CF} = 315.0 Hz), 66.5, 55.6.

^{19}F NMR (471 MHz, DMSO) δ -46.8 – -57.8 (m, 2F).

HRMS-ESI: calcd. for $C_{23}H_{18}Br_3F_2N_2O_3$ [M + H]$^+$ 646.8810, found 646.8805.

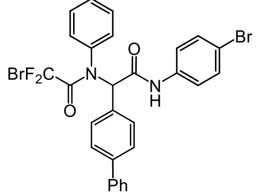

***N*-(1-([1,1'-biphenyl]-4-yl)-2-((4-bromophenyl)amino)-2-oxoethyl)-2-bromo-*N*-(4-bromophenyl)-2,2-difluoroacetamide**

4ai: 112 mg, light yellow solid, 81% yield.

^{1}H NMR (500 MHz, DMSO) δ 10.59 (s, 1H), 7.87 (dd, J = 8.5, 2.6 Hz, 1H), 7.66 – 7.60 (m, 2H), 7.60 – 7.55 (m, 3H), 7.55 – 7.48 (m, 4H), 7.42 (dd, J = 8.4, 6.8 Hz, 2H), 7.37 – 7.31 (m, 1H), 7.26 (dd, J = 7.7, 5.5 Hz, 3H), 6.85 (dd, J = 8.4, 2.6 Hz, 1H), 6.20 (s, 1H).

^{13}C NMR (151 MHz, DMSO) δ 167.9, 158.9 (t, J_{CF} = 25.7 Hz), 140.7, 139.4, 138.5, 136.0, 134.2, 133.9, 132.2, 131.8, 131.6, 131.5, 131.2, 129.4, 128.3, 127.0, 126.8, 122.5, 121.6, 115.8, 111.4 (t, J_{CF} = 314.8 Hz), 66.7.

^{19}F NMR (471 MHz, DMSO-*d*$_6$): δ -52.5 – -54.2 (m, 2F).

HRMS-ESI: calcd. for $C_{28}H_{20}Br_3F_2N_2O_2$ $[M + H]^+$ 692.9018, found 692.9010.

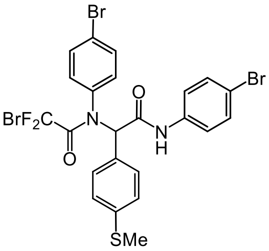

2-bromo-*N*-(4-bromophenyl)-*N*-(2-((4-bromophenyl)amino)-1-(4-(methylthio)phenyl)-2-oxoethyl)-2,2-difluoroacetamide

4aj: 110 mg, light yellow solid, 83% yield.

^{1}H NMR (500 MHz, DMSO-*d*$_6$) δ 10.51 (s, 1H), 7.90 – 7.76 (m, 1H), 7.63 – 7.55 (m, 3H), 7.54 – 7.47 (m, 2H), 7.30 (dd, *J* = 8.6, 2.4 Hz, 1H), 7.13 – 7.01 (m, 4H), 6.88 – 6.75 (m, 1H), 6.10 (s, 1H), 2.37 (s, 3H).

^{13}C NMR (125 MHz, DMSO) δ 167.9, 158.8 (t, J_{CF} = 26.5 Hz), 139.9, 138.5, 136.0, 134.2, 133.8, 132.1, 131.6, 131.3, 128.5, 125.7, 122.5, 121.6, 115.7, 111.4 (t, J_{CF} = 315.0 Hz), 66.6, 14.7.

^{19}F NMR (471 MHz, DMSO) δ -52.20 – -54.55 (m, 2F).

HRMS-ESI: calcd. for $C_{23}H_{18}Br_3F_2N_2O_2S$ $[M + H]^+$ 662.8582, found 662.8573.

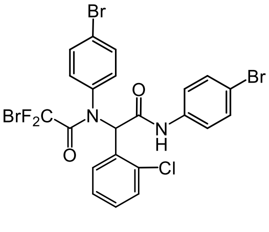

2-bromo-*N*-(4-bromophenyl)-*N*-(2-((4-bromophenyl)amino)-1-(2-chlorophenyl)-2-oxoethyl)-2,2-difluoroacetamide

4ak: 102 mg,light yellow solid, 78% yield.

^{1}H NMR (500 MHz, DMSO) δ 10.71 (s, 1H), 7.91 (dd, *J* = 8.6, 2.6 Hz, 1H), 7.65 – 7.55 (m, 3H), 7.55 – 7.48 (m, 3H), 7.36 – 7.24 (m, 2H), 7.09 (td, *J* = 7.6, 1.3 Hz, 1H), 6.87 (ddd, *J* = 8.3, 5.6, 2.1 Hz, 2H), 6.51 (s, 1H).

^{13}C NMR (150 MHz, DMSO) δ 167.13, 159.17 (t, J_{CF} = 26.4 Hz), 138.24, 135.66, 135.45, 133.57, 132.23, 131.99, 131.96, 131.63, 131.53, 131.50, 130.78, 130.13, 127.96, 122.92, 121.79, 116.05, 110.98 (t, J_{CF} = 314.1 Hz), 64.08.

^{19}F NMR (471 MHz, DMSO) δ -52.87 – -54.36 (m, 2F).

HRMS-ESI: calcd. for $C_{22}H_{15}Br_3ClF_2N_2O_2$ $[M + H]^+$ 650.8315, found 650.8312

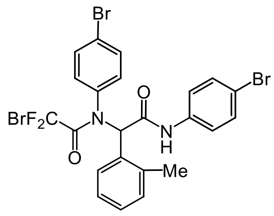

2-bromo-*N*-(4-bromophenyl)-*N*-(2-((4-bromophenyl)amino)-2-oxo-1-(*o*-tolyl)ethyl)-2,2-difluoroacetamide

4al: 95 mg, white solid, 75% yield.

^{1}H NMR (500 MHz, DMSO) δ 10.49 (s, 1H), 7.94 (dd, J = 8.6, 2.6 Hz, 1H), 7.64 – 7.47 (m, 5H), 7.27 – 7.17 (m, 2H), 7.12 (td, J = 7.5, 1.4 Hz, 1H), 6.88 (td, J = 7.6, 1.4 Hz, 1H), 6.72 – 6.67 (m, 1H), 6.64 (dd, J = 8.6, 2.6 Hz, 1H), 6.35 (s, 1H), 2.49 (s, 3H).

^{13}C NMR (125 MHz, DMSO) δ 168.3, 159.4 (t, J_{CF} = 26.5 Hz), 138.6, 138.4, 135.7, 133.8, 132.4, 132.2, 131.6, 131.3, 131.1, 131.0, 129.9, 129.6, 126.5, 122.6, 121.7, 115.8, 111.1 (t, J_{CF} = 315.0 Hz), 63.6, 19.5.

^{19}F NMR (471 MHz, DMSO): δ -53.4 (d, J = 5.4 Hz, 2F).

HRMS-ESI: calcd. for $C_{23}H_{18}Br_3F_2N_2O_2$ $[M + H]^+$630.8861, found 630.8854.

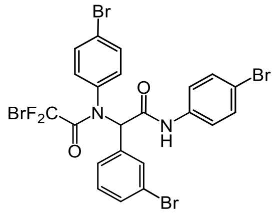

2-bromo-*N*-(4-bromophenyl)-*N*-(1-(3-bromophenyl)-2-((4-bromophenyl)amino)-2-oxoethyl)-2,2-difluoroacetamide

4am: 109 mg, white solid, 78% yield.

^{1}H NMR (500 MHz, DMSO) δ 10.59 (s, 1H), 7.80 (dd, J = 8.5, 2.5 Hz, 1H), 7.59 (dd, J = 9.3, 2.6 Hz, 3H), 7.55 – 7.49 (m, 2H), 7.45 (t, J = 1.8 Hz, 1H), 7.41 (dt, J = 7.8, 1.6 Hz, 1H), 7.31 (dd, J = 8.6, 2.5 Hz, 1H), 7.17 – 7.05 (m, 2H), 6.84 (dd, J = 8.4, 2.6 Hz, 1H), 6.12 (s, 1H).

^{13}C NMR (150 MHz, DMSO) δ 167.4, 158.7 (t, J_{CF} = 26.4 Hz), 138.3, 135.9, 134.9, 134.2, 134.1, 133.8, 132.2, 132.1, 131.6, 131.4, 130.8, 129.9, 122.7, 121.8, 121.7, 115.9, 111.3 (t, J_{CF} = 314.8 Hz), 66.3.

^{19}F NMR (471 MHz, DMSO) δ -49.3 – -59.0 (m, 2F).

HRMS-ESI: calcd. for $C_{22}H_{15}Br_4F_2N_2O_2$ $[M + H]^+$ 696.7789, found 696.7780.

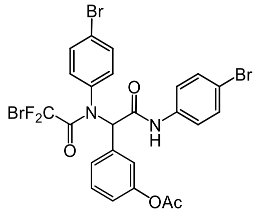

3-(1-(2-bromo-*N*-(4-bromophenyl)-2,2-difluoroacetamido)-2-((4-bromophenyl)amino)-2-oxoethyl)phenyl acetate

4an: 88 mg, light yellow solid, 65% yield.

^{1}H NMR (500 MHz, DMSO) δ 10.64 (s, 1H), 7.78 (dd, *J* = 8.7, 2.6 Hz, 1H), 7.64 – 7.57 (m, 2H), 7.56 – 7.47 (m, 3H), 7.29 – 7.17 (m, 2H), 7.06 – 6.95 (m, 3H), 6.84 (dd, *J* = 8.6, 2.6 Hz, 1H), 6.16 (s, 1H), 2.22 (s, 3H).

^{13}C NMR (150 MHz, DMSO) δ 169.3, 167.5, 158.8 (t, J_{CF} = 26.4 Hz), 150.7, 138.4, 135.8, 134.2, 133.9, 133.7, 132.2, 131.6, 131.2, 129.8, 128.3, 124.7, 122.9, 122.6, 121.7, 115.9, 111.3 (t, J_{CF} = 314.8 Hz), 66.5, 21.3.

^{19}F NMR (471 MHz, DMSO) δ -52.7 – -54.2 (m, 2F).

HRMS-ESI: calcd. for $C_{24}H_{18}Br_3F_2N_2O_4$ $[M + H]^+$674.8759, found 674.8758.

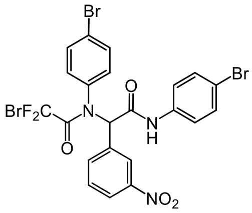

2-bromo-*N*-(4-bromophenyl)-*N*-(2-((4-bromophenyl)amino)-1-(3-nitrophenyl)-2-oxoethyl)-2,2-difluoroacetamide

4ao: 93 mg, light yellow solid, 70% yield.

^{1}H NMR (500 MHz, DMSO) δ 10.66 (s, 1H), 8.17 (t, *J* = 2.1 Hz, 1H), 8.09 (ddd, *J* = 8.1, 2.4, 1.2 Hz, 1H), 7.87 – 7.76 (m, 1H), 7.63 – 7.55 (m, 3H), 7.54 – 7.50 (m, 3H), 7.47 (t, *J* = 7.9 Hz, 1H), 7.33 – 7.21 (m, 1H), 6.92 – 6.79 (m, 1H), 6.30 (s, 1H).

^{13}C NMR (125 MHz, DMSO) δ 167.1, 158.7 (t, J_{CF} = 26.5 Hz), 147.8, 138.2, 137.4, 135.8, 134.6, 134.2, 133.8, 132.2, 131.8, 131.6, 130.4, 126.4, 124.2, 122.8, 121.7, 116.0, 111.3 (t, J_{CF} = 315.0 Hz), 66.0.

^{19}F NMR (471 MHz, DMSO) δ -52.4 – -54.6 (m, 2F).

HRMS-ESI: calcd. for $C_{22}H_{15}Br_3F_2N_3O_4$ $[M + H]^+$ 661.8555, found 661.8551.

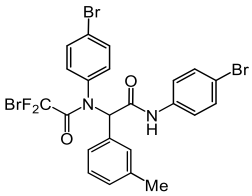

2-bromo-*N*-(4-bromophenyl)-*N*-(2-((4-bromophenyl)amino)-2-oxo-1-(m-tolyl)ethyl)-2,2-difluoroacetamide

4ap: 96 mg, white solid, 76% yield.

^{1}H NMR (500 MHz, DMSO) δ 10.53 (s, 1H), 7.83 (dd, *J* = 8.5, 2.6 Hz, 1H), 7.64 – 7.54 (m, 3H), 7.53 – 7.47 (m, 2H), 7.23 (dd, *J* = 8.5, 2.4 Hz, 1H), 7.10 – 6.95 (m, 3H), 6.89 (d, *J* = 7.4 Hz, 1H), 6.75 (dd, *J* = 8.6, 2.6 Hz, 1H), 6.10 (s, 1H), 2.15 (s, 3H).

^{13}C NMR (150 MHz, DMSO) δ 167.9, 158.8 (t, J_{CF} = 25.7 Hz), 138.5, 137.9, 136.0, 134.1, 133.9, 132.2, 132.1, 131.9, 131.4, 131.1, 129.8, 128.7, 128.1, 122.4, 121.6, 115.7, 111.4 (t, J_{CF} = 314.8 Hz), 67.0, 21.2.

^{19}F NMR (471 MHz, DMSO) δ -52.4 – -54.2 (m, 2F).

HRMS-ESI: calcd. for $C_{23}H_{18}Br_3F_2N_2O_2$ $[M + H]^+$630.8861, found 630.8855.

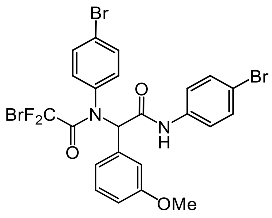

2-bromo-*N*-(4-bromophenyl)-*N*-(2-((4-bromophenyl)amino)-1-(3-methoxyphenyl)-2-oxoethyl)-2,2-difluoroacetamide

4aq: 91 mg, white solid, 70% yield.

^{1}H NMR (500 MHz, DMSO) δ 10.55 (s, 1H), 7.83 (dd, *J* = 8.6, 2.5 Hz, 1H), 7.64 – 7.53 (m, 3H), 7.53 – 7.47 (m, 2H), 7.27 (dd, *J* = 8.6, 2.4 Hz, 1H), 7.07 (t, *J* = 7.9 Hz, 1H), 6.88 – 6.82 (m, 1H), 6.81 – 6.73 (m, 2H), 6.66 (dt, *J* = 7.7, 1.2 Hz, 1H), 6.10 (s, 1H), 3.64 (s, 3H).

^{13}C NMR (125 MHz, DMSO) δ 167.9, 159.3, 158.8 (t, J_{CF} = 26.5 Hz), 138.5, 136.0, 134.2, 133.9, 133.6, 132.2, 131.4, 131.2, 129.8, 123.2, 122.5, 121.6, 117.0, 115.7, 115.0, 111.4 (t, J_{CF} = 315.0 Hz), 67.0, 55.6.

^{19}F NMR (471 MHz, DMSO) δ -51.8 – -54.6 (m, 2F).

HRMS-ESI: calcd. for $C_{23}H_{18}Br_3F_2N_2O_3$ $[M + H]^+$ 646.8810, found 646.8807.

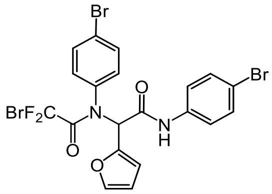

2-bromo-*N*-(4-bromophenyl)-*N*-(2-((4-bromophenyl)amino)-1-(furan-2-yl)-2-oxoethyl)-2,2-difluoroacetamide

4ar: 49 mg, white solid, 40% yield.

^{1}H NMR (500 MHz, DMSO) δ 10.61 (s, 1H), 7.79 (dd, *J* = 8.6, 2.6 Hz, 1H), 7.59 (dq, *J* = 9.8, 2.6 Hz, 3H), 7.52 (dt, *J* = 6.8, 2.5 Hz, 3H), 7.40 (dd, *J* = 8.5, 2.4 Hz, 1H), 6.96 (dd, *J* = 8.7, 2.6 Hz, 1H), 6.33 – 6.21 (m, 3H).

^{13}C NMR (125 MHz, DMSO) δ 165.2, 158.7 (t, J_{CF} = 26.5, 2.6 Hz), 145.2, 145.0, 138.3, 136.1, 133.5, 133.0, 132.2, 131.7, 131.5, 122.8, 121.7, 115.9, 114.1, 111.5, 111.1 (t, J_{CF} = 315.0 Hz), 61.3.

^{19}F NMR (471 MHz, DMSO) δ -53.0 – -54.5 (m, 2F).

HRMS-ESI: calcd. for $C_{20}H_{14}Br_3F_2N_2O_3$ $[M + H]^+$ 606.8497, found 606.8491.

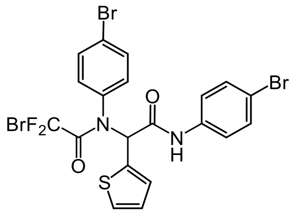

2-bromo-*N*-(4-bromophenyl)-*N*-(2-((4-bromophenyl)amino)-2-oxo-1-(thiophen-2-yl)ethyl)-2,2-difluoroacetamide

4as: 52 mg, white solid, 42% yield.

^{1}H NMR (500 MHz, DMSO) δ 10.64 (s, 1H), 7.80 (dd, *J* = 8.4, 2.5 Hz, 1H), 7.63 – 7.56 (m, 3H), 7.55 – 7.49 (m, 2H), 7.46 (dd, *J* = 5.1, 1.3 Hz, 1H), 7.40 – 7.29 (m, 1H), 7.01 (d, *J* = 3.5 Hz, 1H), 6.94 (dd, *J*

= 8.5, 2.5 Hz, 1H), 6.87 (dd, J = 5.1, 3.5 Hz, 1H), 6.37 (s, 1H).

^{13}C NMR (125 MHz, DMSO) δ 167.4, 158.6 (t, J_{CF} = 26.5 Hz), 138.3, 135.8, 133.9, 133.6, 132.2, 131.9, 131.6, 131.3, 129.9, 127.3, 122.8, 121.7, 115.9, 111.3 (t, J_{CF} = 314.4 Hz), 62.1.

^{19}F NMR (471 MHz, DMSO) δ -48.9 – -56.0 (m, 2F).

HRMS-ESI: calcd. for $C_{20}H_{14}Br_3F_2N_2O_2S$ [M + H]$^+$622.8269, found 622.8258.

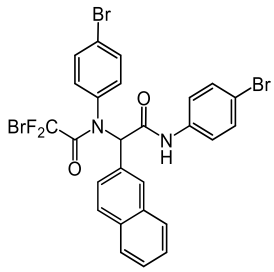

2-bromo-*N*-(4-bromophenyl)-*N*-(2-((4-bromophenyl)amino)-1-(naphthalen-2-yl)-2-oxoethyl)-2,2-difluoroacetamide

4at: 100 mg, white solid, 75% yield.

^{1}H NMR (500 MHz, DMSO) δ 10.59 (s, 1H), 7.92 (d, J = 8.7 Hz, 1H), 7.86 – 7.75 (m, 3H), 7.70 (d, J = 8.6 Hz, 1H), 7.61 (d, J = 8.5 Hz, 2H), 7.57 – 7.42 (m, 5H), 7.23 (d, J = 8.5 Hz, 1H), 7.17 – 7.05 (m, 1H), 6.81 (d, J = 10.0 Hz, 1H), 6.34 (s, 1H).

^{13}C NMR (125 MHz, DMSO) δ 167.9, 158.9 (t, J_{CF} = 26.5 Hz), 138.5, 136.0, 134.1, 133.9, 132.9, 132.7, 132.2, 132.1, 131.5, 131.2, 129.9, 128.4, 128.3, 128.0, 127.9, 127.4, 127.0, 122.5, 121.7, 115.8, 111.4 (t, J_{CF} = 315.0 Hz), 67.2.

^{19}F NMR (471 MHz, DMSO-d_6) δ -52.8 – -53.8 (m, 2F).

HRMS-ESI: calcd. for $C_{26}H_{18}Br_3F_2N_2O_2$ [M + H]$^+$ 666.8861, found 666.8853.

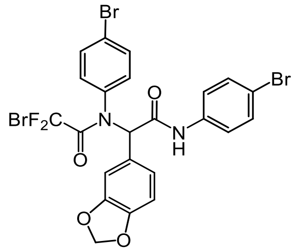

***N*-(1-(benzo[*d*][1,3]dioxol-5-yl)-2-((4-bromophenyl)amino)-2-oxoethyl)-2-bromo-*N*-(4-bromophenyl)-2,2-difluoroacetamide**

4au: 66 mg, white solid, 50% yield.

^{1}H NMR (500 MHz, DMSO) δ 10.51 (s, 1H), 7.80 (dd, J = 8.5, 2.5 Hz, 1H), 7.63 – 7.55 (m, 3H), 7.54 – 7.48 (m, 2H), 7.34 (dd, J = 8.5, 2.4 Hz, 1H), 6.85 (dd, J = 8.7, 2.6 Hz, 1H), 6.76 (d, J = 1.8 Hz, 1H), 6.71 (d, J = 8.1 Hz, 1H), 6.59 (dd, J = 8.1, 1.8 Hz, 1H), 6.04 (s, 1H), 5.94 (dd, J = 3.1, 1.0 Hz, 2H).

^{13}C NMR (125 MHz, DMSO) δ 168.03, 158.70 (t, J_{CF} = 25.8 Hz), 147.89, 147.49, 138.50, 135.99, 134.16, 133.86, 132.14, 131.52, 131.29, 125.69, 125.12, 122.51, 121.60, 115.70, 111.43 (t, J_{CF} = 315.6 Hz), 111.36, 108.50, 101.71, 66.72.

^{19}F NMR (471 MHz, DMSO) δ -48.21 – -59.20 (m, 2F).

HRMS-ESI: calcd. for $C_{23}H_{16}Br_3F_2N_2O_4$ [M + H]$^+$ 660.8603, found 660.8600.

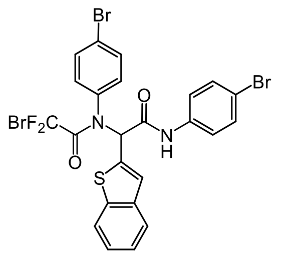

N-(1-(benzo[b]thiophen-2-yl)-2-((4-bromophenyl)amino)-2-oxoethyl)-2-bromo-N-(4-bromophenyl)-2,2-difluoroacetamide

4av: 88 mg, light yellow solid, 65% yield.

^{1}H NMR (500 MHz, DMSO): δ 10.65 (s, 1H), 7.99 – 7.87 (m, 3H), 7.65 – 7.59 (m, 2H), 7.57 – 7.50 (m, 4H), 7.46 – 7.41 (m, 1H), 7.39 (s, 1H), 7.03 (dd, J = 8.6, 2.4 Hz, 1H), 6.58 (s, 1H), 6.44 (dd, J = 8.7, 2.6 Hz, 1H).

^{13}C NMR (125 MHz, DMSO) δ 167.4, 159.3 (t, J_{CF} = 26.5 Hz), 139.5, 138.4, 138.3, 135.8, 133.7, 132.4, 132.2, 131.6, 131.0, 130.8, 127.4, 125.4, 125.3, 123.6, 122.6, 122.1, 121.8, 115.9, 111.2 (t, J_{CF} = 315.0 Hz), 60.5.

^{19}F NMR (471 MHz, DMSO) δ -53.4 (s, 2F).

HRMS-ESI: calcd. for $C_{24}H_{16}Br_3F_2N_2O_2S$ $[M + H]^+$ 672.8425, found 672.8423.

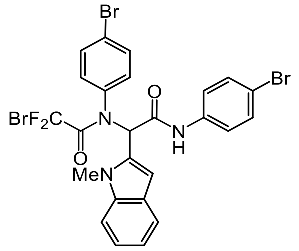

2-bromo-N-(4-bromophenyl)-N-(2-((4-bromophenyl)amino)-1-(1-methyl-1H-indol-2-yl)-2-oxoethyl)-2,2-difluoroacetamide

4aw: 80 mg, white solid, 60% yield.

^{1}H NMR (500 MHz, DMSO) δ 10.58 (s, 1H), 7.93 (dd, J = 8.7, 2.6 Hz, 1H), 7.64 – 7.58 (m, 2H), 7.55 (td, J = 7.2, 2.3 Hz, 3H), 7.44 (d, J = 8.4 Hz, 1H), 7.39 – 7.31 (m, 1H), 7.19 (dd, J = 8.5, 2.5 Hz, 1H), 7.14 (ddd, J = 8.3, 7.0, 1.2 Hz, 1H), 6.99 – 6.87 (m, 2H), 6.48 (s, 1H), 6.07 (s, 1H), 3.86 (s, 3H).

^{13}C NMR (125 MHz, DMSO) δ 166.7, 159.2 (t, J_{CF} = 25.8 Hz), 138.4, 137.6, 135.8, 133.8, 132.3, 132.1, 131.6, 131.3, 130.7, 126.7, 122.7, 121.6, 121.0, 120.0, 115.9, 111.2 (t, J_{CF} = 315.0 Hz), 110.4, 105.0, 59.7, 30.4.

^{19}F NMR (471 MHz, DMSO) δ -53.4 (d, J = 27.7 Hz, 2F).

HRMS-ESI: calcd. for $C_{25}H_{19}Br_3F_2N_3O_2$ $[M + H]^+$ 669.8970, found 669.8962.

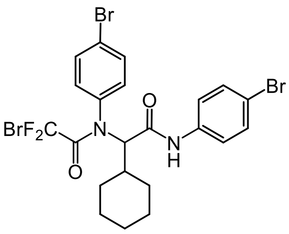

2-bromo-*N*-(4-bromophenyl)-*N*-(2-((4-bromophenyl)amino)-1-cyclohexyl-2-oxoethyl)-2,2-difluoroacetamide

4ax: 91 mg, light yellow solid, 73% yield.

^{1}H NMR (500 MHz, DMSO-*d*$_6$): δ 10.56 (s, 1H), 7.68 (dd, *J* = 8.6, 2.4 Hz, 1H), 7.61 (dd, *J* = 8.6, 2.4 Hz, 1H), 7.56 – 7.51 (m, 2H), 7.51 – 7.47 (m, 2H), 7.44 (dd, *J* = 8.8, 2.5 Hz, 1H), 7.34 (dd, *J* = 8.7, 2.7 Hz, 1H), 4.98 (d, *J* = 10.3 Hz, 1H), 1.95 (d, *J* = 11.1 Hz, 1H), 1.81 – 1.66 (m, 2H), 1.65 – 1.53 (m, 2H), 1.49 – 1.36 (m, 1H), 1.24 – 1.10 (m, 3H), 1.10 – 1.01 (m, 2H).

^{13}C NMR (125 MHz, DMSO) δ 166.8, 159.3 (t, J_{CF} = 25.8 Hz), 138.1, 135.4, 133.9, 132.5, 132.4, 132.0, 132.0, 123.2, 122.1, 116.0, 111.3 (t, J_{CF} = 316.3 Hz), 67.0, 37.1, 30.0, 29.7, 26.2, 25.8.

^{19}F NMR (471 MHz, DMSO) δ -52.3 (s, 2F).

HRMS-ESI: calcd. for $C_{22}H_{22}Br_3F_2N_2O_2$ [M + H] $^+$622.9174, found 622.9170.

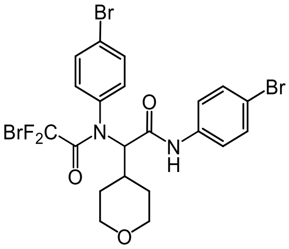

2-bromo-*N*-(4-bromophenyl)-*N*-(2-((4-bromophenyl)amino)-2-oxo-1-(tetrahydro-2*H*-pyran-4-yl)ethyl)-2,2-difluoroacetamide

4ay: 111 mg, light yellow solid, 89% yield.

^{1}H NMR (500 MHz, DMSO) δ 10.60 (s, 1H), 7.72 – 7.64 (m, 1H), 7.64 – 7.58 (m, 1H), 7.50 (s, 4H), 7.45 – 7.36 (m, 2H), 5.04 (d, *J* = 10.3 Hz, 1H), 3.89 (ddd, *J* = 11.6, 4.3, 2.1 Hz, 1H), 3.78 (dt, *J* = 11.6, 3.3 Hz, 1H), 3.29 – 3.22 (m, 1H), 3.18 (td, *J* = 11.2, 3.8 Hz, 1H), 2.01 (ddt, *J* = 14.4, 10.2, 5.3 Hz, 1H), 1.88 – 1.77 (m, 1H), 1.54 – 1.40 (m, 1H), 1.34 (dtd, *J* = 15.1, 11.6, 9.9, 5.0 Hz, 2H).

^{13}C NMR (125 MHz, DMSO) δ 166.1, 159.3 (t, J_{CF} = 25.8 Hz), 138.0, 135.3, 133.7, 132.6, 132.1, 132.0, 123.3, 122.2, 116.1, 111.3 (t, J_{CF} = 315.6 Hz), 67.0, 66.7, 66.4, 34.8, 30.0, 29.9.

^{19}F NMR (471 MHz, DMSO) δ -52.5 (s, 2F).

HRMS-ESI: calcd. for $C_{21}H_{20}Br_3F_2N_2O_3$ [M + H]$^+$ 624.8967, found 624.8965.

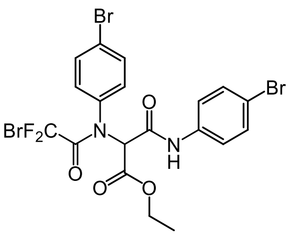

ethyl 2-(2-bromo-*N*-(4-bromophenyl)-2,2-difluoroacetamido)-3-((4-bromophenyl)amino)-3-oxopropanoate

4az: 61 mg, white solid, 50% yield.

^{1}H NMR (500 MHz, DMSO) δ 10.67 (s, 1H), 7.65 (d, *J* = 32.6 Hz, 2H), 7.58 – 7.41 (m, 4H), 7.40 – 7.31 (m, 2H), 5.61 (s, 1H), 4.20 (q, *J* = 7.0 Hz, 2H), 1.21 (t, *J* = 7.1 Hz, 3H).

^{13}C NMR (125 MHz, DMSO) δ 165.9, 161.5, 158.9 (t, J_{CF} = 26.5 Hz), 137.5, 136.6, 132.5, 132.4, 132.3, 123.6, 121.8, 116.5, 110.9 (t, J_{CF} = 313.1 Hz), 67.5, 62.6, 14.3.

^{19}F NMR (471 MHz, DMSO) δ -53.8 (s, 2F).

HRMS-ESI: calcd. for $C_{19}H_{16}Br_3F_2N_2O_4$ $[M + H]^+$ 612.8603, found 612.8597.

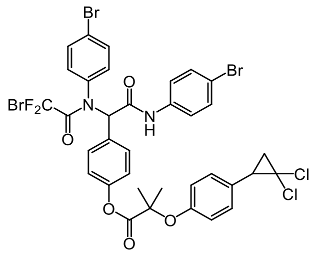

4-(1-(2-bromo-*N*-(4-bromophenyl)-2,2-difluoroacetamido)-2-((4-bromophenyl)amino)-2-oxoethyl)phenyl 2-(4-(2,2-dichlorocyclopropyl)phenoxy)-2-methylpropanoate

4A: 105 mg, white solid, 58% yield.

^{1}H NMR (500 MHz, DMSO-*d*$_6$): δ 10.58 (s, 1H), 7.82 (dd, *J* = 8.6, 2.6 Hz, 1H), 7.66 – 7.58 (m, 2H), 7.56 (dd, *J* = 8.7, 2.5 Hz, 1H), 7.53 – 7.48 (m, 2H), 7.30 – 7.19 (m, 5H), 6.88 (ddd, *J* = 14.5, 7.6, 1.8 Hz, 4H), 6.81 (dd, *J* = 8.8, 2.6 Hz, 1H), 6.17 (s, 1H), 3.03 (dd, *J* = 10.5, 8.9 Hz, 1H), 2.13 – 1.99 (m, 2H), 1.65 (s, 6H).

^{13}C NMR (125 MHz, DMSO): δ 172.2, 167.8, 158.8 (t, J_{CF} = 25.8 Hz), 154.8, 150.7, 138.4, 135.9, 134.2, 133.8, 132.6, 132.2, 132.0, 131.6, 131.3, 130.5, 130.4, 128.7, 128.6, 122.6, 122.0, 121.7, 120.7, 118.7, 118.7, 115.8, 111.4 (t, J_{CF} = 315.0 Hz), 79.3, 66.3, 62.4, 34.4, 25.6, 25.4, 25.3.

^{19}F NMR (471 MHz, DMSO) δ -52.3 – -54.3 (m, 2F).

HRMS-ESI: calcd. for $C_{35}H_{28}Br_3Cl_2F_2N_2O_5$ $[M + H]^+$ 902.8868, found 902.8865.

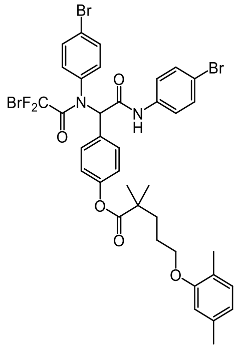

4-(1-(2-bromo-*N*-(4-bromophenyl)-2,2-difluoroacetamido)-2-((4-bromophenyl)amino)-2-oxoethyl)phenyl 5-(2,5-dimethylphenoxy)-2,2-dimethylpentanoate

4B: 111 mg, white solid, 64% yield.

^{1}H NMR (500 MHz, DMSO) δ 10.57 (s, 1H), 7.82 (dd, *J* = 8.6, 2.5 Hz, 1H), 7.64 – 7.49 (m, 5H), 7.29 (dd, *J* = 8.5, 2.4 Hz, 1H), 7.22 – 7.16 (m, 2H), 6.97 (d, *J* = 7.5 Hz, 1H), 6.90 – 6.78 (m, 3H), 6.71 (d, *J* = 1.6 Hz, 1H), 6.61 (dd, *J* = 7.6, 1.6 Hz, 1H), 6.16 (s, 1H), 4.04 – 3.89 (m, 2H), 2.22 (s, 3H), 2.04 (s, 3H), 1.82 – 1.74 (m, 2H), 1.70 (td, *J* = 7.6, 3.8 Hz, 2H), 1.25 (s, 3H), 1.24 (s, 3H);

^{13}C NMR (125 MHz, DMSO) δ 175.9, 167.9, 158.8 (t, J_{CF} = 27.7 Hz), 156.8, 151.2, 138.4, 136.5, 135.9, 134.2, 133.8, 132.4, 132.2, 131.6, 131.3, 130.6, 129.9, 123.0, 122.6, 122.2, 121.7, 121.0, 115.8, 112.5, 111.4 (t, J_{CF} = 314.4 Hz), 67.6, 66.3, 42.3, 36.9, 25.2, 25.1, 21.5, 16.0.
^{19}F NMR (471 MHz, DMSO) δ -52.6 – -54.0 (m, 2F).
HRMS-ESI: calcd. for $C_{37}H_{36}Br_3F_2N_2O_5$ $[M + H]^+$ 865.0117, found 865.0114.

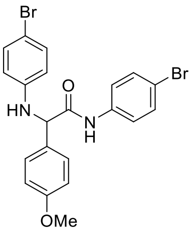

***N*-(4-bromophenyl)-2-(*N*-(4-bromophenyl)formamido)-2-(4-methoxyphenyl)acetamide**
5: 90 mg, white solid, 92% yield.
^{1}H NMR (500 MHz, DMSO) δ 10.44 (s, 1H), 7.64 – 7.55 (m, 2H), 7.54 – 7.43 (m, 4H), 7.28 – 7.17 (m, 2H), 6.99 – 6.88 (m, 2H), 6.73 – 6.61 (m, 2H), 6.54 (d, J = 7.5 Hz, 1H), 5.11 (d, J = 7.5 Hz, 1H), 3.73 (s, 3H).
^{13}C NMR (125 MHz, DMSO) δ 170.5, 159.5, 147.0, 138.5, 132.1, 131.8, 130.7, 129.0, 121.8, 115.7, 115.5, 114.4, 108.0, 61.1, 55.6.
HRMS-ESI: calcd. for $C_{21}H_{19}Br_2N_2O_2$ $[M + H]^+$ 490.9788, found 490.9784.

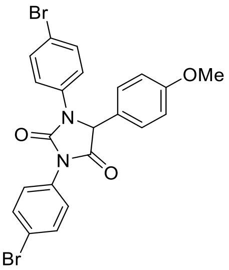

1,3-bis(4-bromophenyl)-5-(4-methoxyphenyl)imidazolidine-2,4-dione
6: 93 mg, white solid, 90% yield.
^{1}H NMR (500 MHz, DMSO) δ 7.86 – 7.68 (m, 2H), 7.54 (d, J = 1.7 Hz, 4H), 7.51 – 7.42 (m, 4H), 7.03 – 6.83 (m, 2H), 6.07 (s, 1H), 3.72 (s, 3H).
^{13}C NMR (125 MHz, DMSO) δ 169.9, 160.1, 153.4, 136.1, 132.4, 132.2, 131.5, 129.7, 129.6, 125.5, 123.8, 121.8, 117.4, 114.9, 63.4, 55.6.
HRMS-ESI: calcd. for $C_{22}H_{17}Br_2N_2O_3$ $[M + H]^+$ 516.9580, found 516.9578.

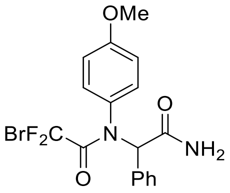

***N*-(2-amino-2-oxo-1-phenylethyl)-2-bromo-2,2-difluoro-*N*-(4-methoxyphenyl)acetamide**
7: 46 mg, white solid, 55% yield.
^{1}H NMR (500 MHz, $CDCl_3$) δ 7.65 (d, J = 8.9 Hz, 1H), 7.29 – 7.22 (m, 1H), 7.22 – 7.16 (m, 2H), 7.11

(d, J = 7.1 Hz, 2H), 6.88 – 6.68 (m, 1H), 6.50 (s, 2H), 6.05 (s, 1H), 6.00 (s, 1H), 5.82 (s, 1H), 3.73 (s, 3H).

^{13}C NMR (125 MHz, $CDCl_3$) δ 170.5, 159.9 (t, J_{CF} = 25.8 Hz), 159.6, 132.6, 132.2, 132.1, 130.8, 129.3, 129.1, 128.6, 113.3, 113.2, 111.3 (t, J_{CF} = 317.5 Hz), 67.4, 55.3.

^{19}F NMR (471 MHz, $CDCl_3$) δ -51.5 – -53.2 (m, 2F).

HRMS-ESI: calcd. for $C_{17}H_{16}BrF_2N_2O_3$ $[M + H]^+$ 413.0307, found 413.0305.

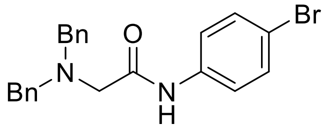

***N*-(4-bromophenyl)-2-(dibenzylamino)acetamide**

9: 48 mg, white solid, 58% yield

^{1}H NMR (500 MHz, DMSO) δ 9.75 (s, 1H), 7.57 (d, J = 8.5 Hz, 2H), 7.47 (d, J = 8.5 Hz, 2H), 7.42 (d, J = 7.5 Hz, 4H), 7.34 (t, J = 7.5 Hz, 4H), 7.25 (t, J = 7.3 Hz, 2H), 3.76 (s, 4H), 3.24 (s, 2H).

^{13}C NMR (125 MHz, DMSO) δ 169.7, 139.0, 138.4, 131.9, 129.3, 128.8, 127.6, 121.7, 115.4, 58.1, 56.6.

HRMS-ESI: calcd. for $C_{22}H_{22}BrN_2O$ $[M + H]^+$ 409.0910, found 409.0909.

8. NMR spectra of products

^{1}H NMR (500 MHz, DMSO), ^{13}C NMR (150 MHz, DMSO) and ^{19}F NMR (471 MHz, DMSO) spectra for 4a

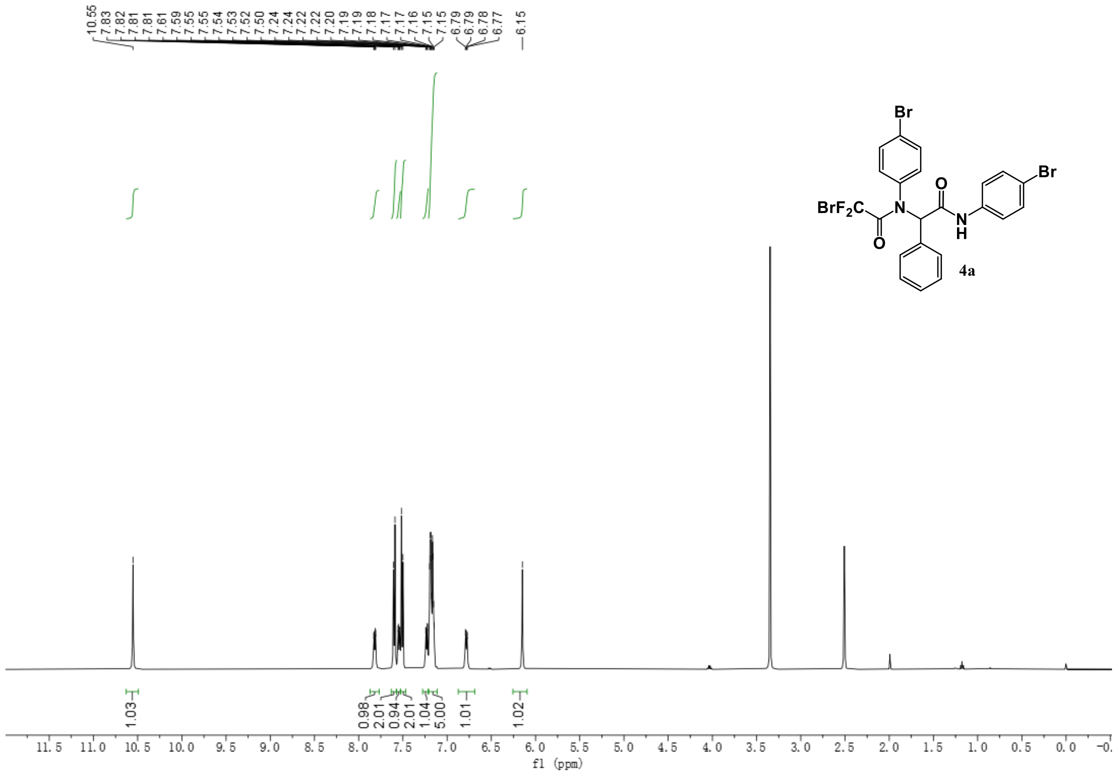

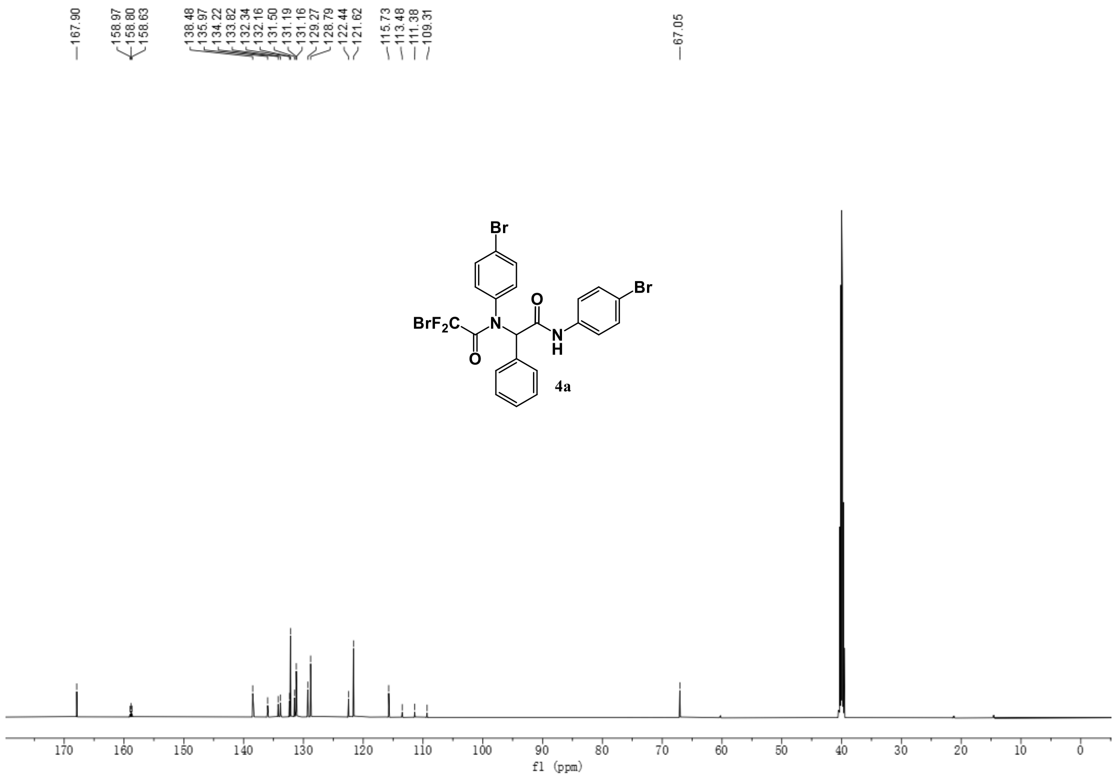

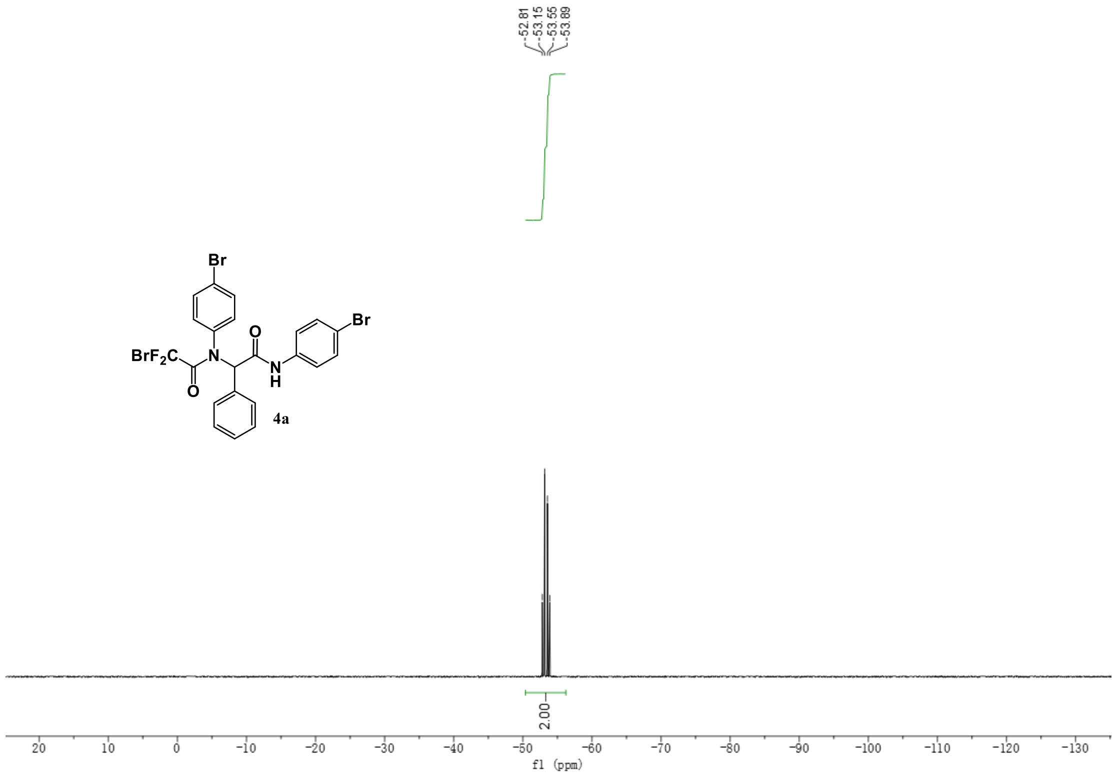

^{1}H NMR (500 MHz, DMSO), ^{13}C NMR (125 MHz, DMSO) and ^{19}F NMR (471 MHz, DMSO) spectra for 4b

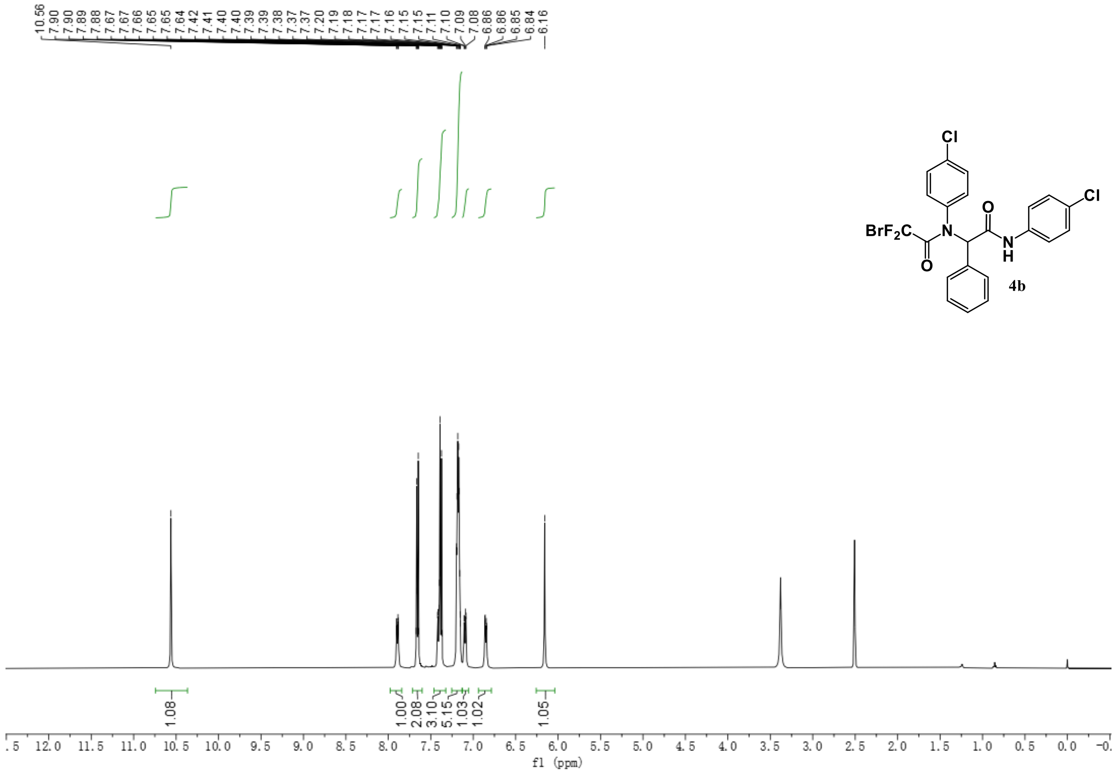

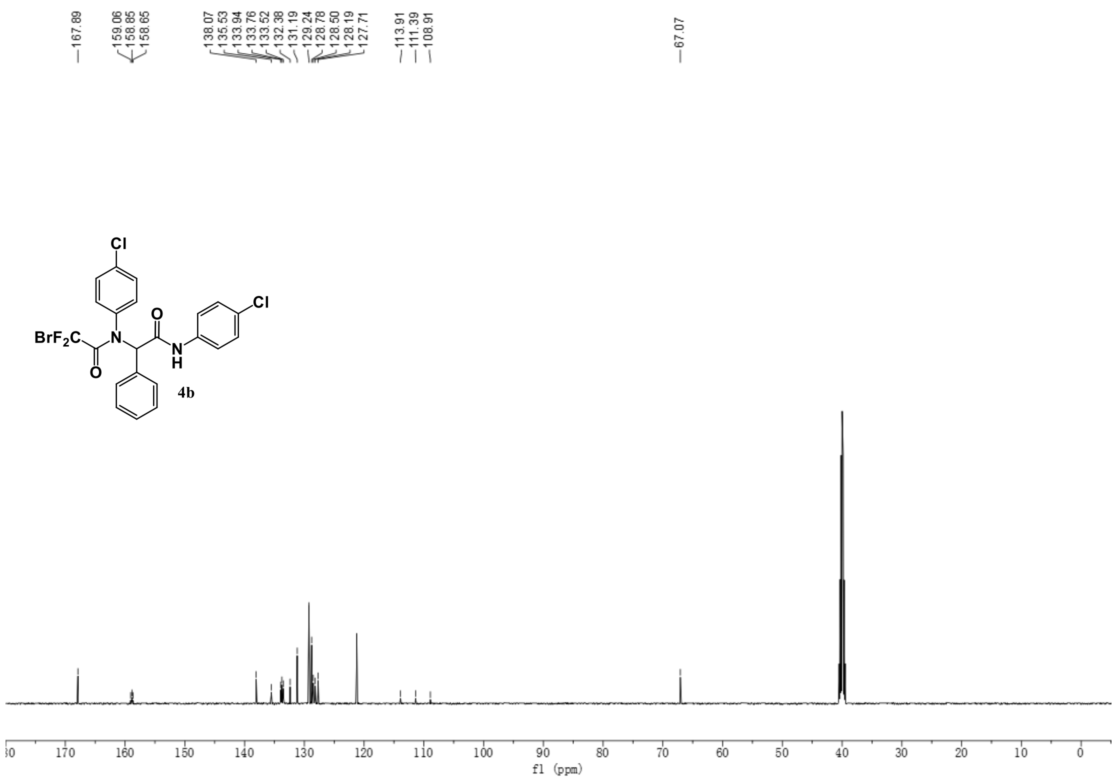
167.89
159.06
158.85
158.65
138.07
135.53
133.94
133.76
133.52
132.38
131.19
129.24
128.78
128.50
128.19
127.71
113.91
111.39
108.91
67.07
Cl
Cl
BrF2C
N
O
O
N
H
4b
f1 (ppm)

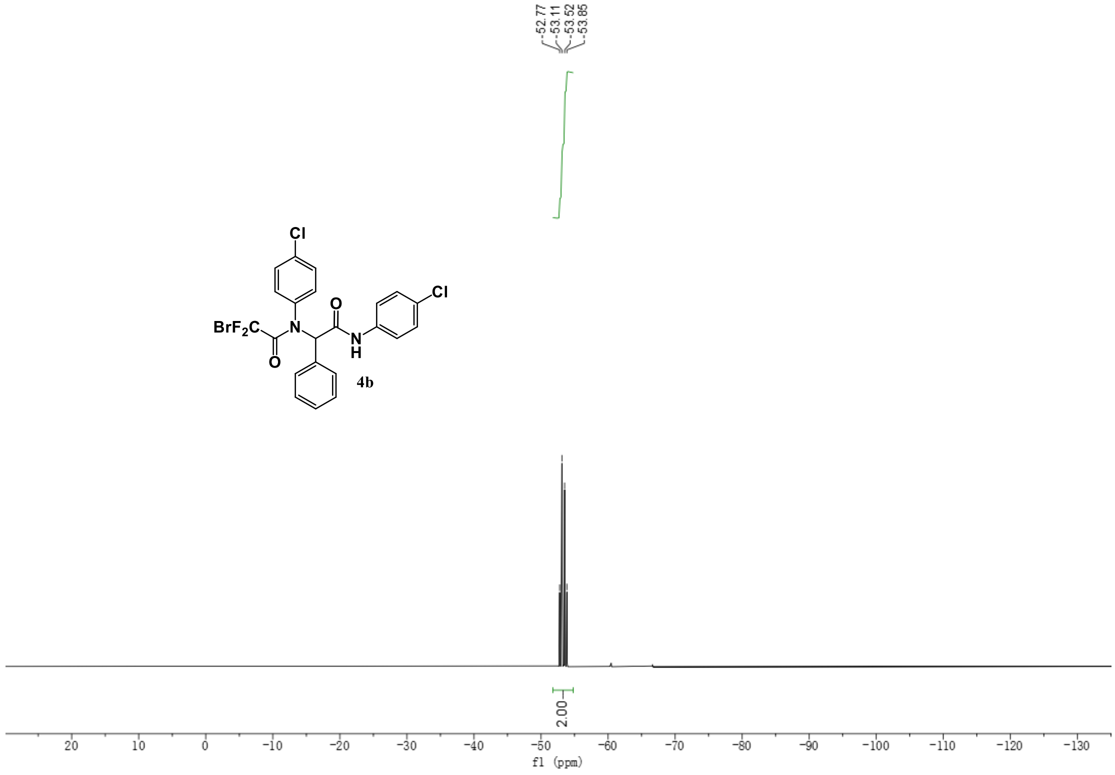
-52.77
-53.11
-53.52
-53.85
Cl
Cl
BrF2C
N
O
O
N
H
4b
2.00
f1 (ppm)

^{1}H NMR (500 MHz, DMSO), ^{13}C NMR (125 MHz, DMSO) and ^{19}F NMR (471 MHz, DMSO) spectra for 4c

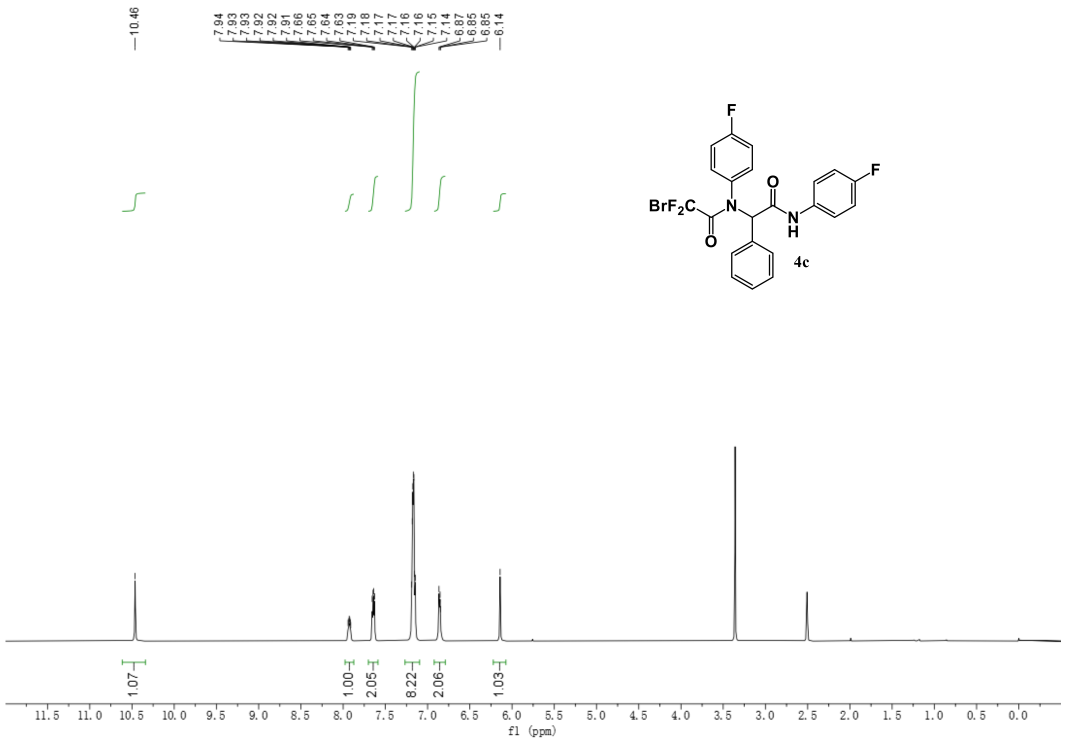

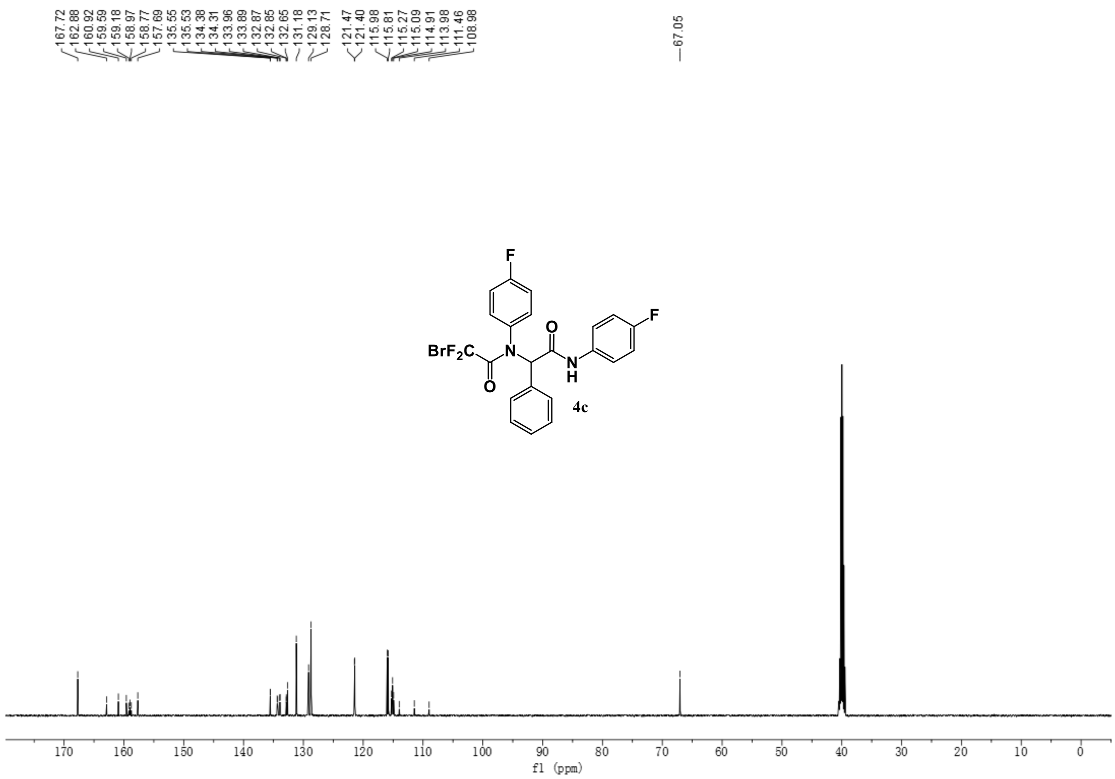

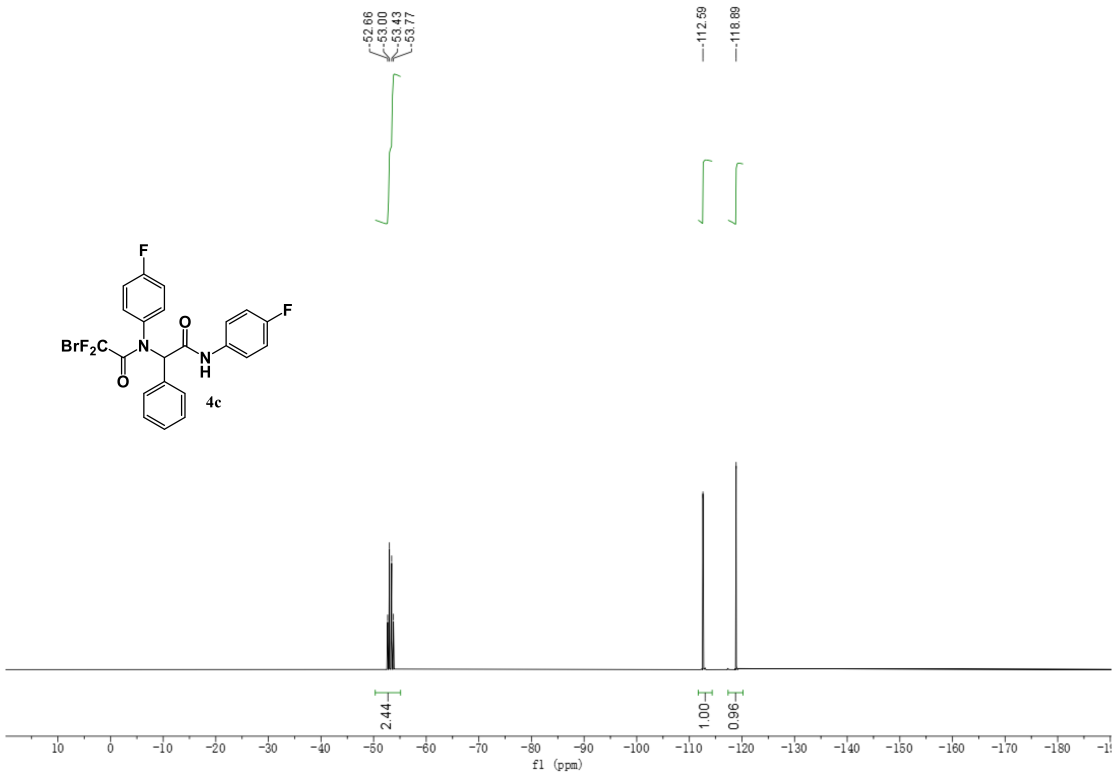

^{1}H NMR (500 MHz, DMSO), ^{13}C NMR (150 MHz, DMSO) and ^{19}F NMR (471 MHz, DMSO) spectra for 4d

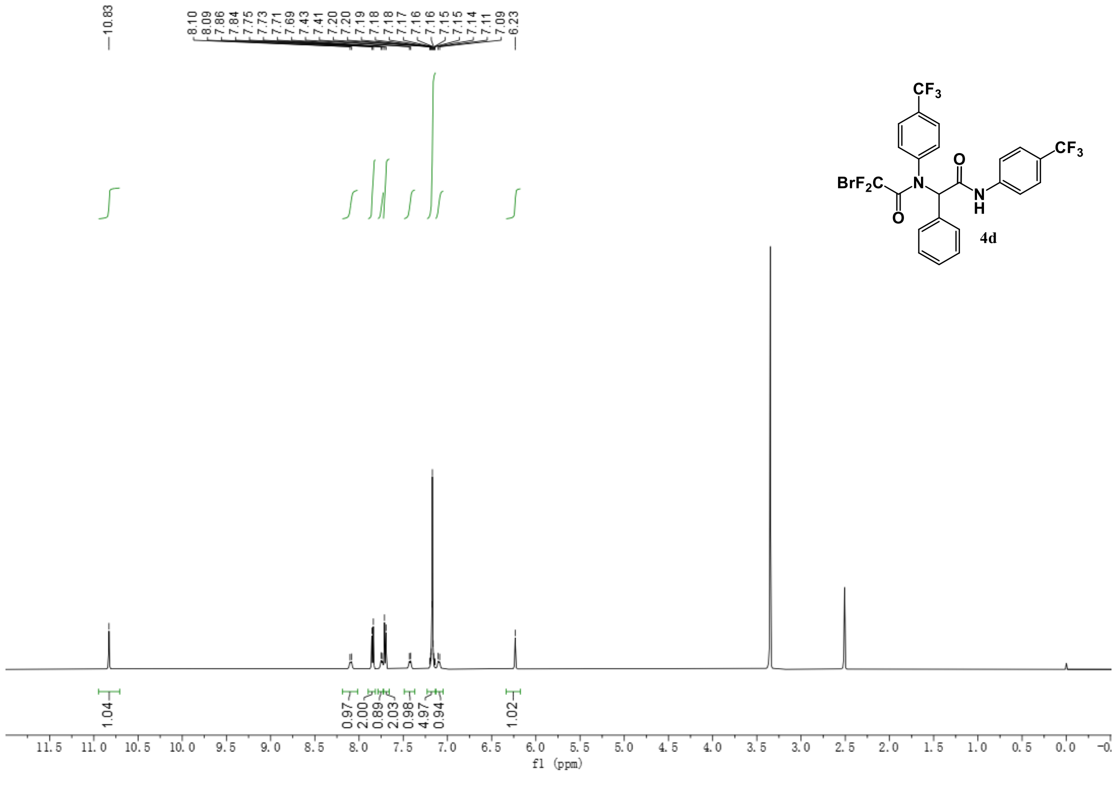

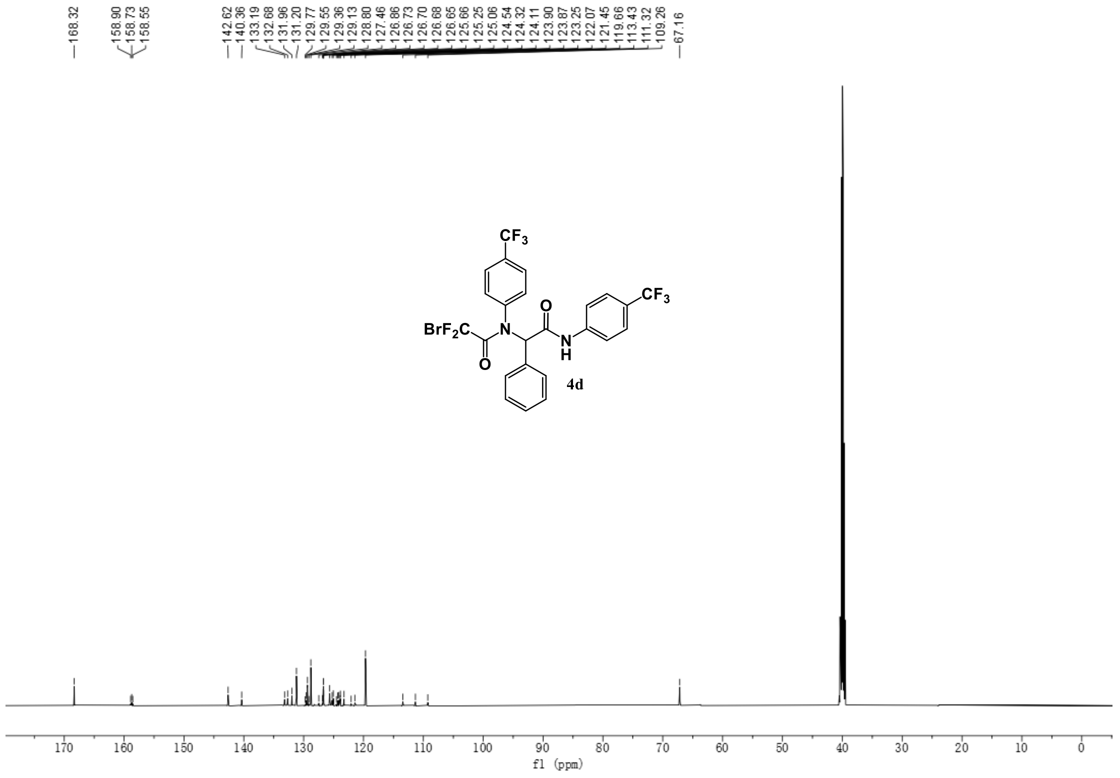
168.32
158.90
158.73
158.55
142.62
140.36
133.19
132.68
131.96
131.20
129.77
129.55
129.36
129.13
128.80
127.46
126.86
126.73
126.70
126.68
126.65
125.66
125.25
125.06
124.54
124.32
124.11
123.90
123.87
123.25
122.07
121.45
119.66
113.43
111.32
109.26
67.16
CF3
CF3
BrF2C
N
O
N
H
O
4d
fl (ppm)

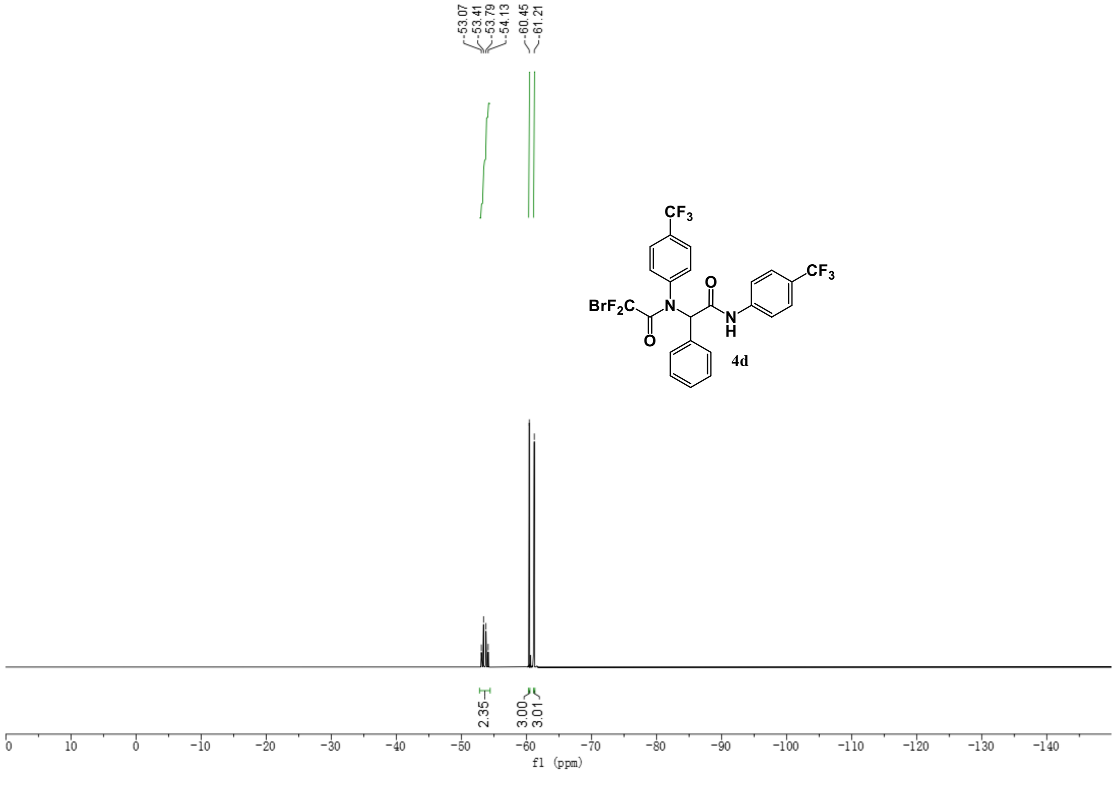
-53.07
-53.41
-53.79
-54.13
-60.45
-61.21
CF3
CF3
BrF2C
N
O
N
H
O
4d
2.35
3.00
3.01
fl (ppm)

^{1}H NMR (500 MHz, DMSO), ^{13}C NMR (125 MHz, DMSO) and ^{19}F NMR (471 MHz, DMSO) spectra for 4e

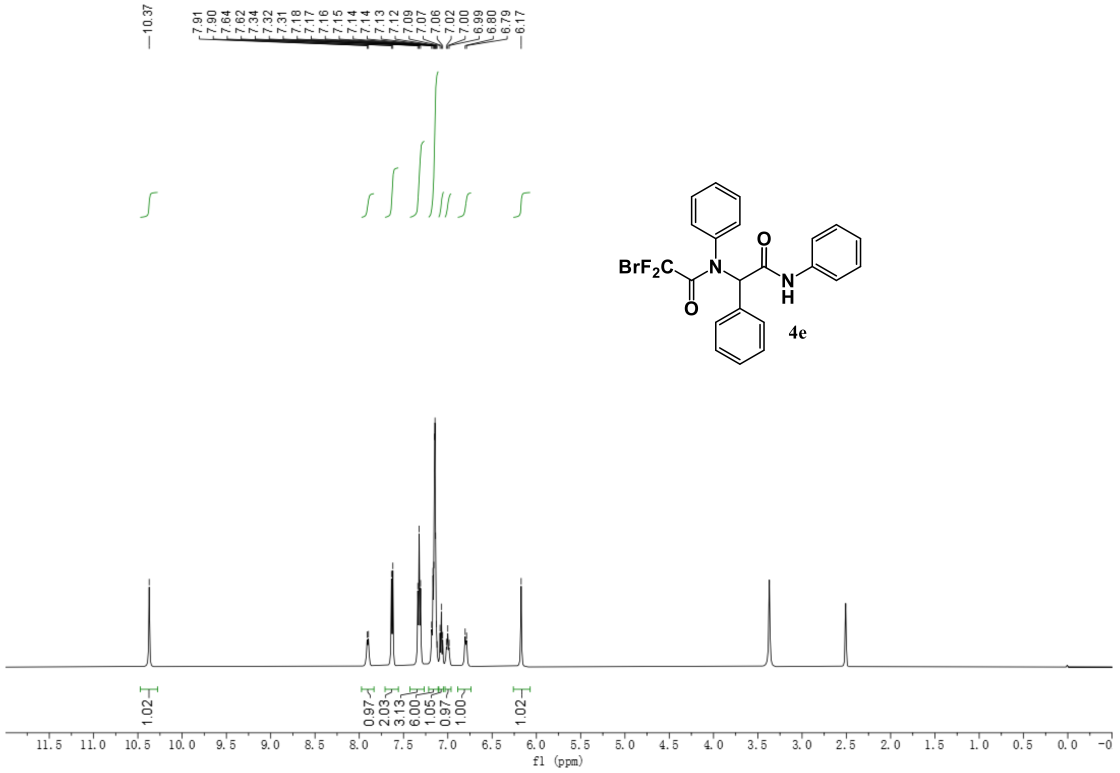

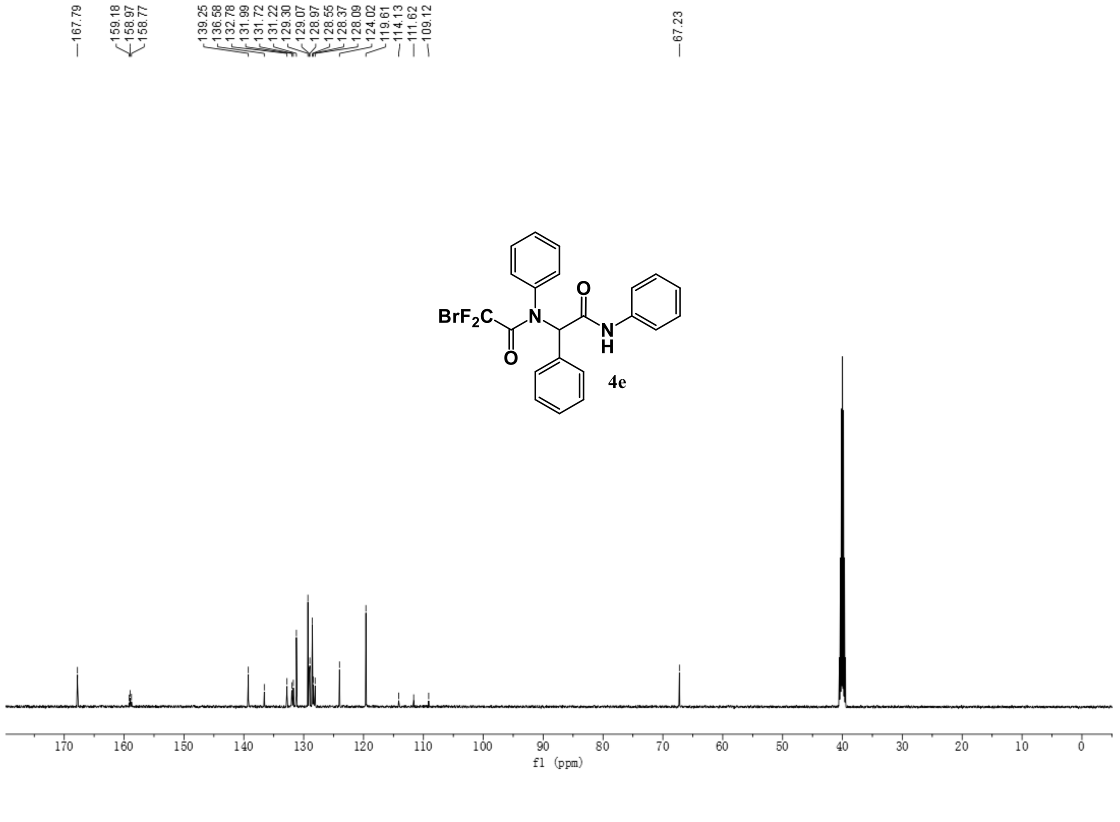

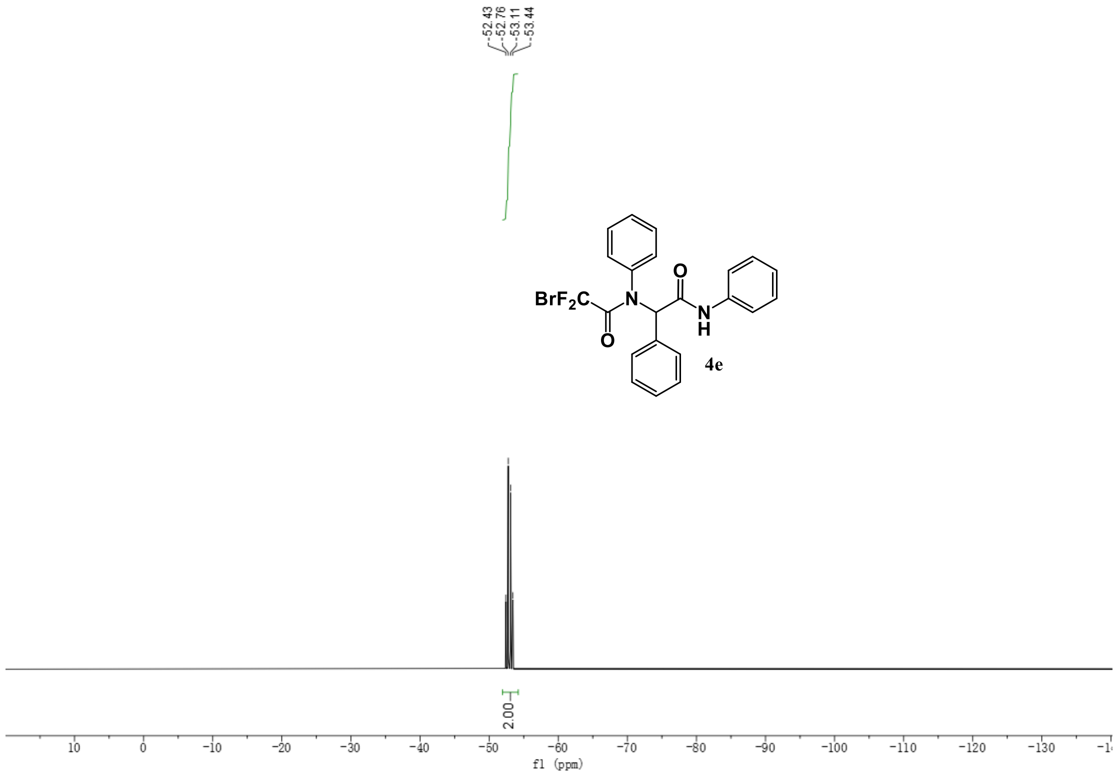

^{1}H NMR (500 MHz, DMSO), ^{13}C NMR (150 MHz, DMSO) and ^{19}F NMR (471 MHz, DMSO) spectra for 4f

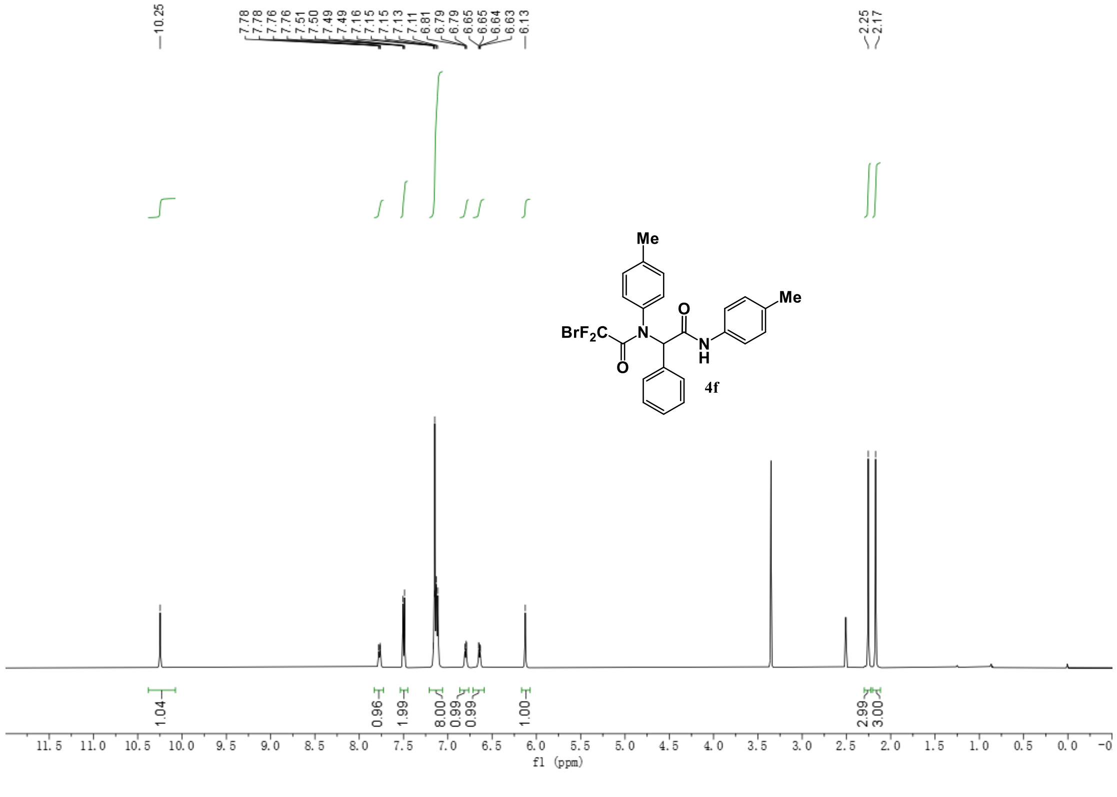

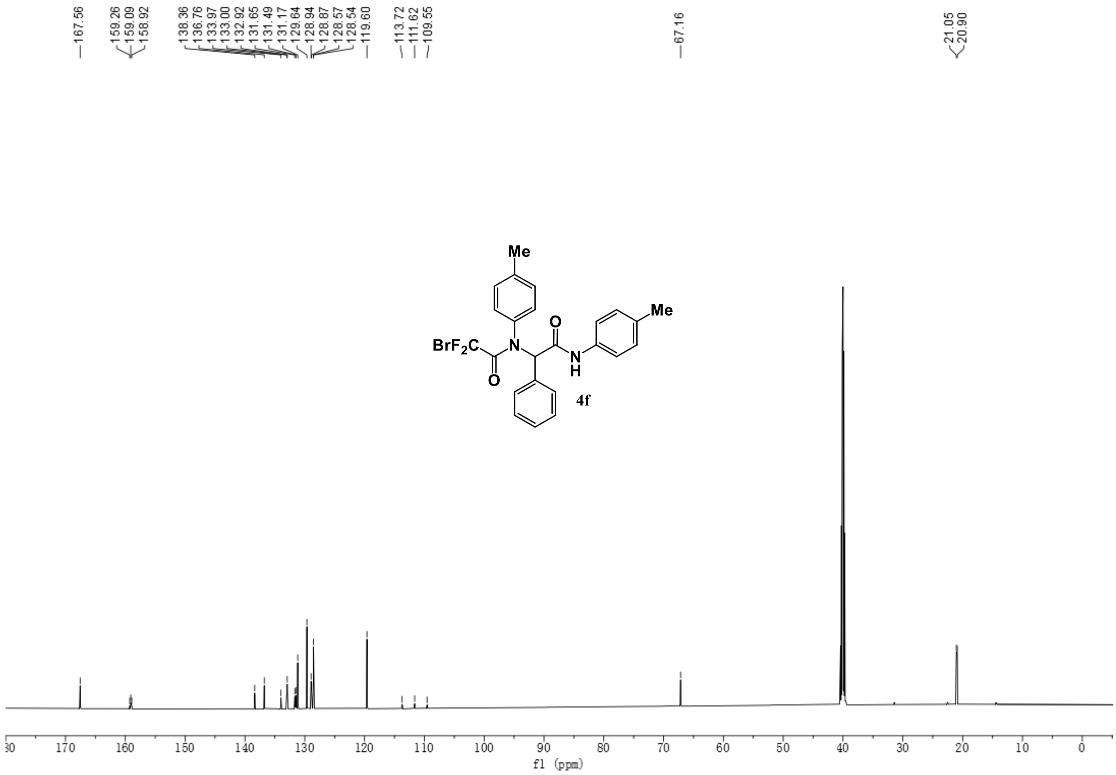
167.56
159.26
159.09
158.92
138.36
136.76
133.97
133.00
132.92
131.65
131.49
131.17
129.64
128.94
128.87
128.57
128.54
119.60
113.72
111.62
109.55
67.16
21.05
20.90
Me
Me
O
BrF2C
N
N
H
O
4f
f1 (ppm)

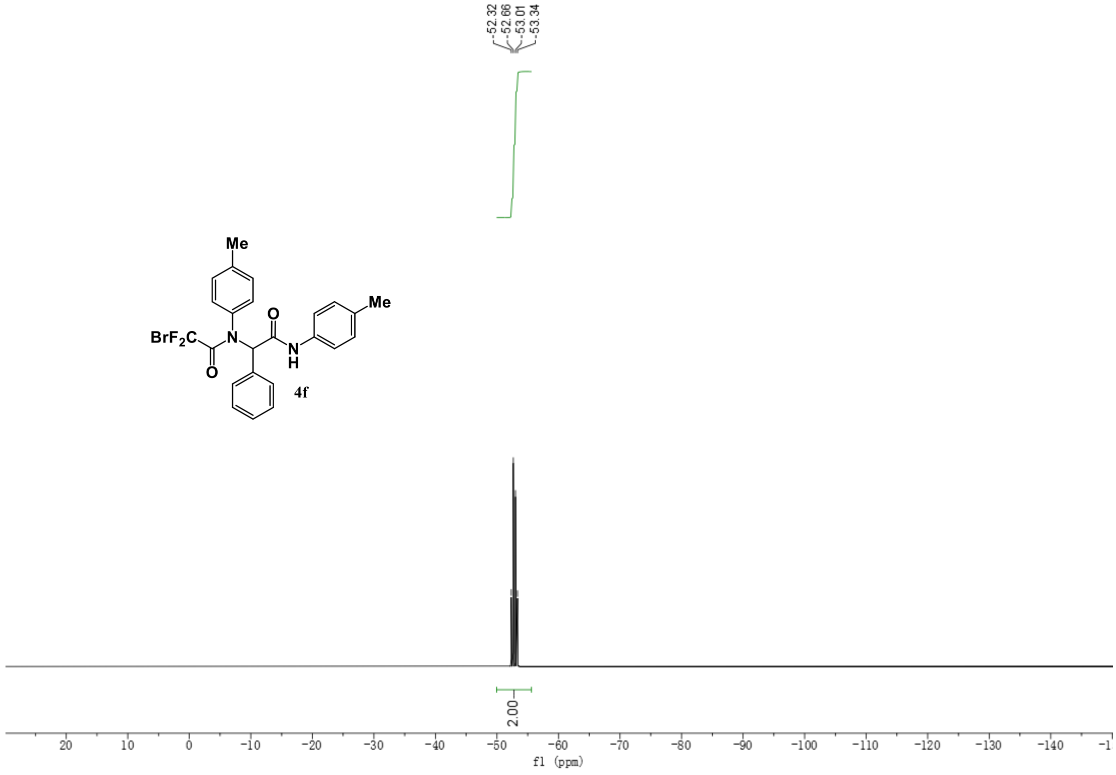
-52.32
-52.66
-53.01
-53.34
Me
Me
O
BrF2C
N
N
H
O
4f
2.00
f1 (ppm)

^{1}H NMR (500 MHz, CDCl$_3$), ^{13}C NMR (125 MHz, CDCl$_3$) and ^{19}F NMR (471 MHz, CDCl$_3$) spectra for 4g

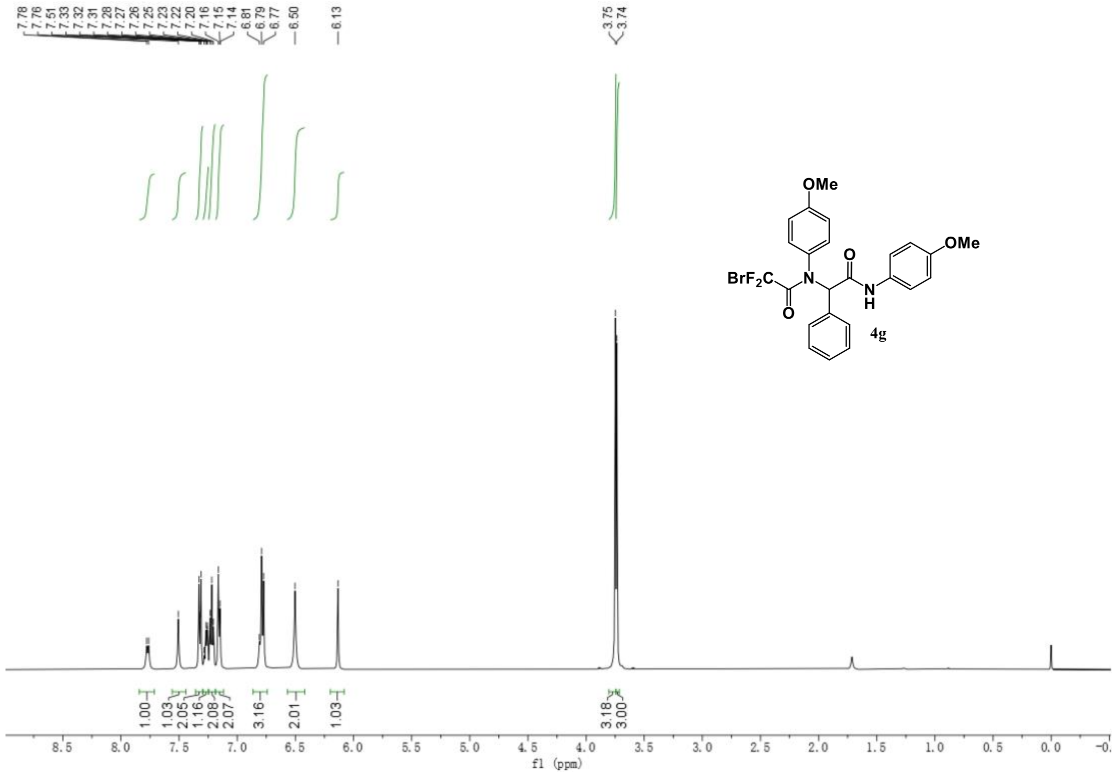

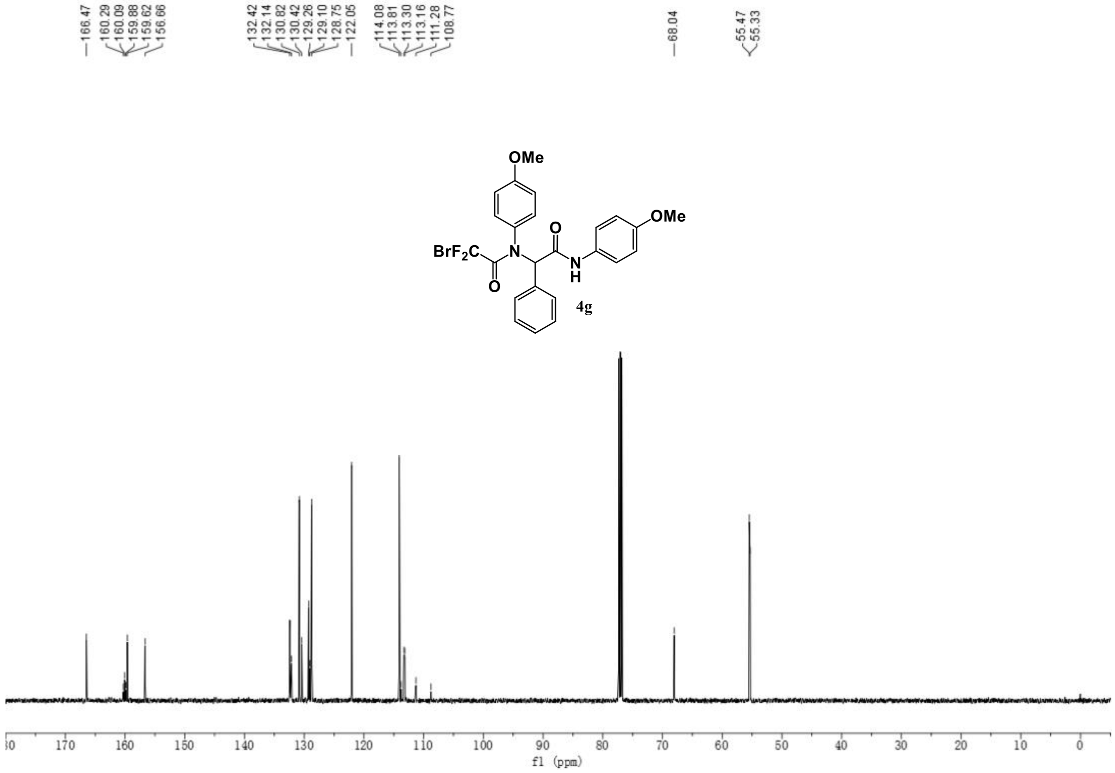

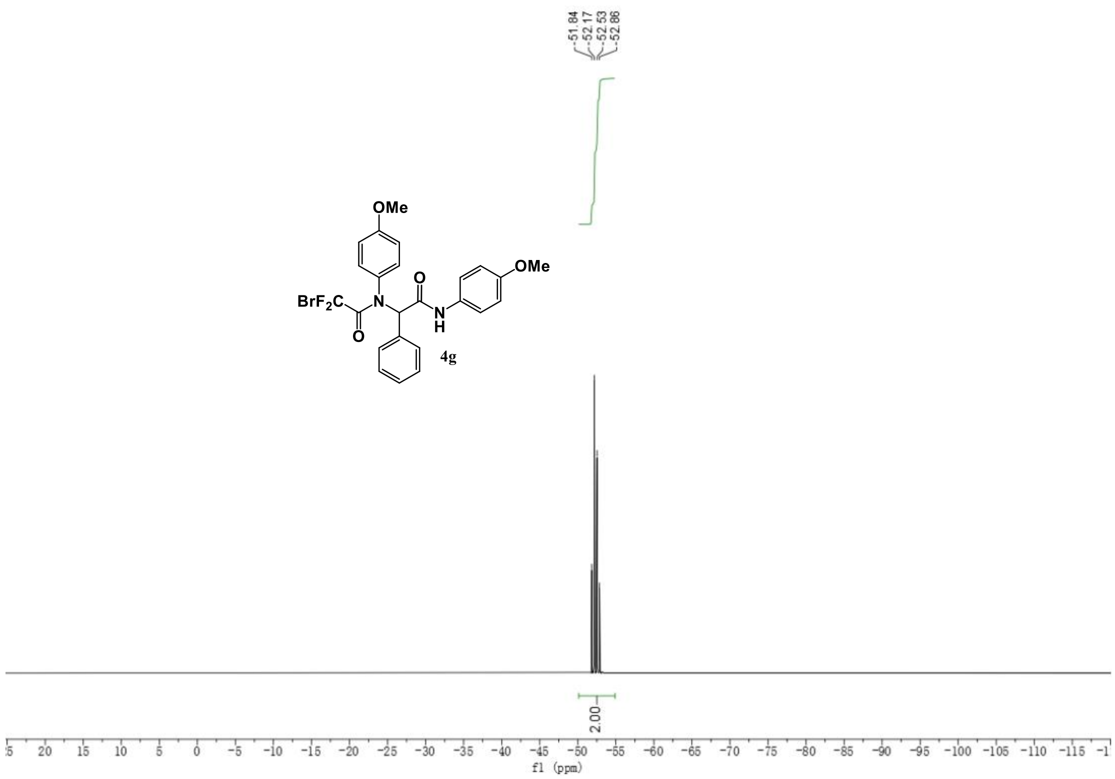

^{1}H NMR (500 MHz, DMSO), ^{13}C NMR (125 MHz, DMSO) and ^{19}F NMR (471 MHz, DMSO) spectra for 4h

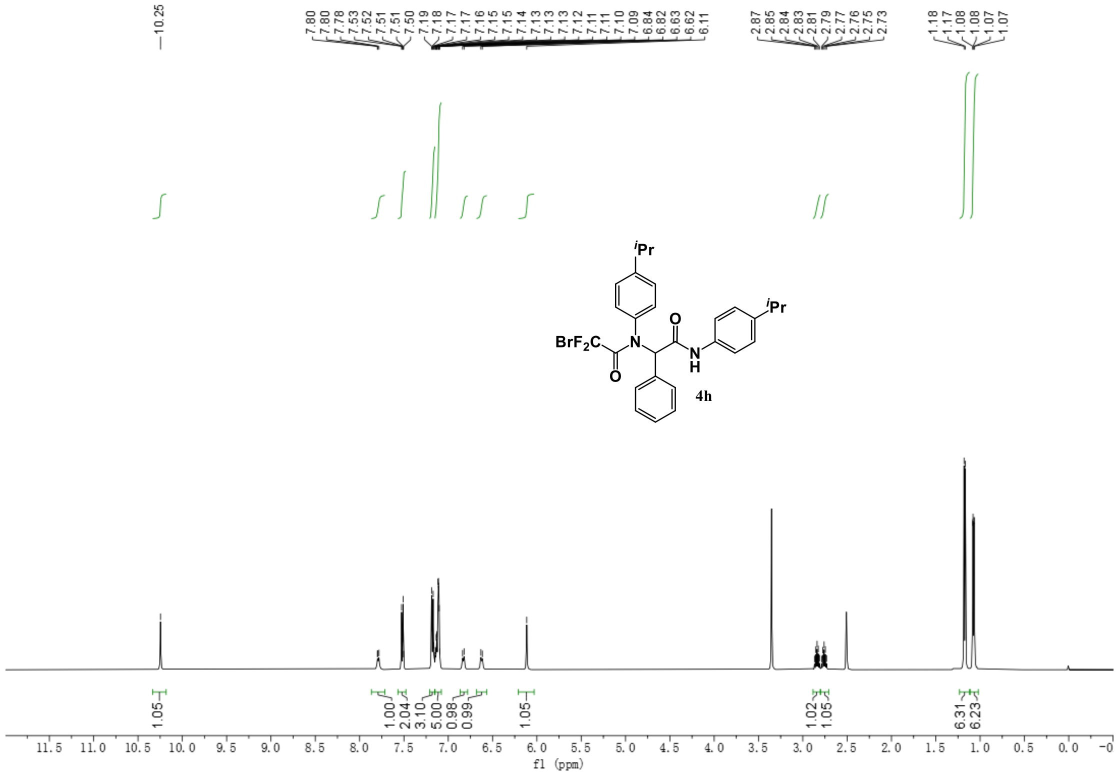

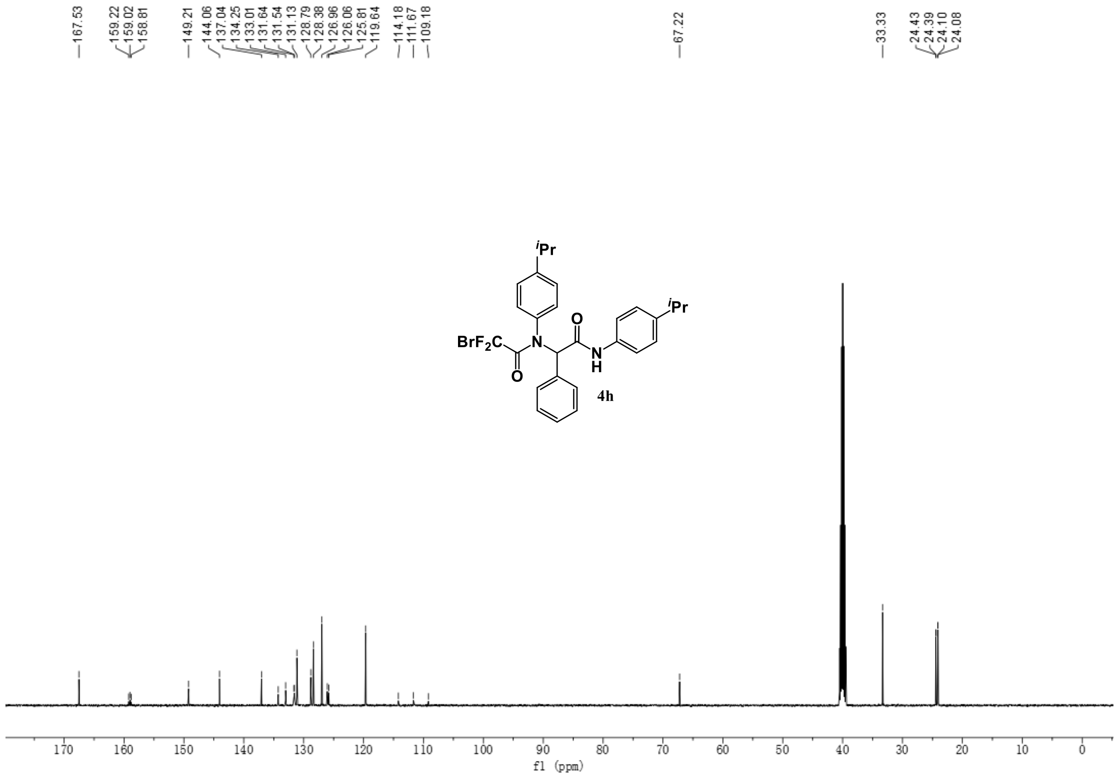

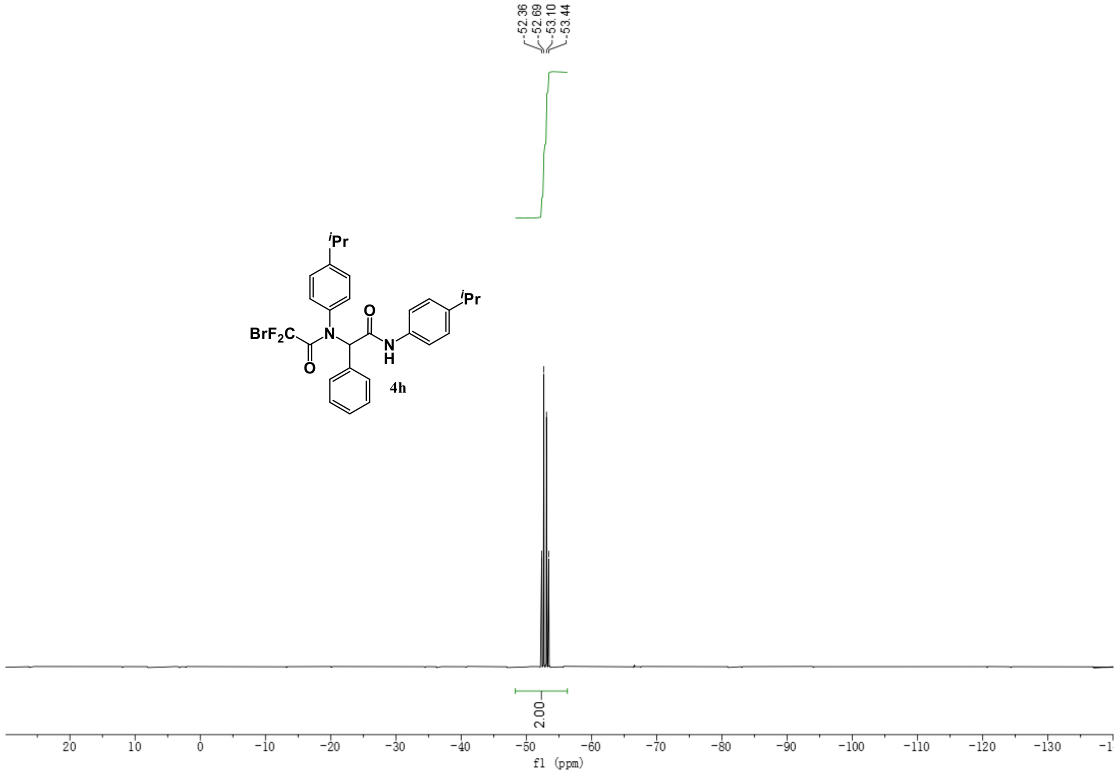

^{1}H NMR (500 MHz, DMSO), ^{13}C NMR (125 MHz, DMSO) and ^{19}F NMR (471 MHz, DMSO) spectra for 4i

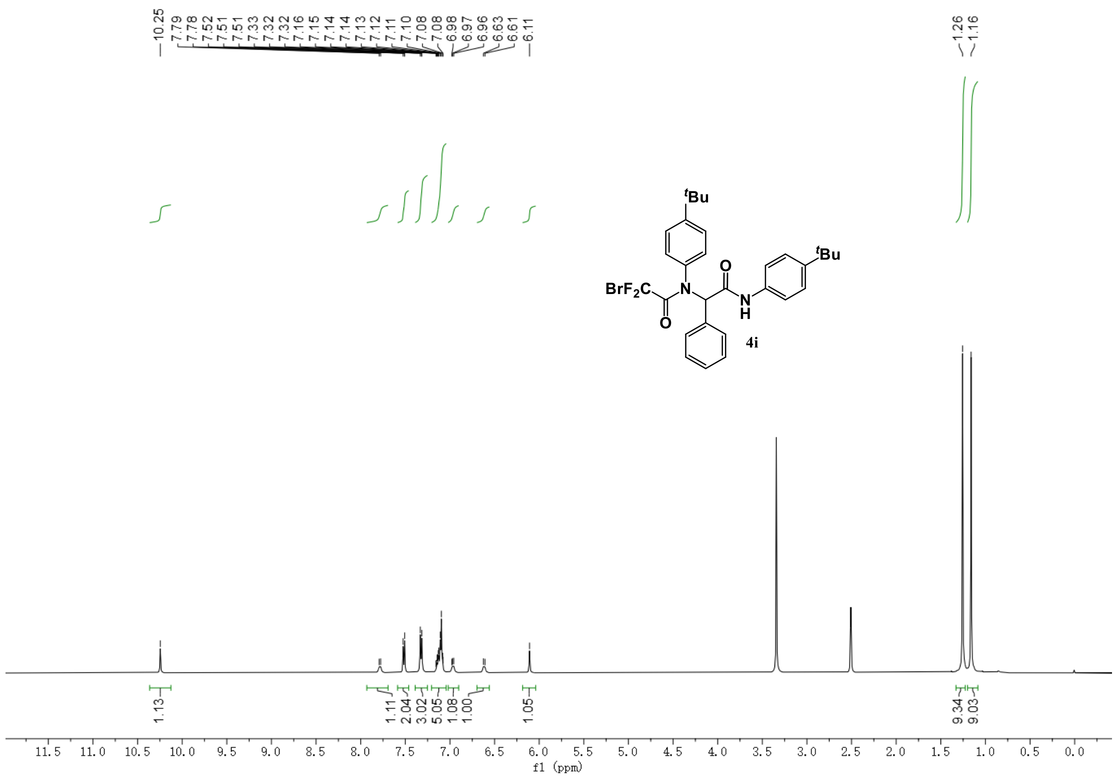

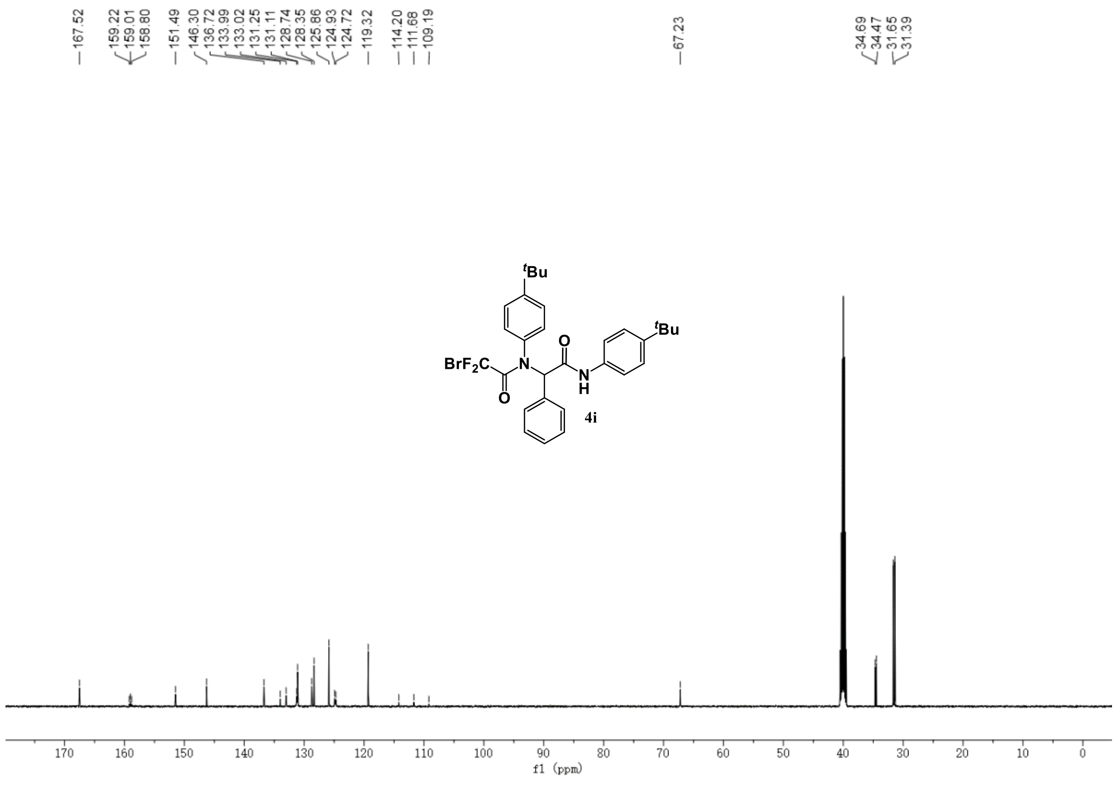

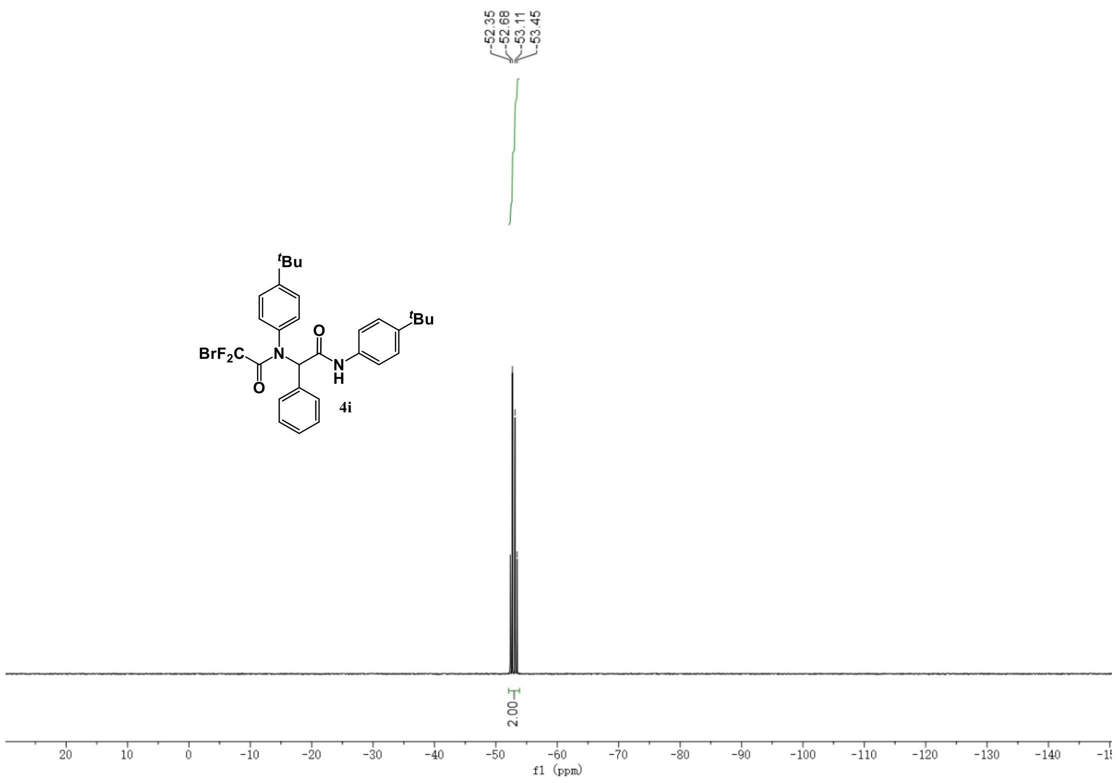

^{1}H NMR (500 MHz, DMSO), ^{13}C NMR (125 MHz, DMSO) and ^{19}F NMR (471 MHz, DMSO) spectra for 4j

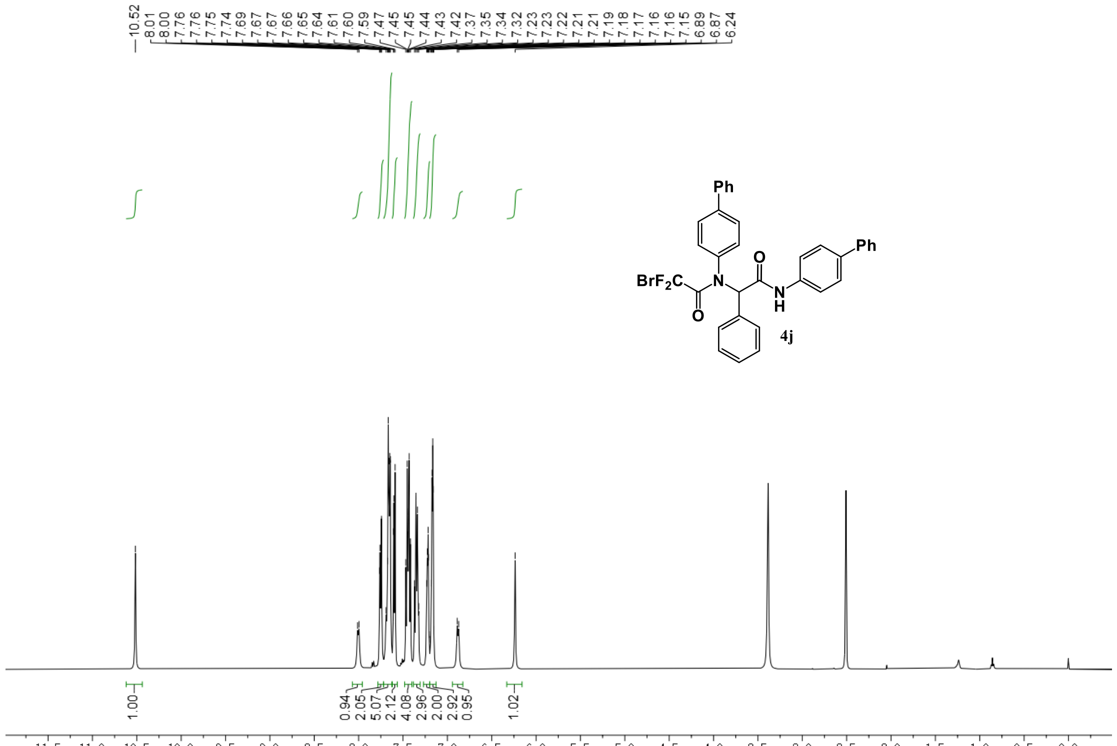

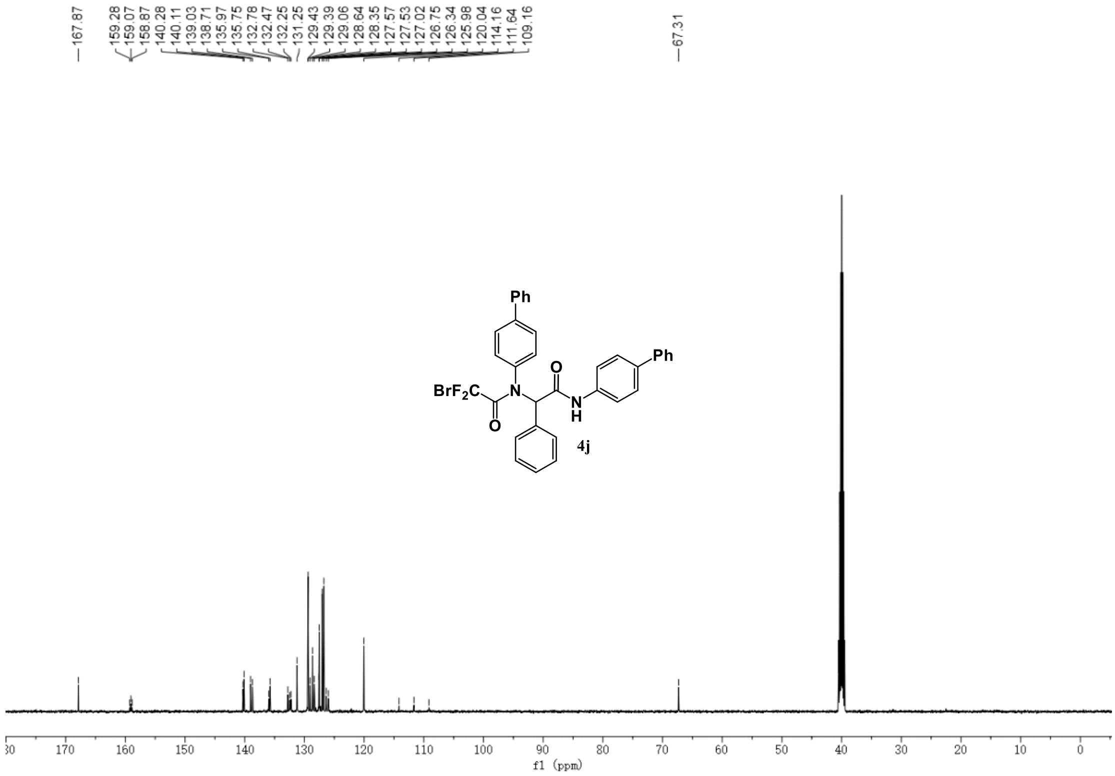

167.87
159.28
159.07
158.87
140.28
140.11
139.03
138.71
135.97
135.75
132.78
132.47
132.25
131.25
129.43
129.39
129.06
128.64
128.35
127.57
127.53
127.02
126.75
126.34
125.98
120.04
114.16
111.64
109.16
67.31
Ph
Ph
BrF2C
N
O
O
N
H
4j
f1 (ppm)

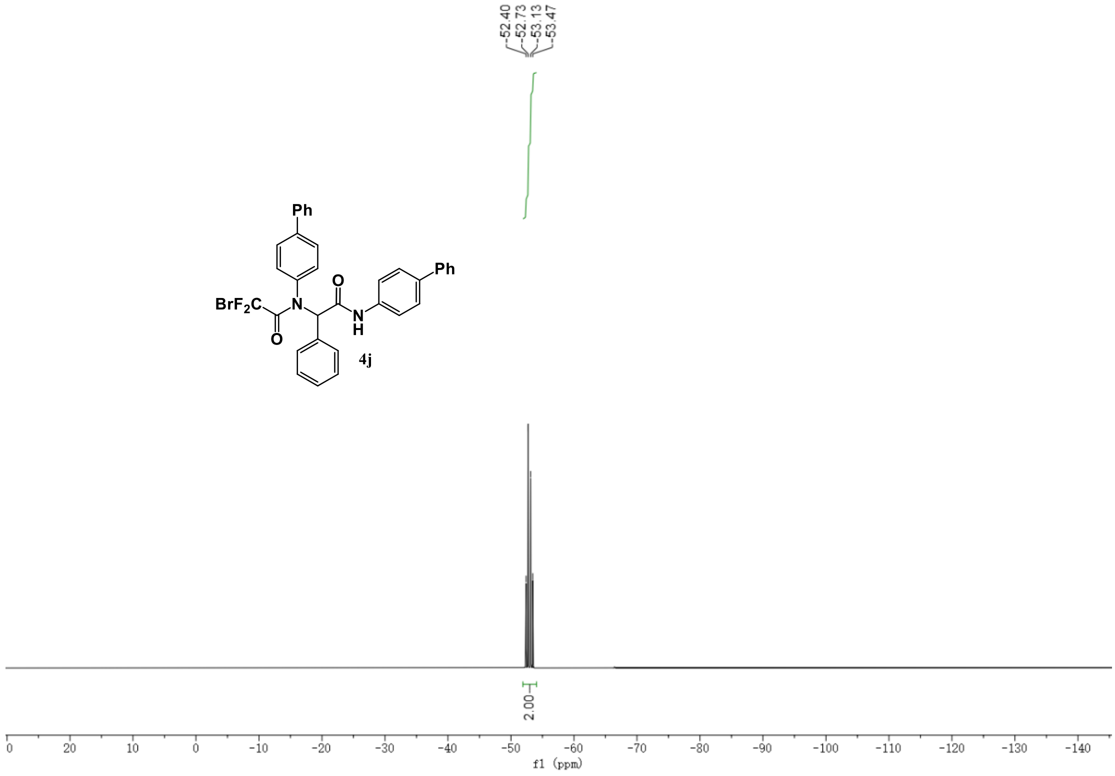

-52.40
-52.73
-53.13
-53.47
Ph
Ph
BrF2C
N
O
O
N
H
4j
2.00
f1 (ppm)

^{1}H NMR (500 MHz, DMSO), ^{13}C NMR (150 MHz, DMSO) and ^{19}F NMR (471 MHz, DMSO) spectra for 4k

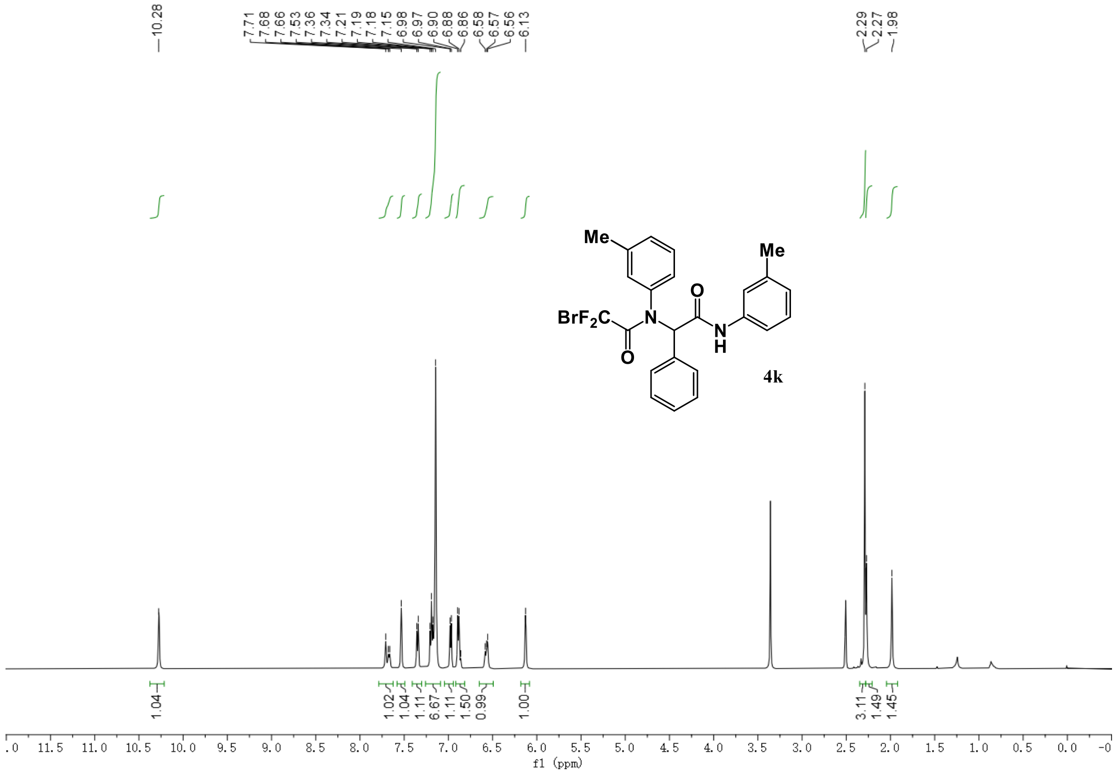

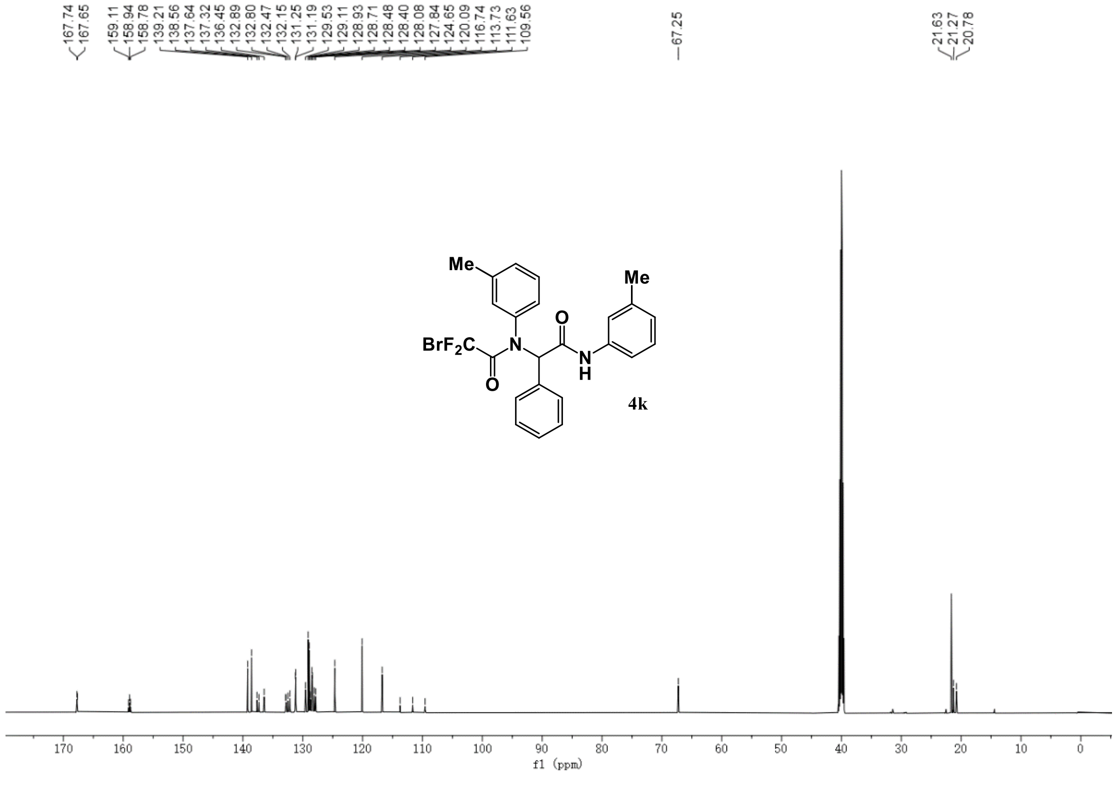

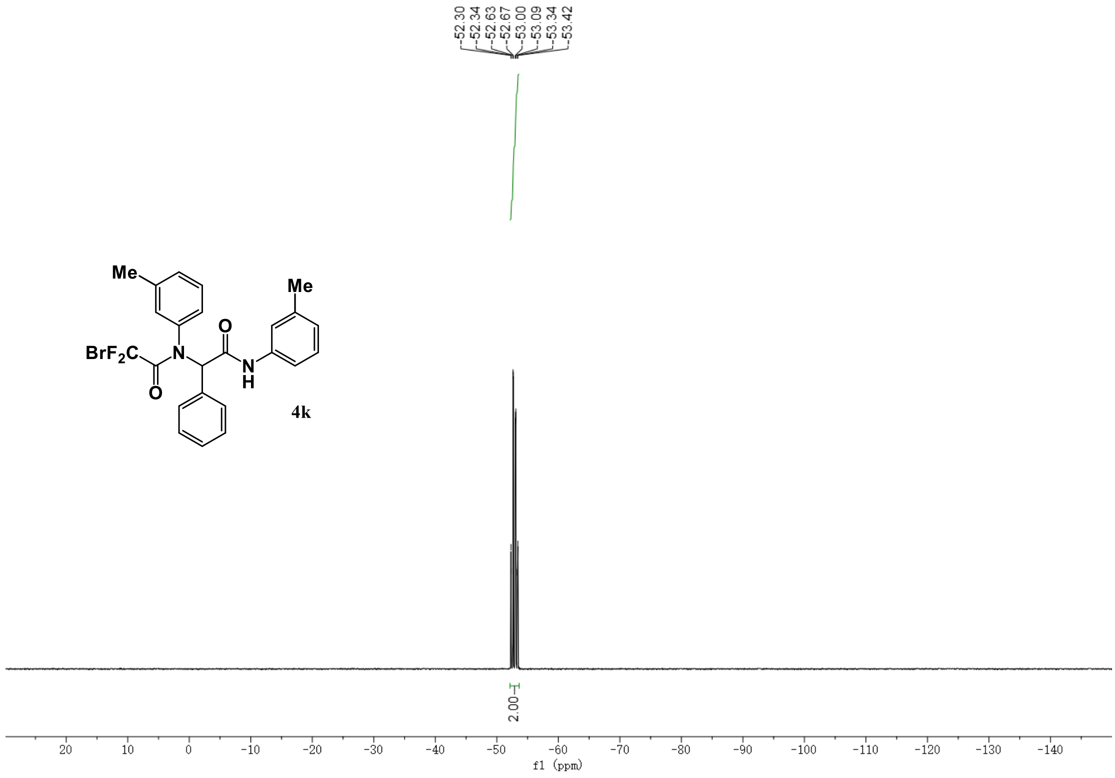

^{1}H NMR (500 MHz, DMSO), ^{13}C NMR (125 MHz, DMSO) and ^{19}F NMR (471 MHz, DMSO) spectra for 4l

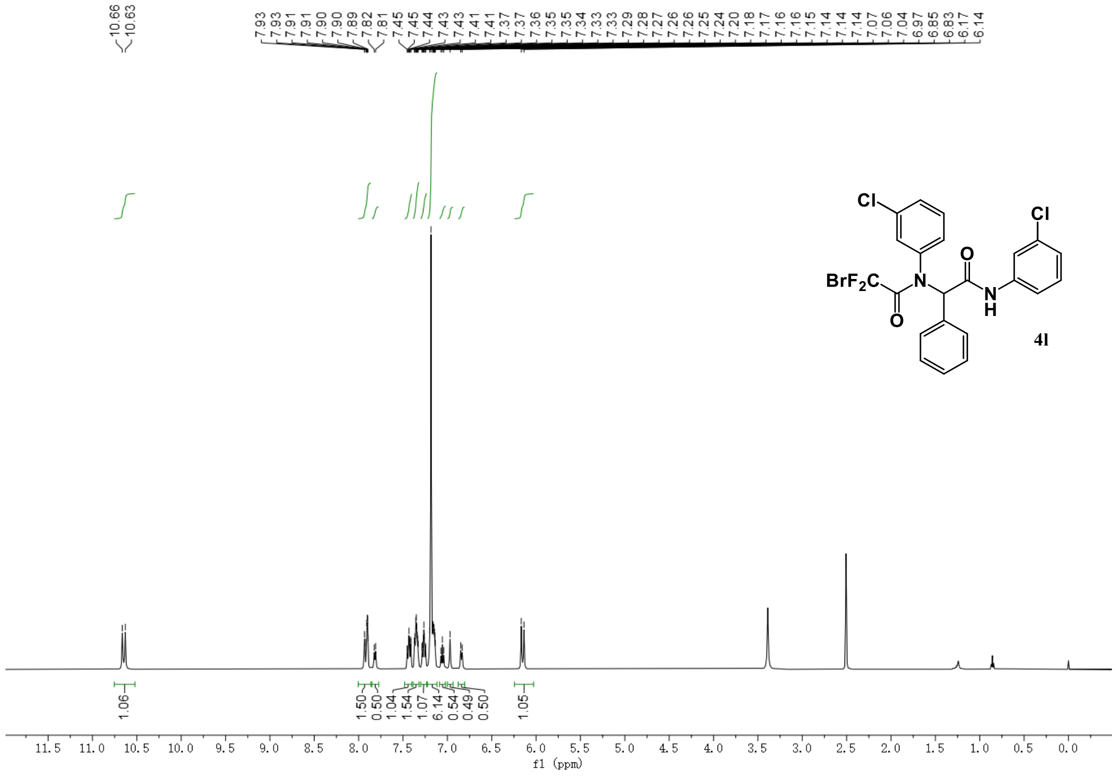

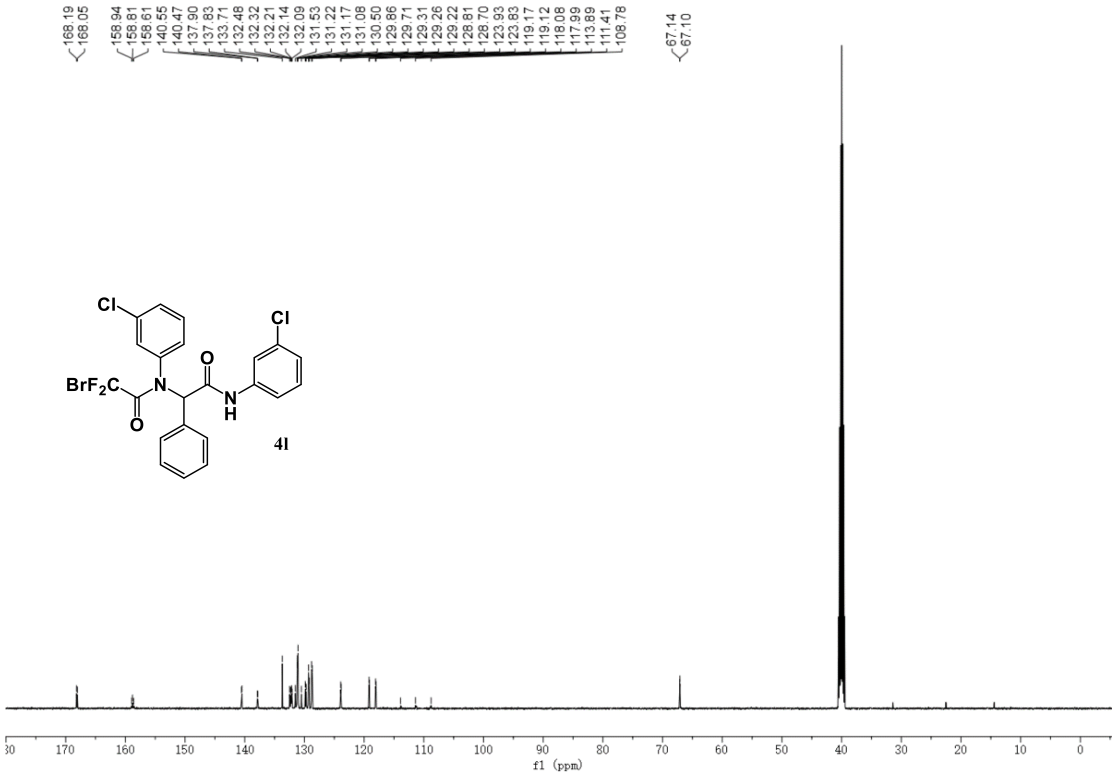
168.19
168.05
158.94
158.81
158.61
140.55
140.47
137.90
137.83
133.71
132.48
132.32
132.21
132.14
132.09
131.53
131.22
131.17
131.08
130.50
129.86
129.71
129.31
129.26
129.22
128.81
128.70
123.93
123.83
119.17
119.12
118.08
117.99
113.89
111.41
108.78
67.14
67.10
Cl
Cl
O
BrF_2C
N
N
H
O
4l
f1 (ppm)

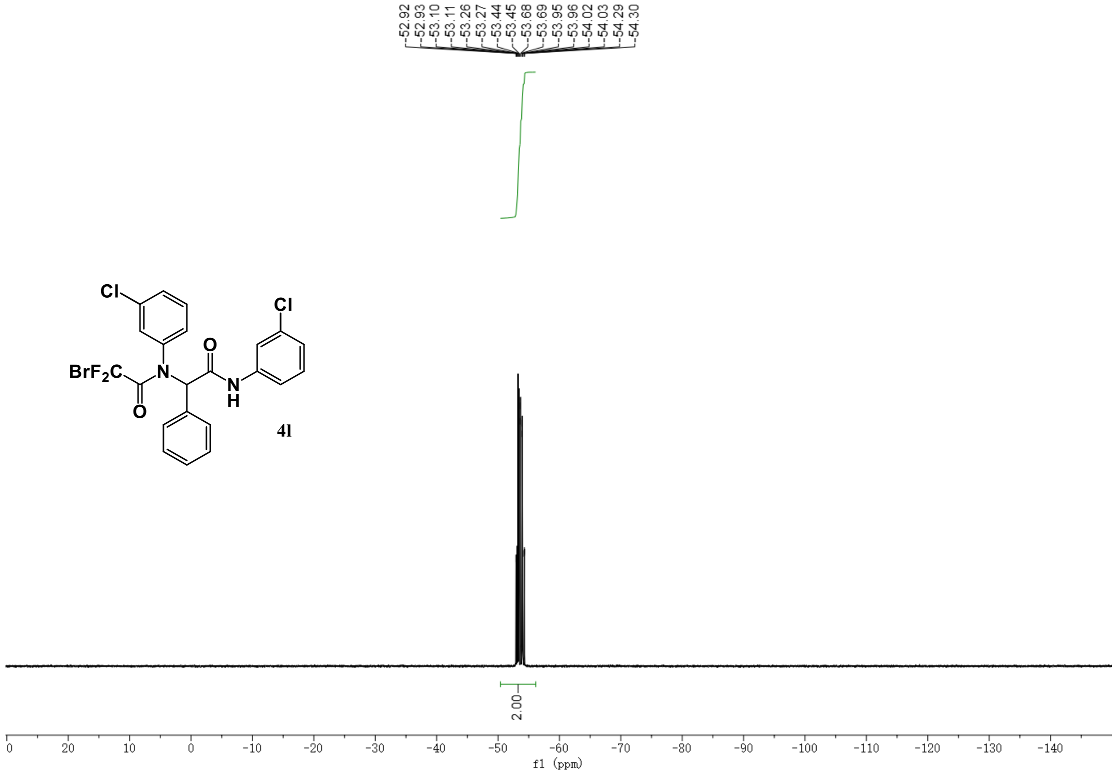
-52.92
-52.93
-53.10
-53.11
-53.26
-53.27
-53.44
-53.45
-53.68
-53.69
-53.95
-53.96
-54.02
-54.03
-54.29
-54.30
Cl
Cl
O
BrF_2C
N
N
H
O
4l
2.00
f1 (ppm)

^{1}H NMR (500 MHz, CDCl3), ^{13}C NMR (150 MHz, DMSO) and ^{19}F NMR (471 MHz, DMSO) spectra for 4m

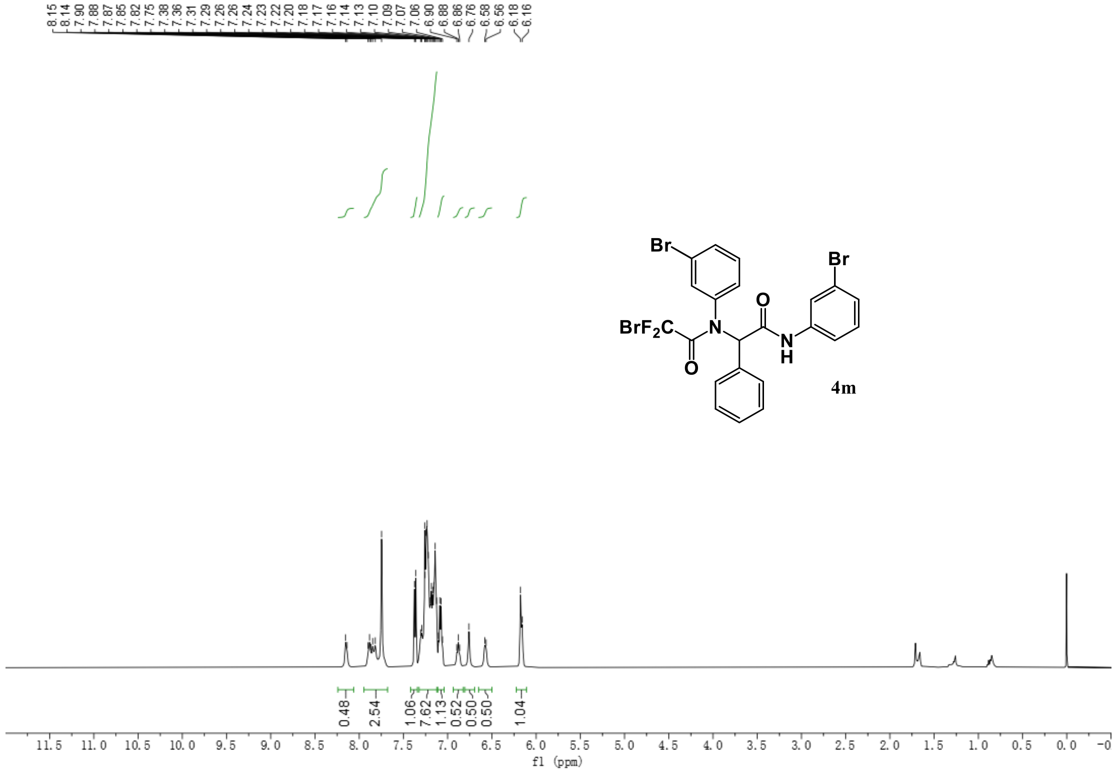

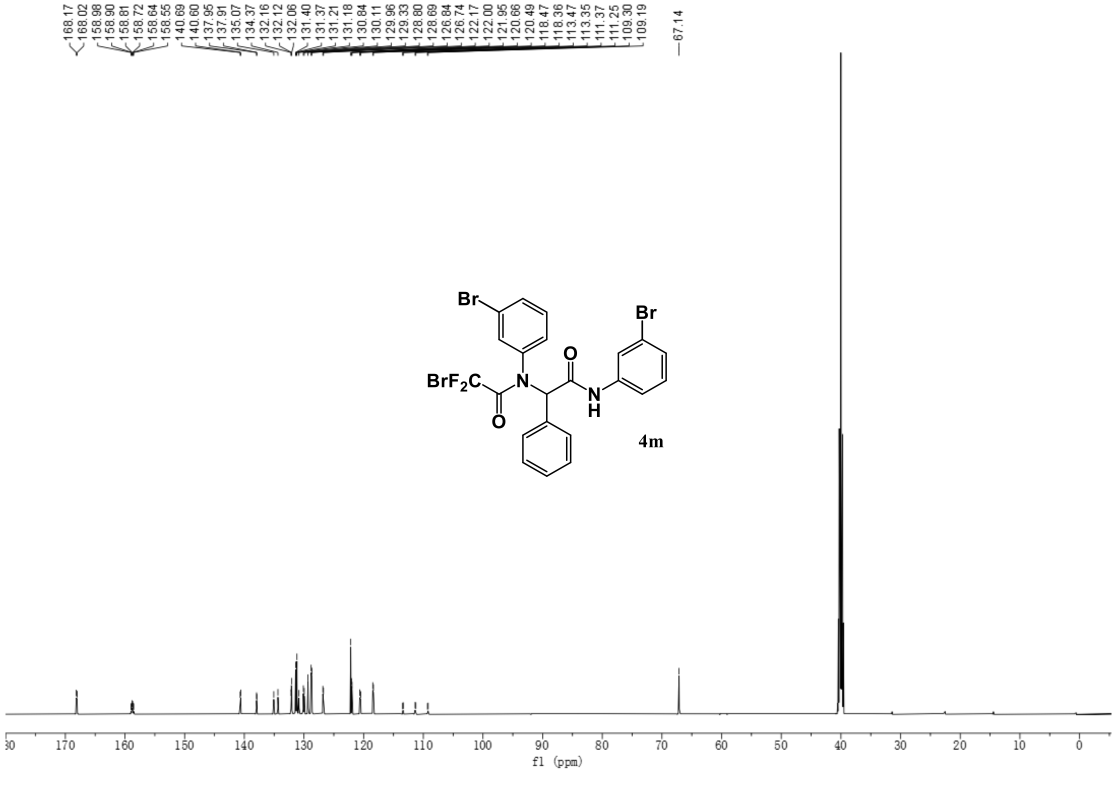

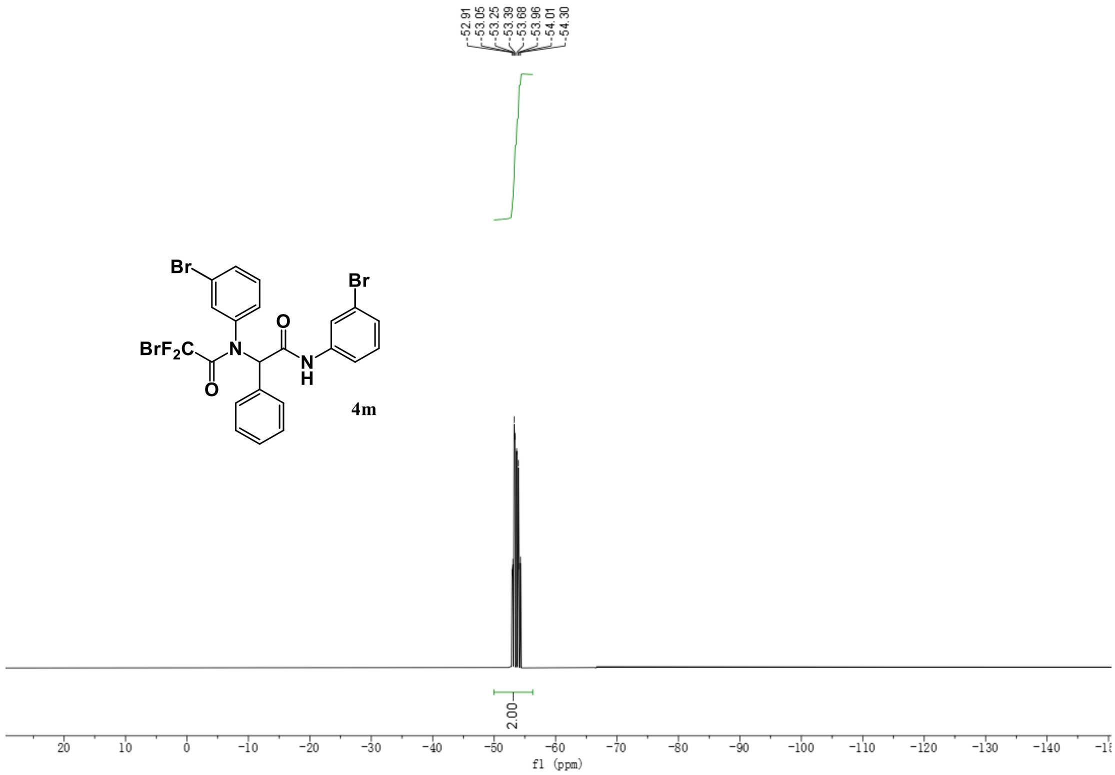

^{1}H NMR (500 MHz, DMSO), ^{13}C NMR (125 MHz, DMSO) and ^{19}F NMR (471 MHz, DMSO) spectra for 4n

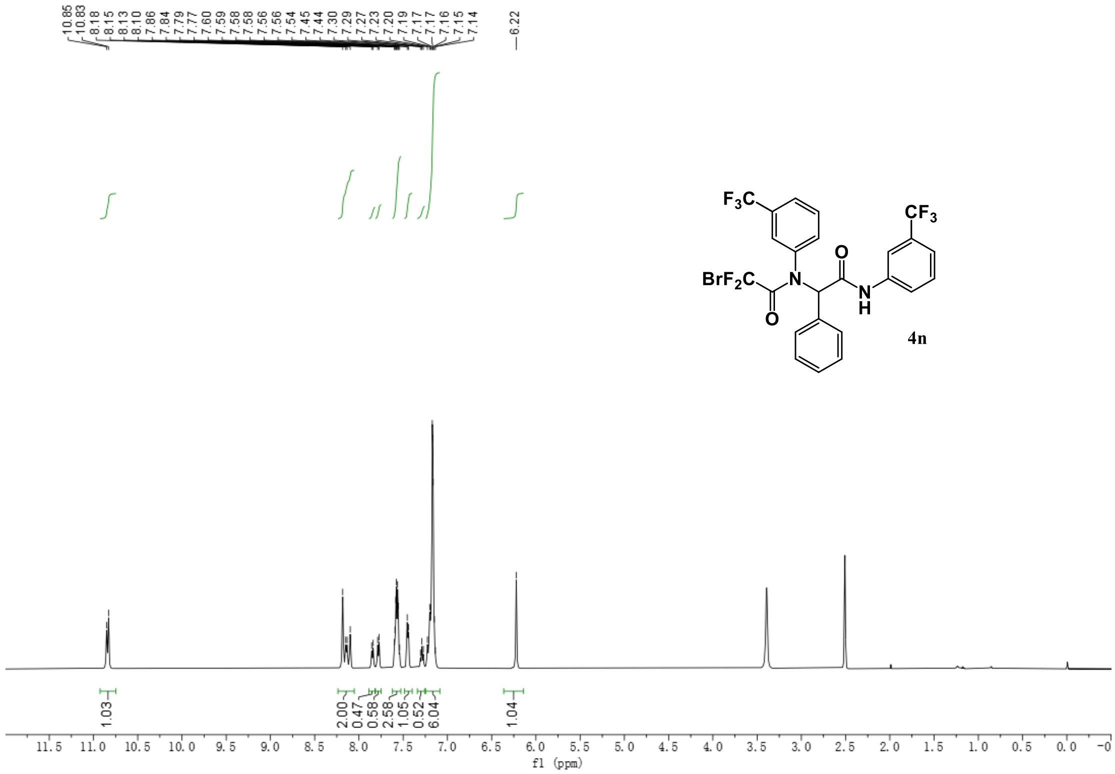

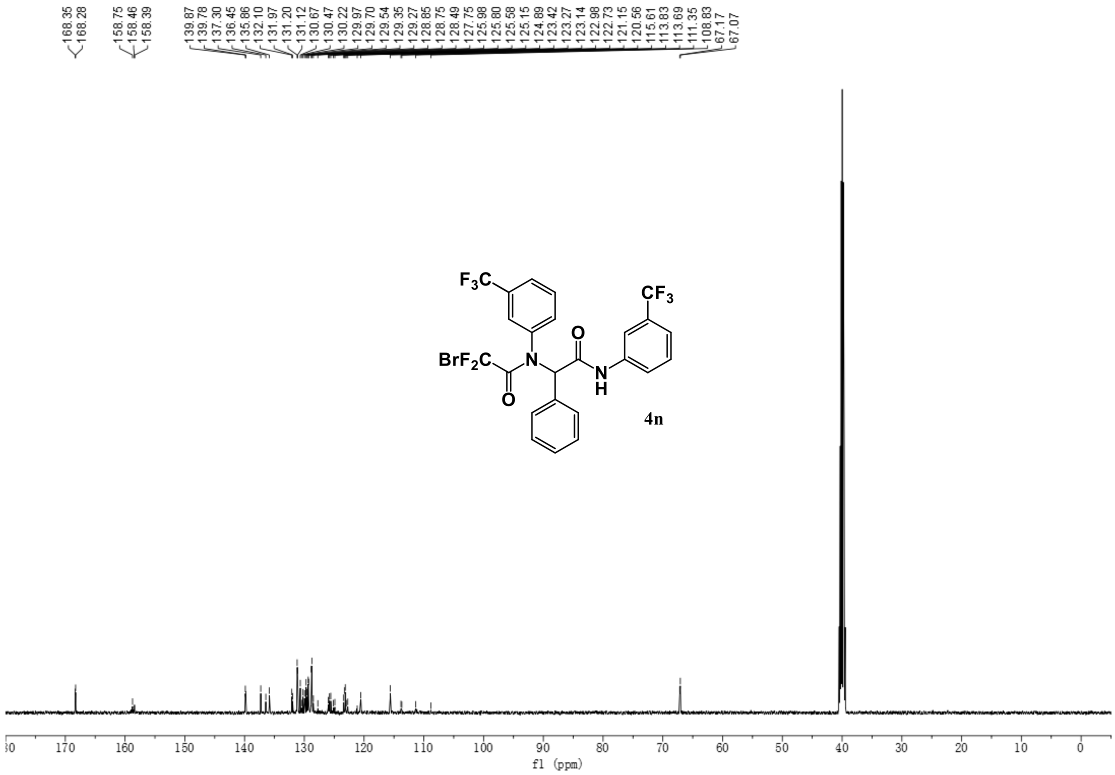
168.35
168.28
158.75
158.46
158.39
139.87
139.78
137.30
136.45
135.86
132.10
131.97
131.20
131.12
130.67
130.47
130.22
129.97
129.70
129.54
129.35
129.27
128.85
128.75
128.49
127.75
125.98
125.80
125.58
125.15
124.89
123.42
123.27
123.14
122.98
122.73
121.15
120.56
115.61
113.83
113.69
111.35
108.83
67.17
67.07
F_3C
CF_3
O
BrF_2C
N
N
H
O
4n
170
160
150
140
130
120
110
100
90
80
70
60
50
40
30
20
10
0
f1 (ppm)

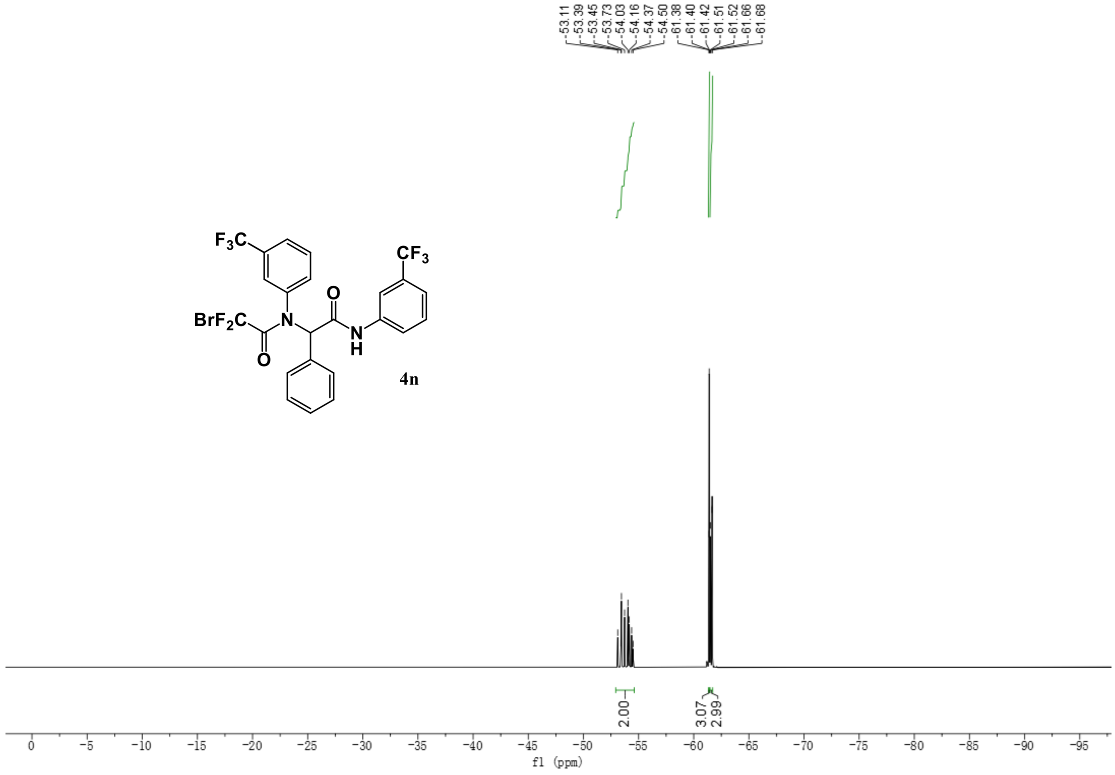
-53.11
-53.39
-53.45
-53.73
-54.03
-54.16
-54.37
-54.50
-61.38
-61.40
-61.42
-61.51
-61.52
-61.66
-61.68
F_3C
CF_3
O
BrF_2C
N
N
H
O
4n
2.00
3.07
2.99
0
-5
-10
-15
-20
-25
-30
-35
-40
-45
-50
-55
-60
-65
-70
-75
-80
-85
-90
-95
f1 (ppm)

^{1}H NMR (500 MHz, DMSO), ^{13}C NMR (150 MHz, DMSO) and ^{19}F NMR (471 MHz, DMSO) spectra for 4q

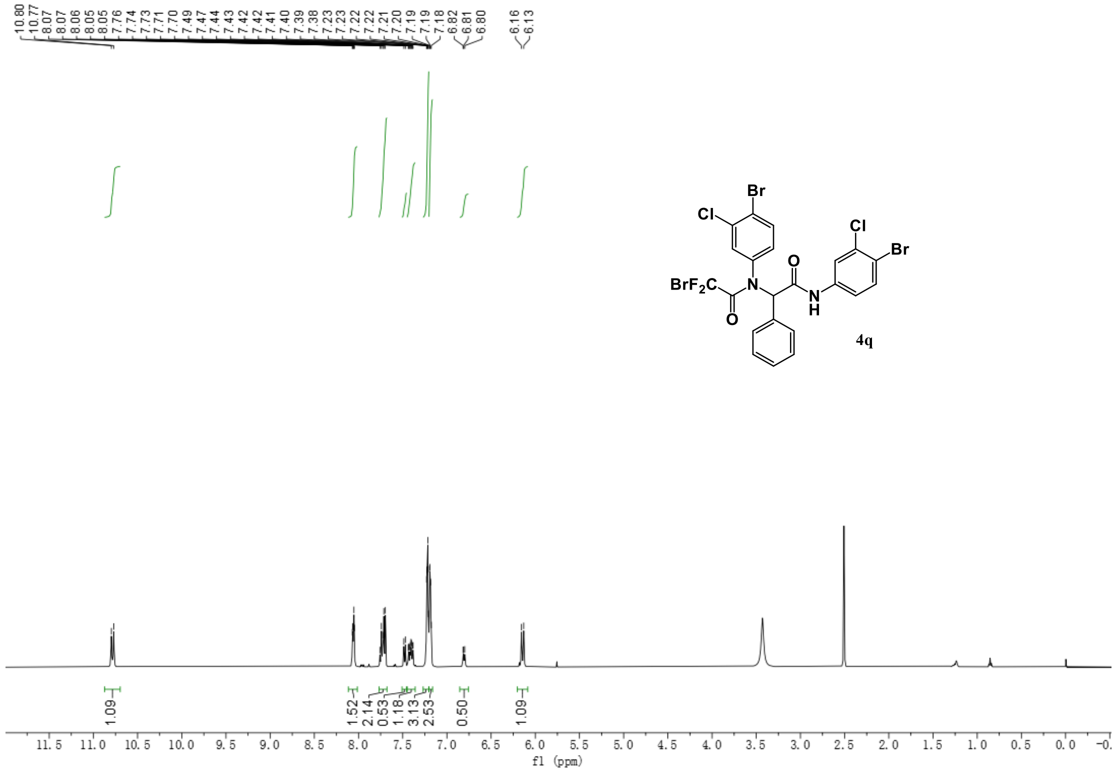

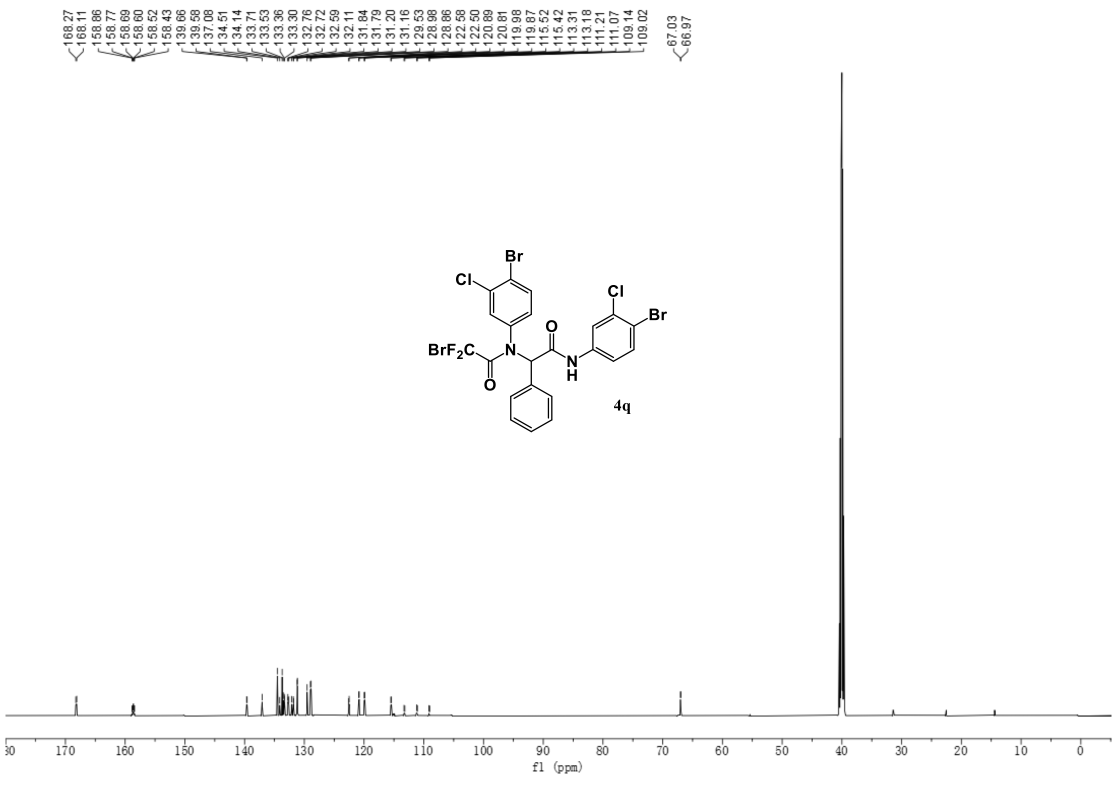

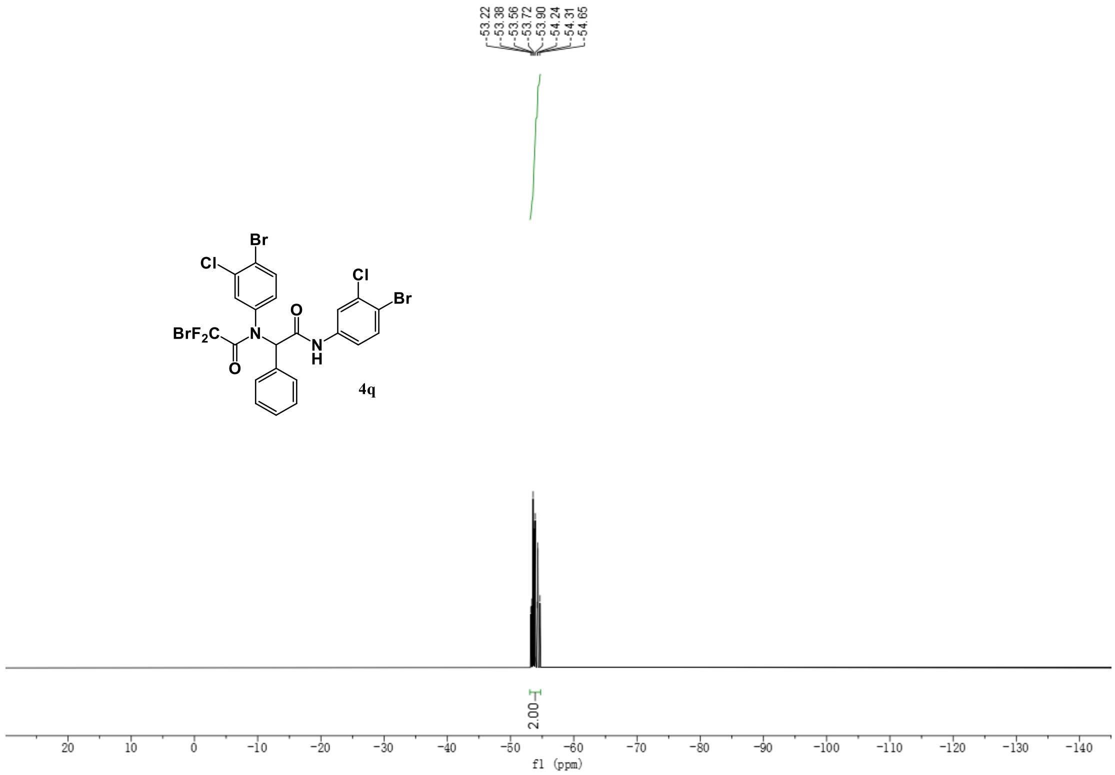

^{1}H NMR (500 MHz, DMSO), ^{13}C NMR (125 MHz, DMSO) and ^{19}F NMR (471 MHz, DMSO) spectra for 4r

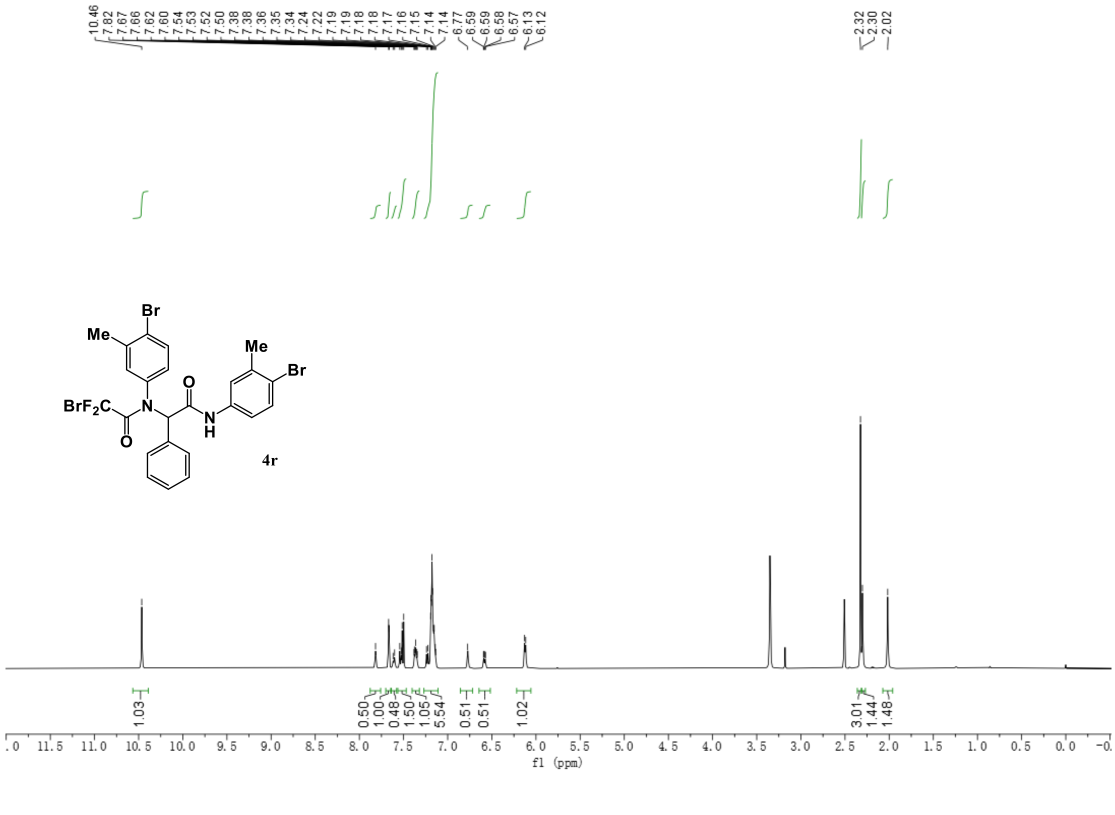

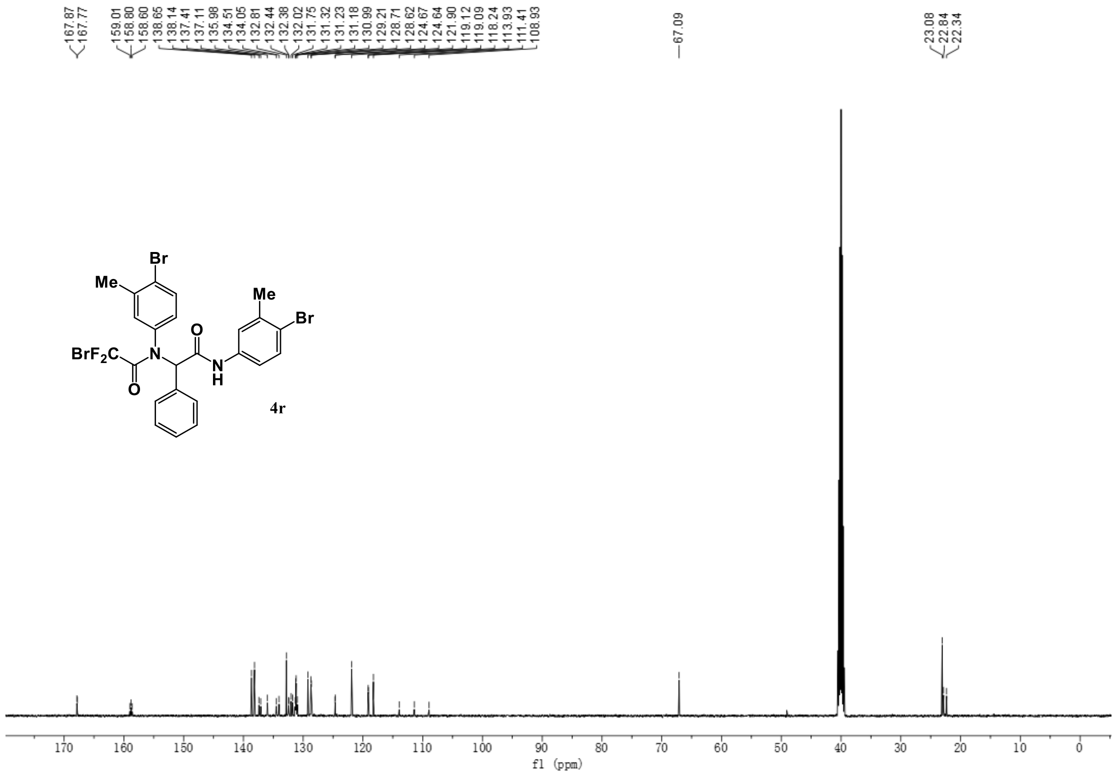
167.87
167.77
159.01
158.80
158.60
138.65
138.14
137.41
137.11
135.98
134.51
134.05
132.81
132.44
132.38
132.02
131.75
131.32
131.23
131.18
130.99
129.21
128.71
128.62
124.67
124.64
121.90
119.12
119.09
118.24
113.93
111.41
108.93
67.09
23.08
22.84
22.34
Br
Me
Me
Br
BrF2C
N
O
O
N
H
4r
f1 (ppm)

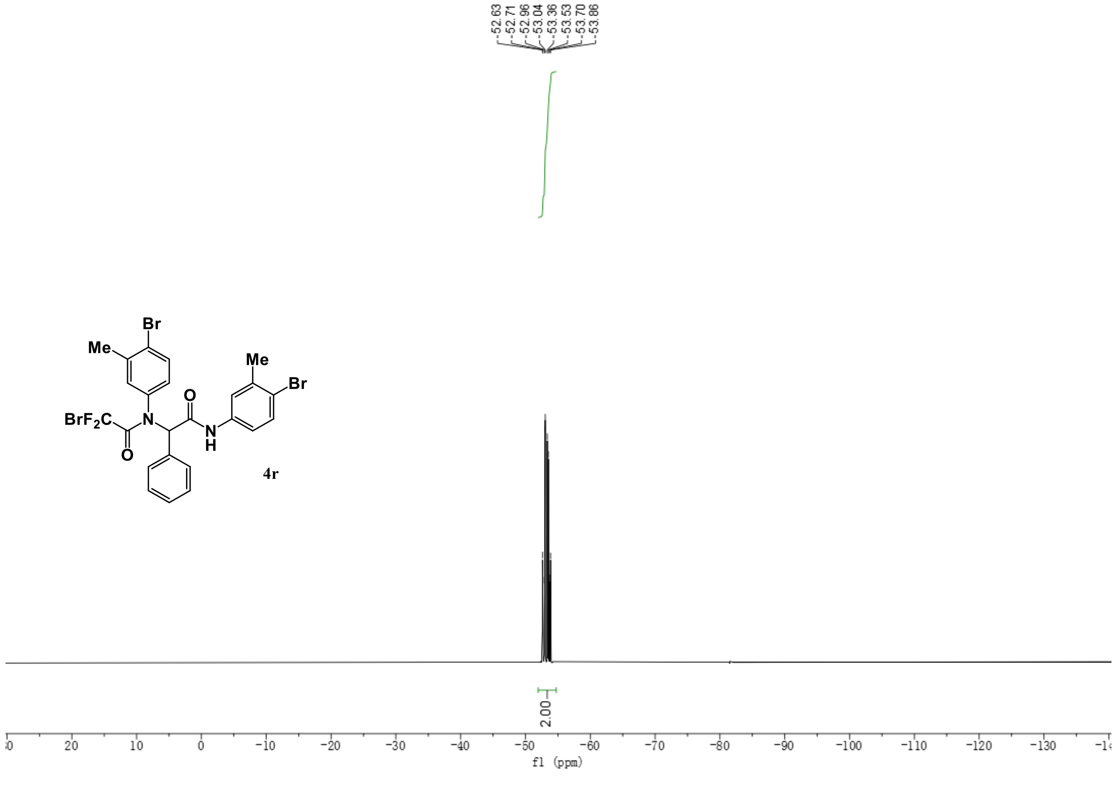
-52.63
-52.71
-52.96
-53.04
-53.36
-53.53
-53.70
-53.86
Br
Me
Me
Br
BrF2C
N
O
O
N
H
4r
2.00
f1 (ppm)

^{1}H NMR (500 MHz, DMSO), ^{13}C NMR (125 MHz, DMSO) and ^{19}F NMR (471 MHz, DMSO) spectra for 4s

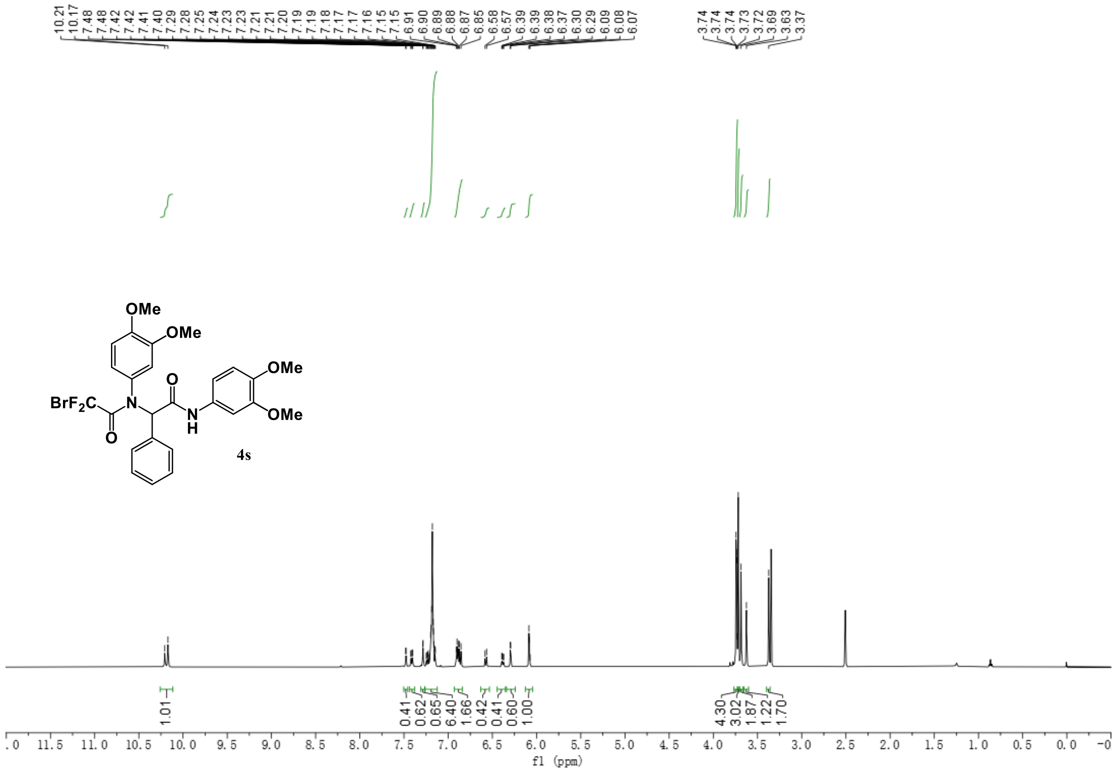

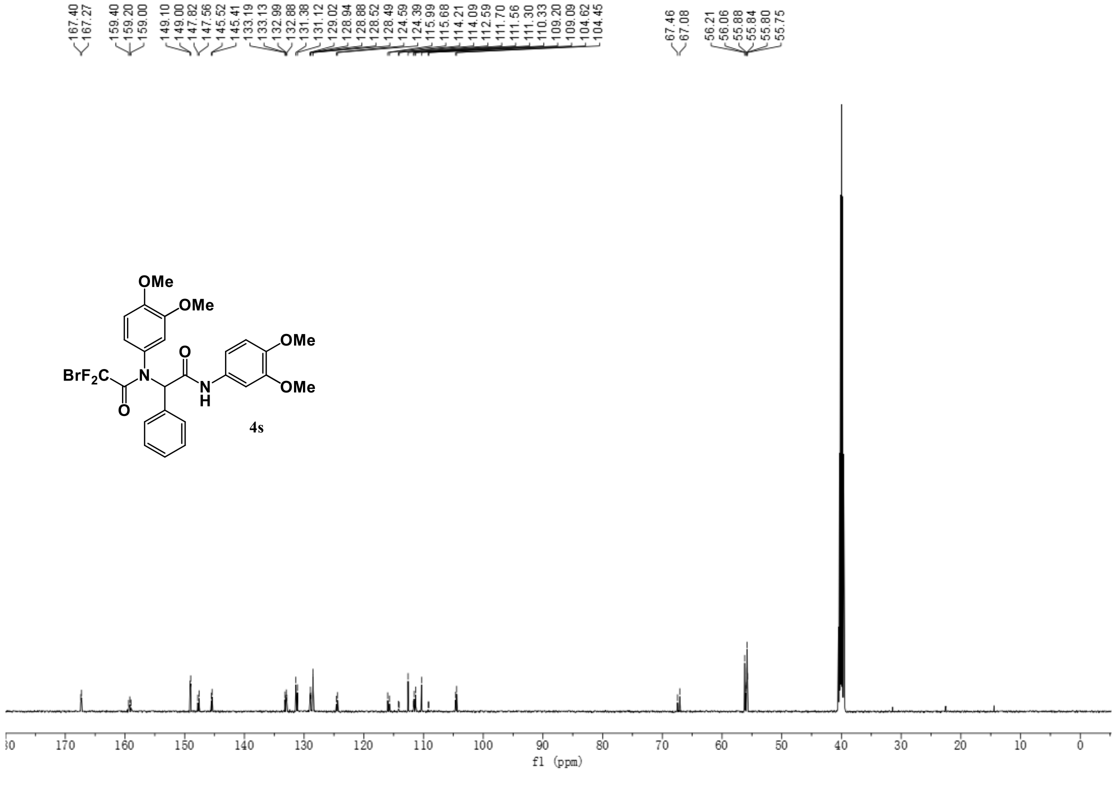

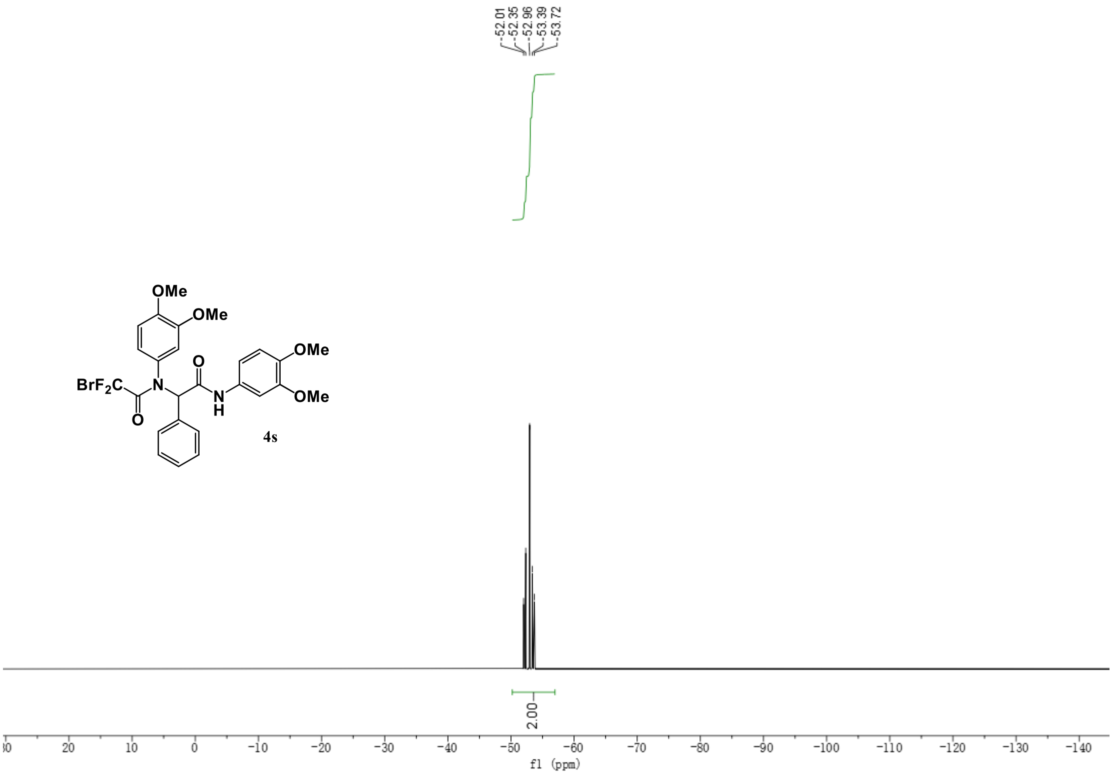

^{1}H NMR (500 MHz, CD$_3$OD), ^{13}C NMR (125 MHz, DMSO) and ^{19}F NMR (471 MHz, DMSO) spectra for 4t

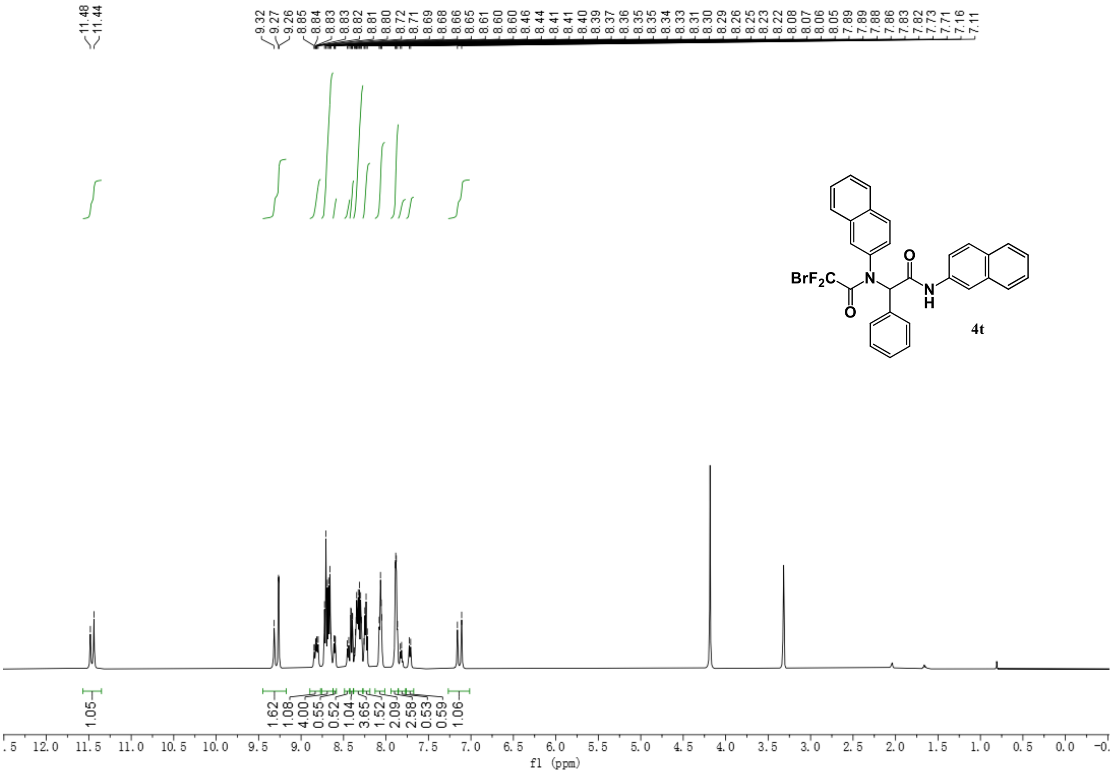

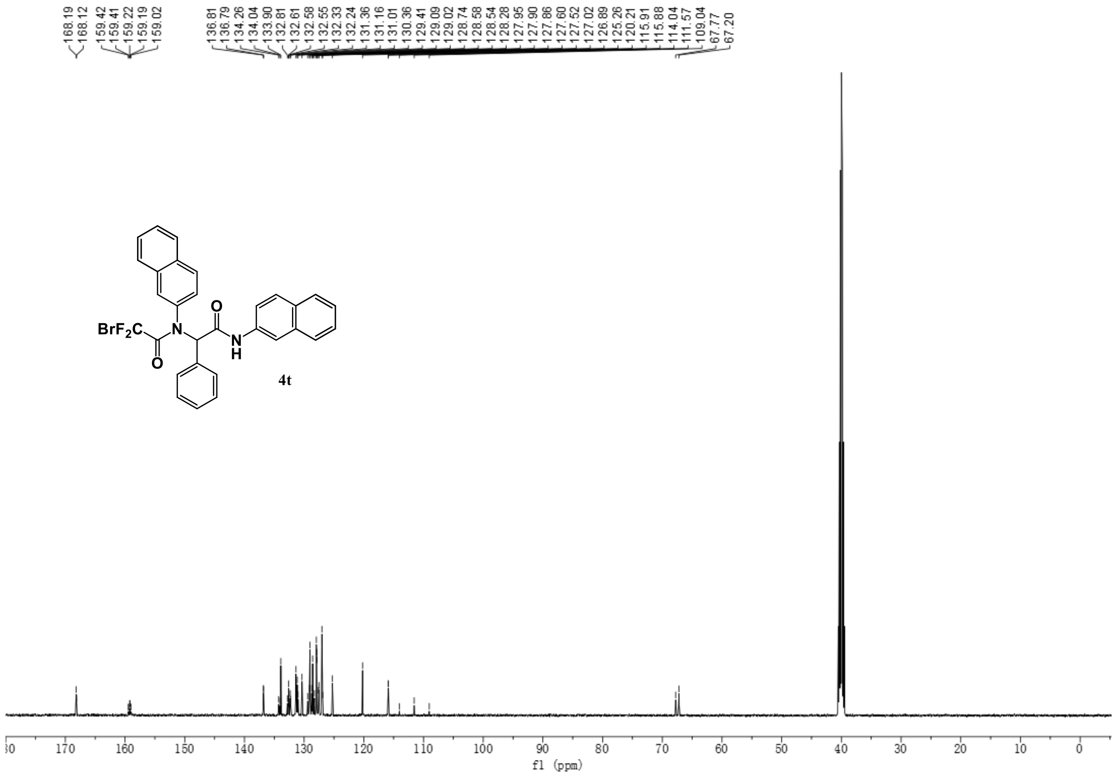
168.19
168.12
159.42
159.41
159.22
159.19
159.02
136.81
136.79
134.26
134.04
133.90
132.81
132.61
132.58
132.55
132.33
132.24
131.36
131.16
131.01
130.36
129.41
129.09
129.02
128.74
128.58
128.54
128.28
127.95
127.90
127.86
127.60
127.52
127.02
126.89
125.26
120.21
115.91
115.88
114.04
111.57
109.04
67.77
67.20
BrF2C
O
N
H
4t
f1 (ppm)

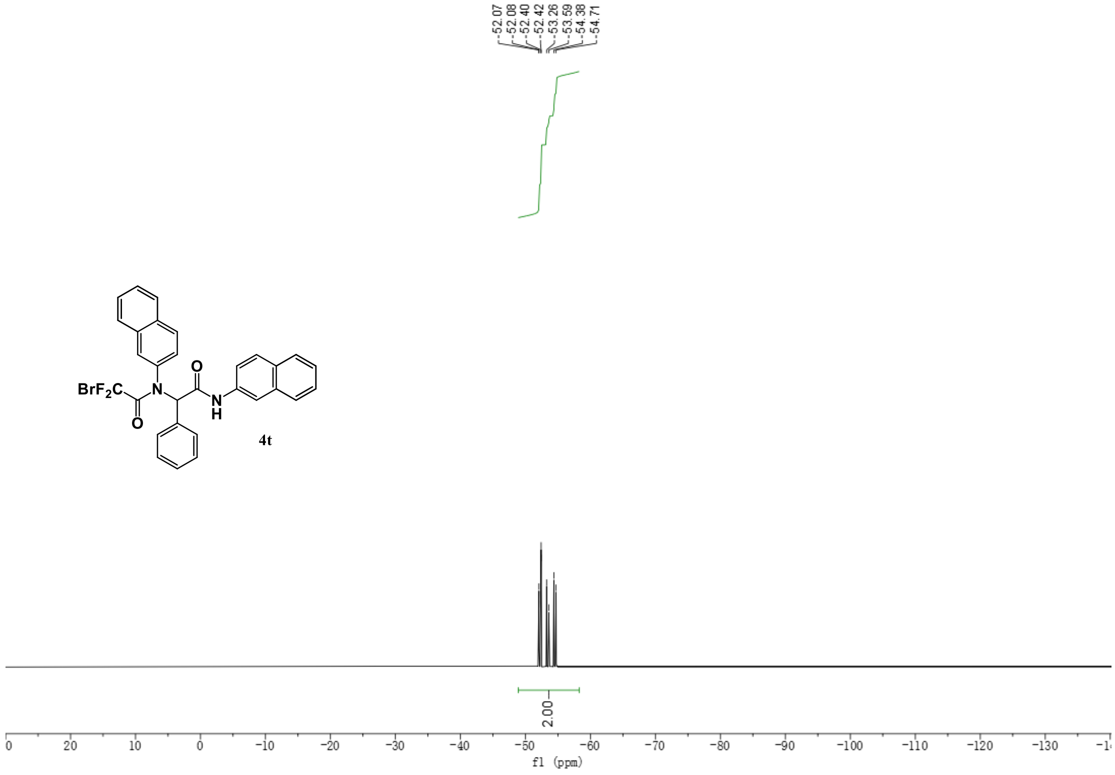
-52.07
-52.08
-52.40
-52.42
-53.26
-53.59
-54.38
-54.71
BrF2C
O
N
H
4t
2.00
f1 (ppm)

^{1}H NMR (500 MHz, DMSO), ^{13}C NMR (125 MHz, DMSO) and ^{19}F NMR (471 MHz, DMSO) spectra for 4u

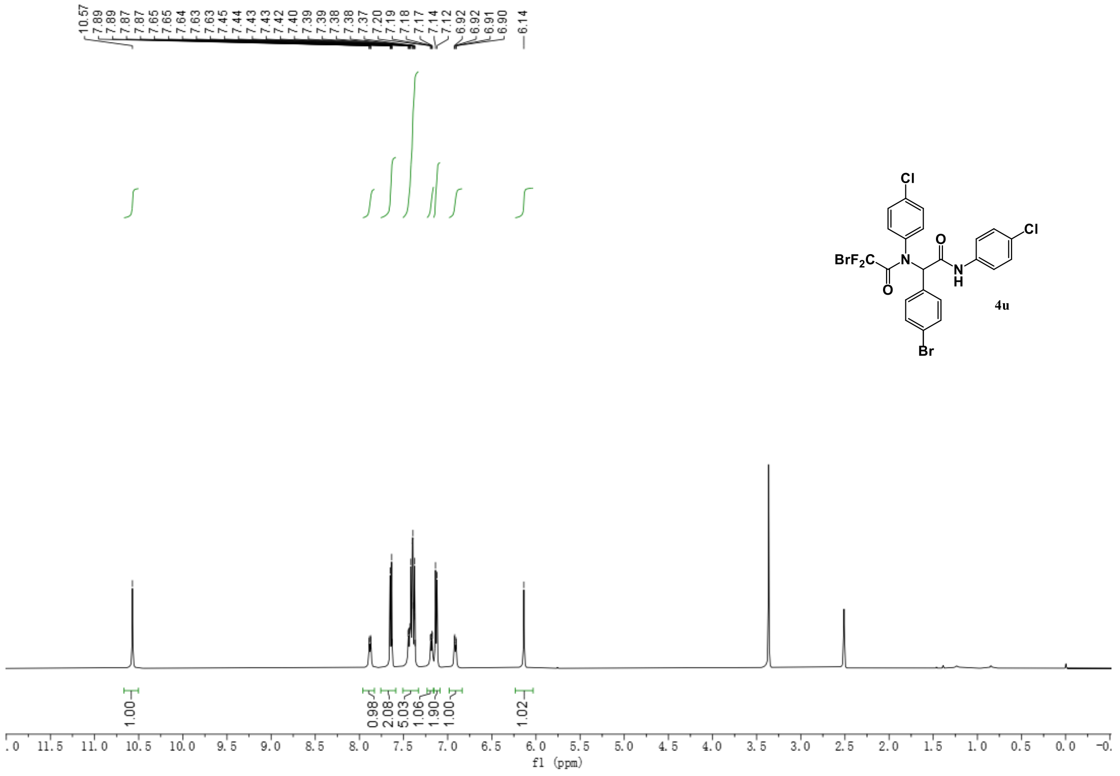

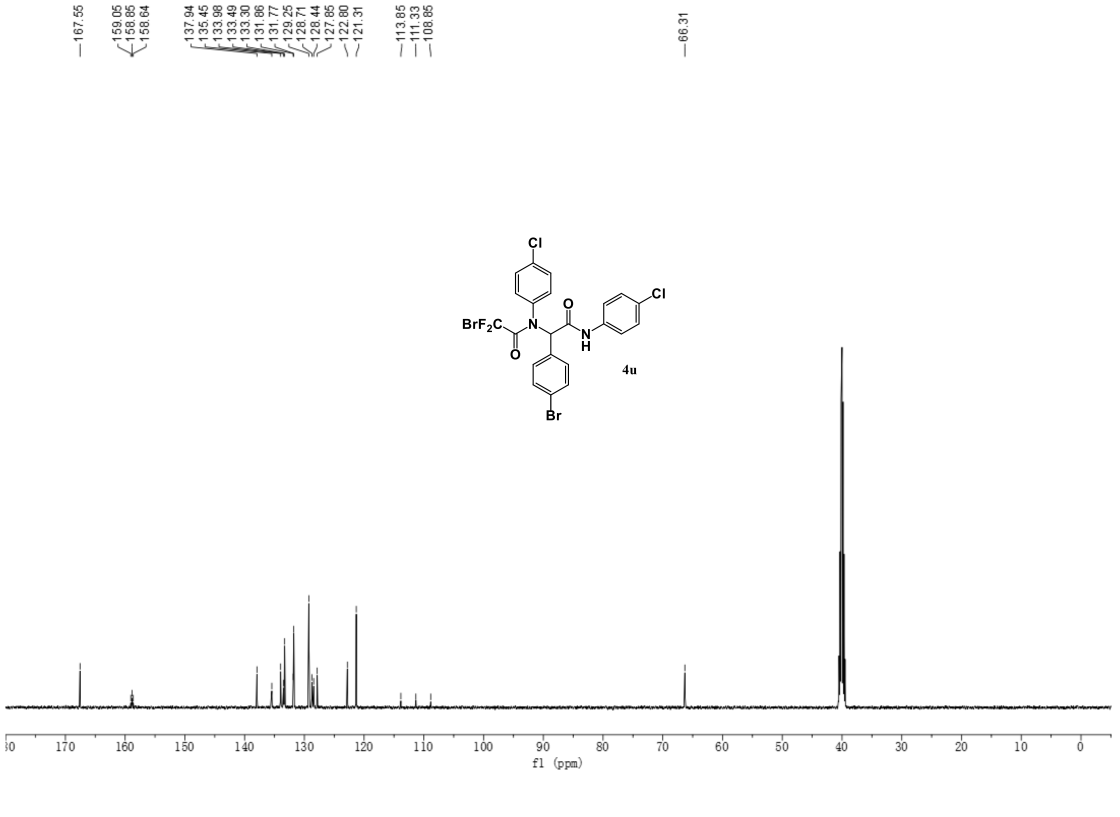

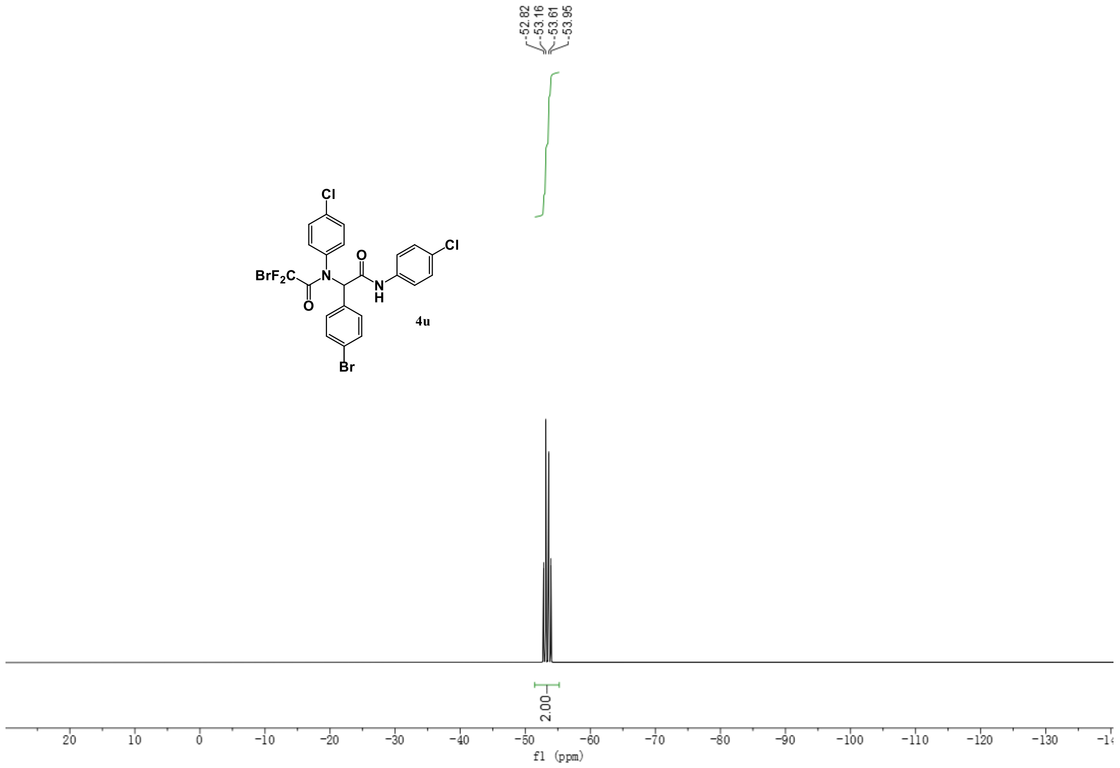

^{1}H NMR (500 MHz, DMSO), ^{13}C NMR (150 MHz, DMSO) and ^{19}F NMR (471 MHz, DMSO) spectra for 4v

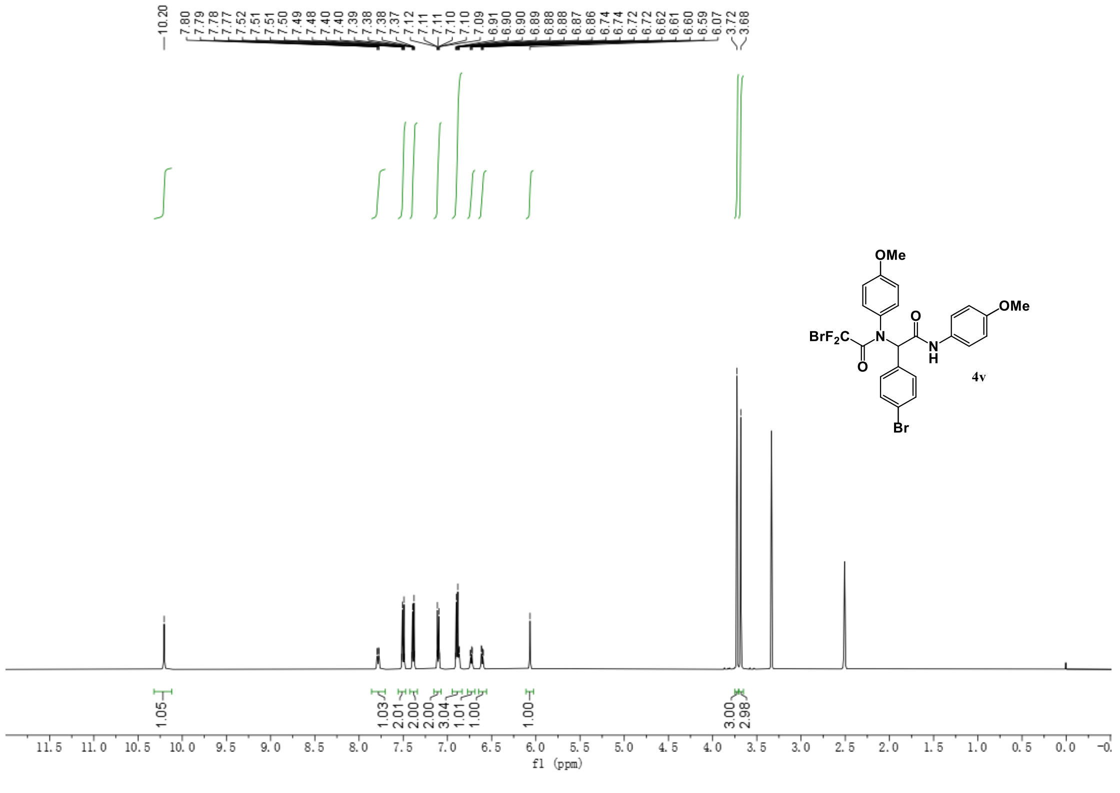

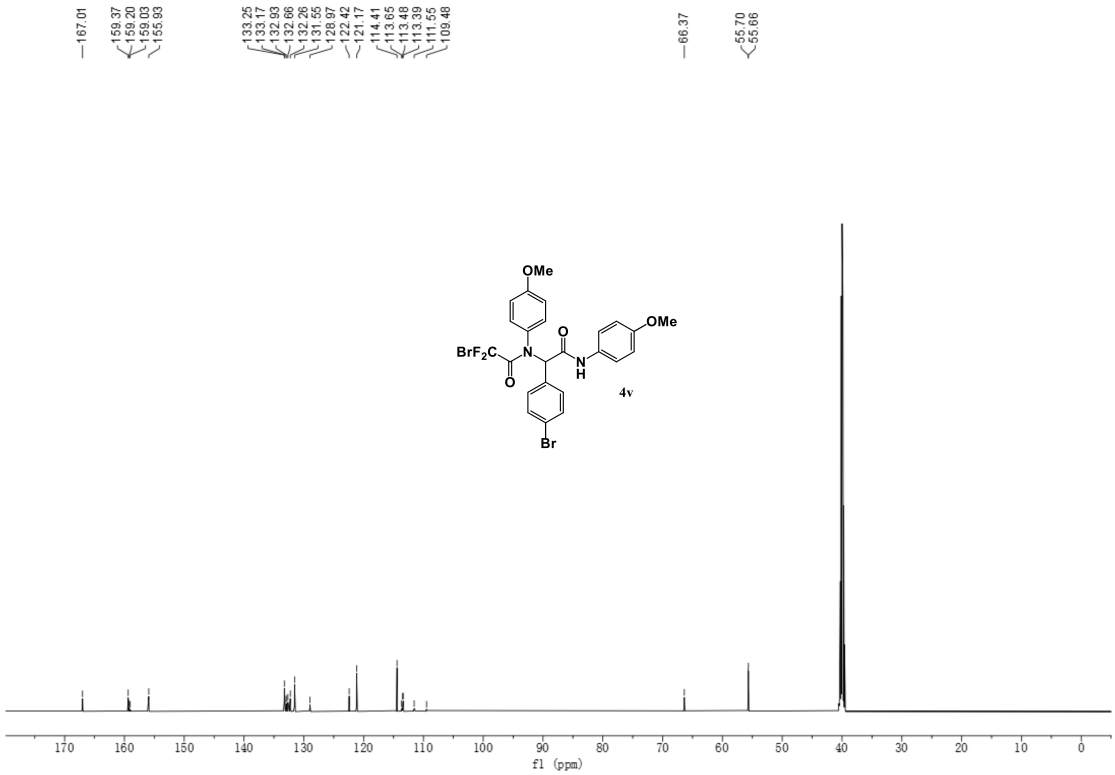
167.01
159.37
159.20
159.03
155.93
133.25
133.17
132.93
132.66
132.26
131.55
128.97
122.42
121.17
114.41
113.65
113.48
113.39
111.55
109.48
66.37
55.70
55.66
OMe
BrF2C
N
O
N
H
OMe
Br
4v
f1 (ppm)

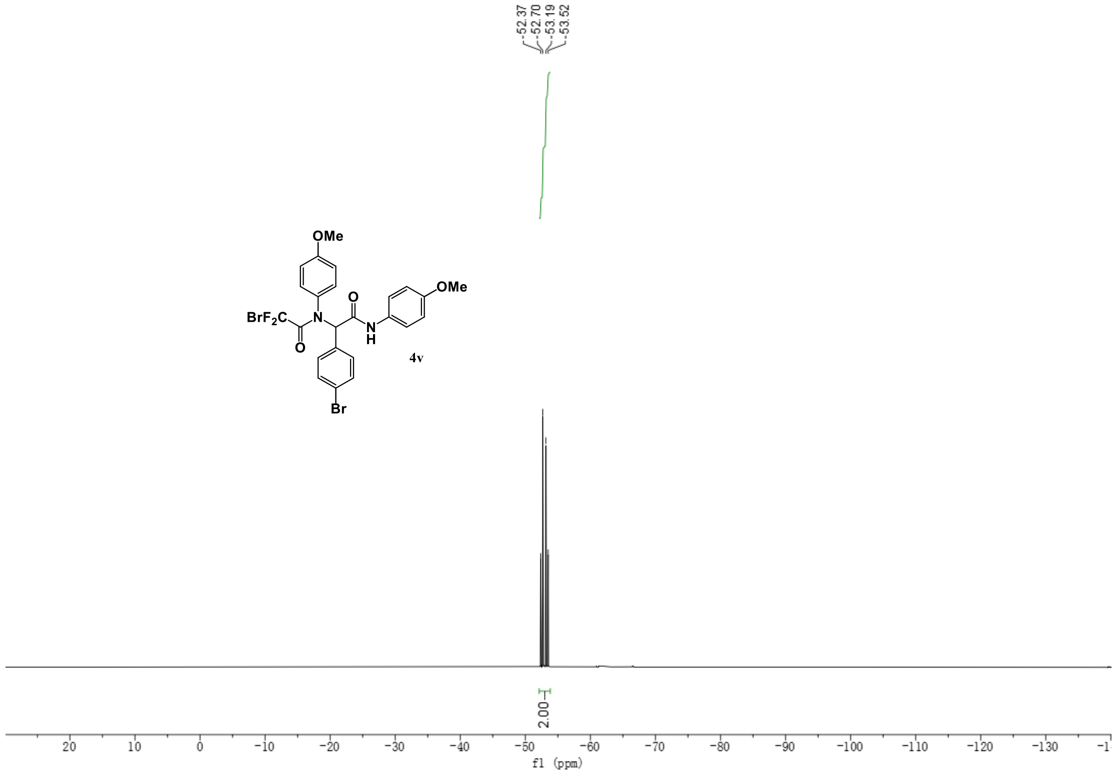
-52.37
-52.70
-53.19
-53.52
OMe
BrF2C
N
O
N
H
OMe
Br
4v
2.00
f1 (ppm)

^{1}H NMR (500 MHz, DMSO), ^{13}C NMR (125 MHz, DMSO) and ^{19}F NMR (471 MHz, DMSO) spectra for 4w

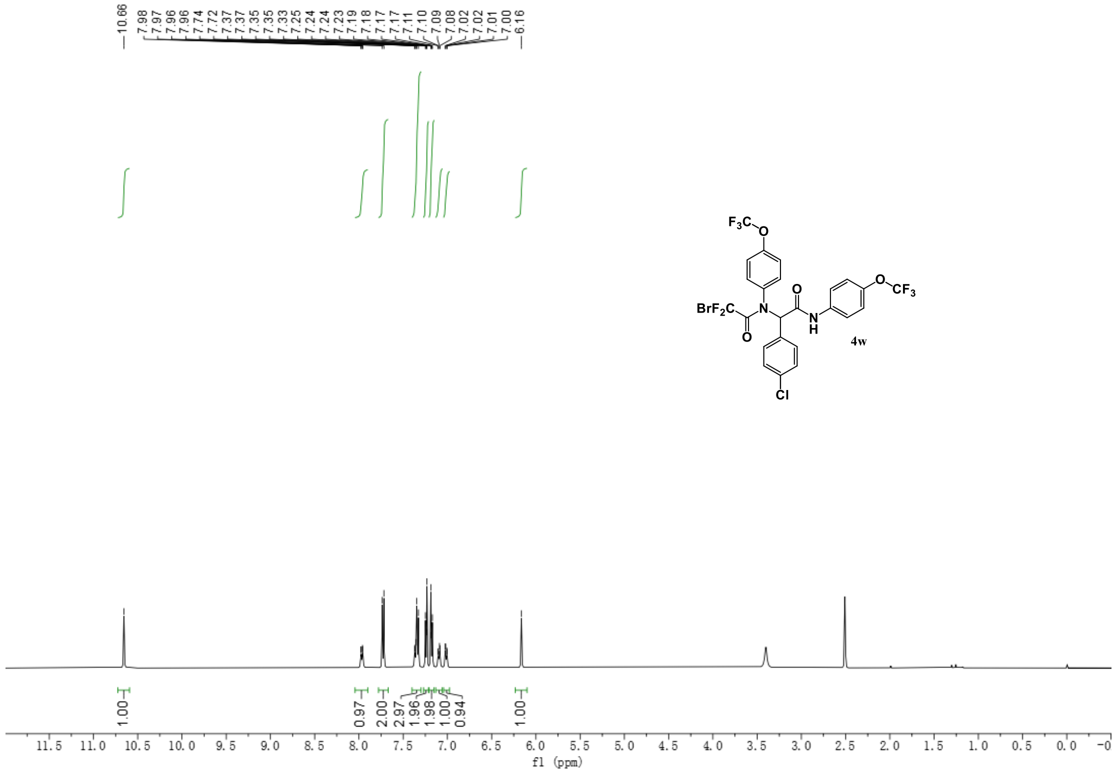

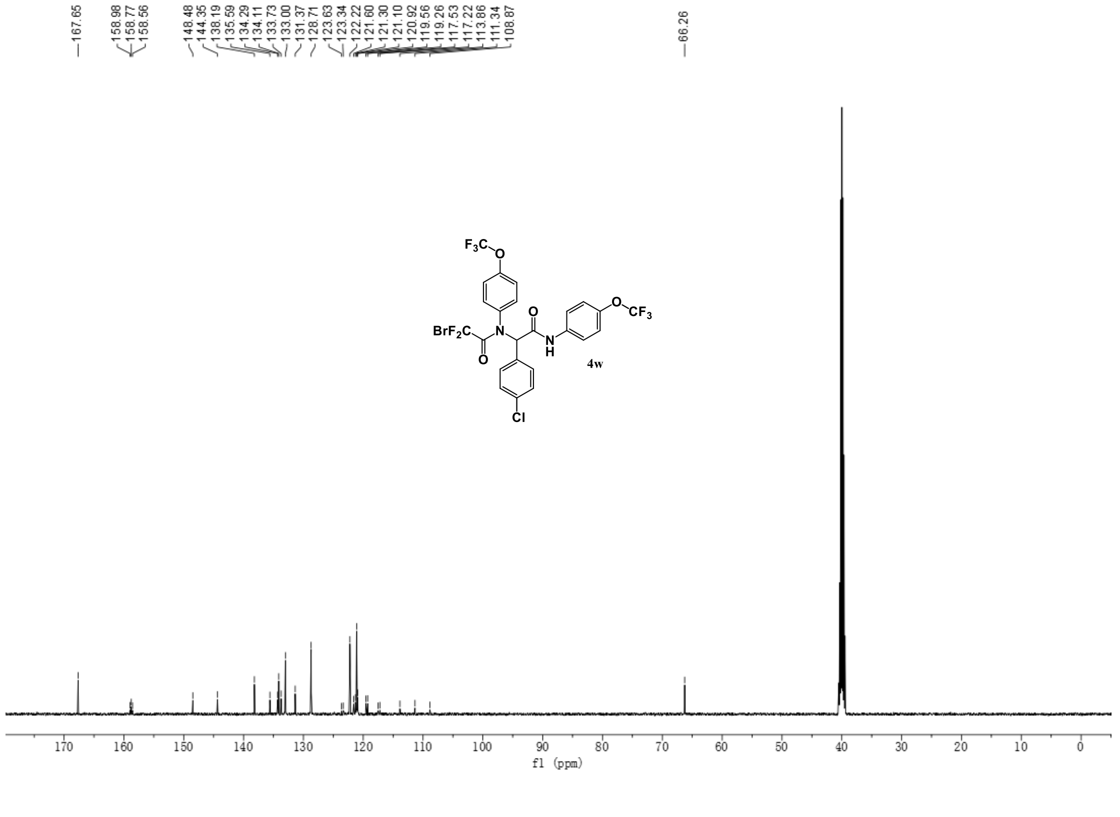

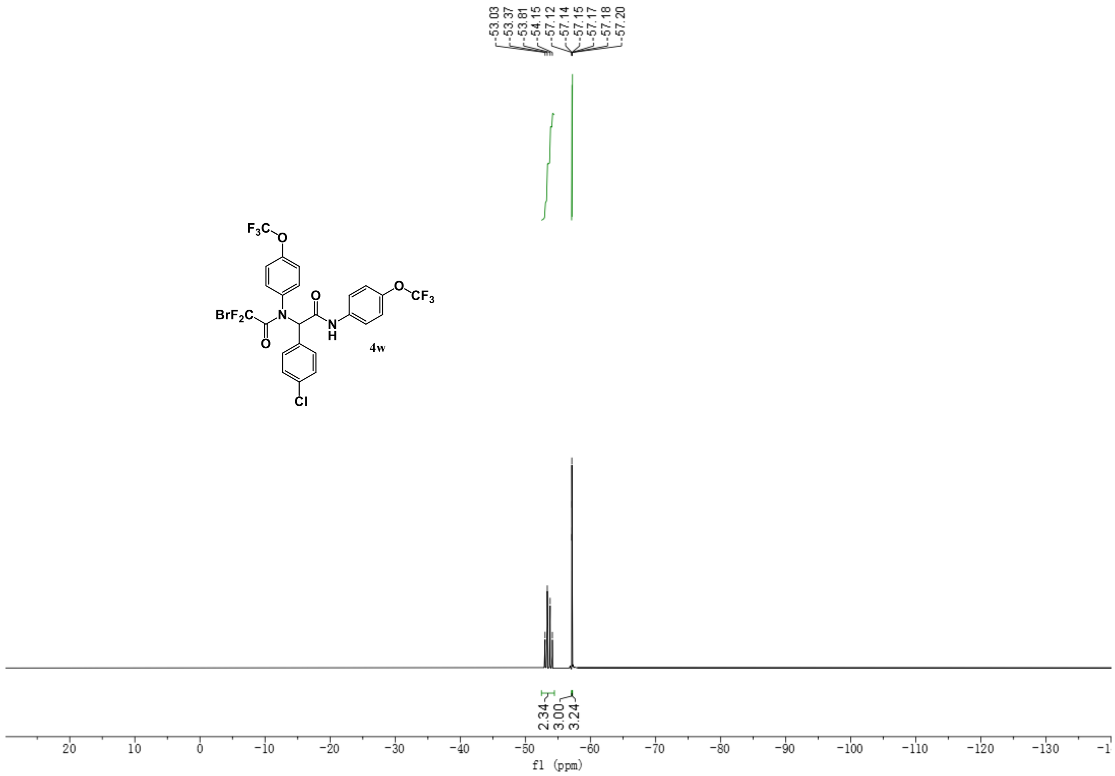

^{1}H NMR (600 MHz, DMSO), ^{13}C NMR (150 MHz, DMSO) and ^{19}F NMR (471 MHz, DMSO) spectra for 4x

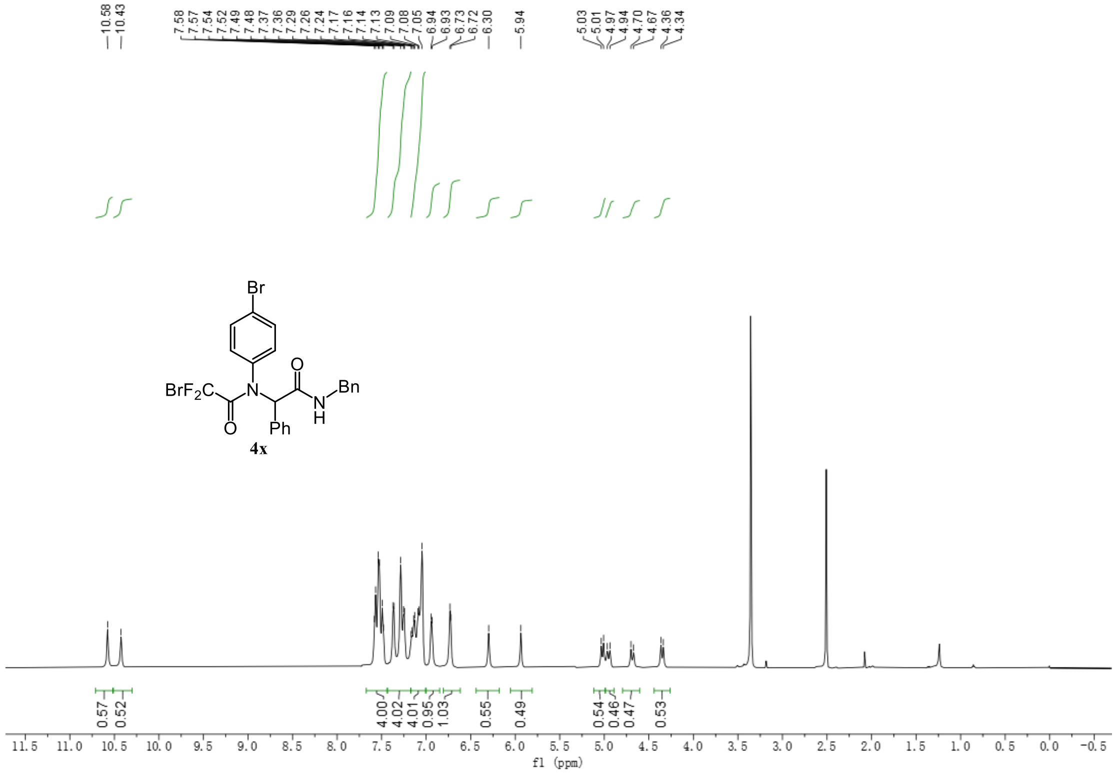

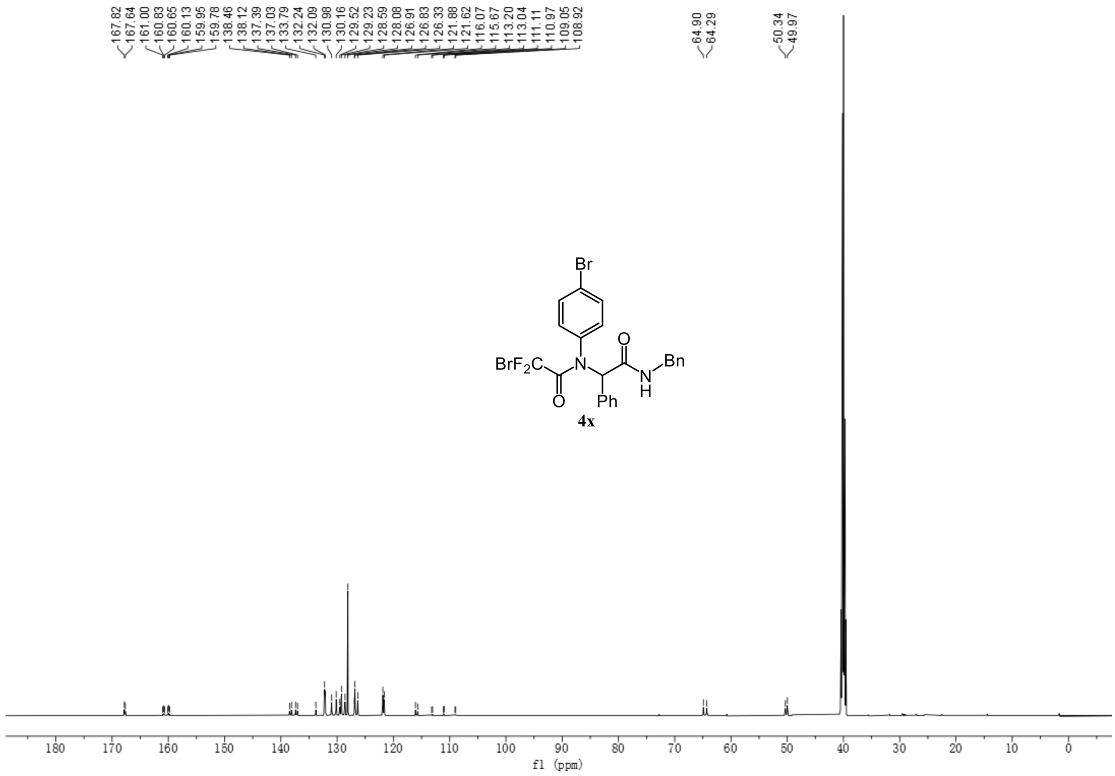
167.82
167.64
161.00
160.83
160.65
160.13
159.95
159.78
138.46
138.12
137.39
137.03
133.79
132.24
132.09
130.98
130.16
129.52
129.23
128.59
128.08
126.91
126.83
126.33
121.88
121.62
116.07
115.67
113.20
113.04
111.11
110.97
109.05
108.92
64.90
64.29
50.34
49.97
Br
BrF2C
N
O
O
N
H
Bn
Ph
4x
f1 (ppm)

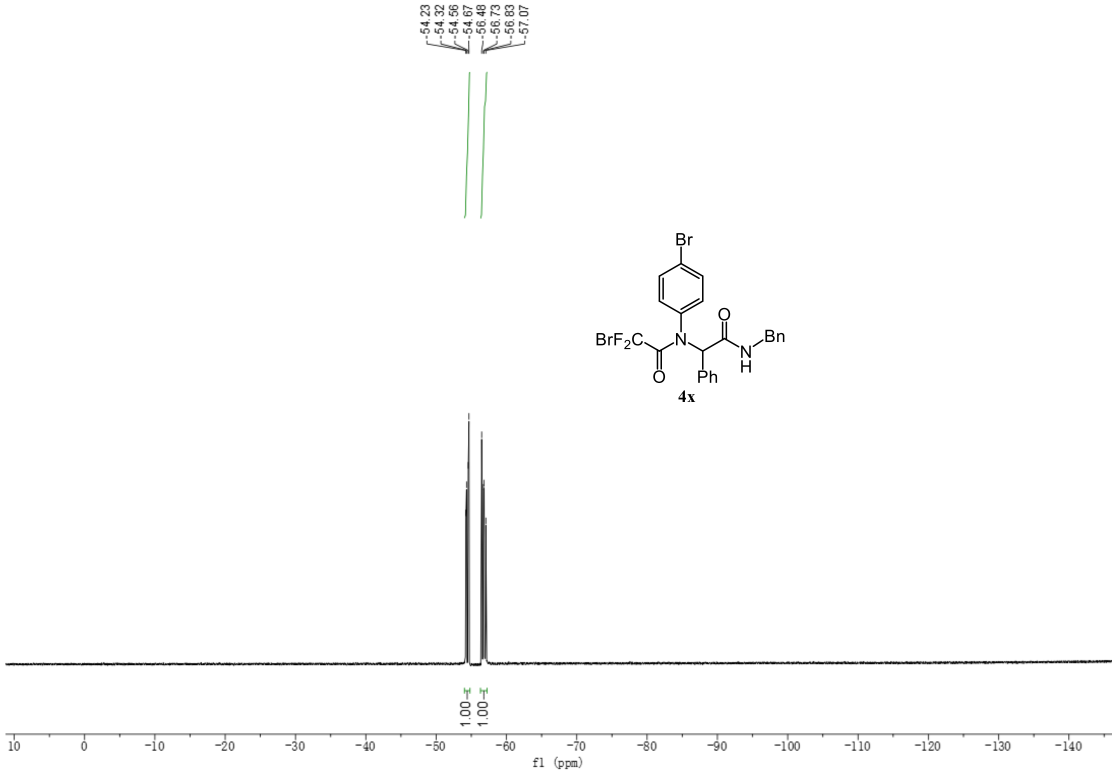
-54.23
-54.32
-54.56
-54.67
-56.48
-56.73
-56.83
-57.07
Br
BrF2C
N
O
O
N
H
Bn
Ph
4x
1.00
1.00
f1 (ppm)

^{1}H NMR (600 MHz, DMSO), ^{13}C NMR (150 MHz, DMSO) and ^{19}F NMR (471 MHz, DMSO) spectra for 4y

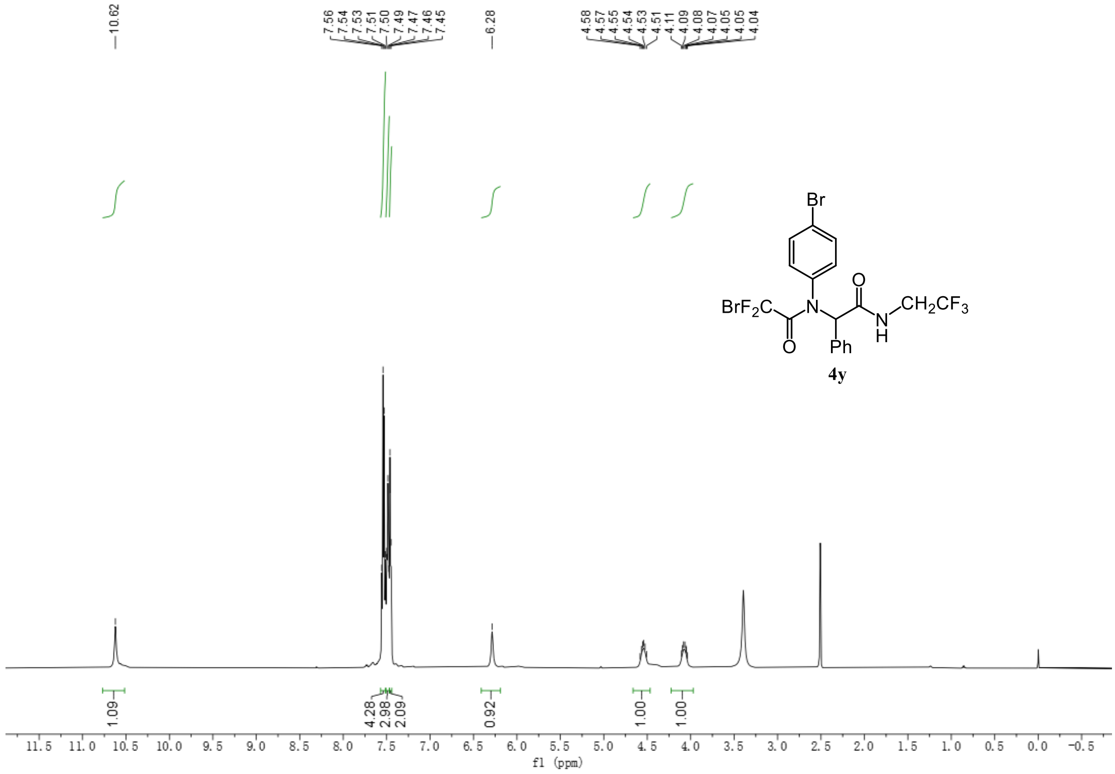

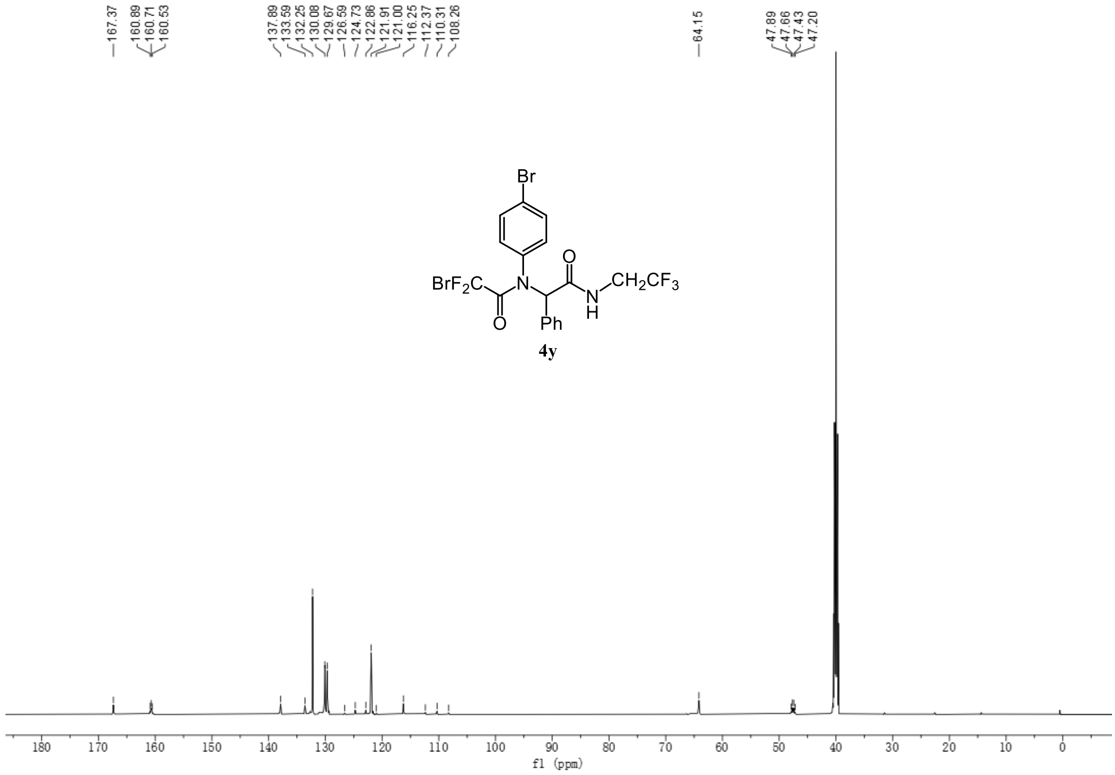

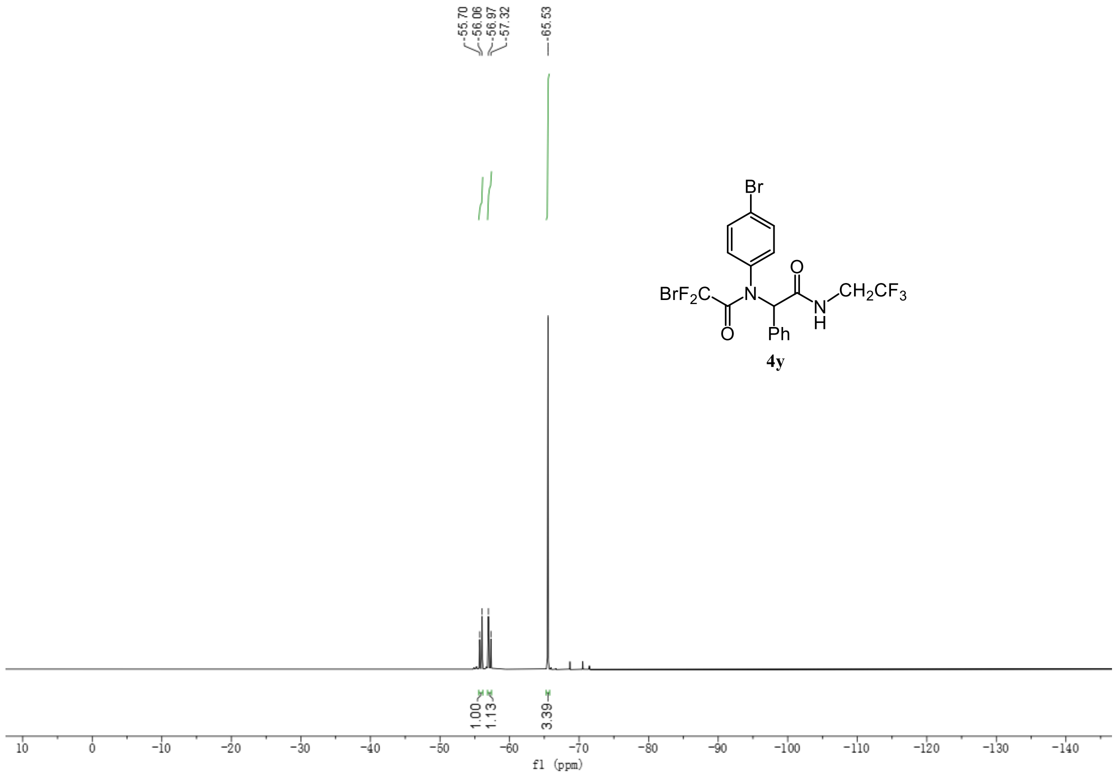

^{1}H NMR (600 MHz, CDCl3), ^{13}C NMR (150 MHz, CDCl3) and ^{19}F NMR (471 MHz, CDCl3) spectra for 4z

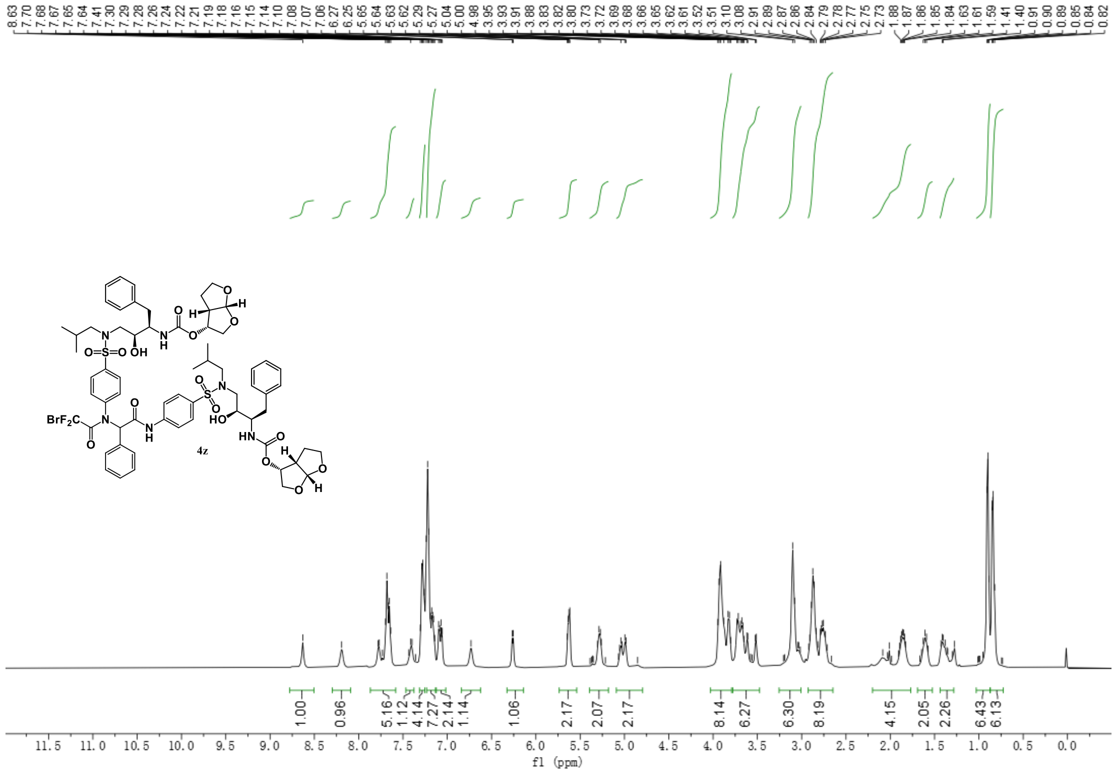

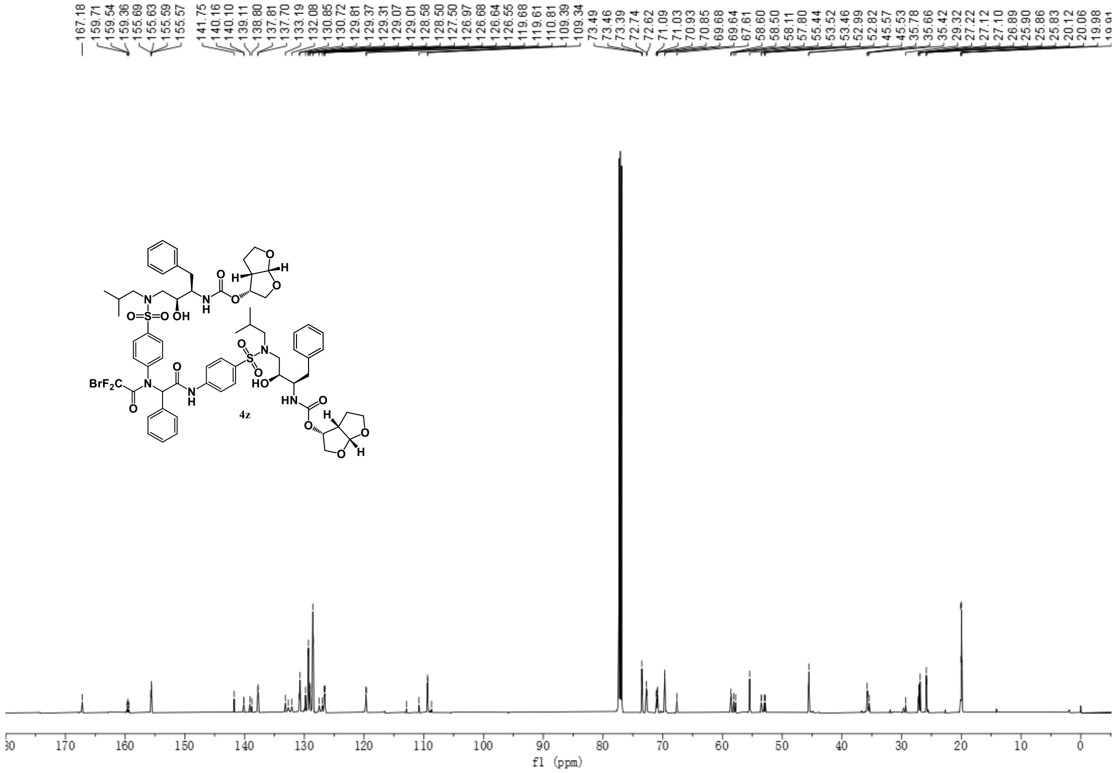
167.18
159.71
159.54
159.36
155.69
155.63
155.59
155.57
141.75
140.16
140.10
139.11
138.80
137.81
137.70
133.19
132.08
130.85
130.72
129.81
129.37
129.31
129.07
129.01
128.58
128.50
127.50
126.97
126.68
126.64
126.55
119.68
119.61
110.81
109.39
109.34
73.49
73.46
73.39
72.74
72.62
71.09
71.03
70.93
70.85
69.68
69.64
67.61
58.60
58.50
58.11
57.80
55.44
53.52
53.46
52.99
52.82
45.57
45.53
35.78
35.66
35.42
29.32
27.22
27.12
27.10
26.89
25.90
25.86
25.83
20.12
20.06
19.98
BrF2C
4z
f1 (ppm)

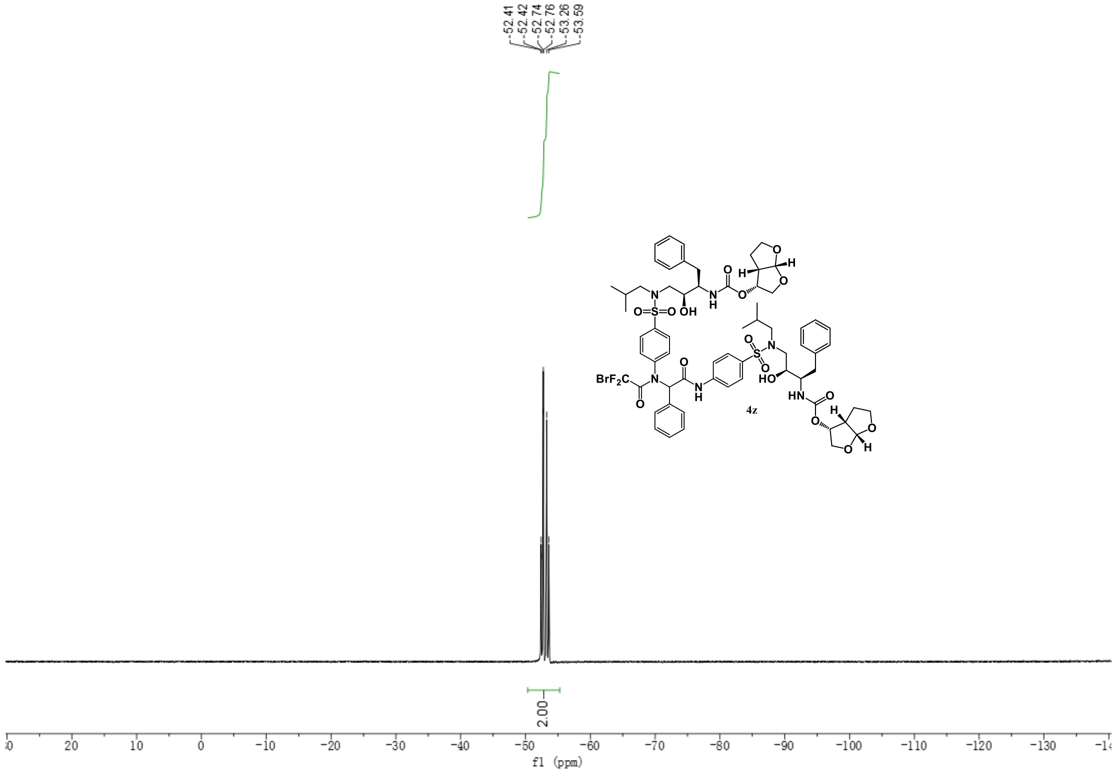
52.41
52.42
52.74
52.76
53.26
53.59
BrF2C
4z
2.00
f1 (ppm)

^{1}H NMR (500 MHz, DMSO), ^{13}C NMR (150 MHz, DMSO) and ^{19}F NMR (471 MHz, DMSO) spectra for 4aa

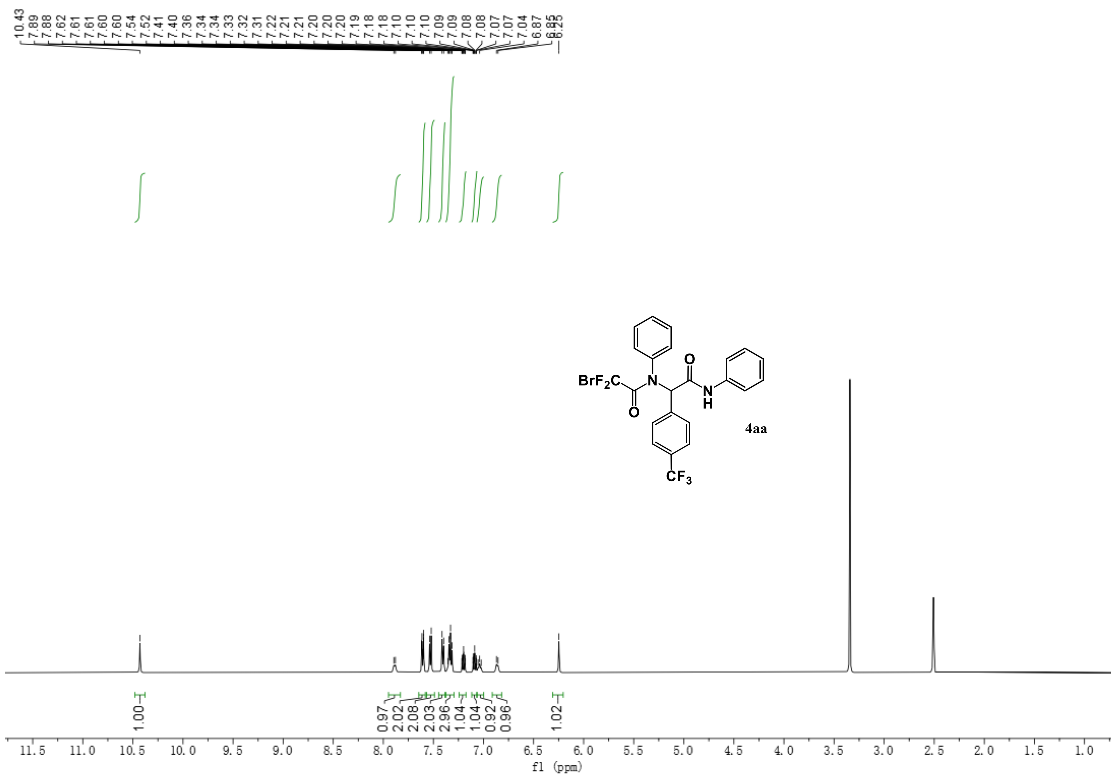

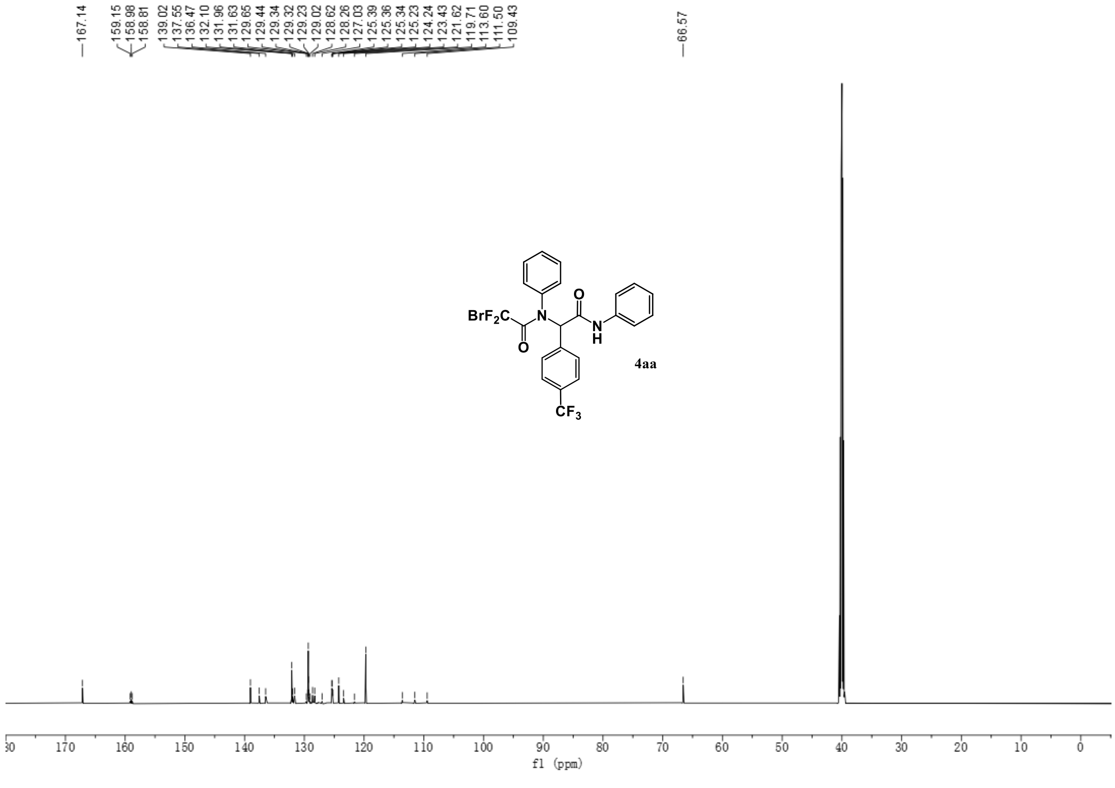

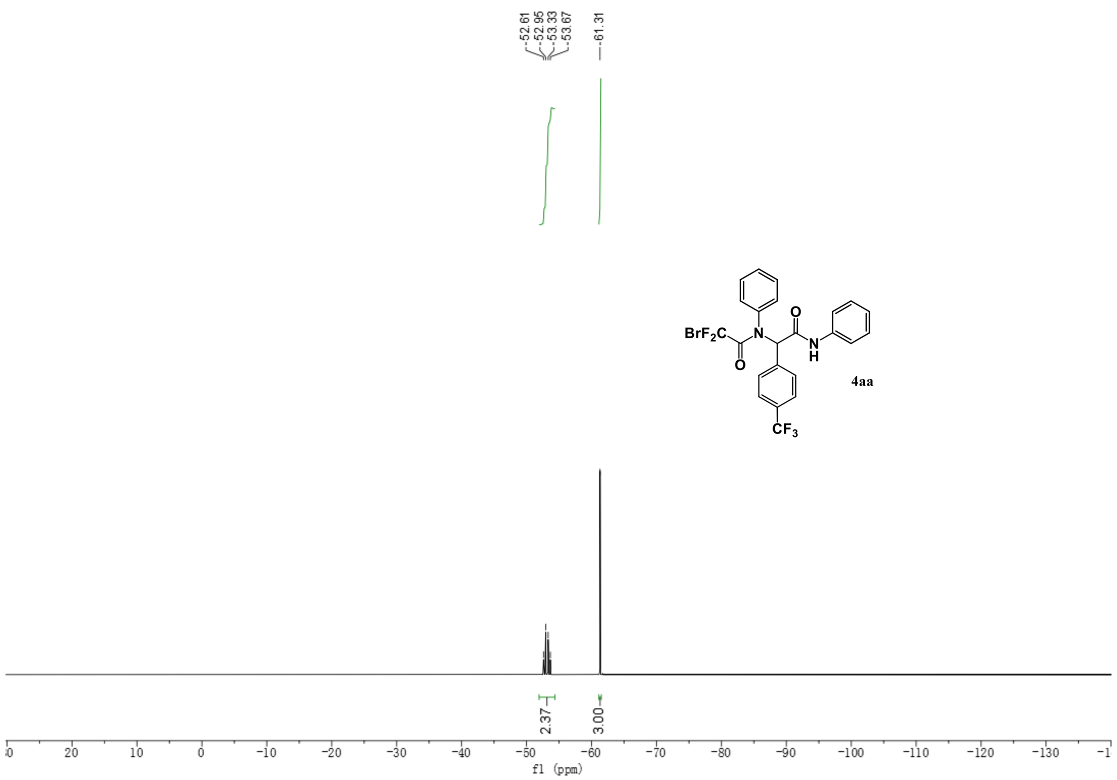

^{1}H NMR (500 MHz, DMSO), ^{13}C NMR (150 MHz, DMSO) and ^{19}F NMR (471 MHz, DMSO) spectra for 4ab

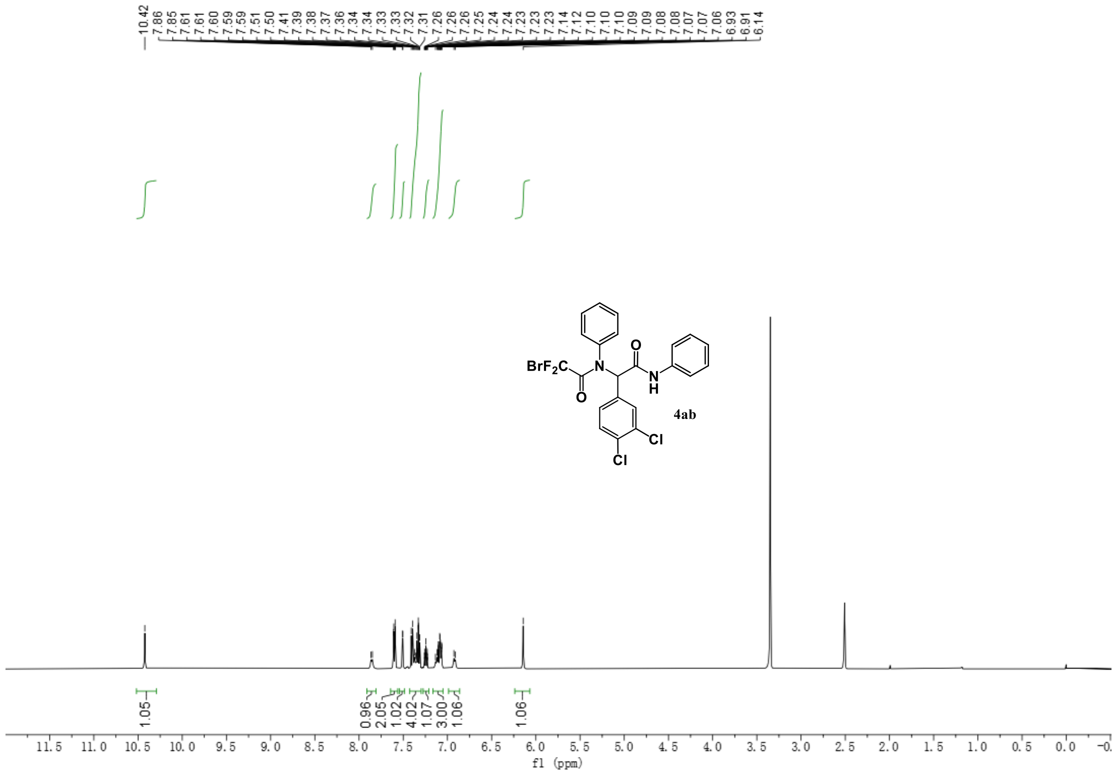

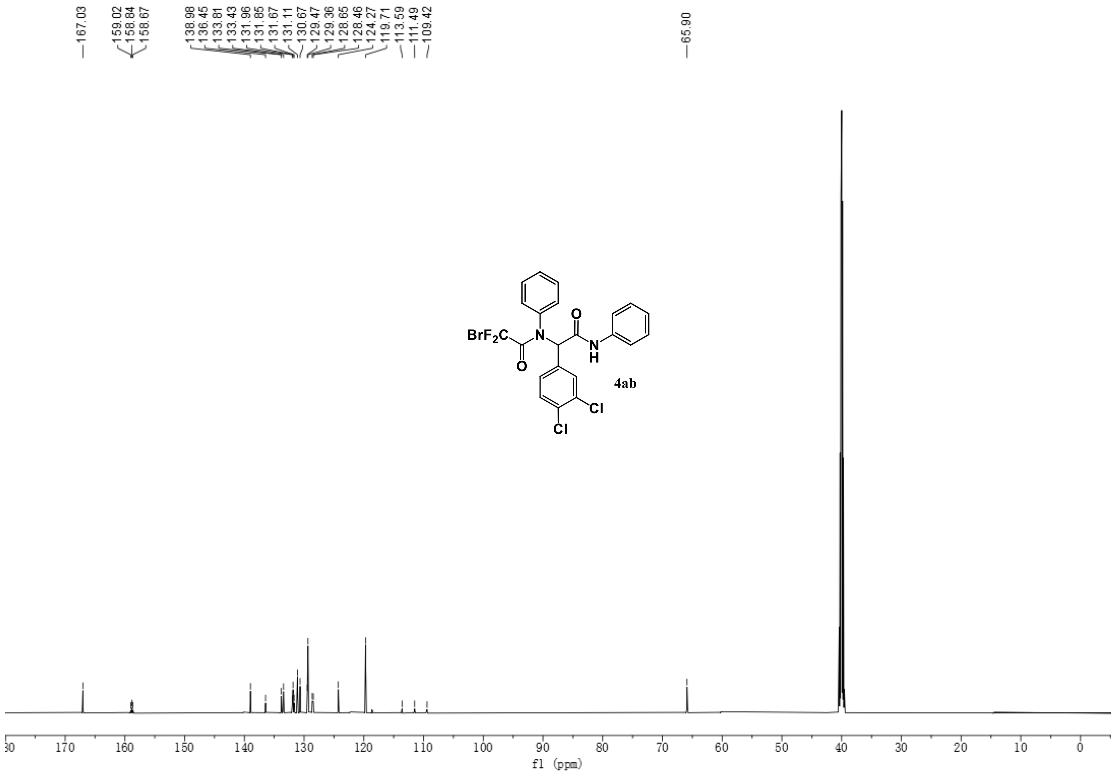
167.03
159.02
158.84
158.67
138.98
136.45
133.81
133.43
131.96
131.85
131.67
131.11
130.67
129.47
129.36
128.65
128.46
124.27
119.71
113.59
111.49
109.42
65.90
4ab
f1 (ppm)

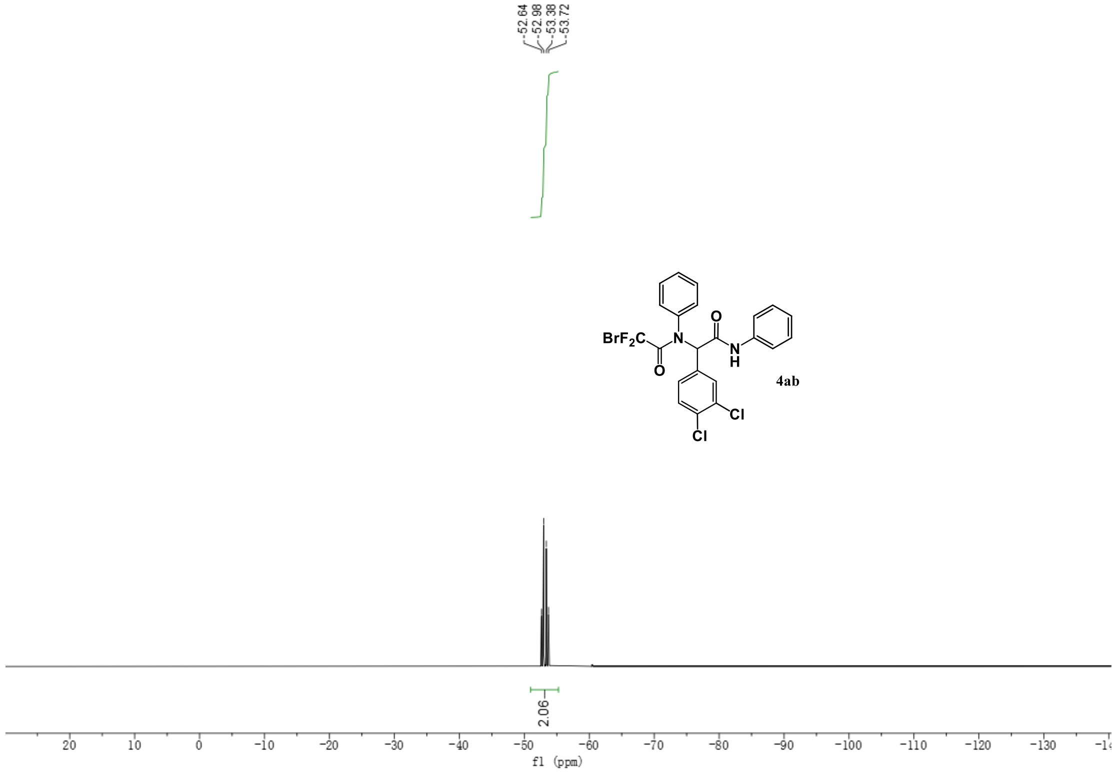
-52.64
-52.98
-53.38
-53.72
4ab
2.06
f1 (ppm)

^{1}H NMR (500 MHz, DMSO), ^{13}C NMR (125 MHz, DMSO) and ^{19}F NMR (471 MHz, DMSO) spectra for 4ac

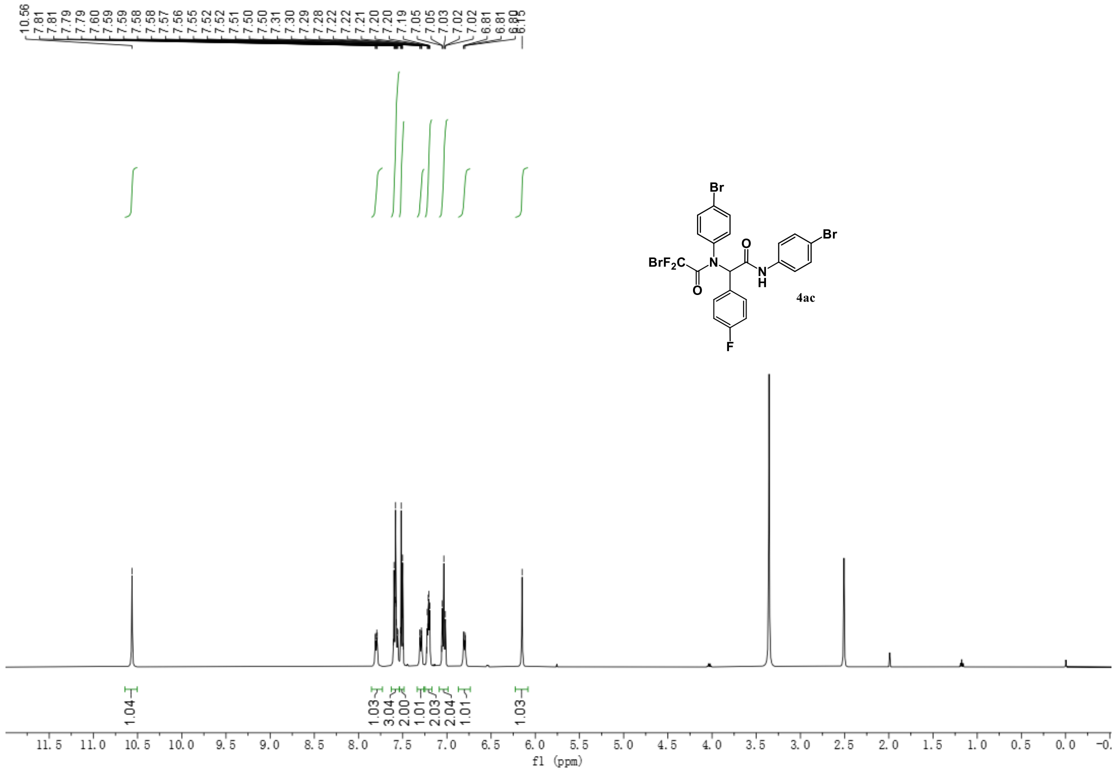

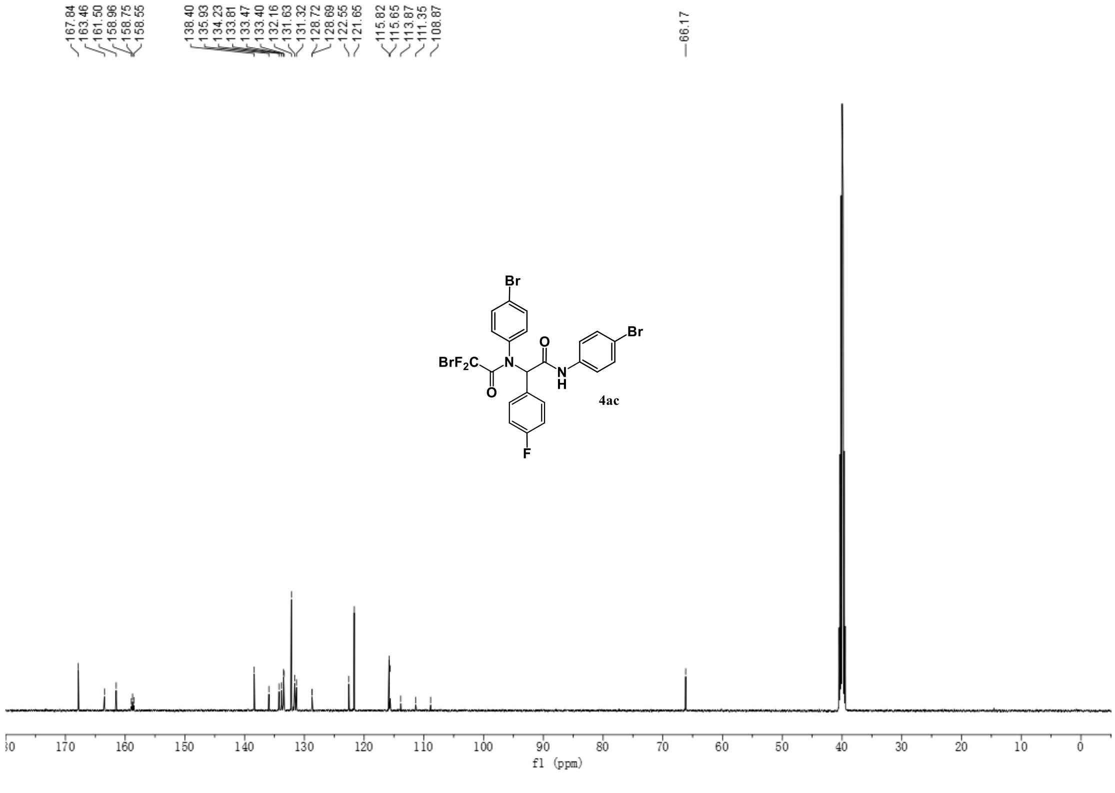

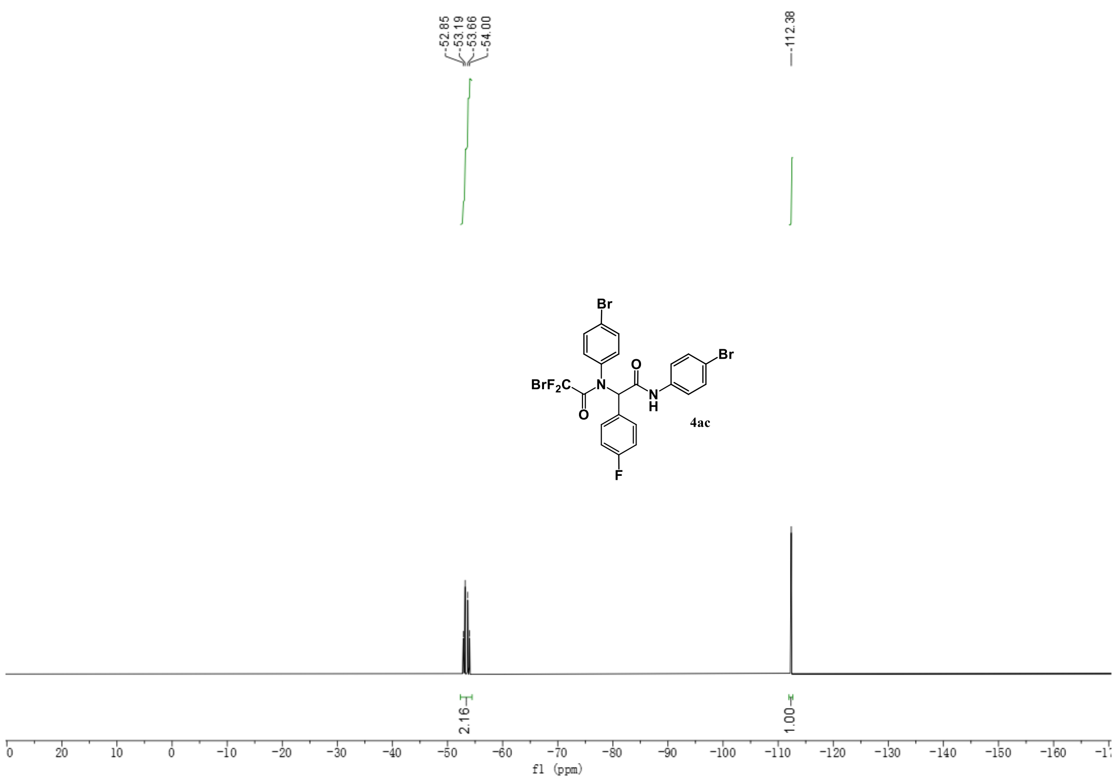

^{1}H NMR (500 MHz, DMSO), ^{13}C NMR (150 MHz, DMSO) and ^{19}F NMR (471 MHz, DMSO) spectra for 4ad

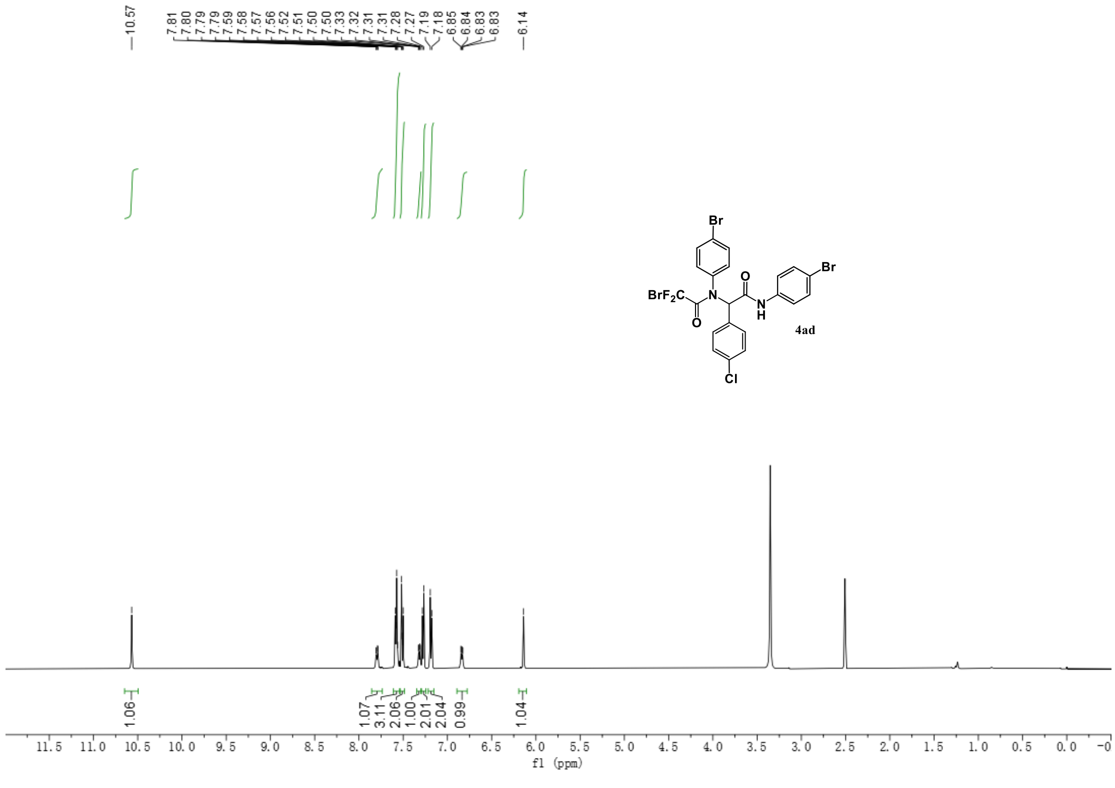

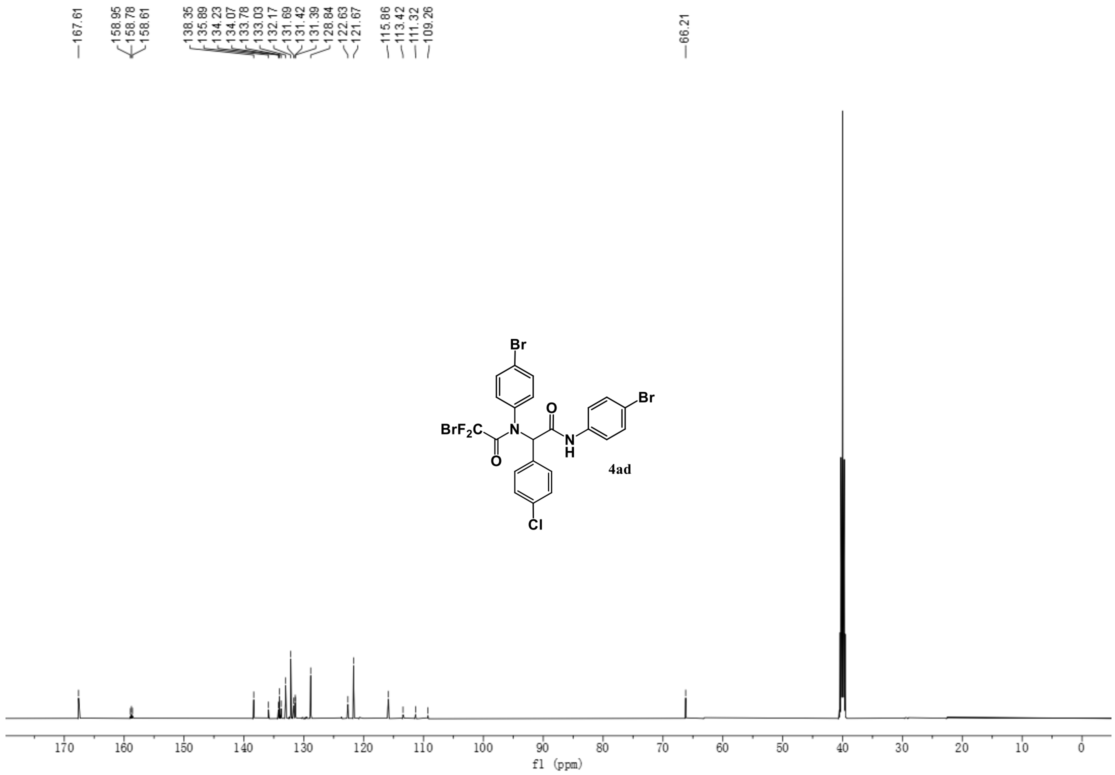
167.61
158.95
158.78
158.61
138.35
135.89
134.23
134.07
133.78
133.03
132.17
131.69
131.42
131.39
128.84
122.63
121.67
115.86
113.42
111.32
109.26
66.21
Br
BrF2C
N
O
H
Br
Cl
4ad
170 160 150 140 130 120 110 100 90 80 70 60 50 40 30 20 10 0
f1 (ppm)

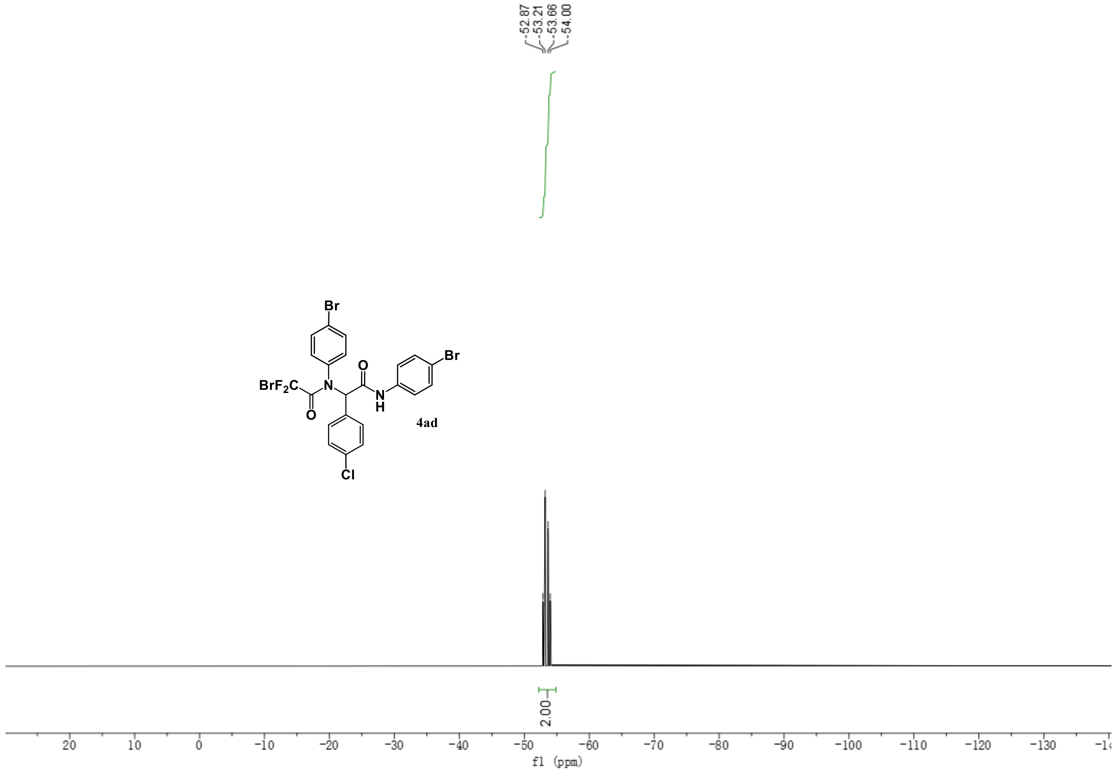
52.87
53.21
53.66
54.00
Br
BrF2C
N
O
H
Br
Cl
4ad
2.00
20 10 0 -10 -20 -30 -40 -50 -60 -70 -80 -90 -100 -110 -120 -130
f1 (ppm)

^{1}H NMR (500 MHz, DMSO), ^{13}C NMR (125 MHz, DMSO) and ^{19}F NMR (471 MHz, DMSO) spectra for 4ae

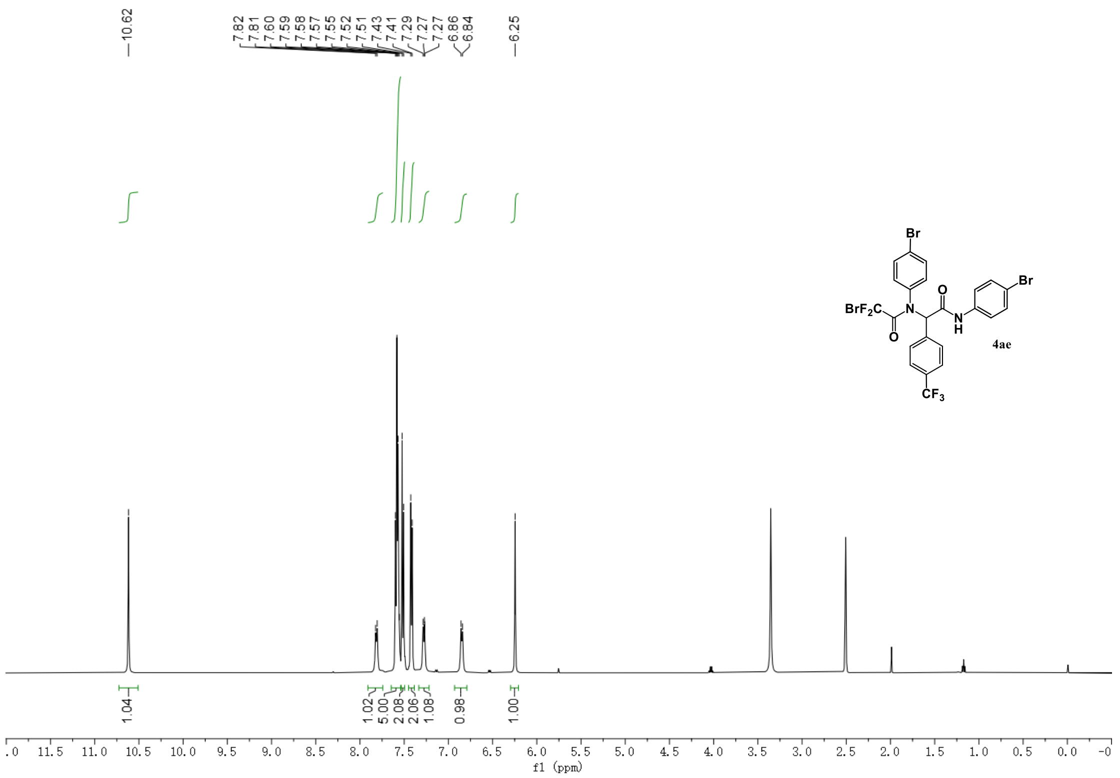

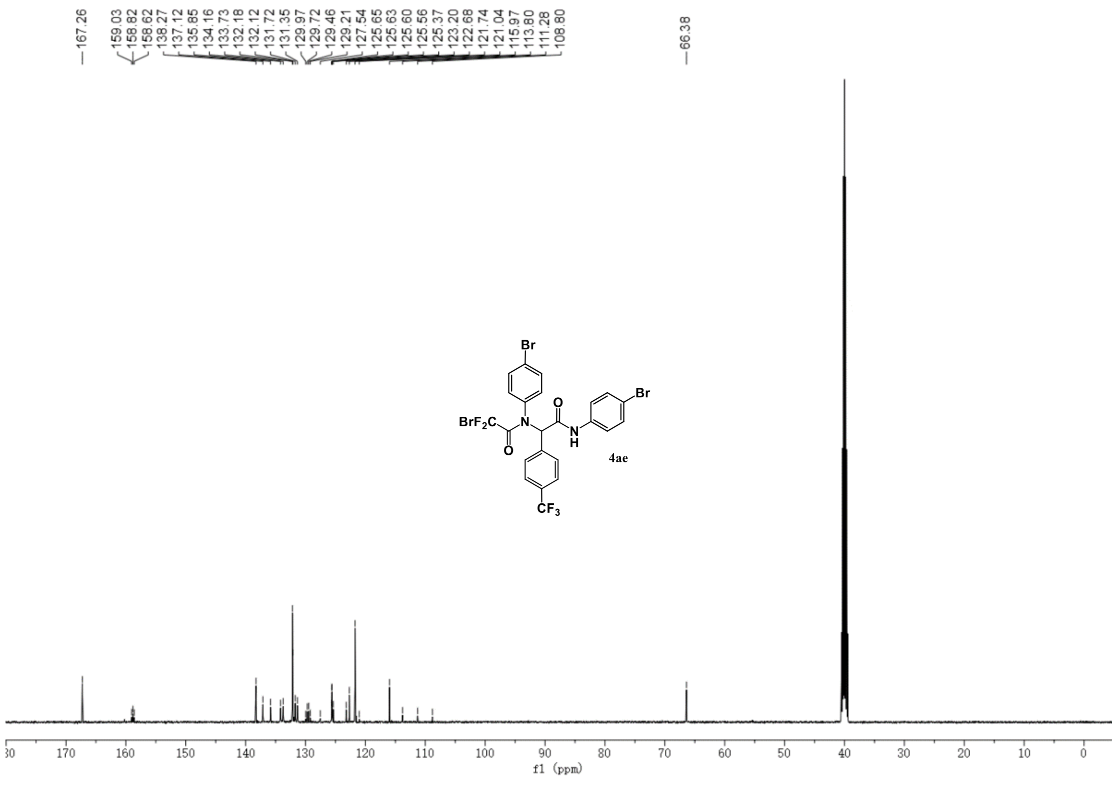

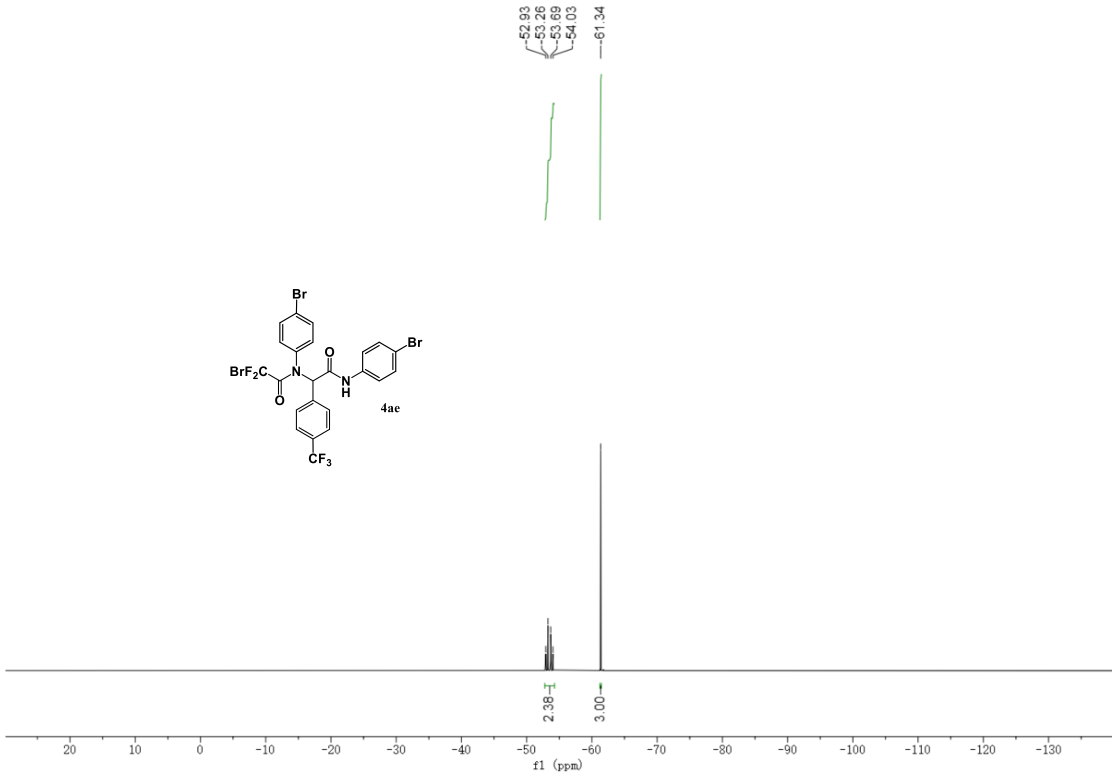

^{1}H NMR (500 MHz, DMSO), ^{13}C NMR (150 MHz, DMSO) and ^{19}F NMR (471 MHz, DMSO) spectra for 4af

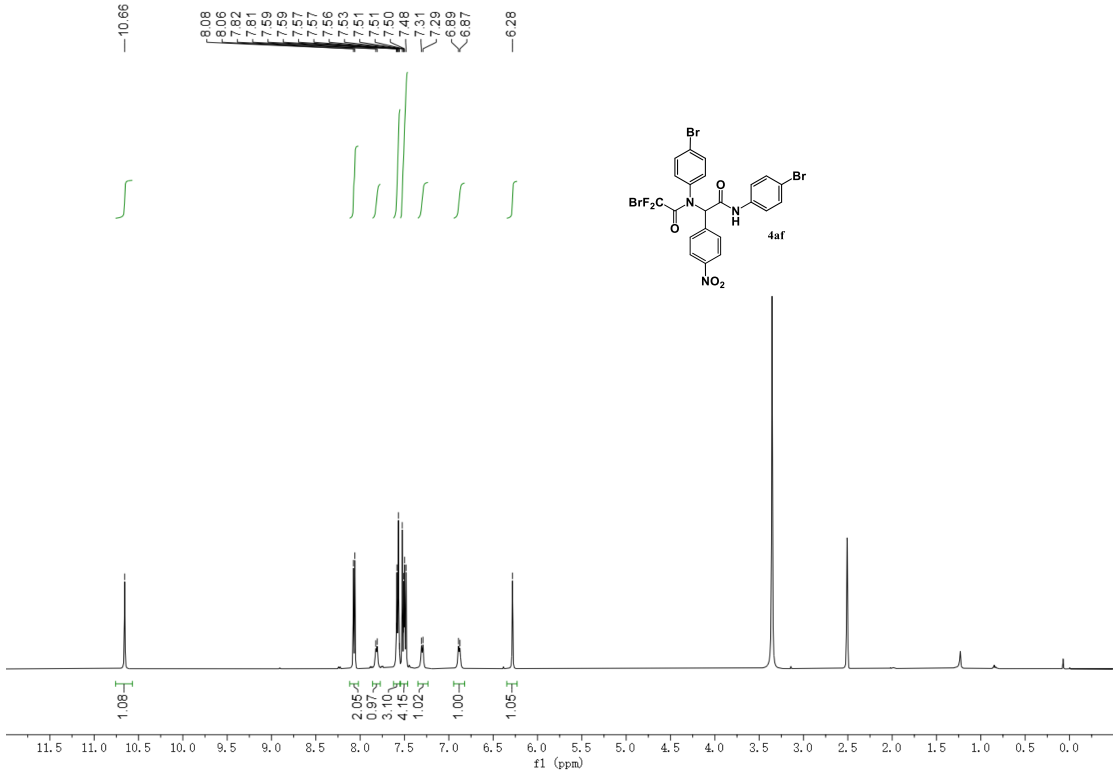

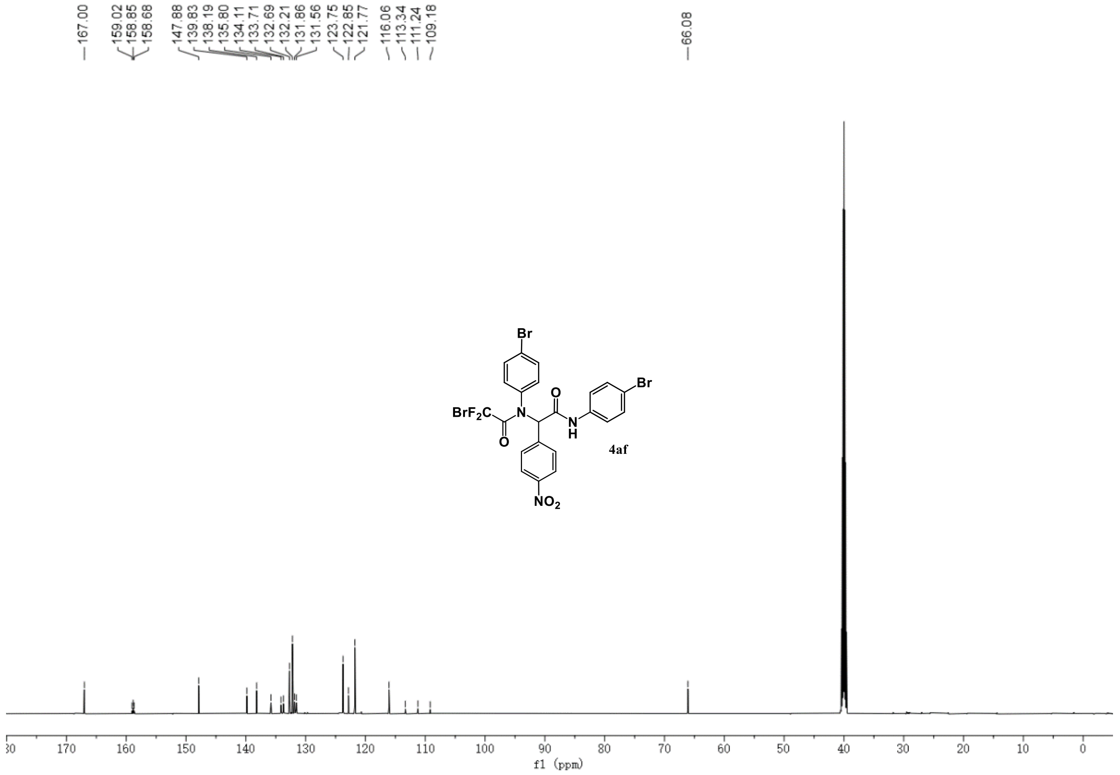
167.00
159.02
158.85
158.68
147.88
139.83
138.19
135.80
134.11
133.71
132.69
132.21
131.86
131.56
123.75
122.85
121.77
116.06
113.34
111.24
109.18
66.08
BrF2C
Br
Br
O
N
N
H
O
NO2
4af
f1 (ppm)

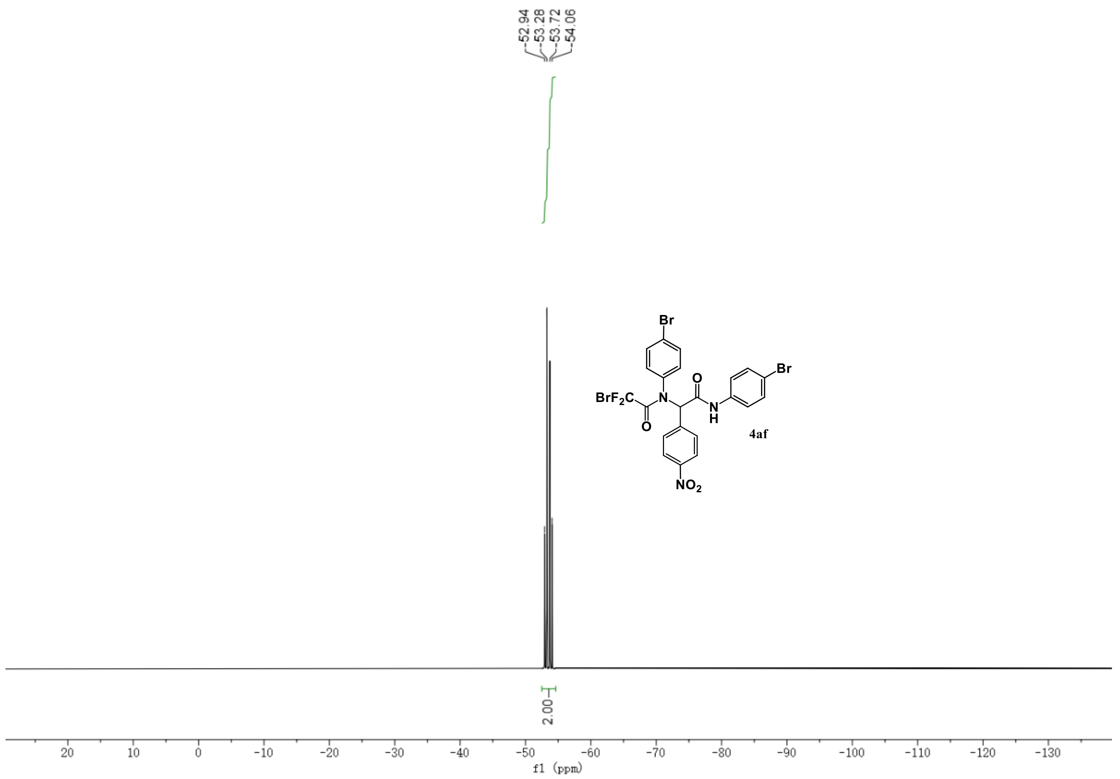
-52.94
-53.28
-53.72
-54.06
BrF2C
Br
Br
O
N
N
H
O
NO2
4af
2.00
f1 (ppm)

^{1}H NMR (500 MHz, DMSO), ^{13}C NMR (150 MHz, DMSO) and ^{19}F NMR (471 MHz, DMSO) spectra for 4ag

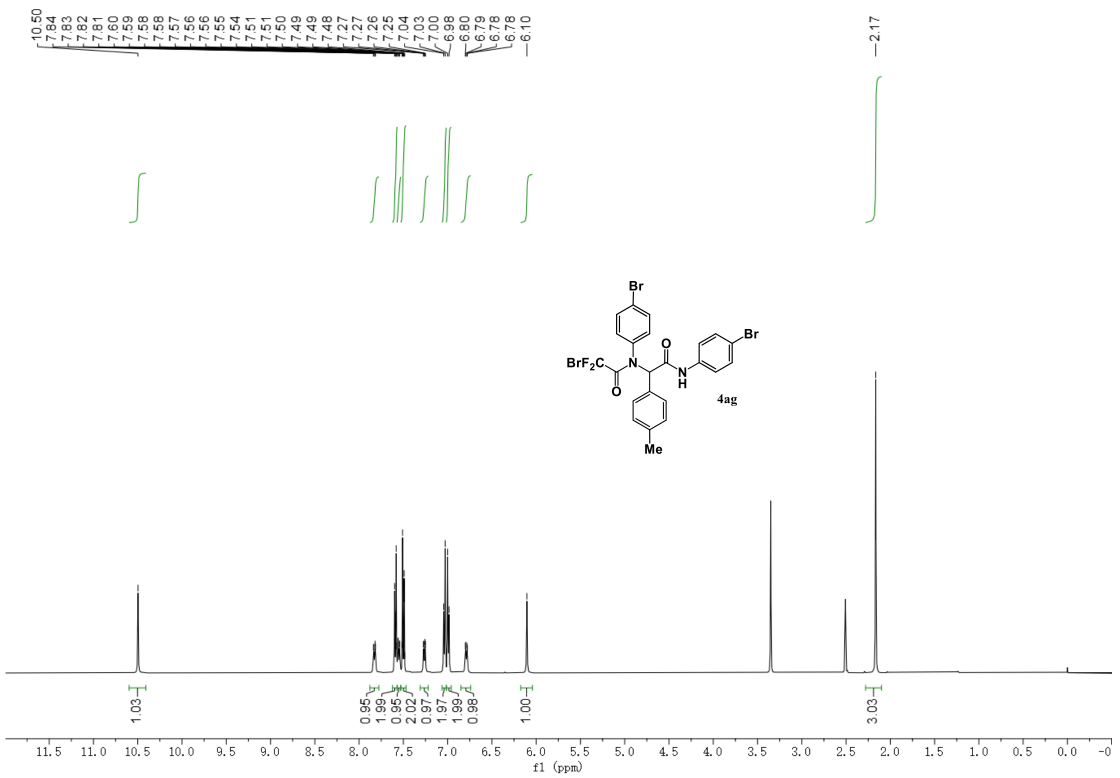

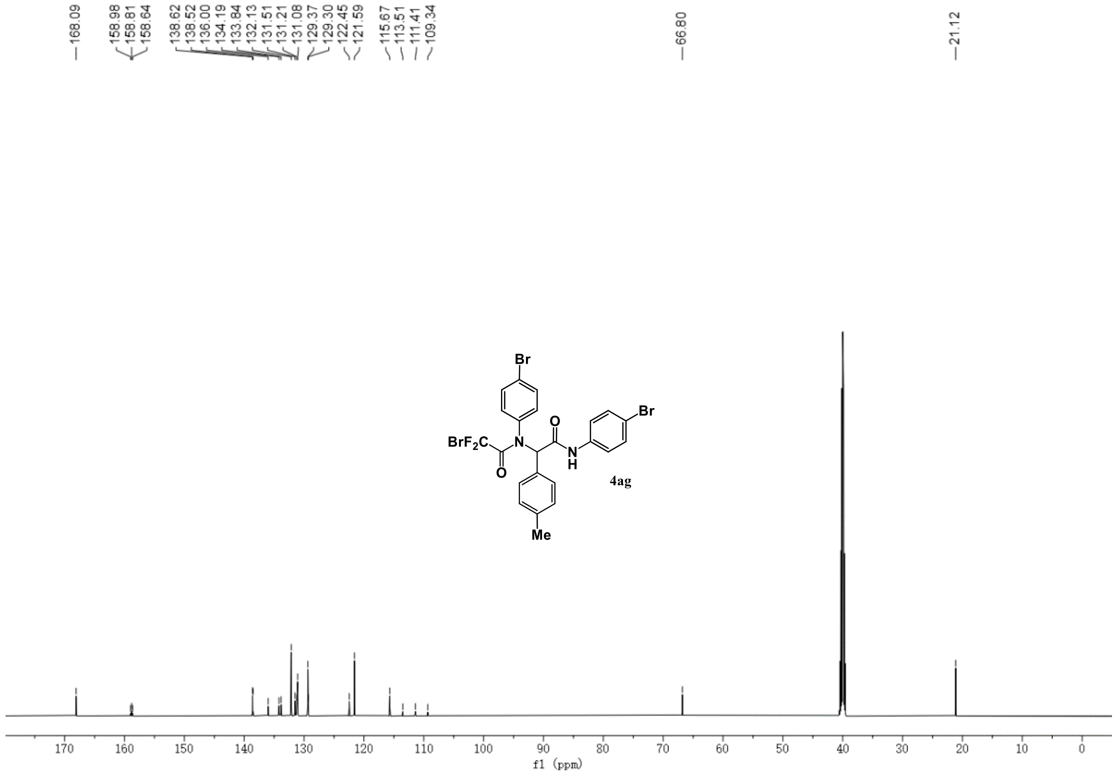

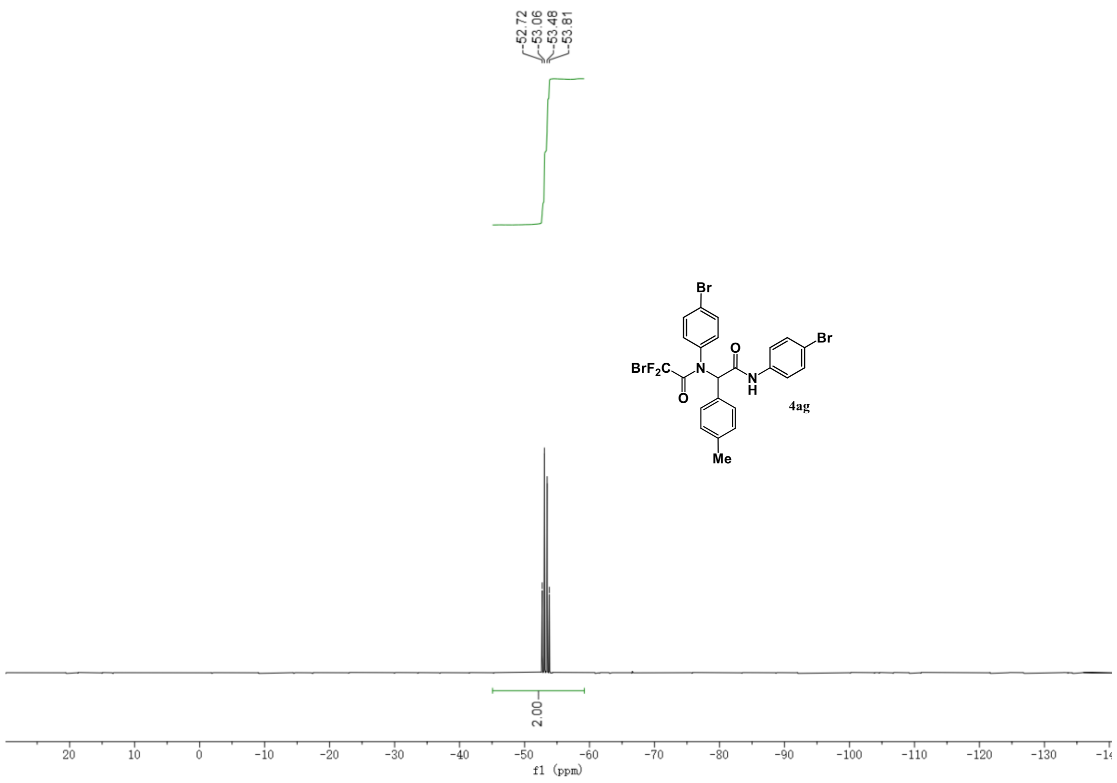

^{1}H NMR (500 MHz, DMSO), ^{13}C NMR (125 MHz, DMSO) and ^{19}F NMR (471 MHz, DMSO) spectra for 4ah

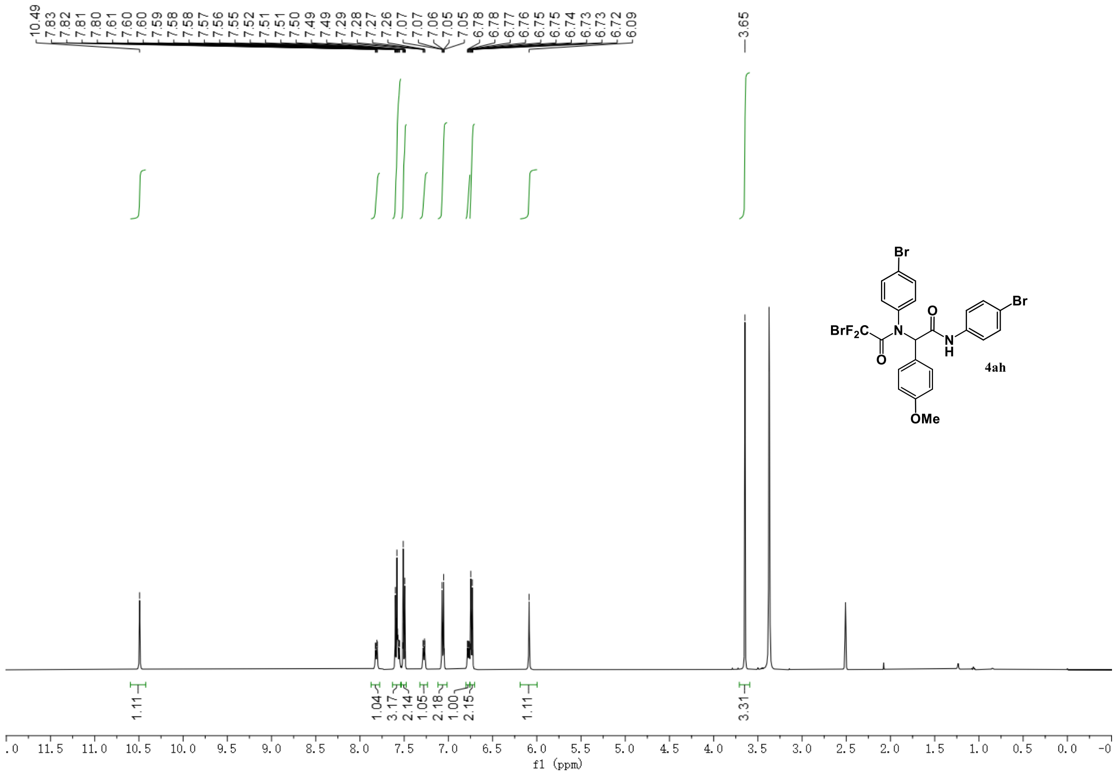

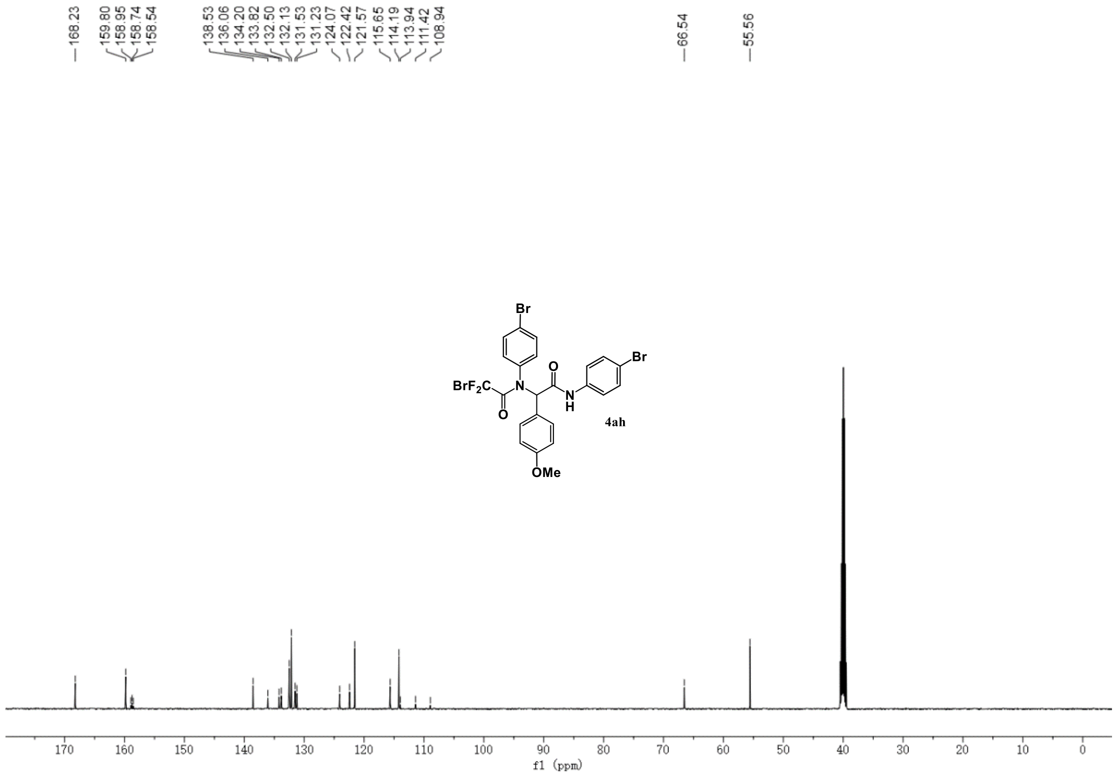
168.23
159.80
158.95
158.74
158.54
138.53
136.06
134.20
133.82
132.50
132.13
131.53
131.23
124.07
122.42
121.57
115.65
114.19
113.94
111.42
108.94
66.54
55.56
Br
BrF2C
N
O
O
N
H
Br
OMe
4ah
f1 (ppm)

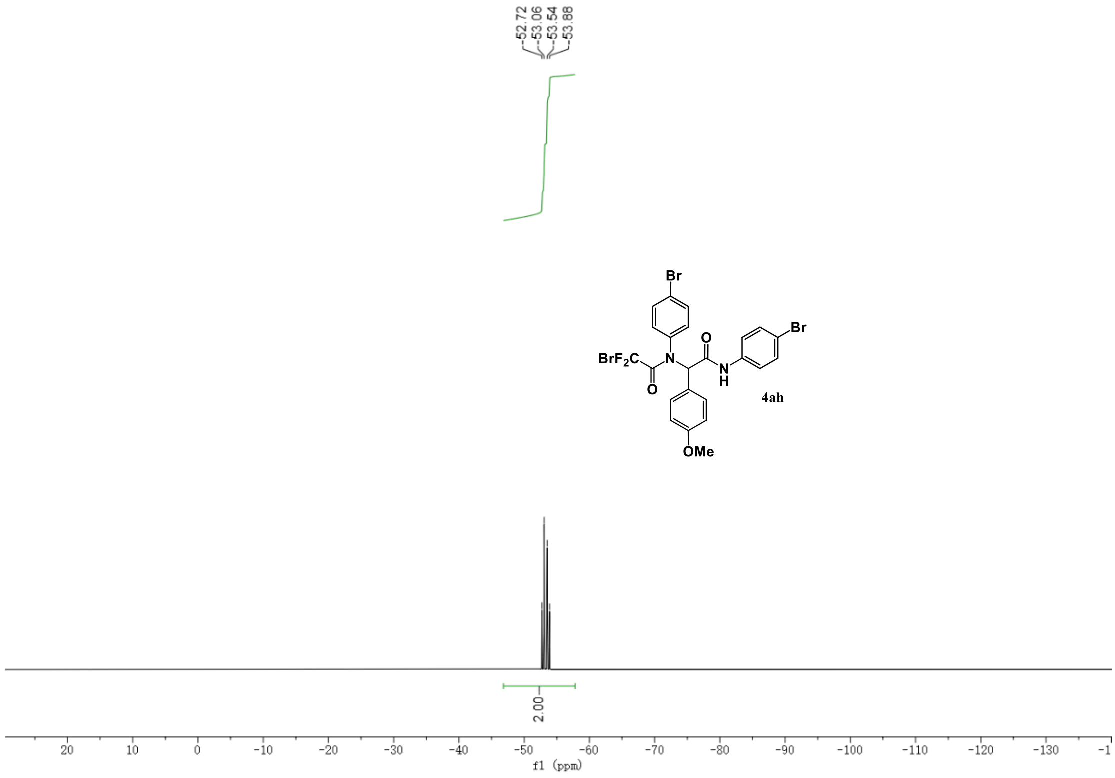
52.72
53.06
53.54
53.88
Br
BrF2C
N
O
O
N
H
Br
OMe
4ah
2.00
f1 (ppm)

^{1}H NMR (500 MHz, DMSO), ^{13}C NMR (150 MHz, DMSO) and ^{19}F NMR (471 MHz, DMSO) spectra for 4ai

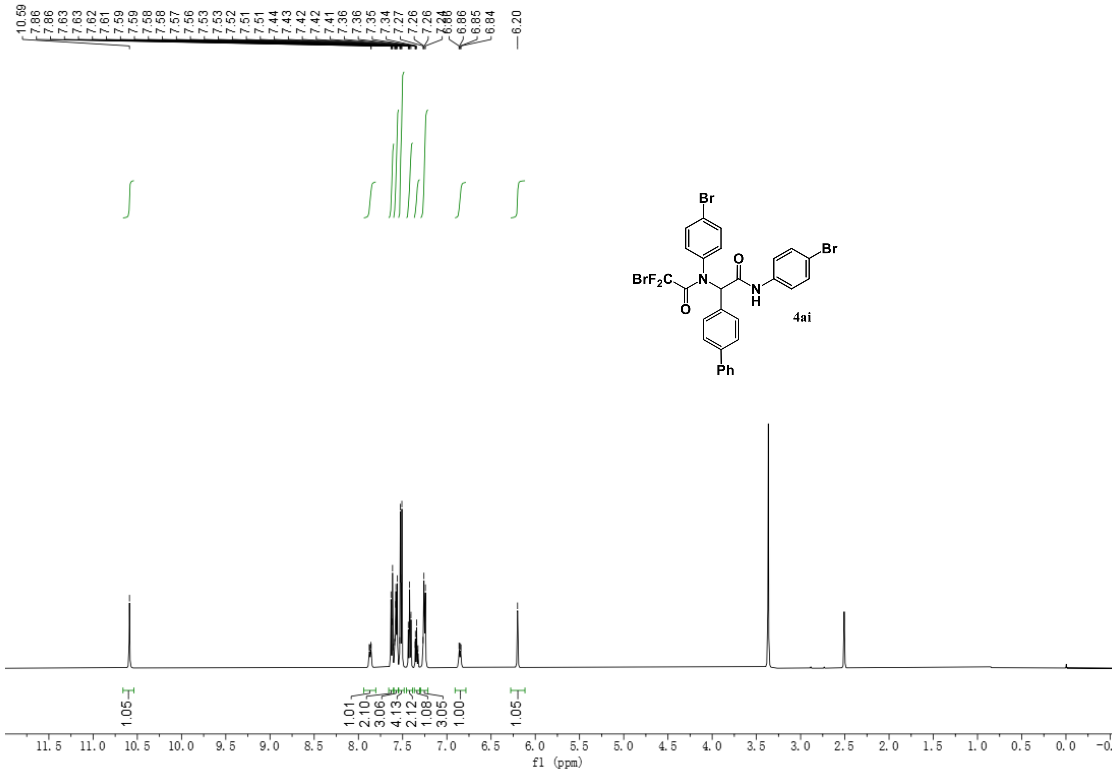

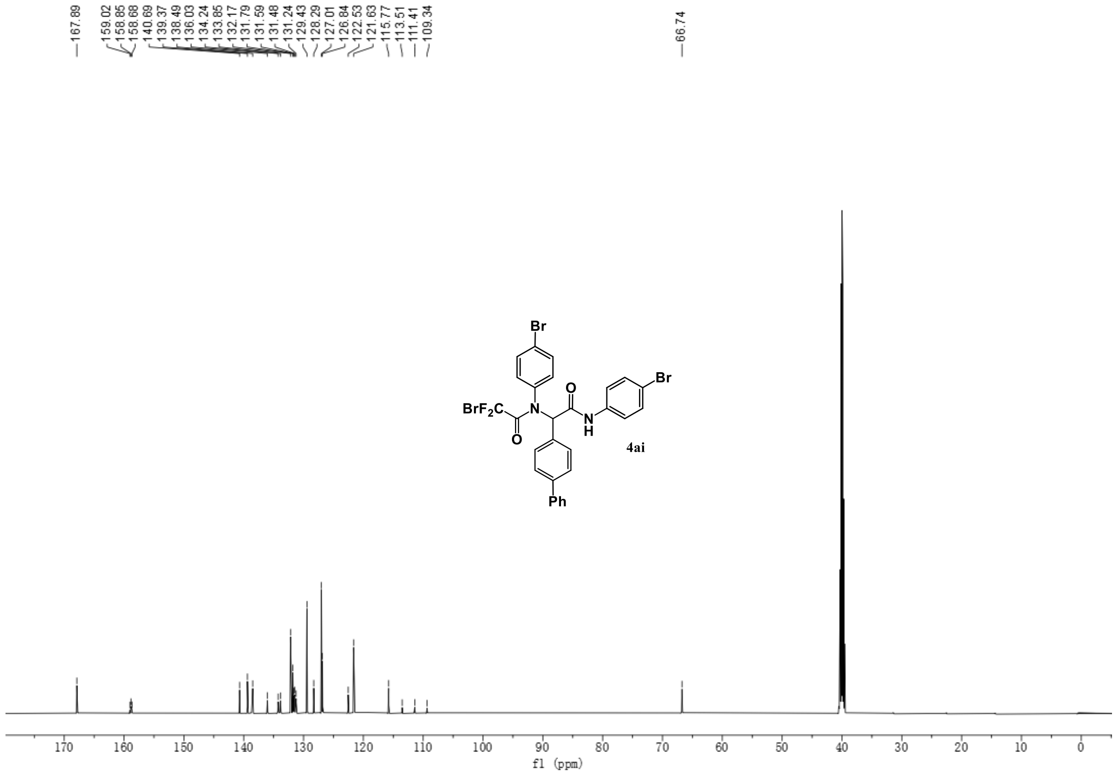

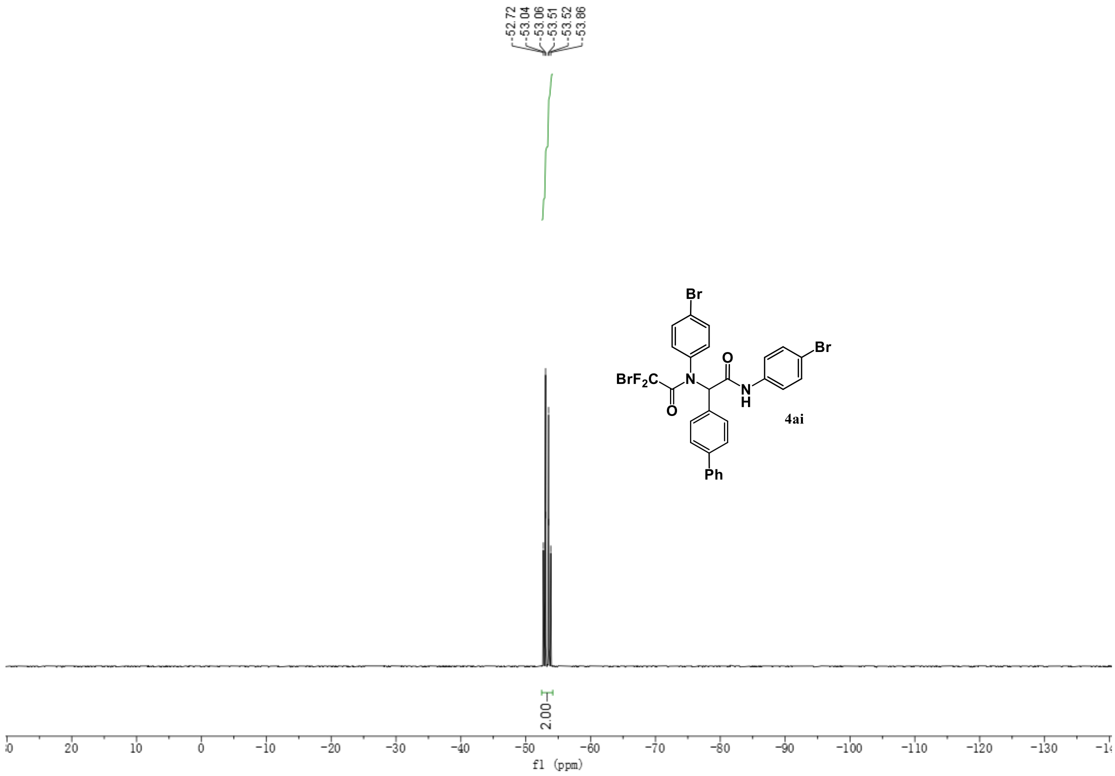

^{1}H NMR (500 MHz, DMSO), ^{13}C NMR (125 MHz, DMSO) and ^{19}F NMR (471 MHz, DMSO) spectra for 4aj

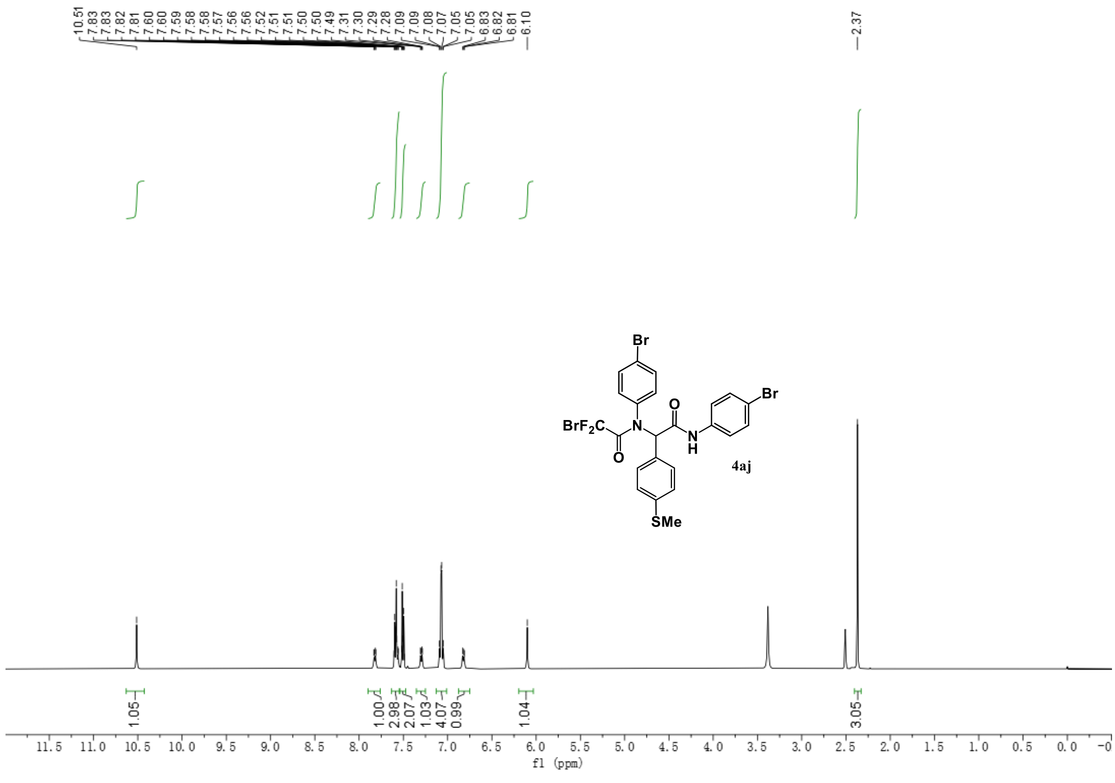

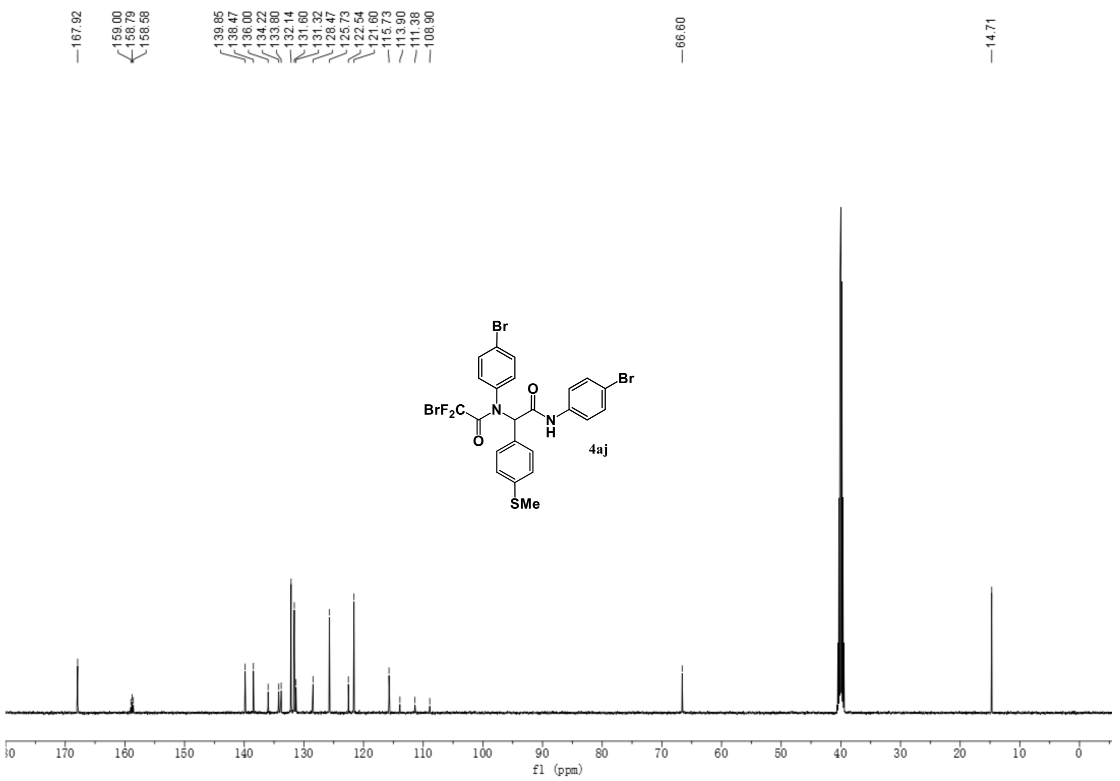
167.92
159.00
158.79
158.58
139.85
138.47
136.00
134.22
133.80
132.14
131.60
131.32
128.47
125.73
122.54
121.60
115.73
113.90
111.38
108.90
66.60
14.71
Br
BrF2C
N
O
H
SMe
4aj
f1 (ppm)

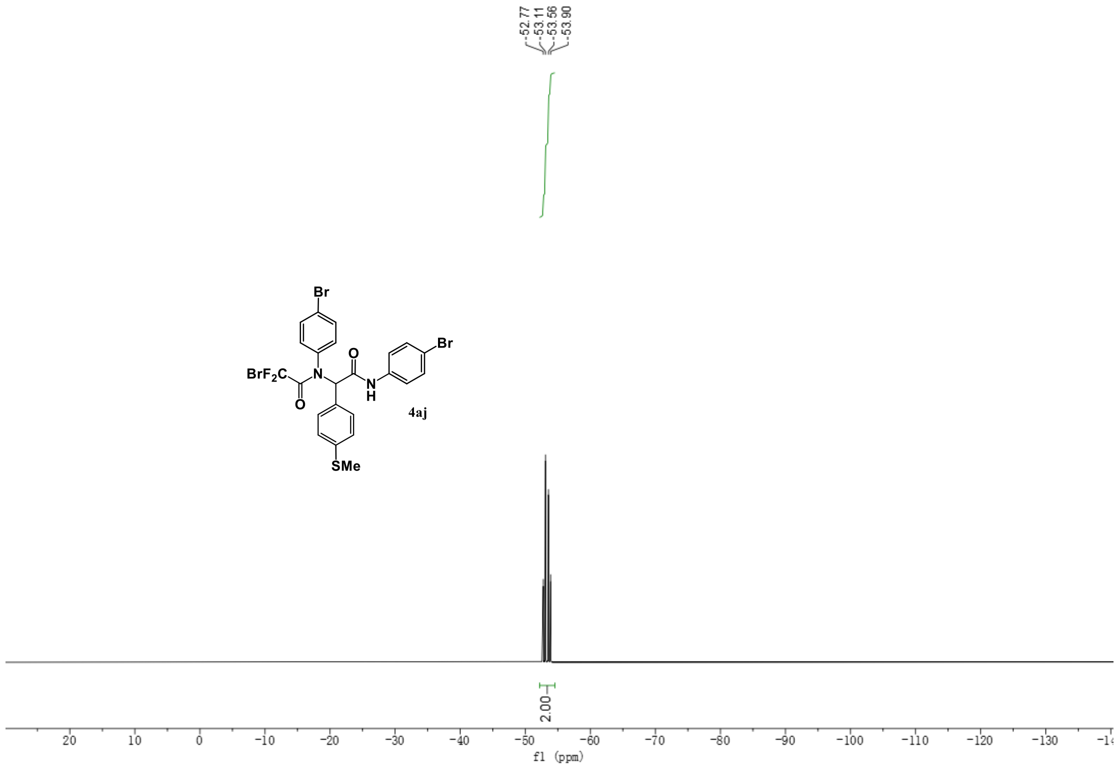
-52.77
-53.11
-53.56
-53.90
Br
BrF2C
N
O
H
SMe
4aj
2.00
f1 (ppm)

^{1}H NMR (500 MHz, DMSO), ^{13}C NMR (150 MHz, DMSO) and ^{19}F NMR (471 MHz, DMSO) spectra for 4ak

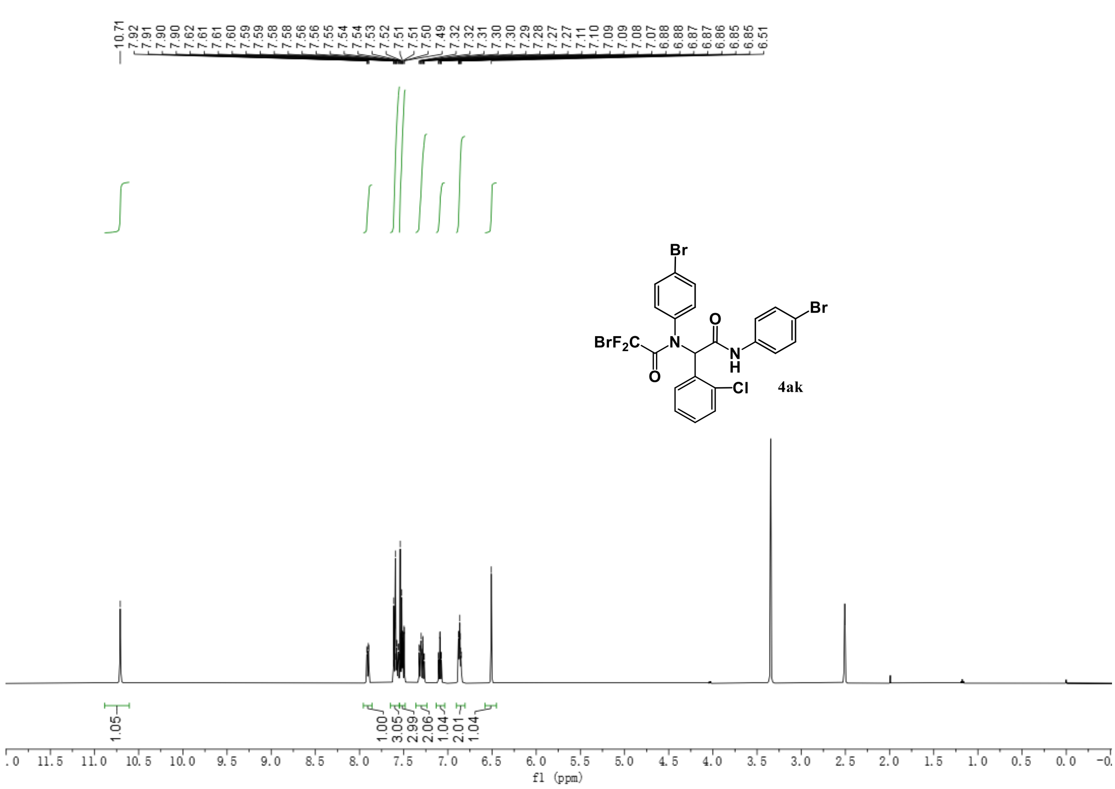

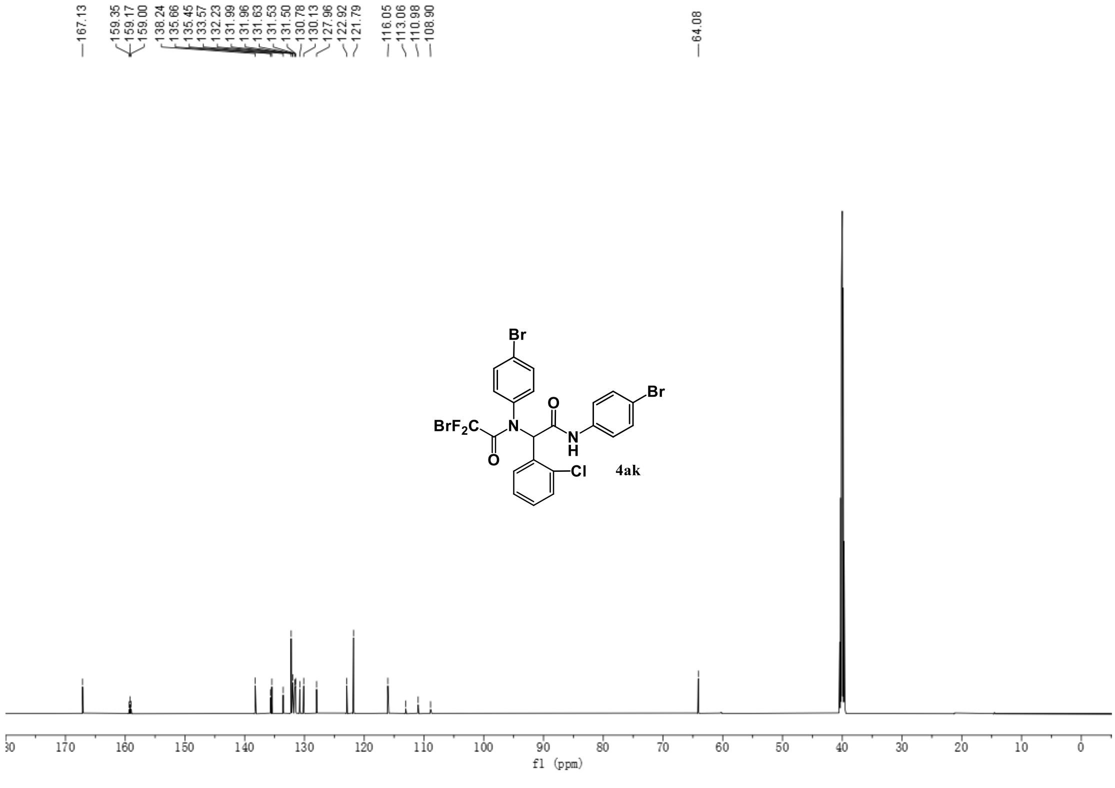

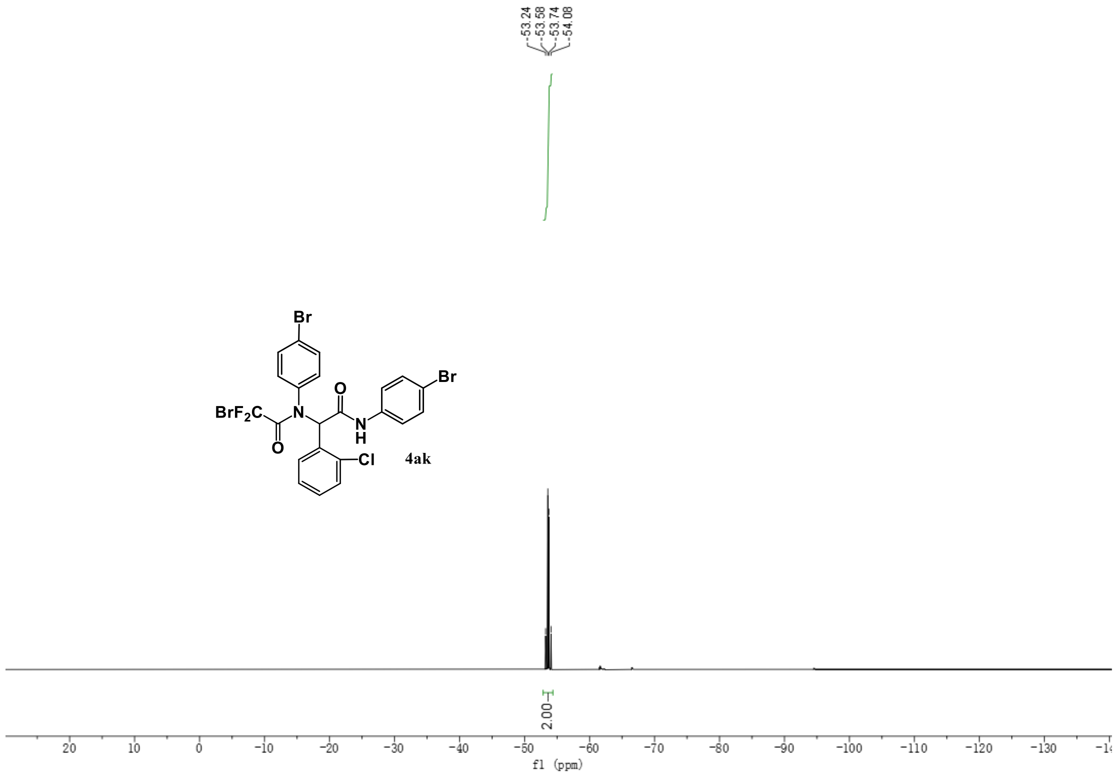

^{1}H NMR (500 MHz, DMSO), ^{13}C NMR (125 MHz, DMSO) and ^{19}F NMR (471 MHz, DMSO) spectra for 4al

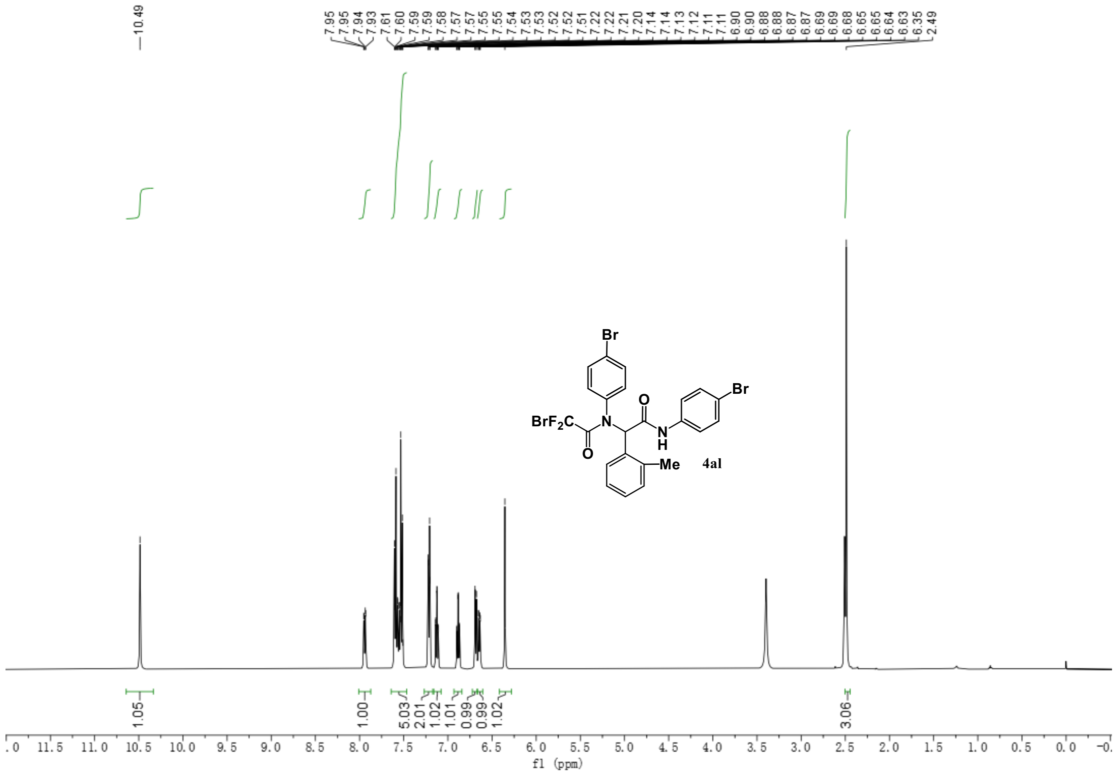

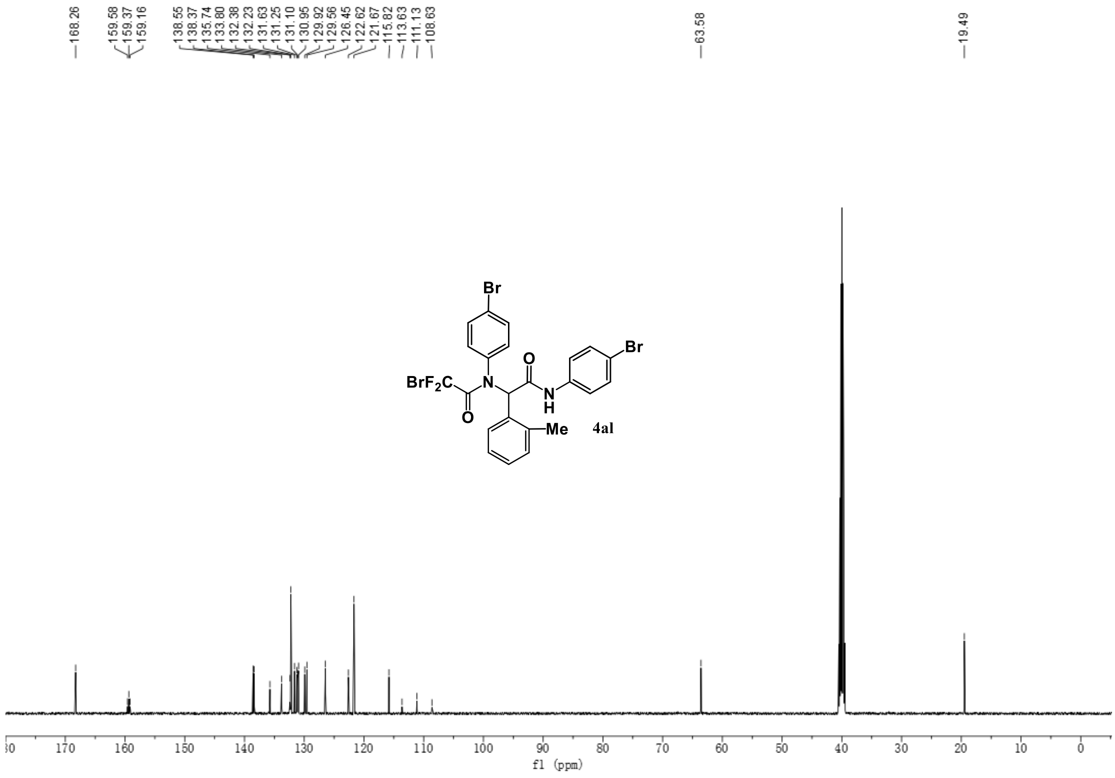
168.26
159.58
159.37
159.16
138.55
138.37
135.74
133.80
132.38
132.23
131.63
131.25
131.10
130.95
129.92
129.56
126.45
122.62
121.67
115.82
113.63
111.13
108.63
63.58
19.49
4al
fl (ppm)

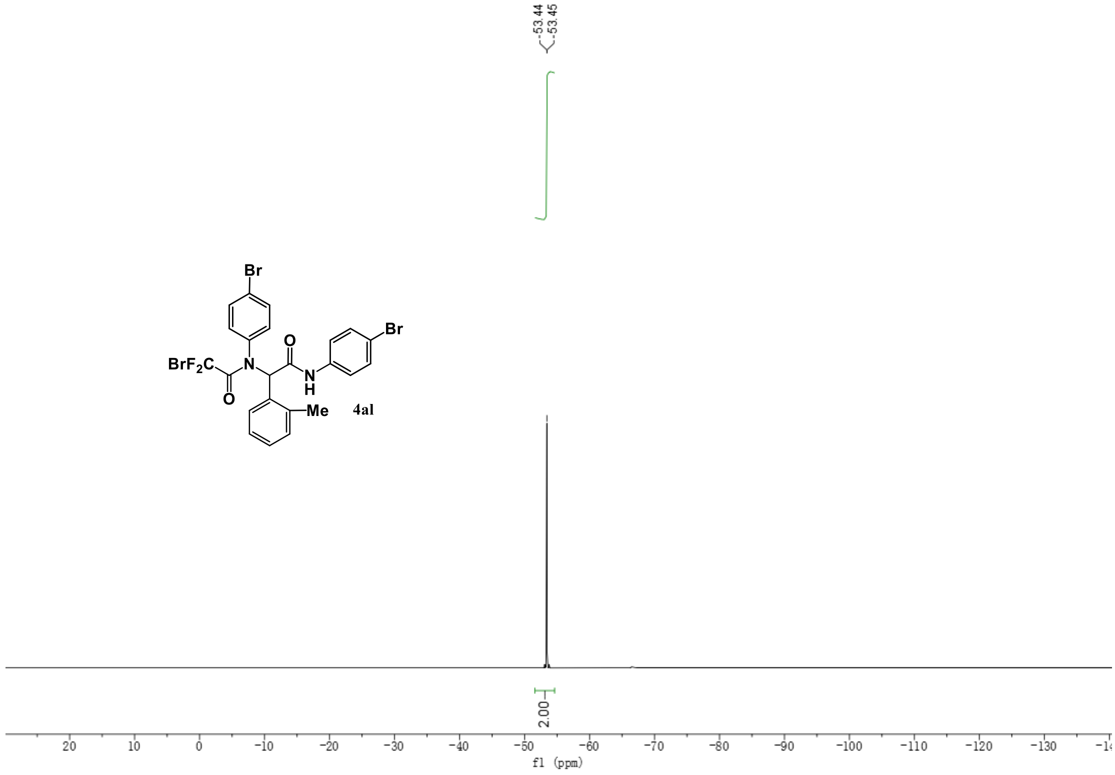
-53.44
-53.45
4al
2.00
fl (ppm)

^1H NMR (500 MHz, DMSO), ^{13}C NMR (150 MHz, DMSO) and ^{19}F NMR (471 MHz, DMSO) spectra for 4am

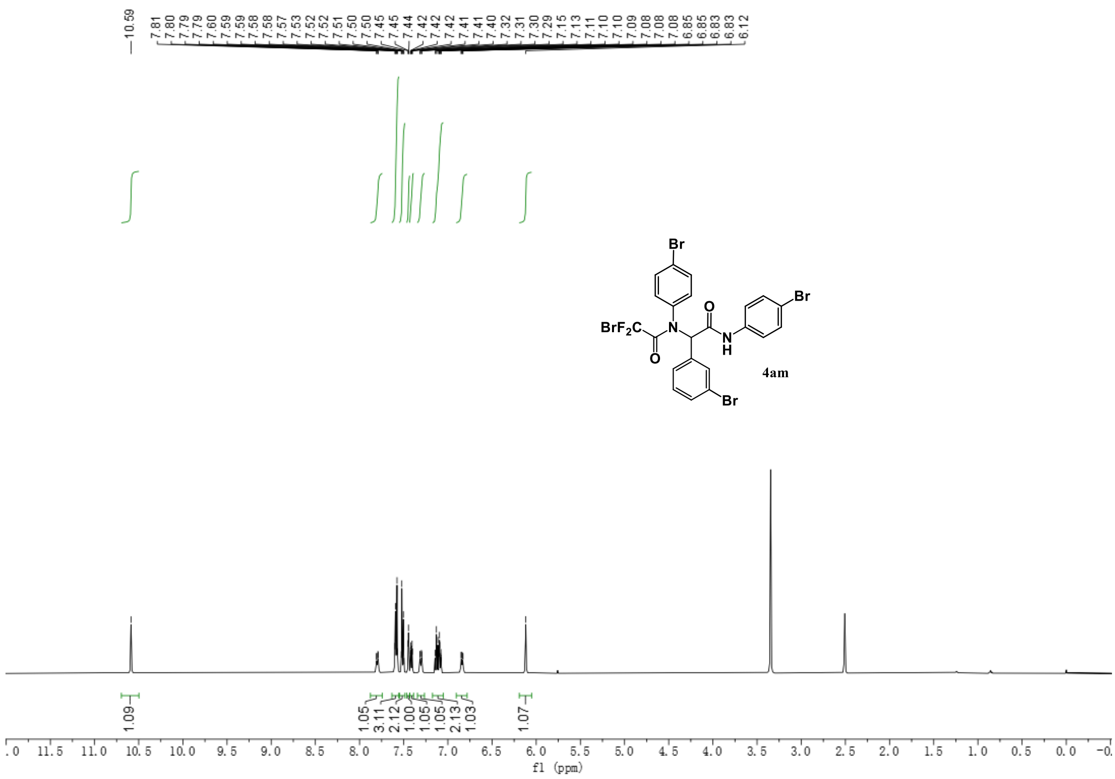

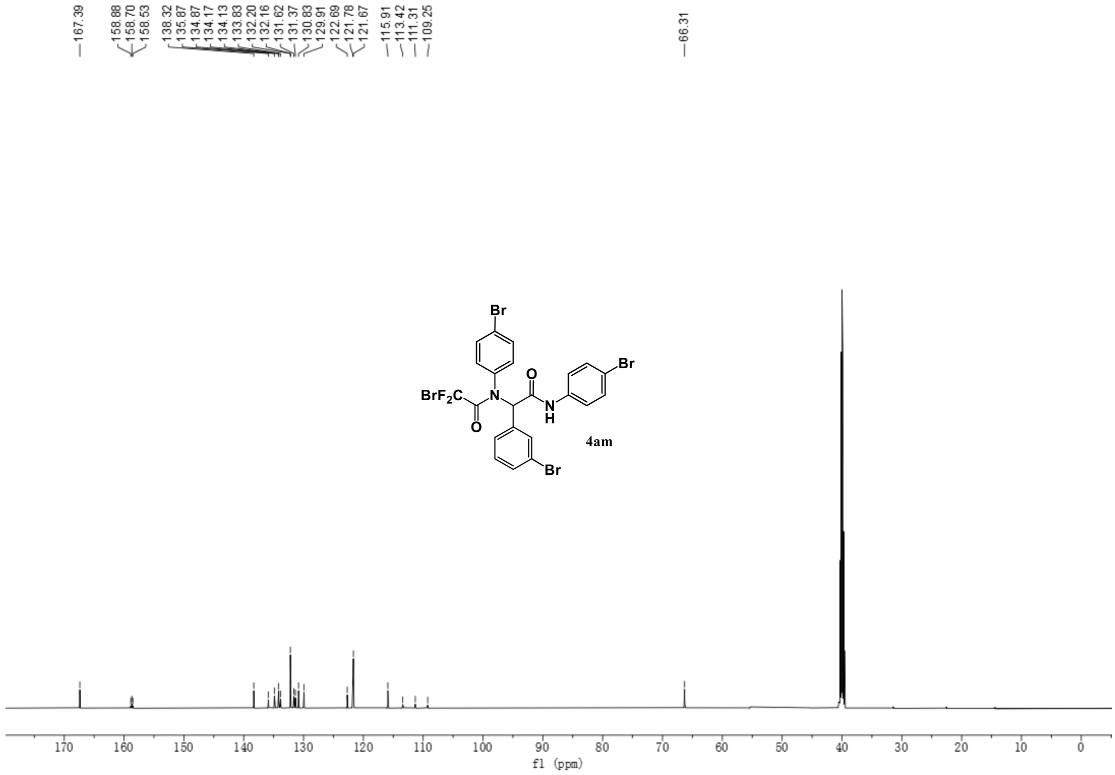

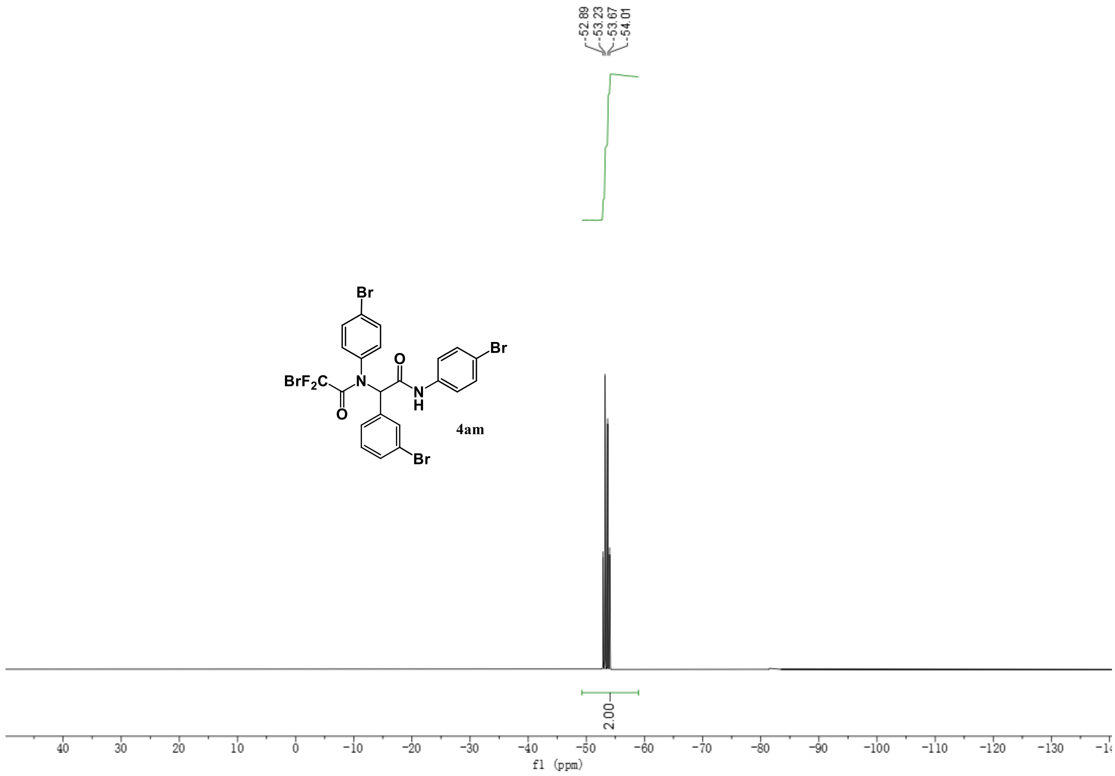

4am

^{1}H NMR (500 MHz, DMSO), ^{13}C NMR (150 MHz, DMSO) and ^{19}F NMR (471 MHz, DMSO) spectra for 4an

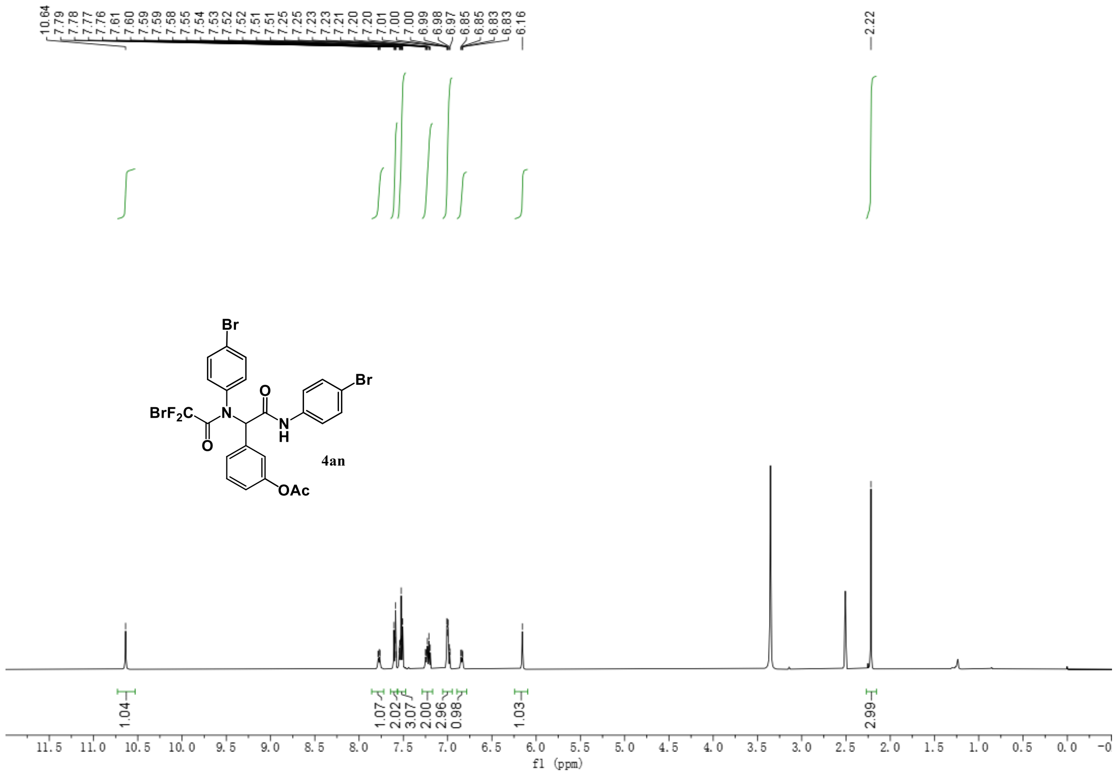

4an

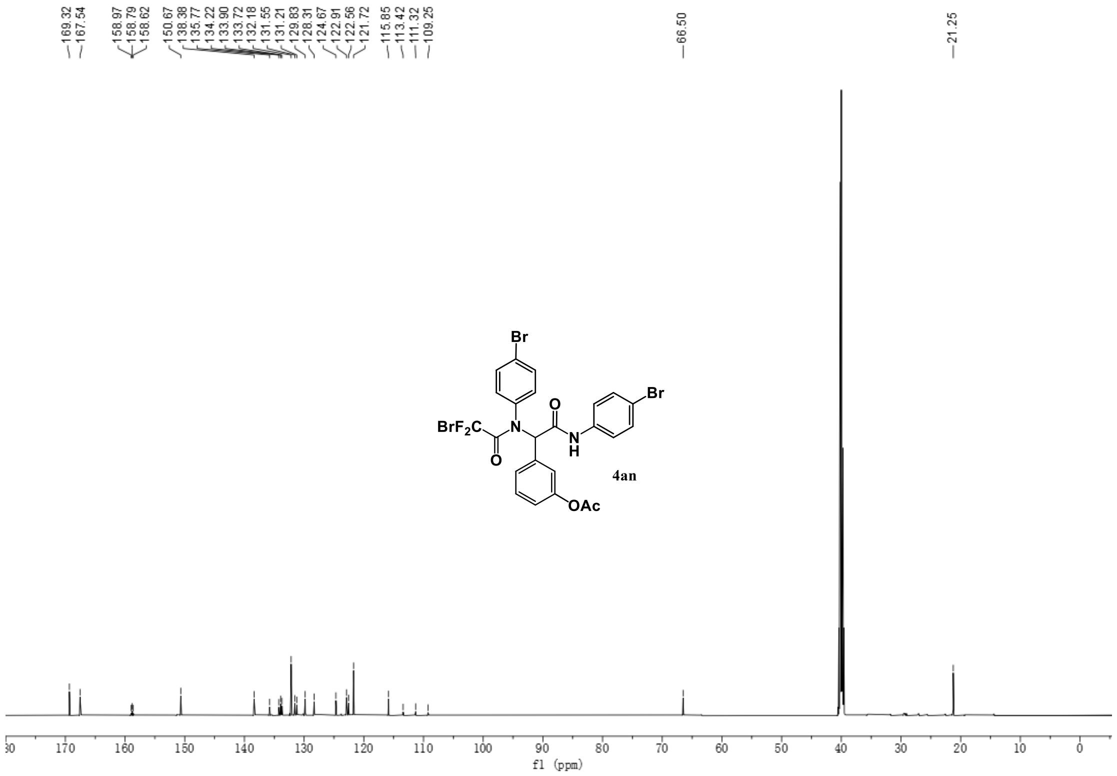
169.32
167.54
158.97
158.79
158.62
150.67
138.38
135.77
134.22
133.90
133.72
132.18
131.55
131.21
129.83
128.31
124.67
122.91
122.56
121.72
115.85
113.42
111.32
109.25
66.50
21.25
Br
Br
BrF2C
N
O
O
N
H
4an
OAc
f1 (ppm)

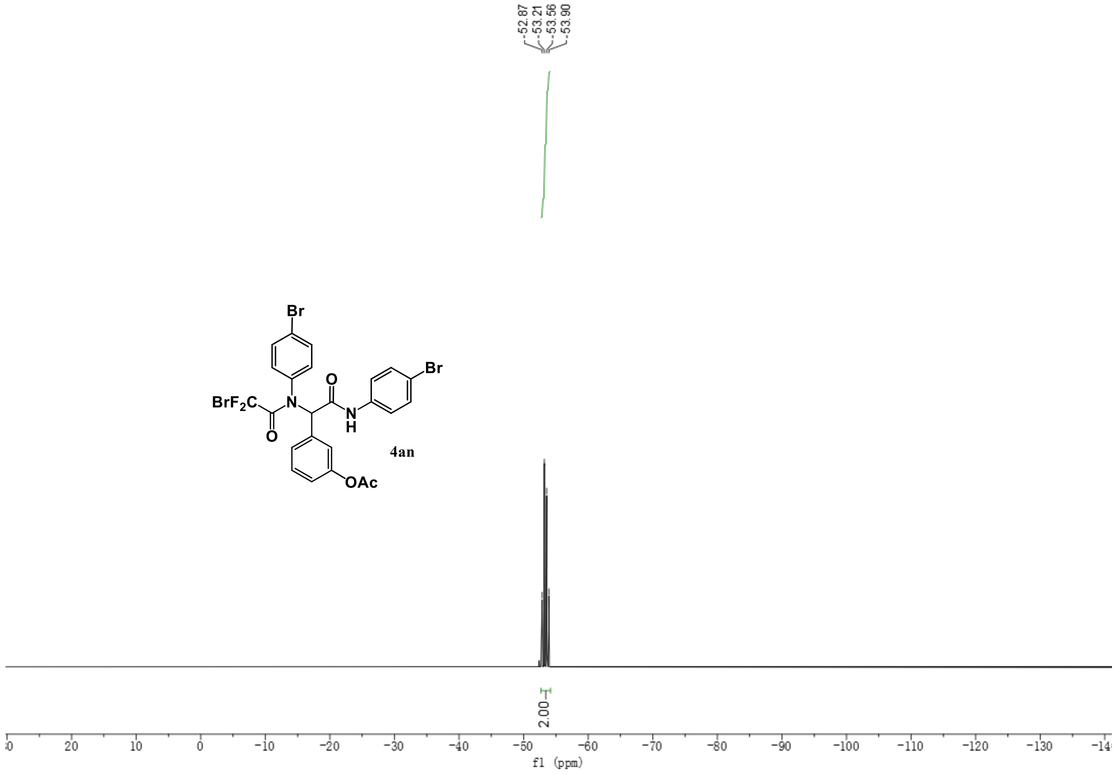
52.87
53.21
53.56
53.90
Br
Br
BrF2C
N
O
O
N
H
4an
OAc
2.00
f1 (ppm)

^{1}H NMR (500 MHz, DMSO), ^{13}C NMR (125 MHz, DMSO) and ^{19}F NMR (471 MHz, DMSO) spectra for 4ao

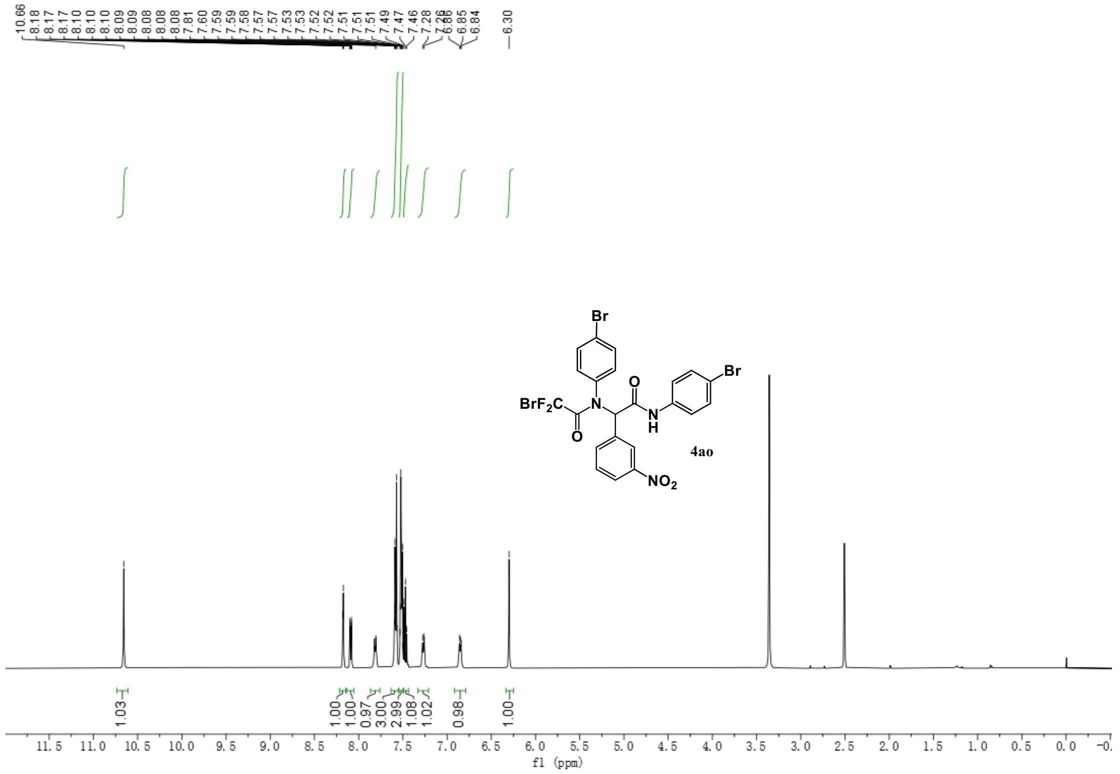

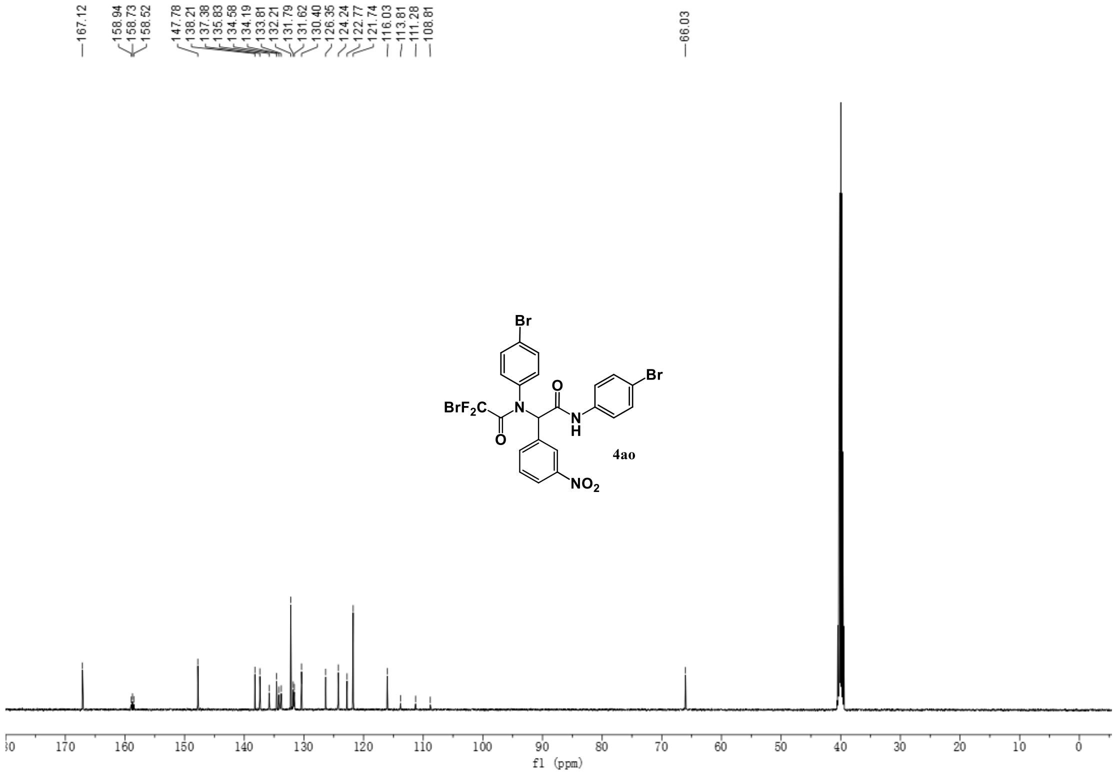

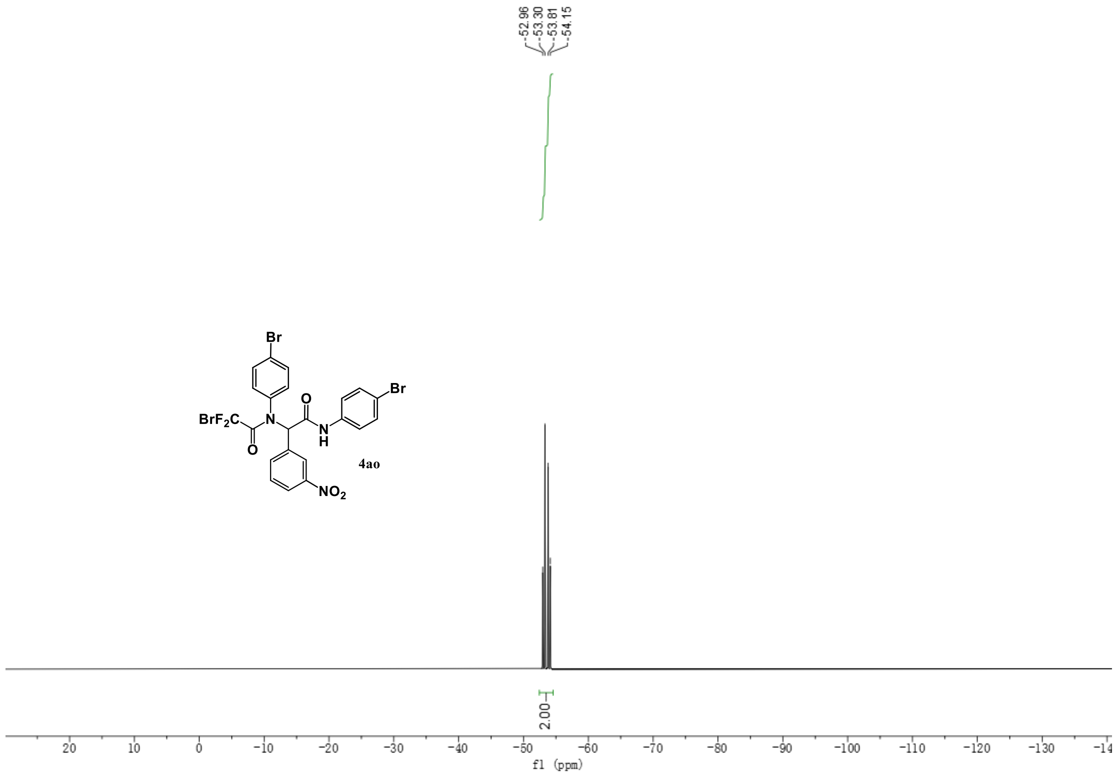

^{1}H NMR (500 MHz, DMSO), ^{13}C NMR (150 MHz, DMSO) and ^{19}F NMR (471 MHz, DMSO) spectra for 4ap

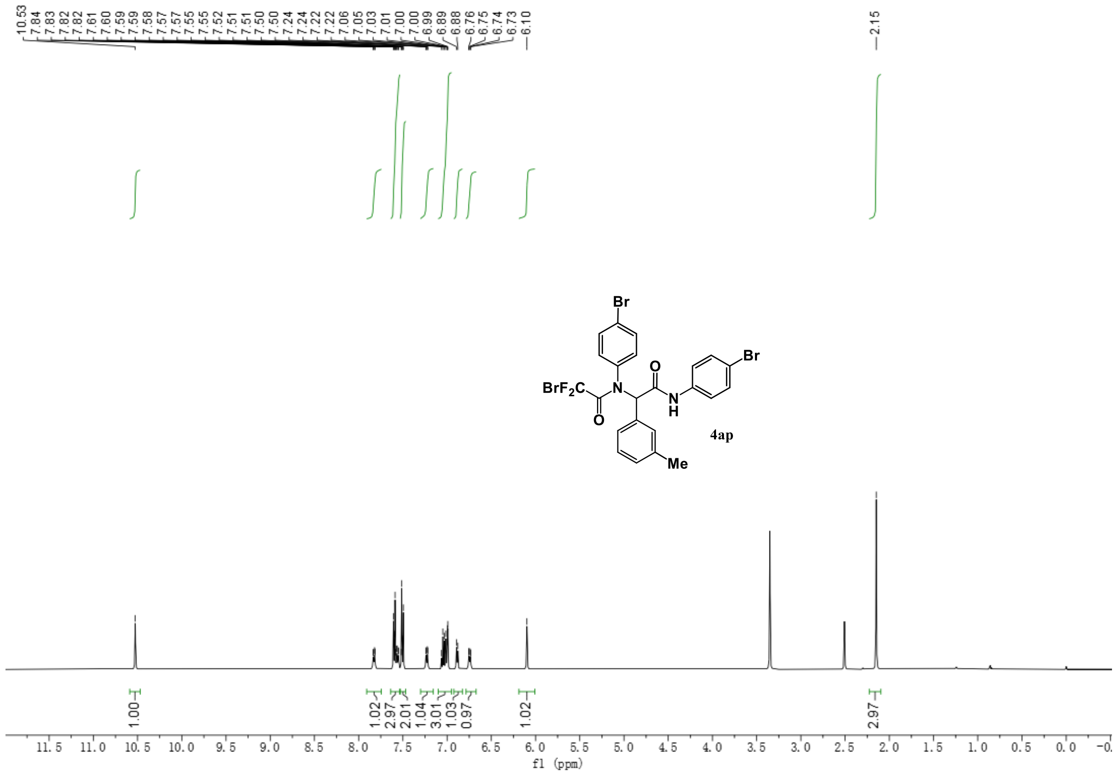

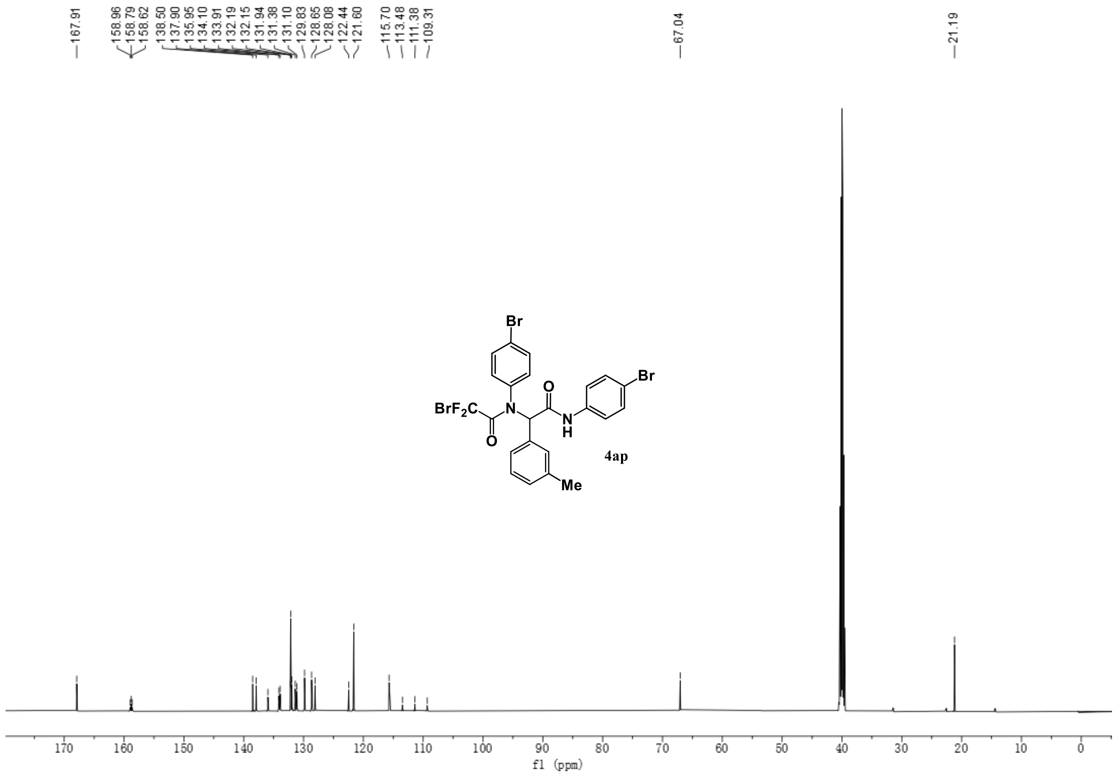
167.91
158.96
158.79
158.62
138.50
137.90
135.95
134.10
133.91
132.19
132.15
131.94
131.38
131.10
129.83
128.65
128.08
122.44
121.60
115.70
113.48
111.38
109.31
67.04
21.19
Br
BrF2C
N
O
H
4ap
Me
f1 (ppm)

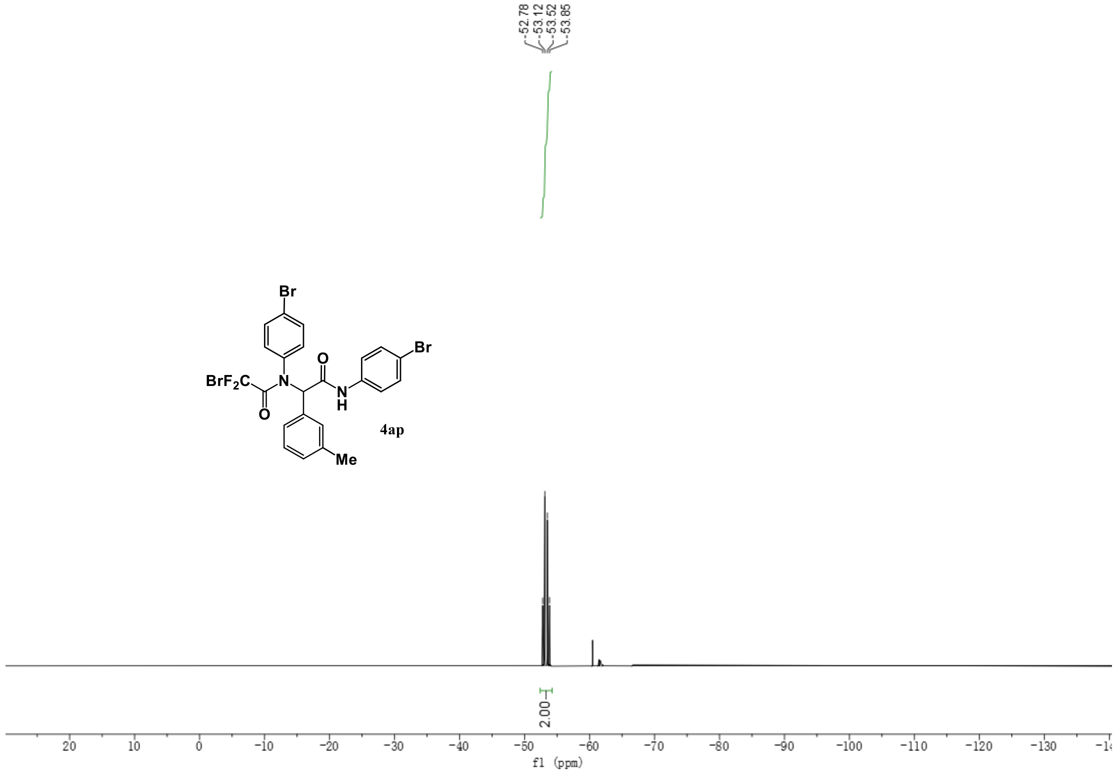
-52.78
-53.12
-53.52
-53.85
Br
BrF2C
N
O
H
4ap
Me
2.00
f1 (ppm)

^{1}H NMR (500 MHz, DMSO), ^{13}C NMR (125 MHz, DMSO) and ^{19}F NMR (471 MHz, DMSO) spectra for 4aq

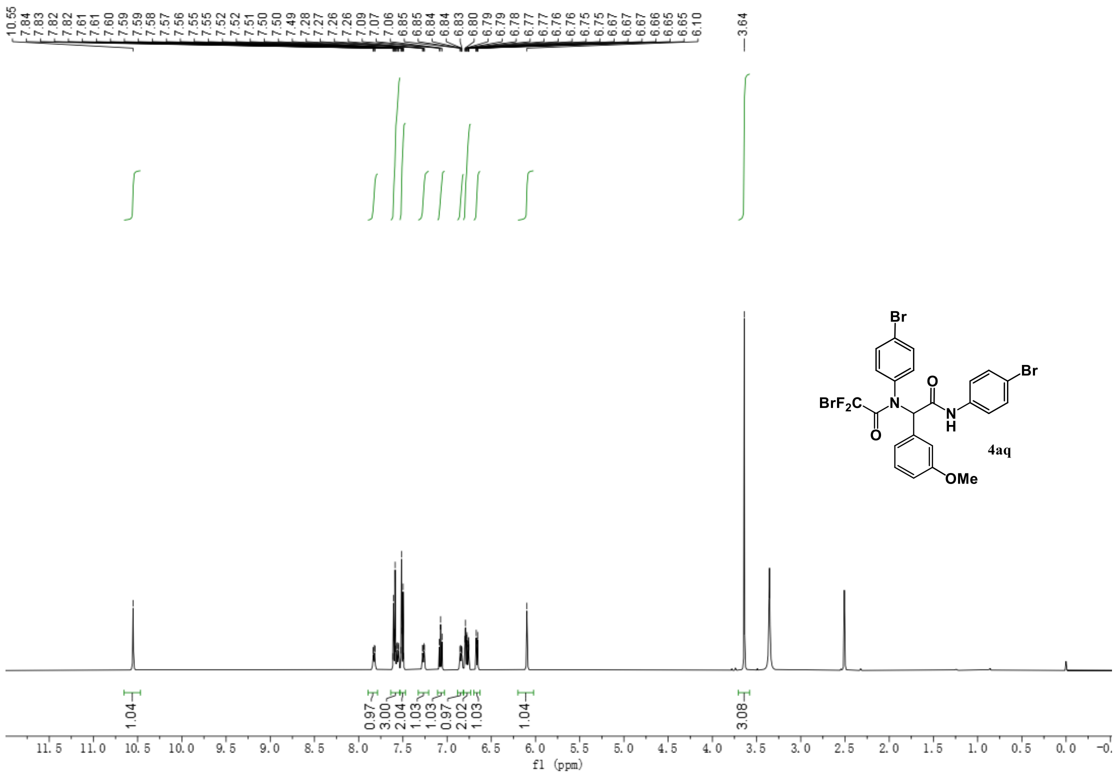

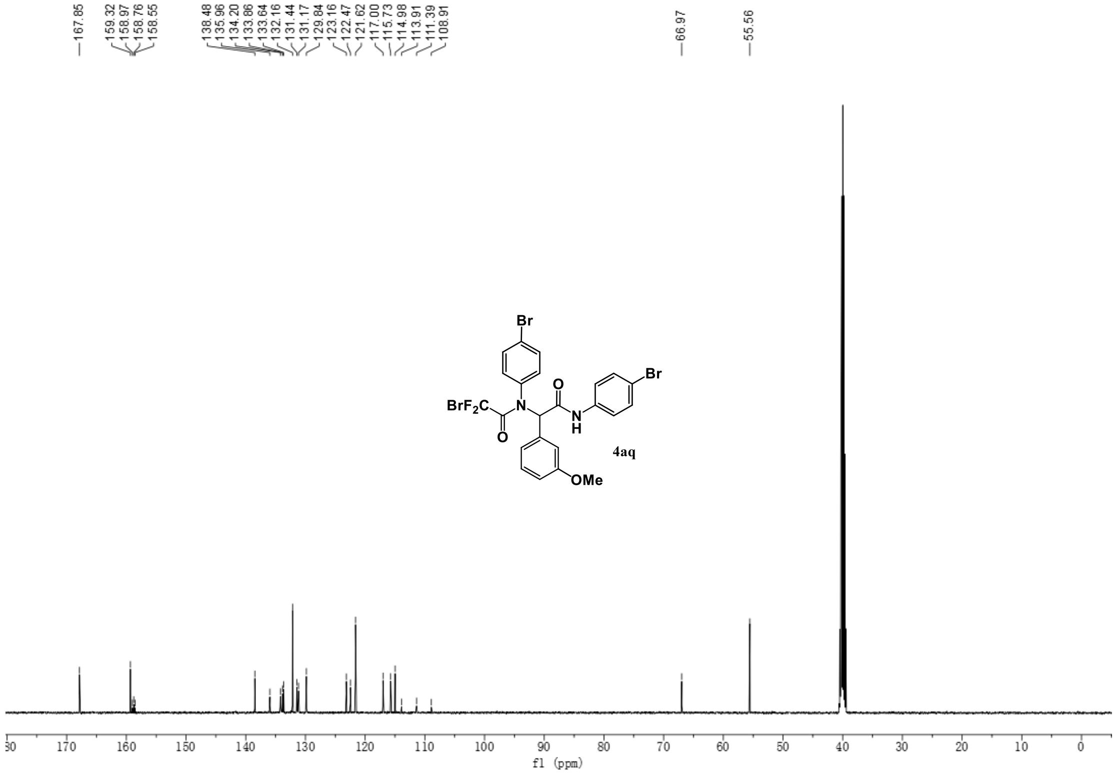

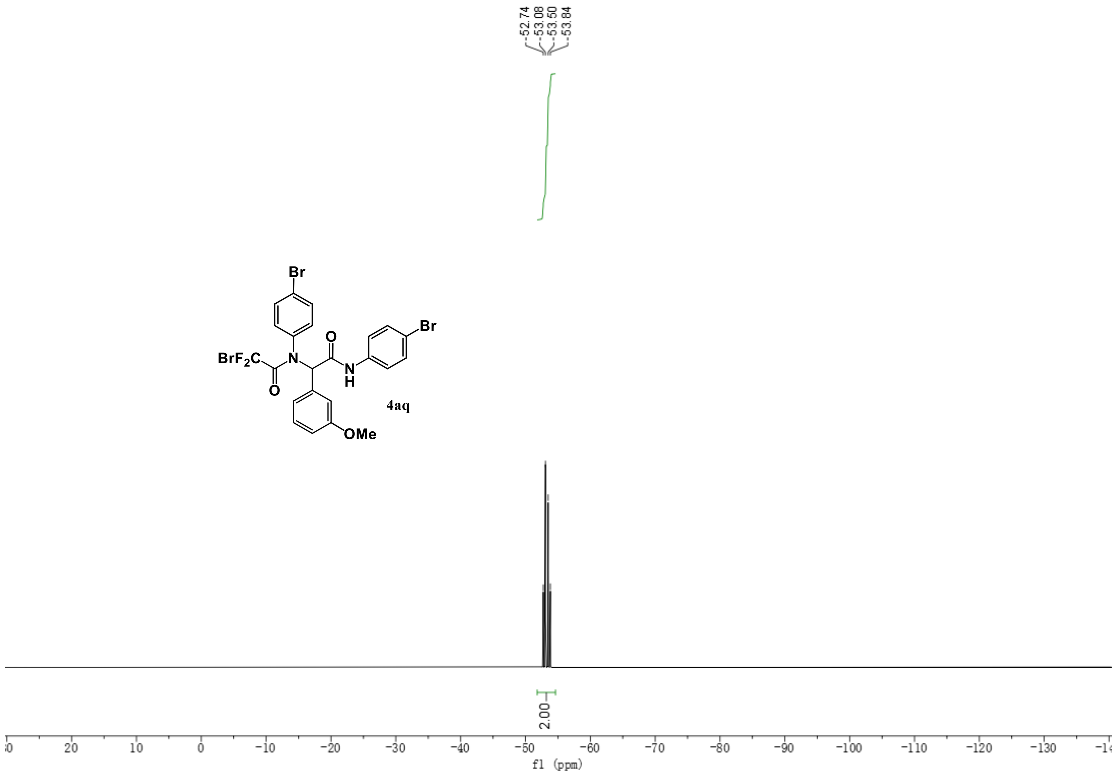

^{1}H NMR (500 MHz, DMSO), ^{13}C NMR (125 MHz, DMSO) and ^{19}F NMR (471 MHz, DMSO) spectra for 4ar

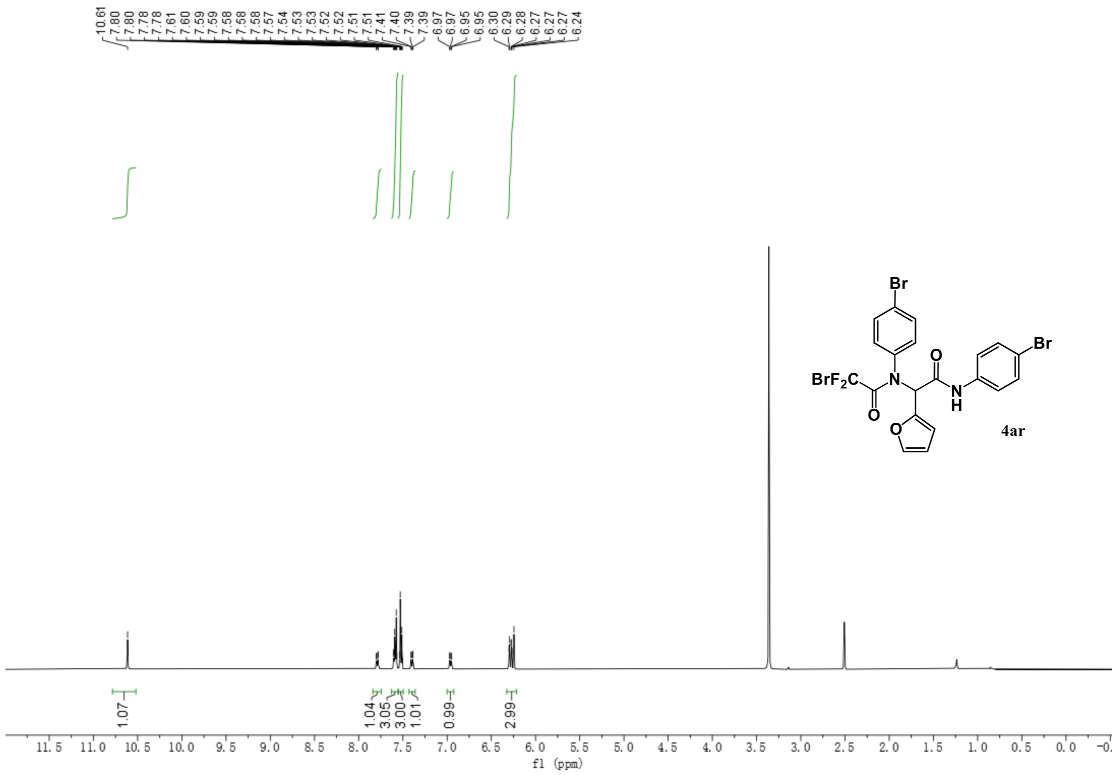

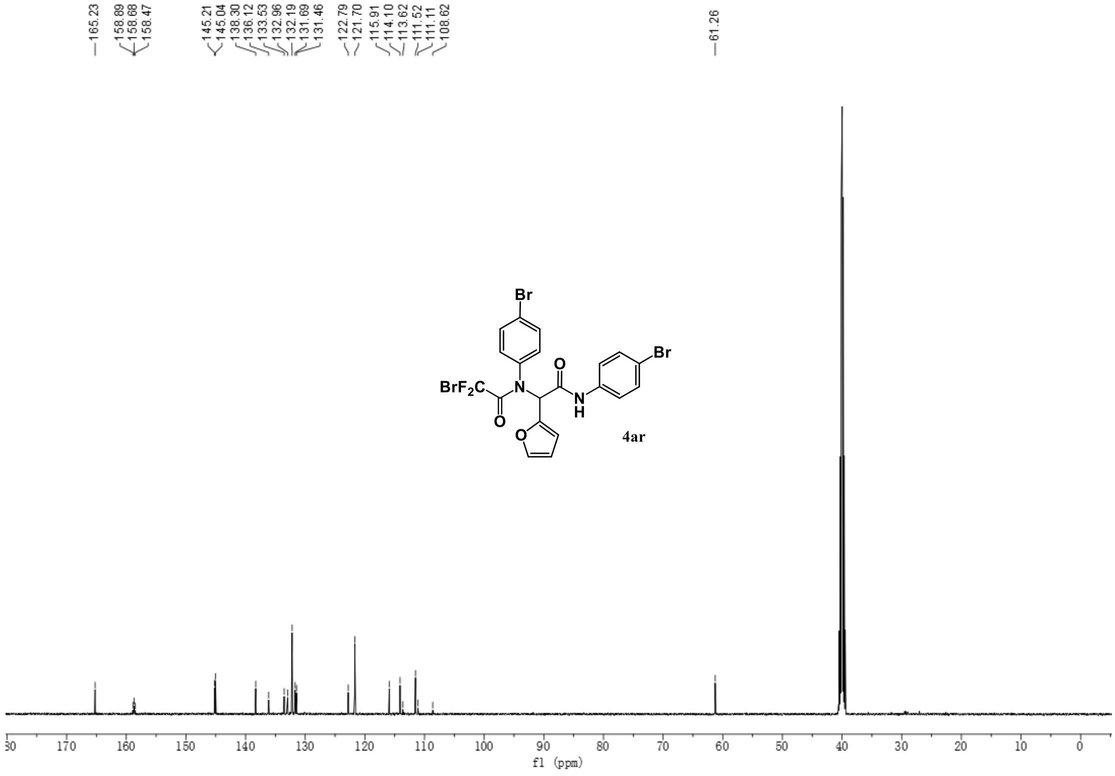
165.23
158.89
158.68
158.47
145.21
145.04
138.30
136.12
133.53
132.96
132.19
131.69
131.46
122.79
121.70
115.91
114.10
113.62
111.52
111.11
108.62
61.26
Br
O
Br
BrF2C
N
N
H
O
O
4ar
f1 (ppm)

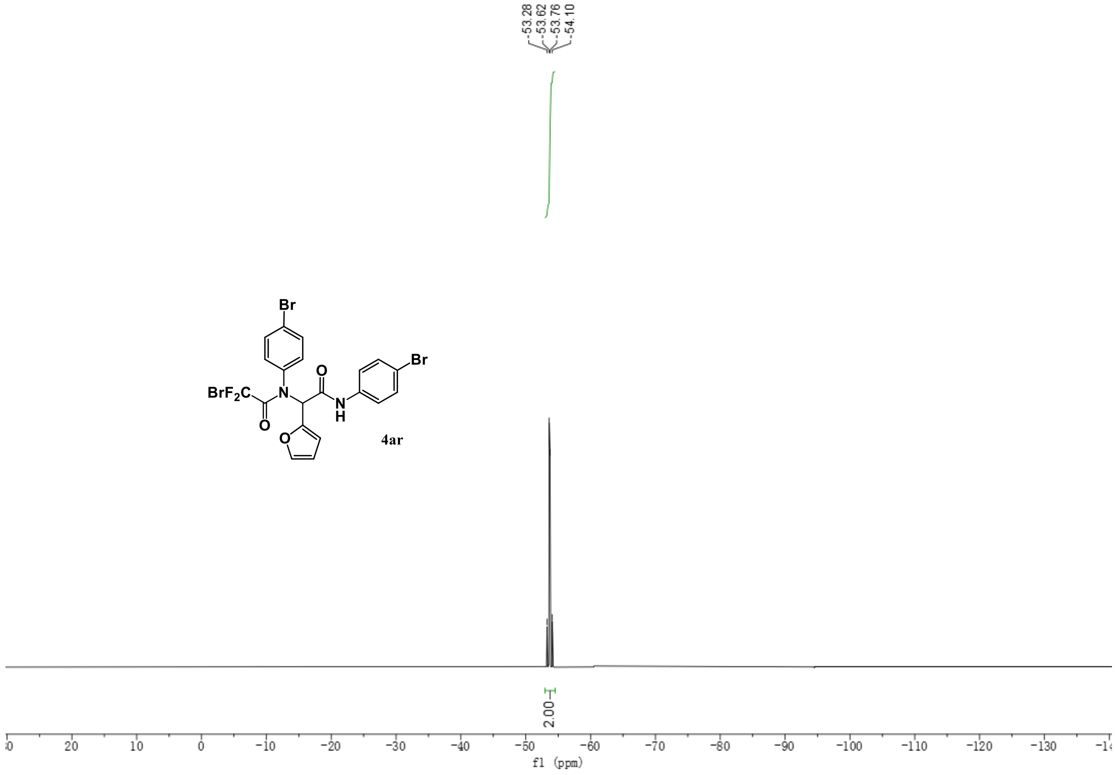
-53.28
-53.62
-53.76
-54.10
Br
O
Br
BrF2C
N
N
H
O
O
4ar
2.00
f1 (ppm)

^{1}H NMR (500 MHz, DMSO), ^{13}C NMR (125 MHz, DMSO) and ^{19}F NMR (471 MHz, DMSO) spectra for 4as

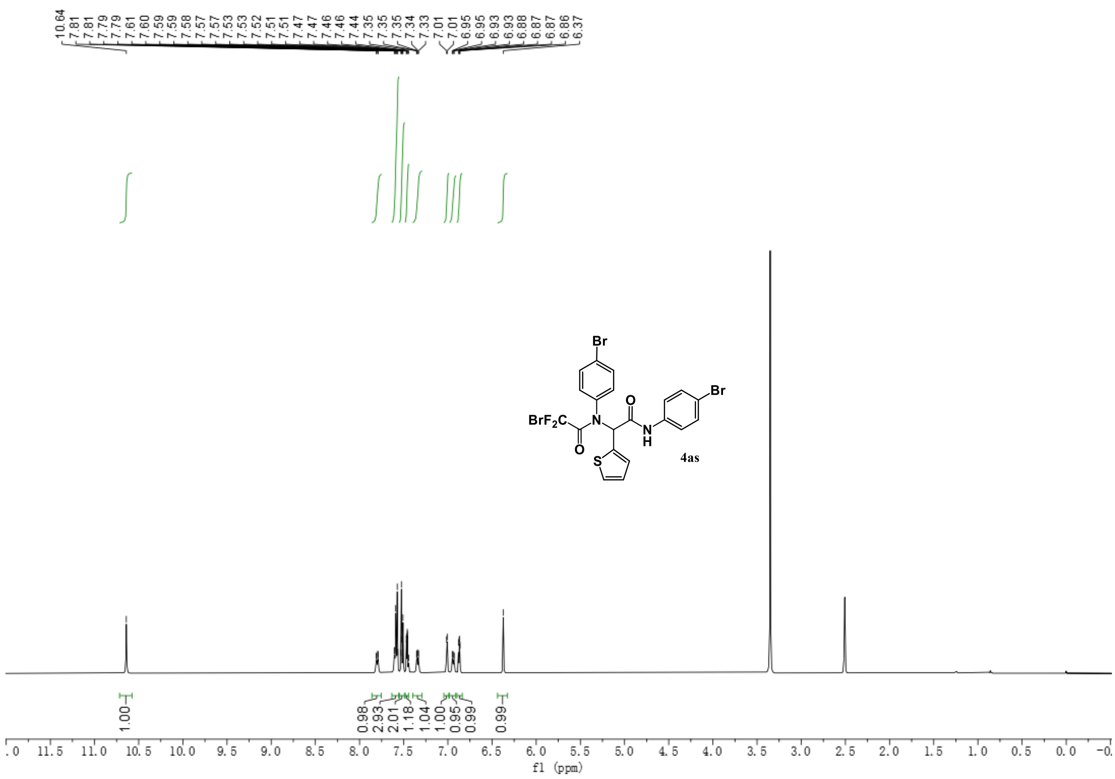

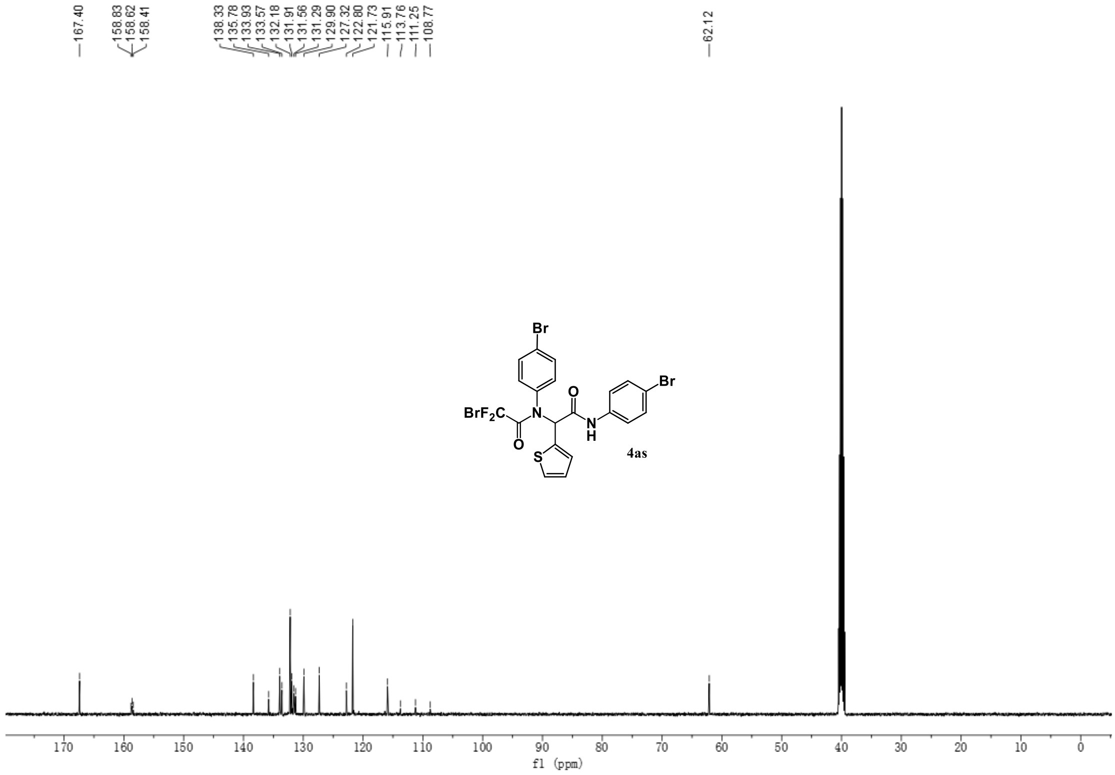

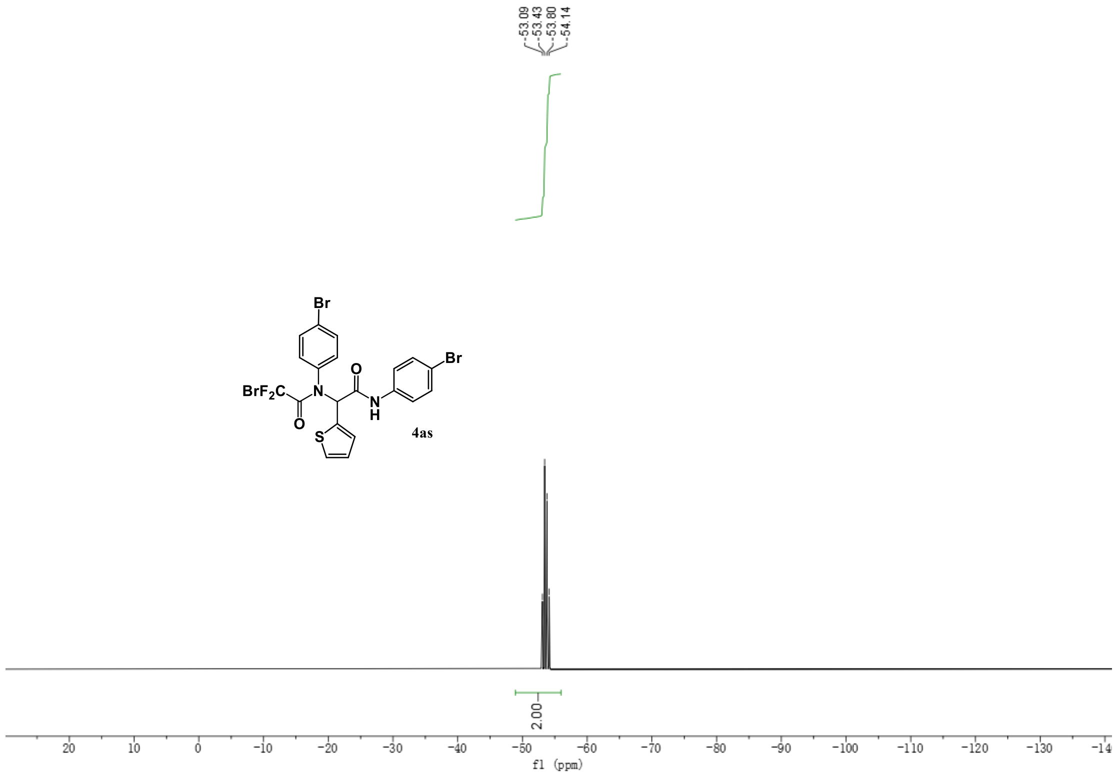

^{1}H NMR (500 MHz, DMSO), ^{13}C NMR (125 MHz, DMSO) and ^{19}F NMR (471 MHz, DMSO) spectra for 4at

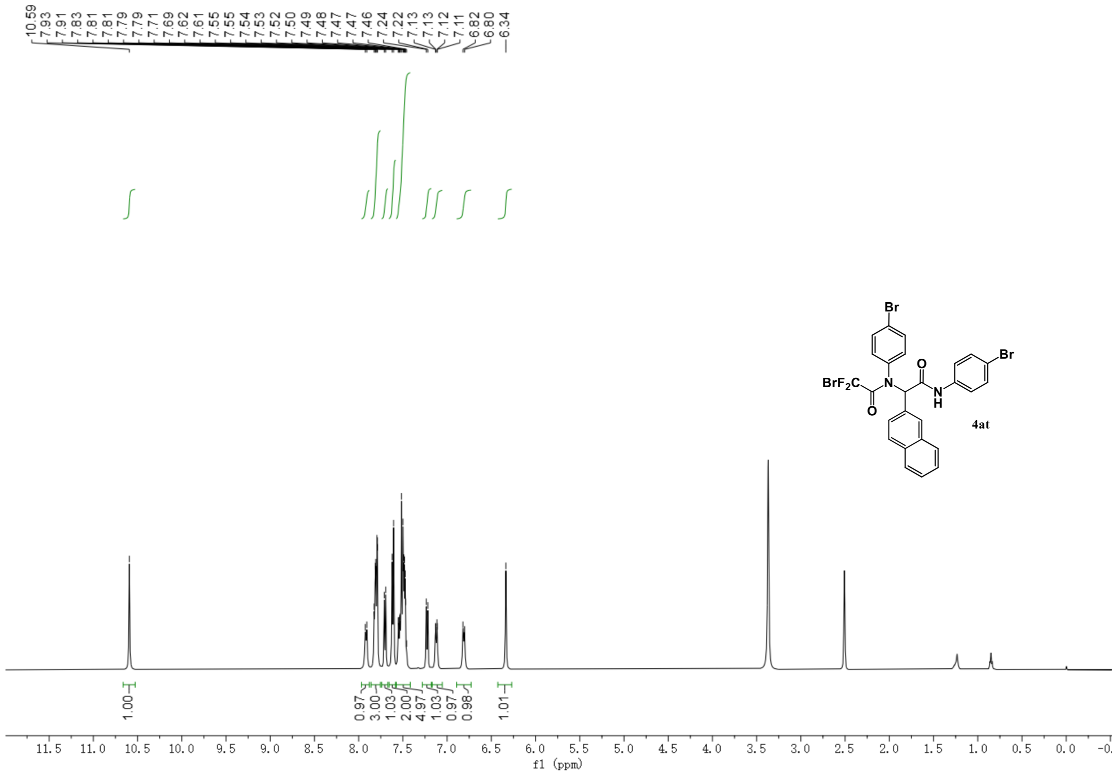

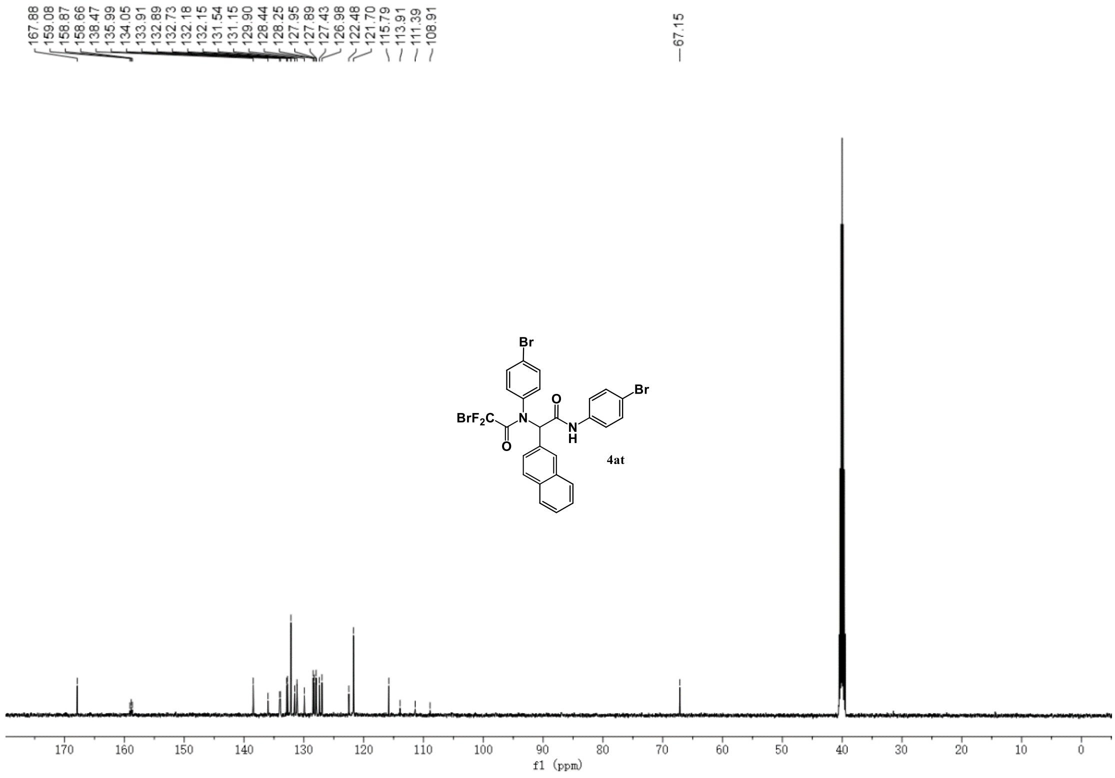
167.88
159.08
158.87
158.66
158.47
135.99
134.05
133.91
132.89
132.73
132.18
132.15
131.54
131.15
129.90
128.44
128.25
127.95
127.89
127.43
126.98
122.48
121.70
115.79
113.91
111.39
108.91
67.15
Br
Br
BrF2C
N
O
O
N
H
4at
f1 (ppm)

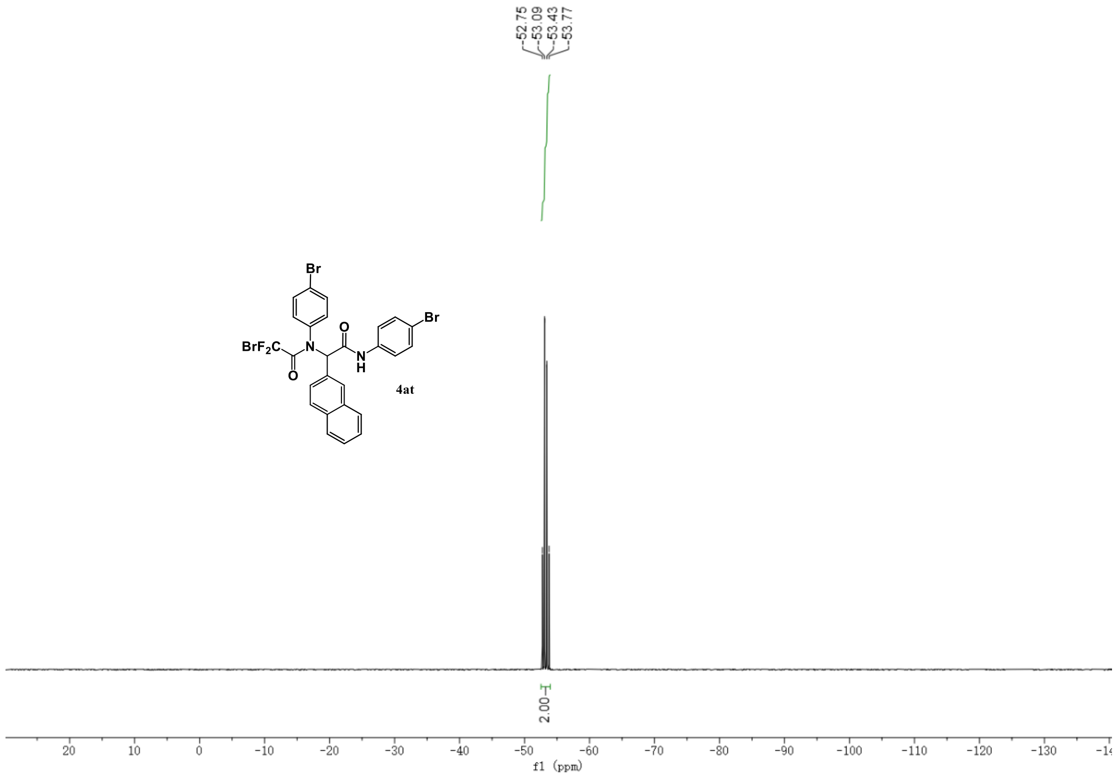
-52.75
-53.09
-53.43
-53.77
Br
Br
BrF2C
N
O
O
N
H
4at
2.00
f1 (ppm)

^{1}H NMR (500 MHz, DMSO), ^{13}C NMR (125 MHz, DMSO) and ^{19}F NMR (471 MHz, DMSO) spectra for 4au

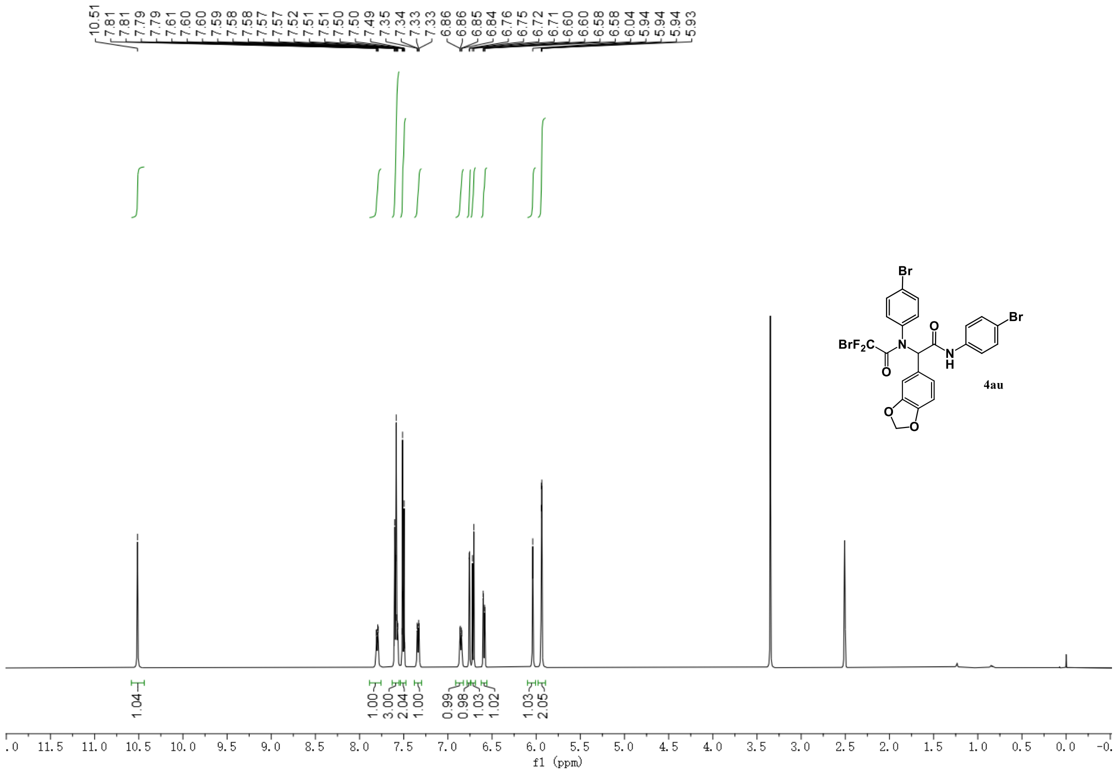

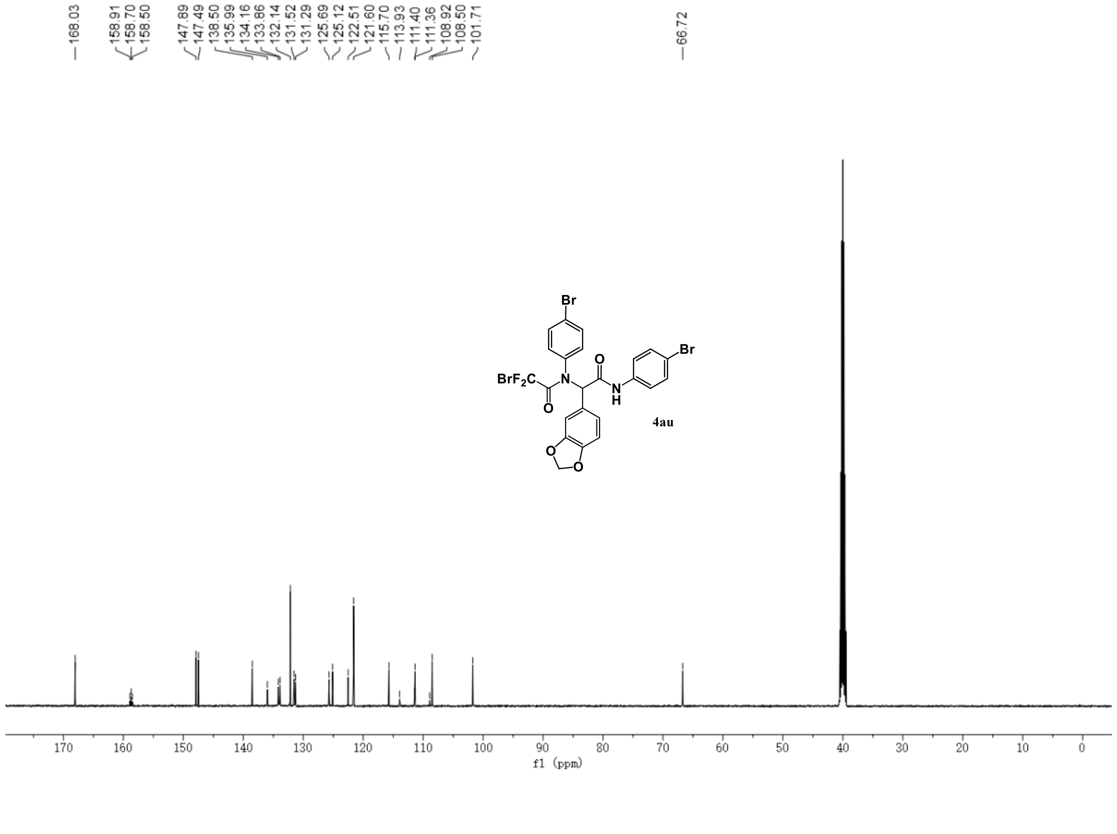

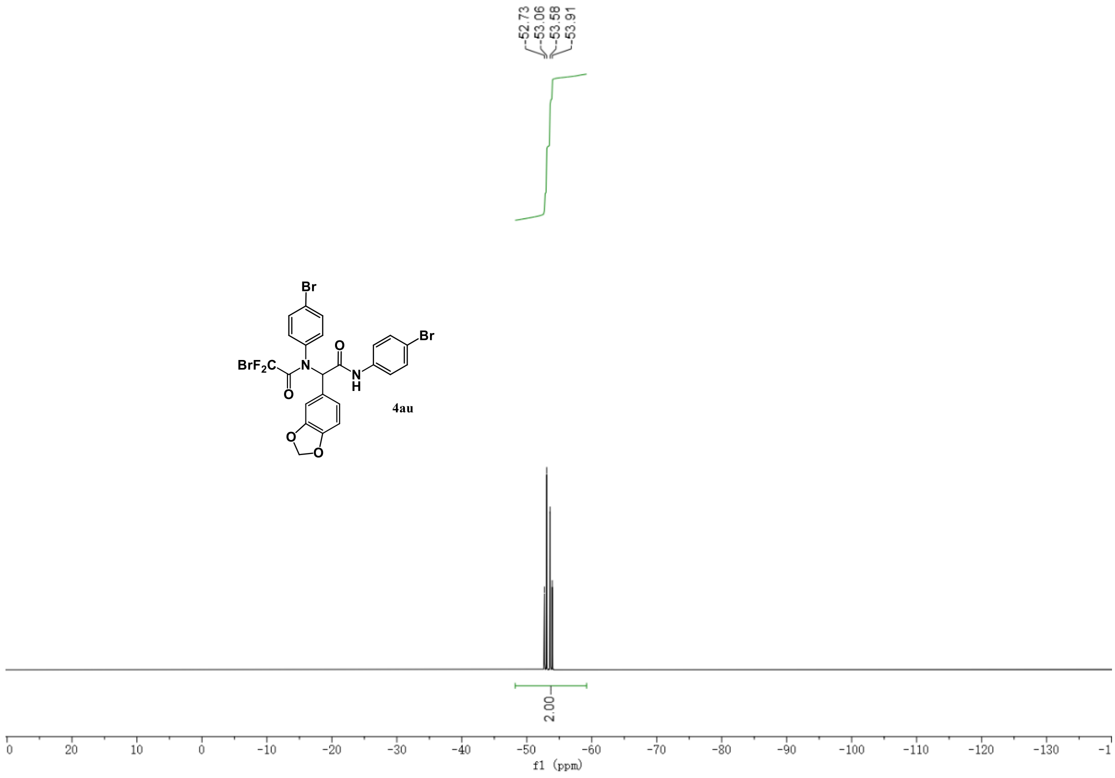

^{1}H NMR (500 MHz, DMSO), ^{13}C NMR (125 MHz, DMSO) and ^{19}F NMR (471 MHz, DMSO) spectra for 4av

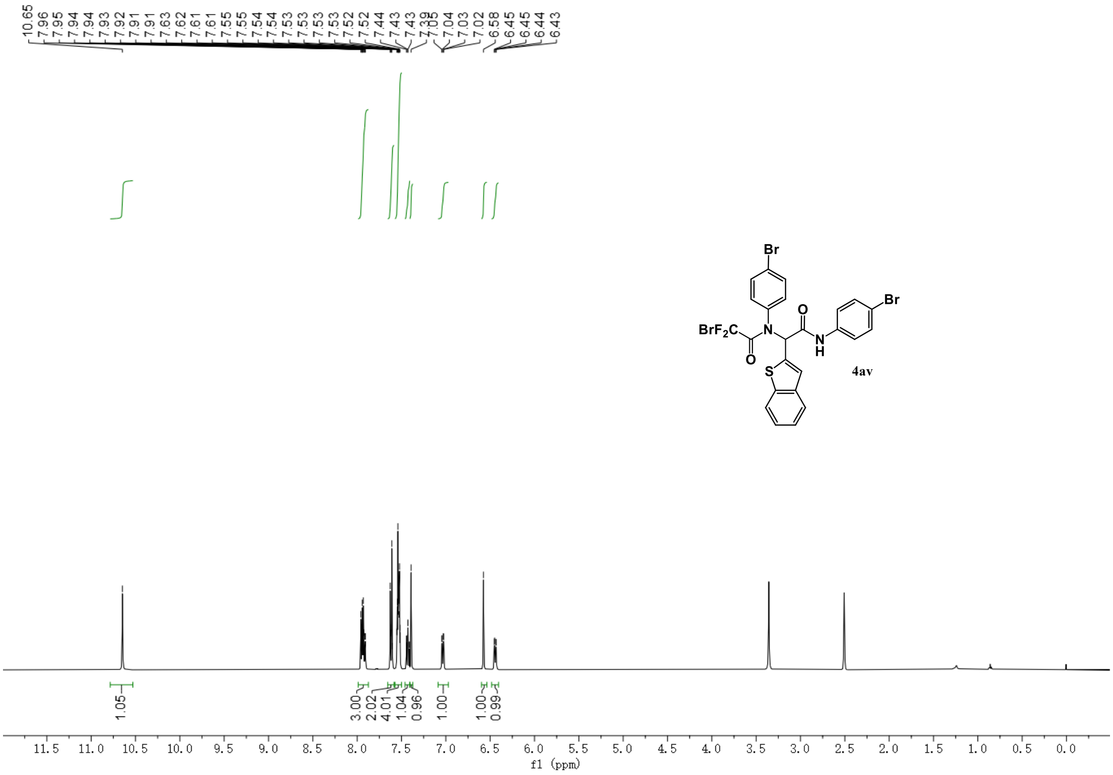

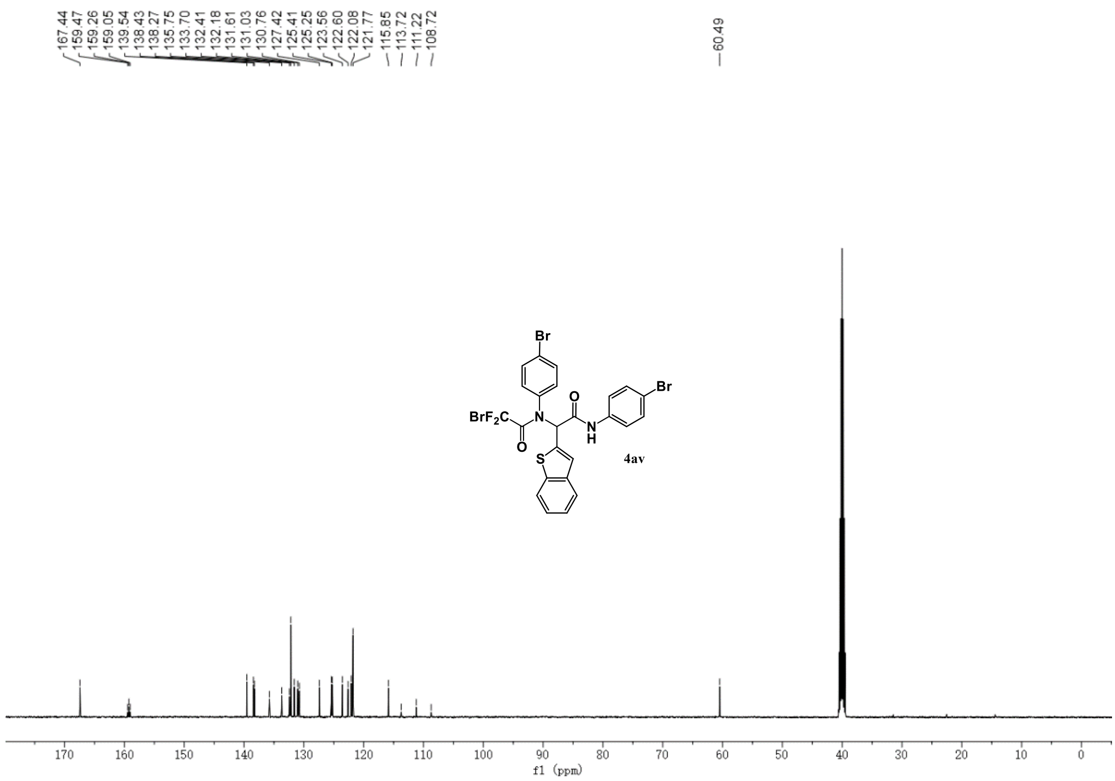
167.44
159.47
159.26
159.05
139.54
138.43
138.27
135.75
133.70
132.41
132.18
131.61
131.03
130.76
127.42
125.41
125.25
123.56
122.60
122.08
121.77
115.85
113.72
111.22
108.72
60.49
Br
Br
BrF_2C
N
O
O
N
H
S
4av
f1 (ppm)

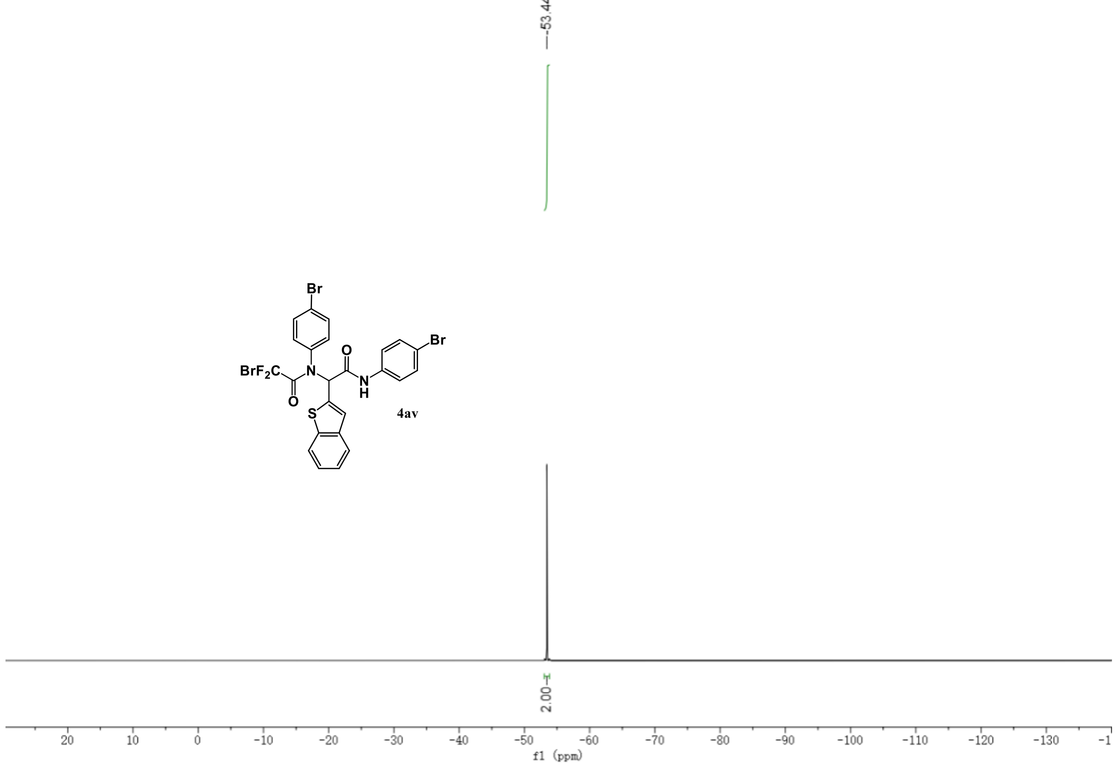
-53.44
Br
Br
BrF_2C
N
O
O
N
H
S
4av
2.00
f1 (ppm)

^{1}H NMR (500 MHz, DMSO), ^{13}C NMR (125 MHz, DMSO) and ^{19}F NMR (471 MHz, DMSO) spectra for 4aw

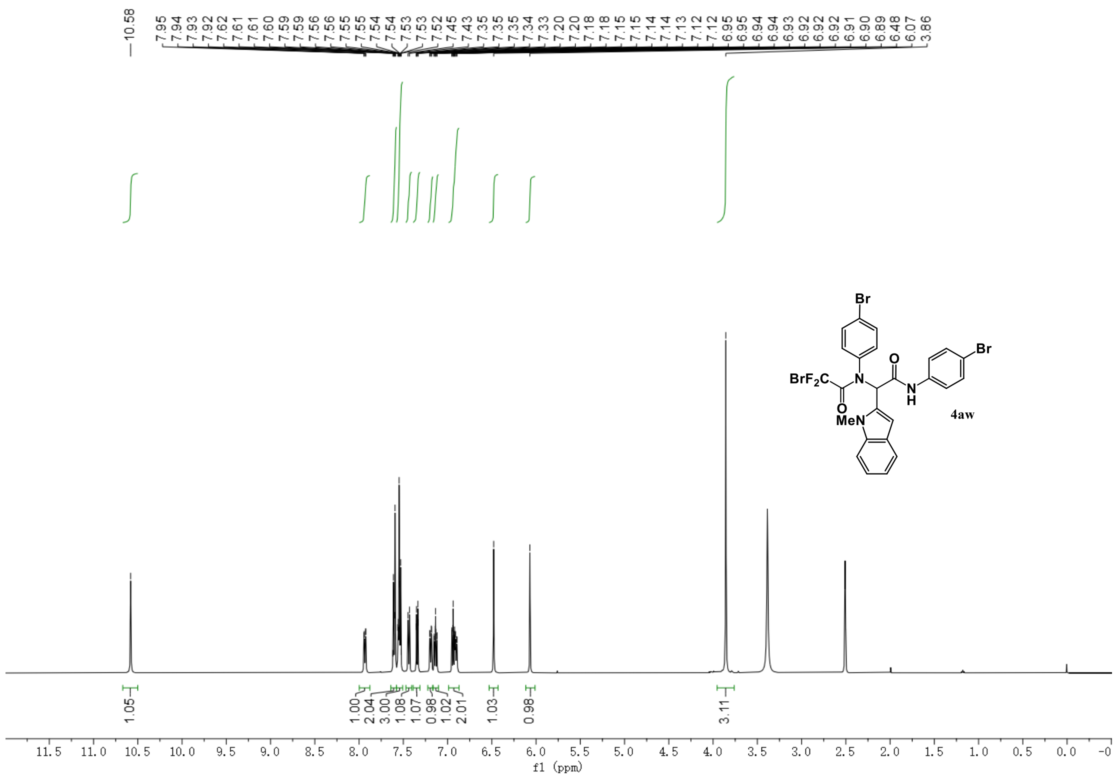

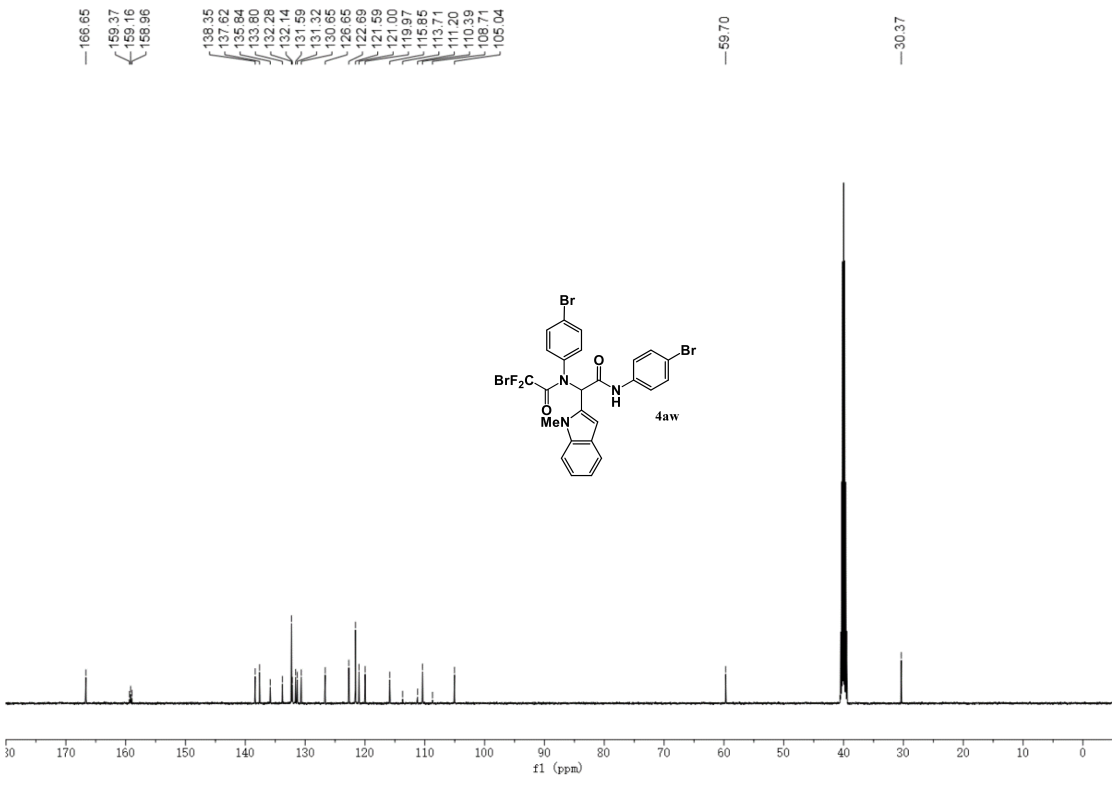

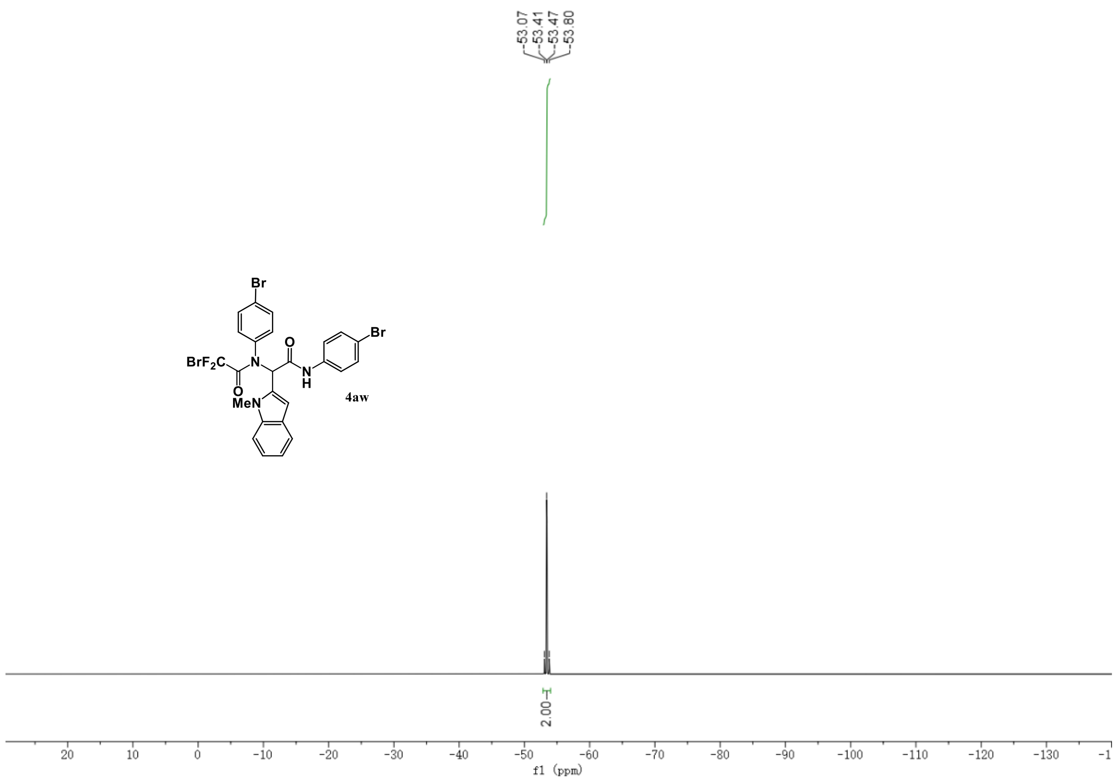

^{1}H NMR (500 MHz, DMSO), ^{13}C NMR (125 MHz, DMSO) and ^{19}F NMR (471 MHz, DMSO) spectra for 4ax

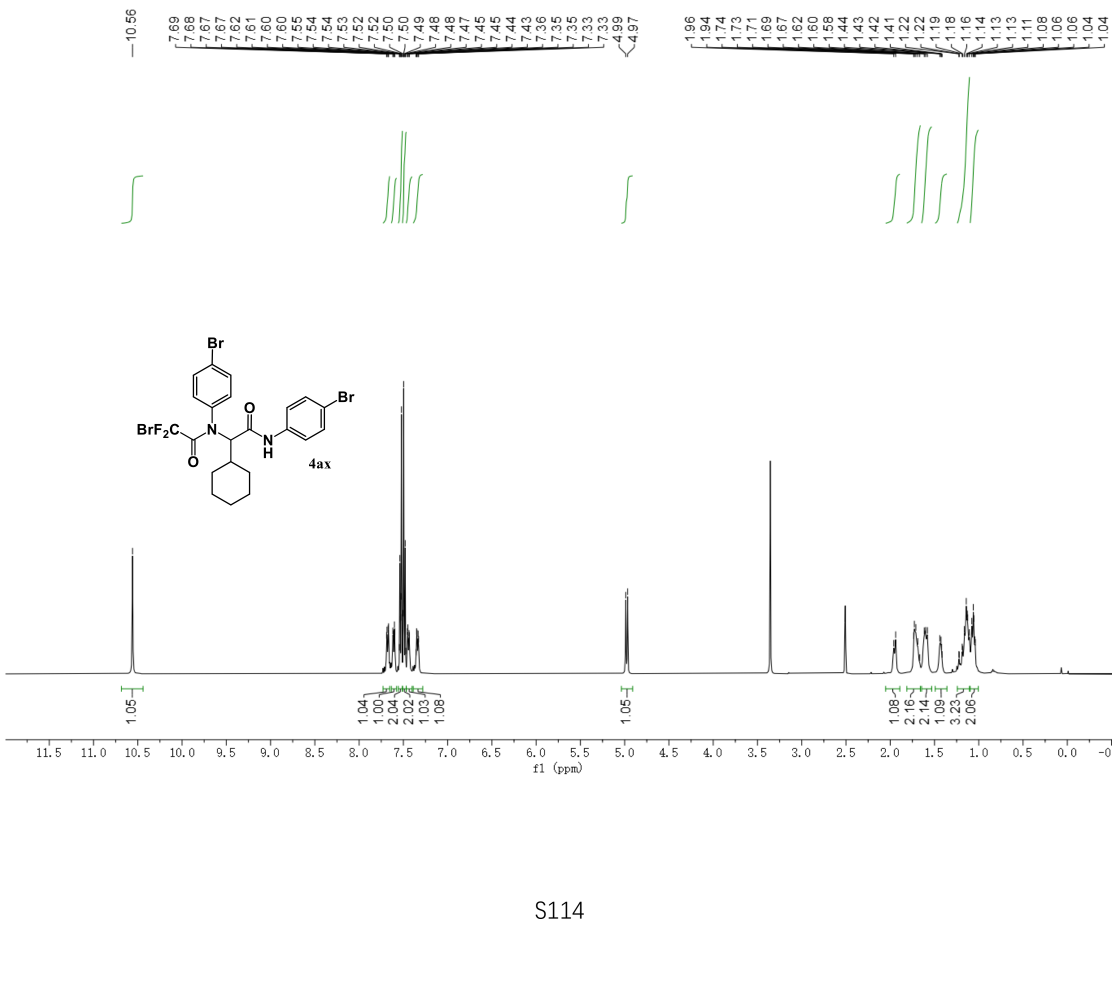

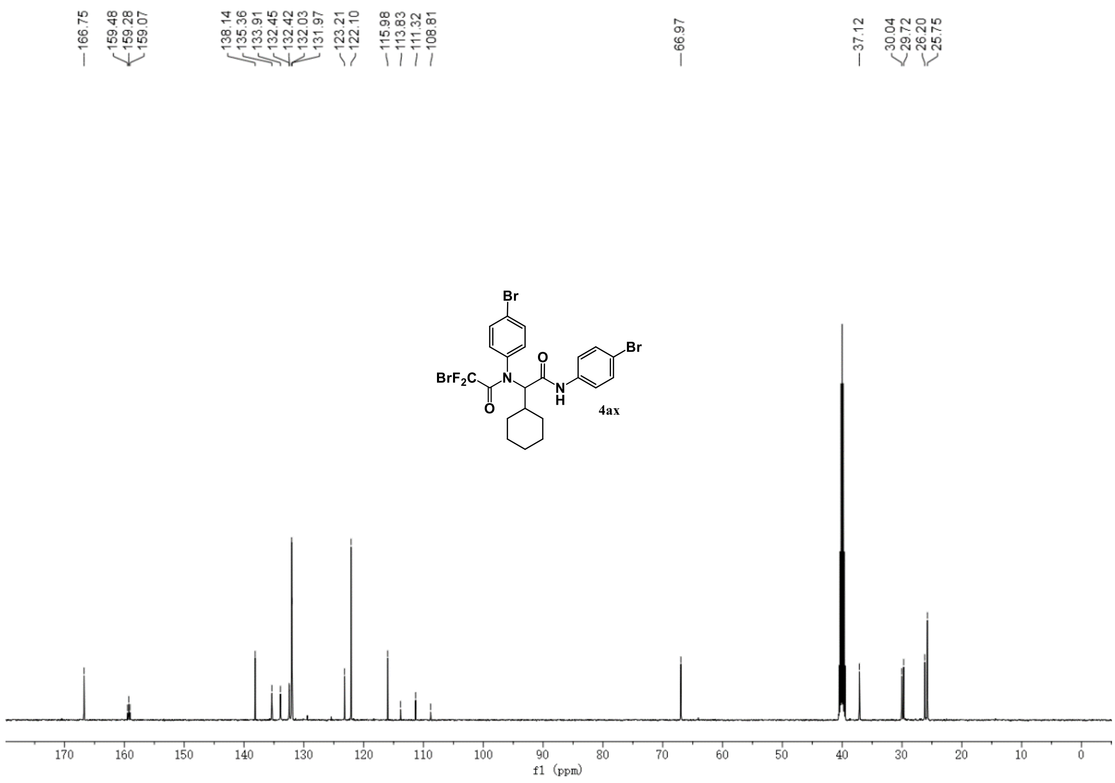
166.75
159.48
159.28
159.07
138.14
135.36
133.91
132.45
132.42
132.03
131.97
123.21
122.10
115.98
113.83
111.32
108.81
66.97
37.12
30.04
29.72
26.20
25.75
Br
BrF2C
N
O
H
Br
4ax
f1 (ppm)

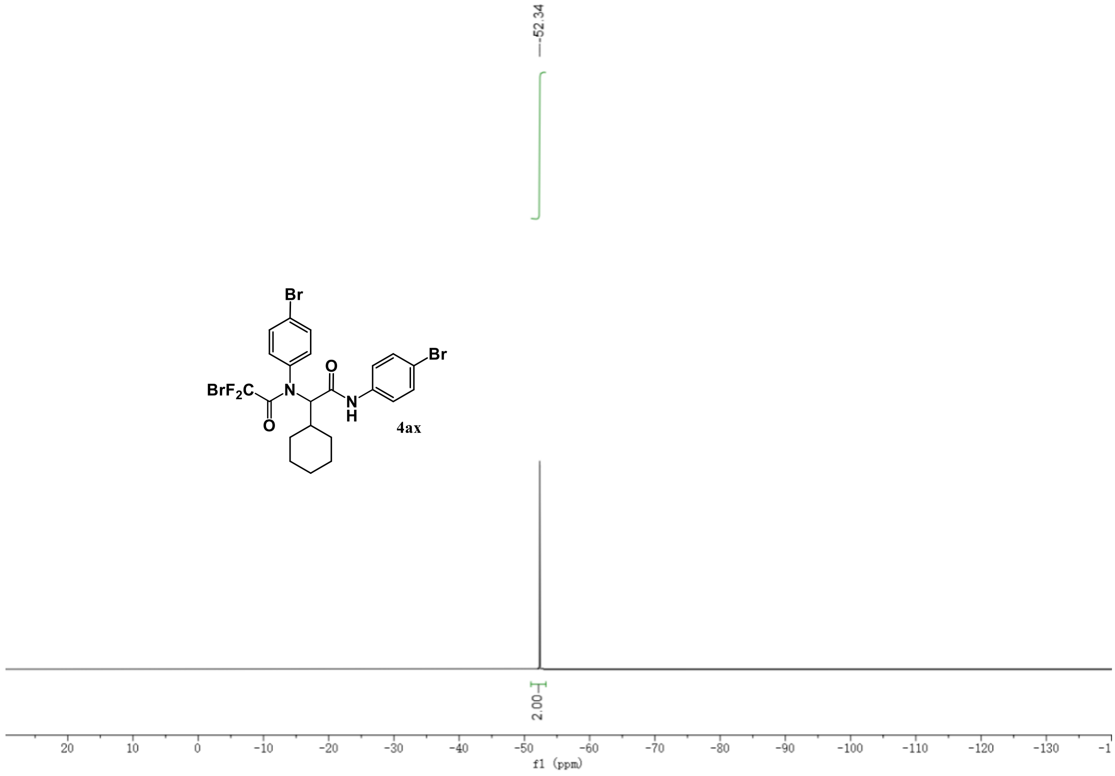
-52.34
Br
BrF2C
N
O
H
Br
4ax
2.00
f1 (ppm)

^{1}H NMR (500 MHz, DMSO), ^{13}C NMR (125 MHz, DMSO) and ^{19}F NMR (471 MHz, DMSO) spectra for 4ay

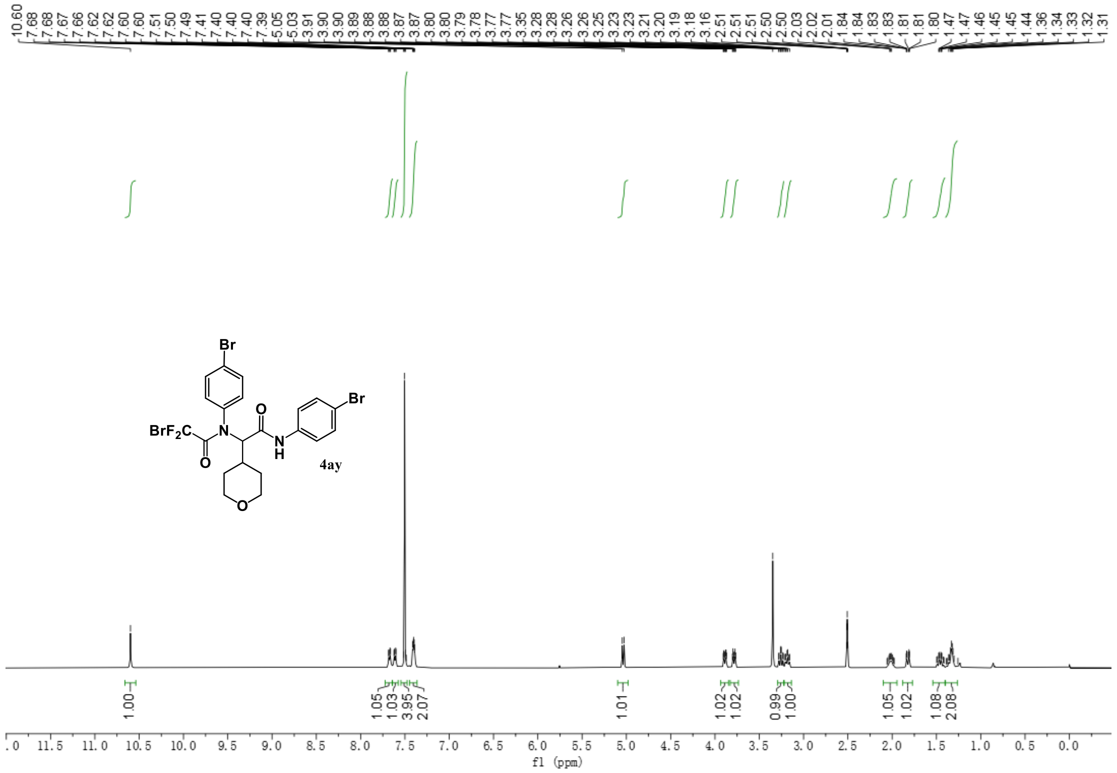

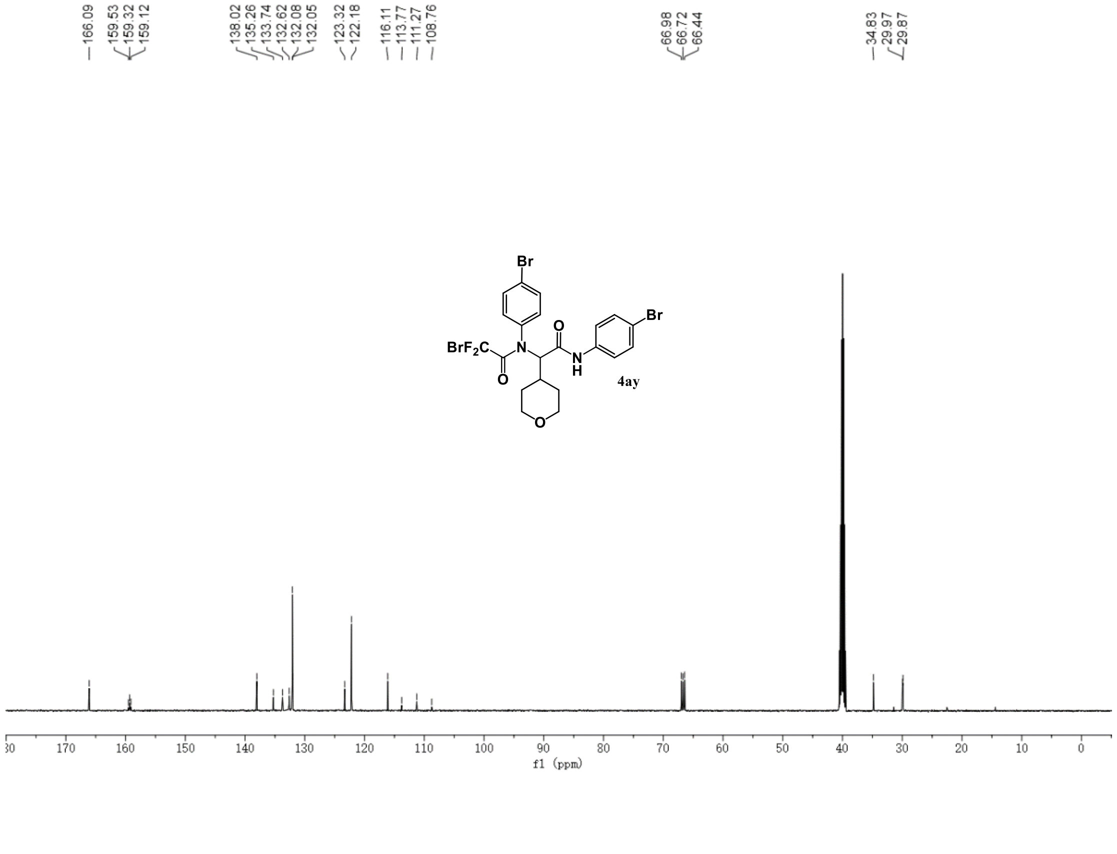

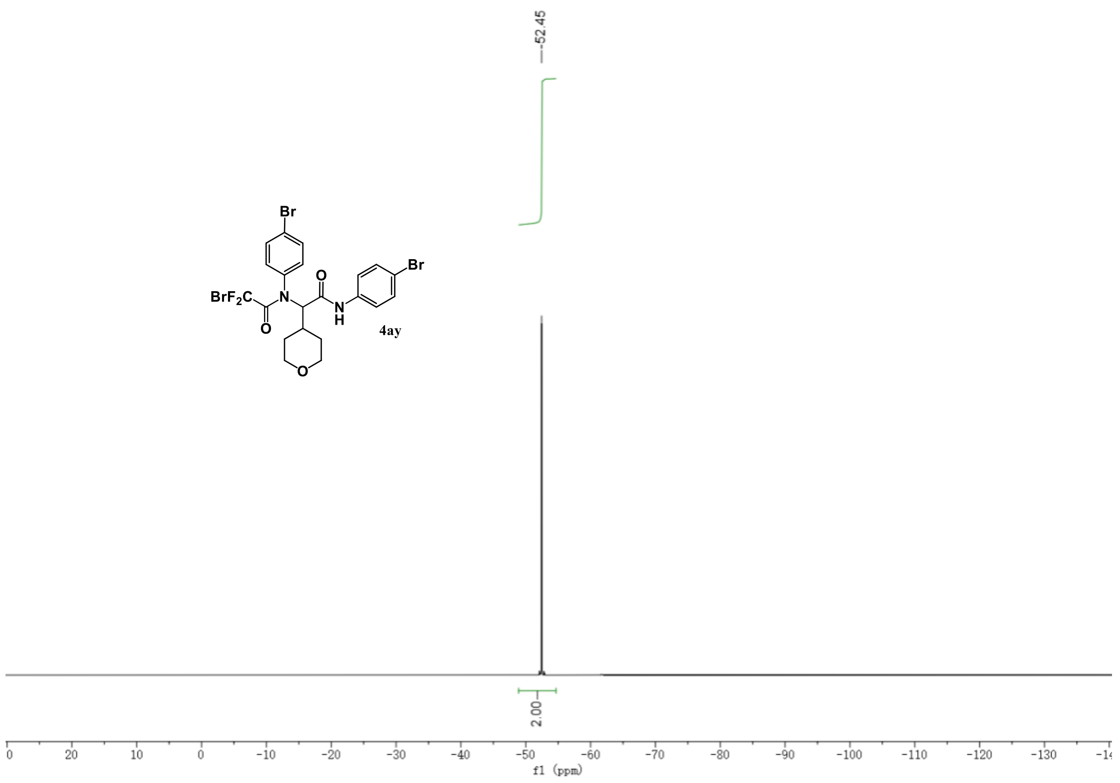

^{1}H NMR (500 MHz, DMSO), ^{13}C NMR (125 MHz, DMSO) and ^{19}F NMR (471 MHz, DMSO) spectra for 4az

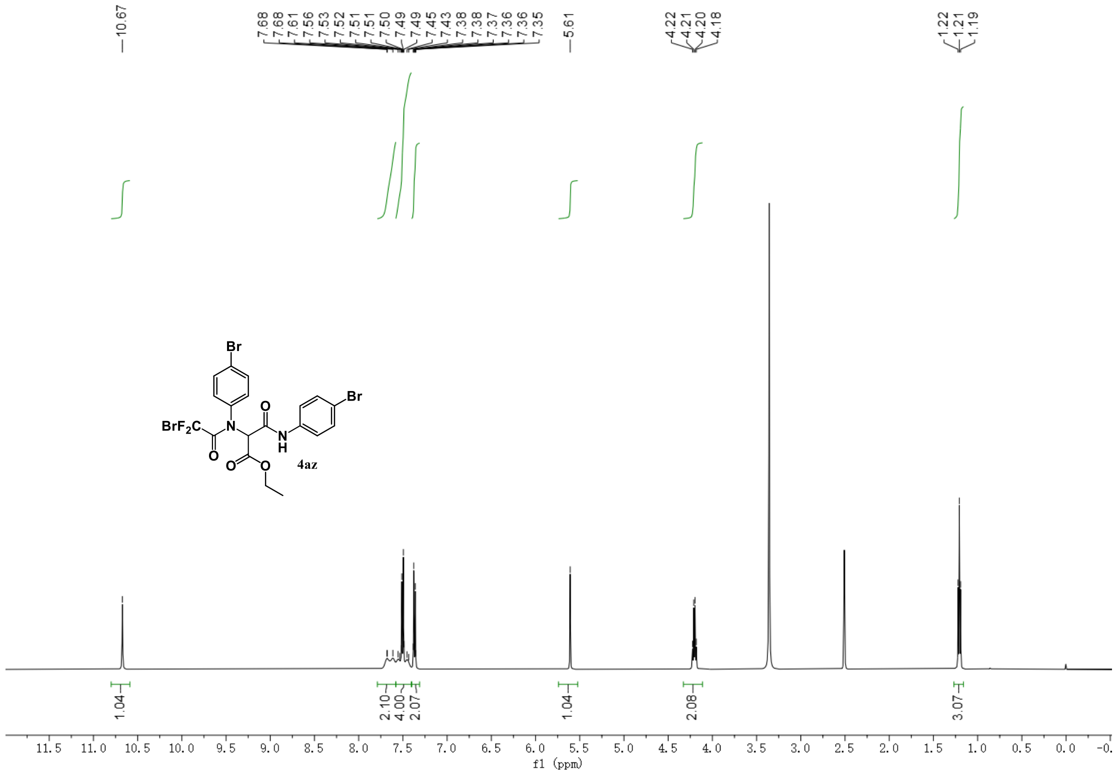

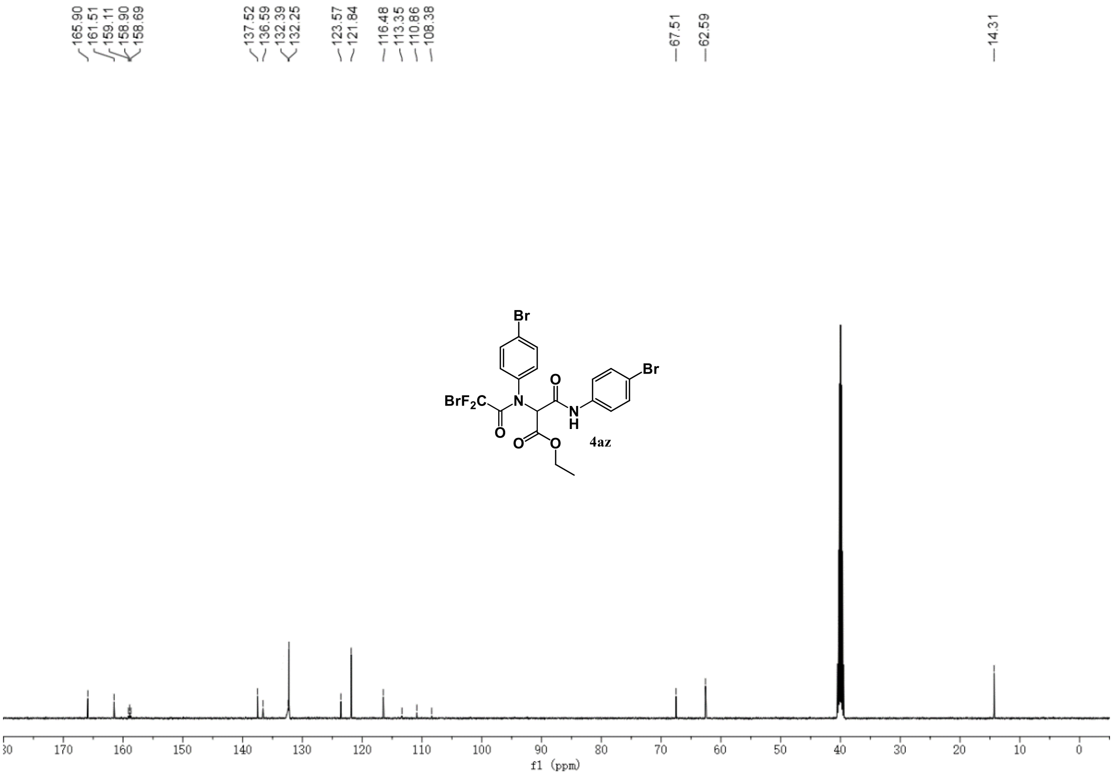
165.90
161.51
159.11
158.90
158.69
137.52
136.59
132.39
132.25
123.57
121.84
116.48
113.35
110.86
108.38
67.51
62.59
14.31
Br
Br
BrF2C
N
O
H
O
O
4az
f1 (ppm)

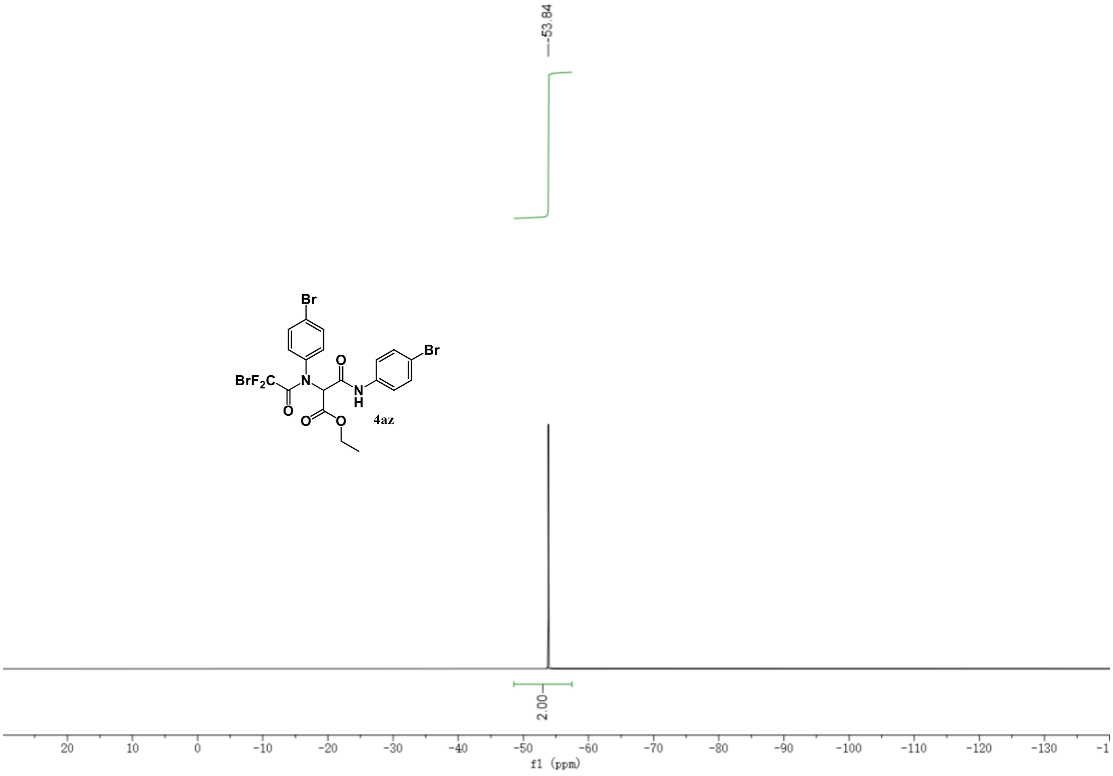
-53.84
Br
Br
BrF2C
N
O
H
O
O
4az
2.00
f1 (ppm)

^{1}H NMR (500 MHz, DMSO), ^{13}C NMR (125 MHz, DMSO) and ^{19}F NMR (471 MHz, DMSO) spectra for 4A

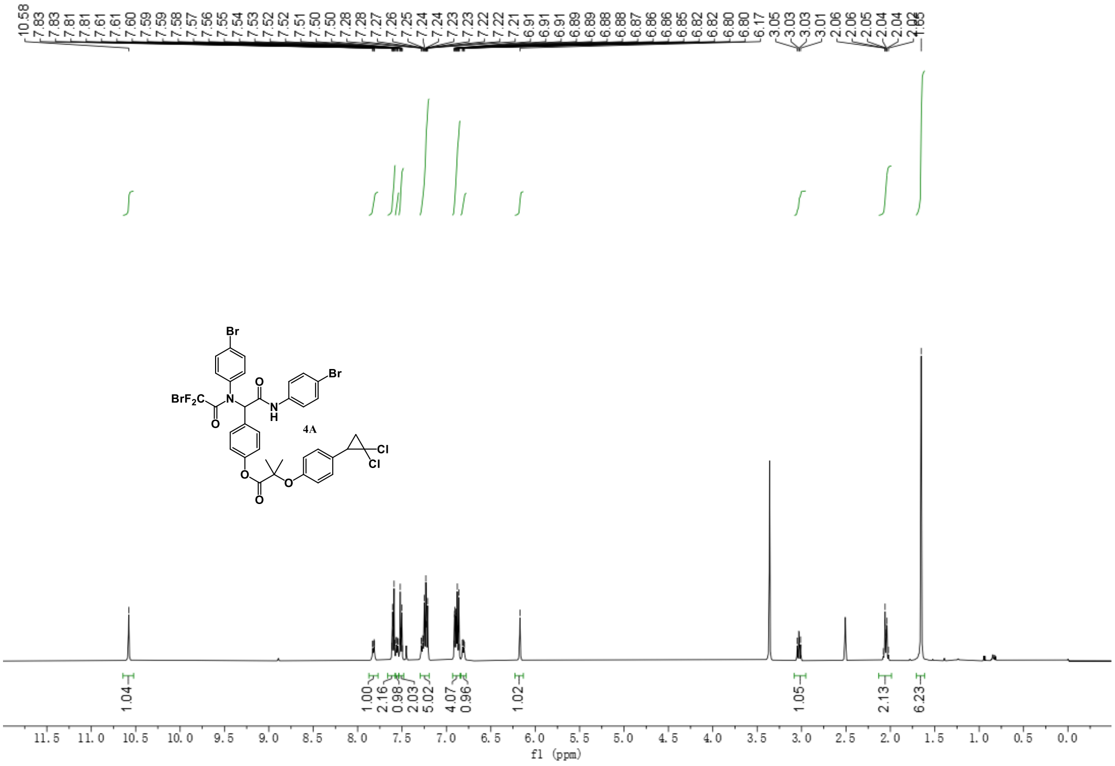

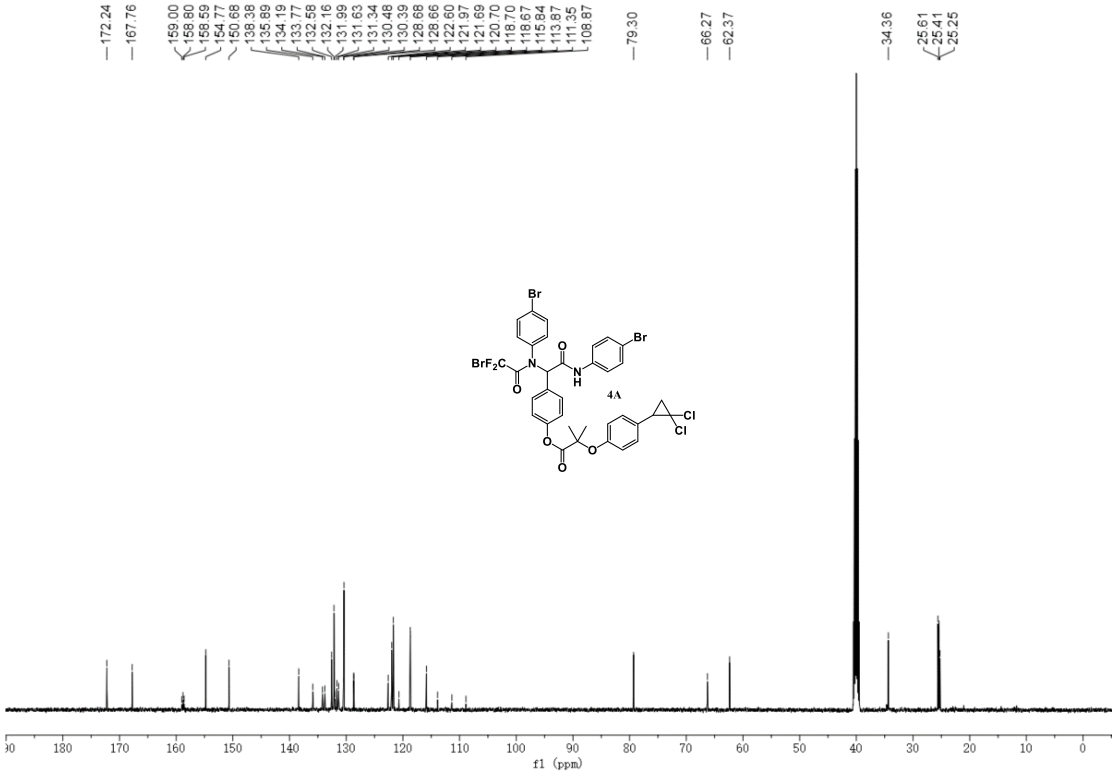

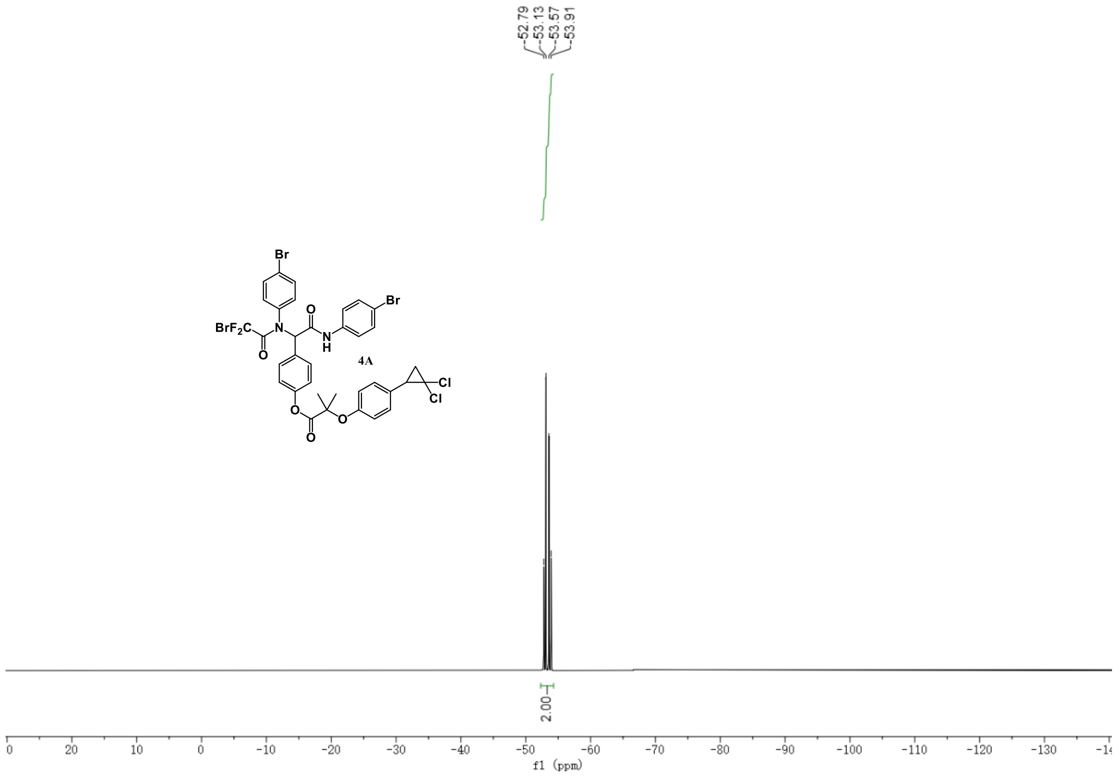

^{1}H NMR (500 MHz, DMSO), ^{13}C NMR (125 MHz, DMSO) and ^{19}F NMR (471 MHz, DMSO) spectra for 4B

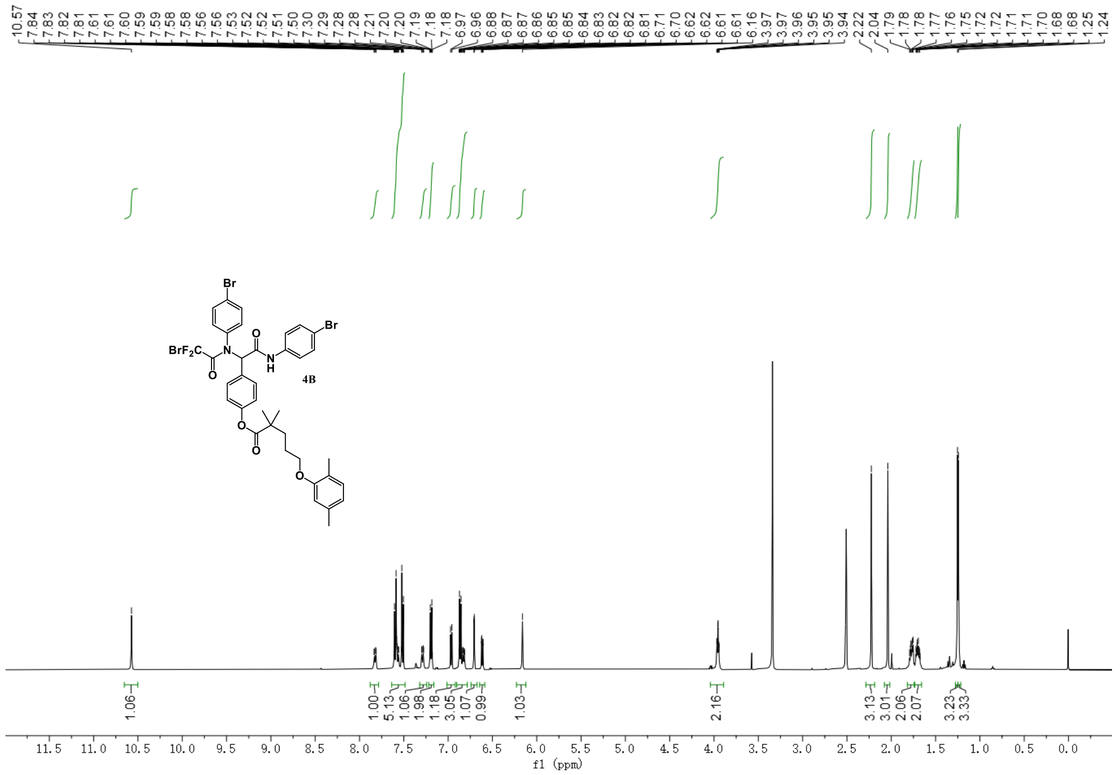

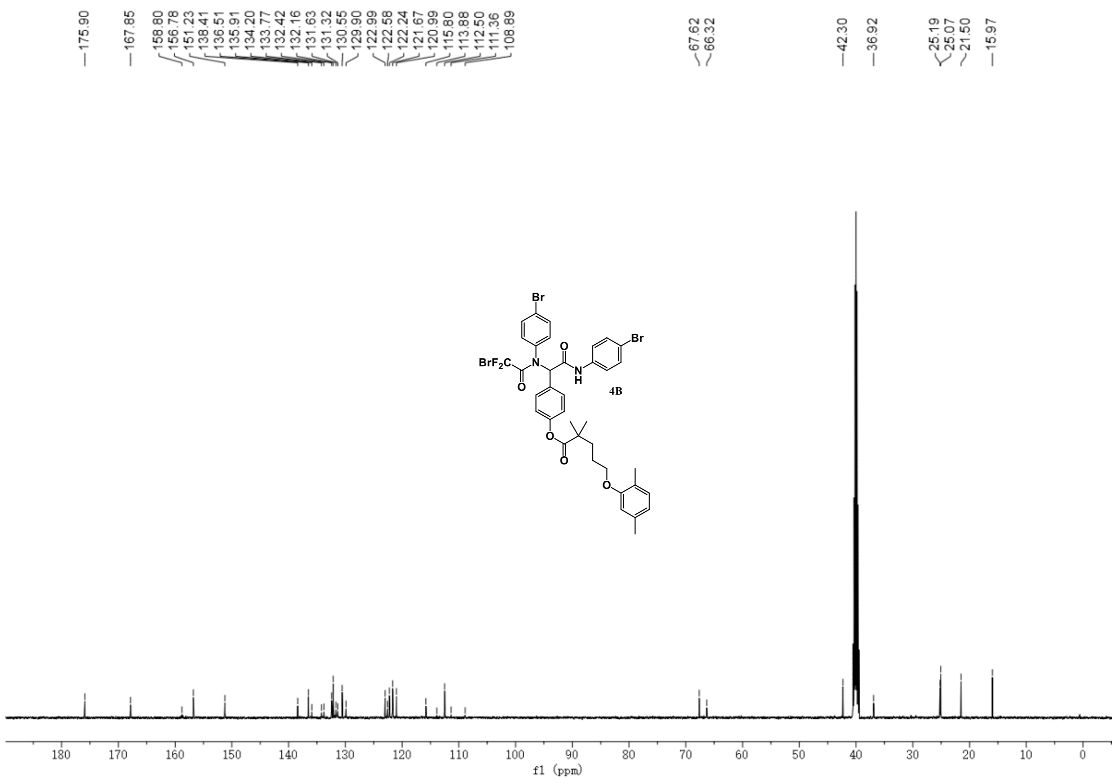
175.90
167.85
158.80
156.78
151.23
138.41
136.51
135.91
134.20
133.77
132.42
132.16
131.63
131.32
130.55
129.90
122.99
122.58
122.24
121.67
120.99
115.80
113.88
112.50
111.36
108.89
67.62
66.32
42.30
36.92
25.19
25.07
21.50
15.97
Br
BrF2C
N
O
H
4B
f1 (ppm)

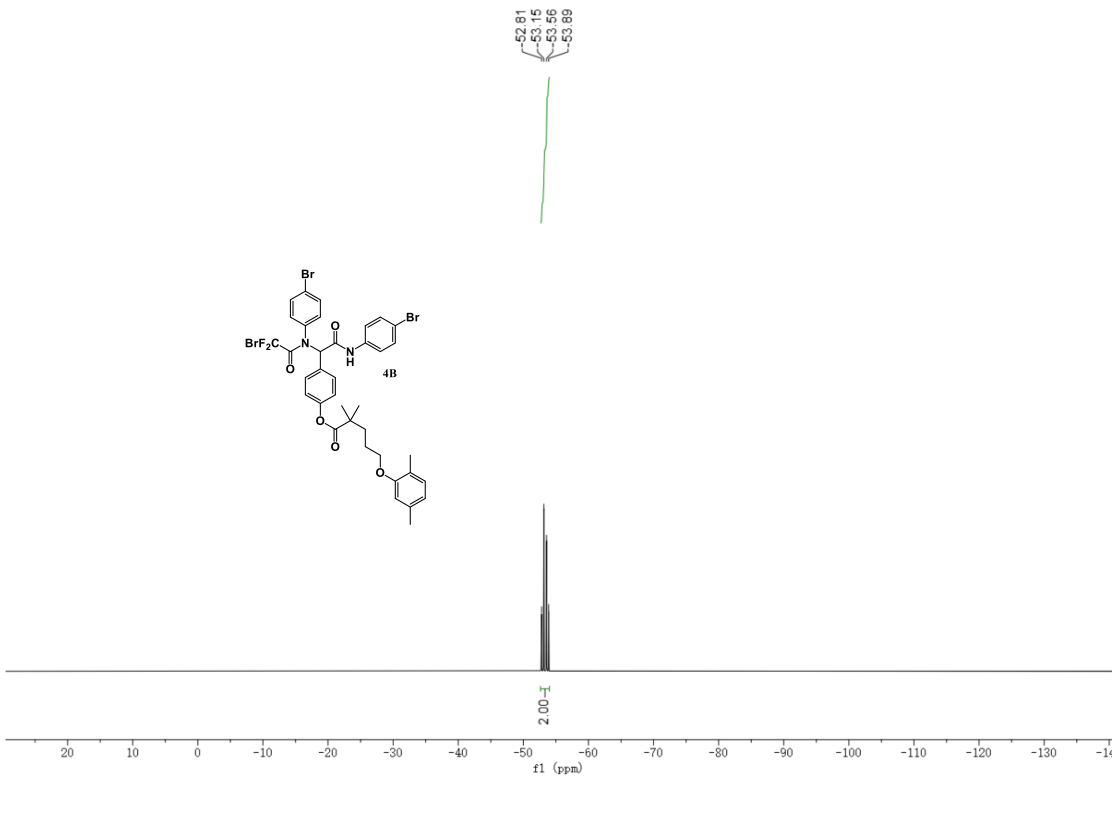
52.81
53.15
53.56
53.89
Br
BrF2C
N
O
H
4B
2.00
f1 (ppm)

^{1}H NMR (500 MHz, DMSO) and ^{13}C NMR (125 MHz, DMSO) spectra for 5

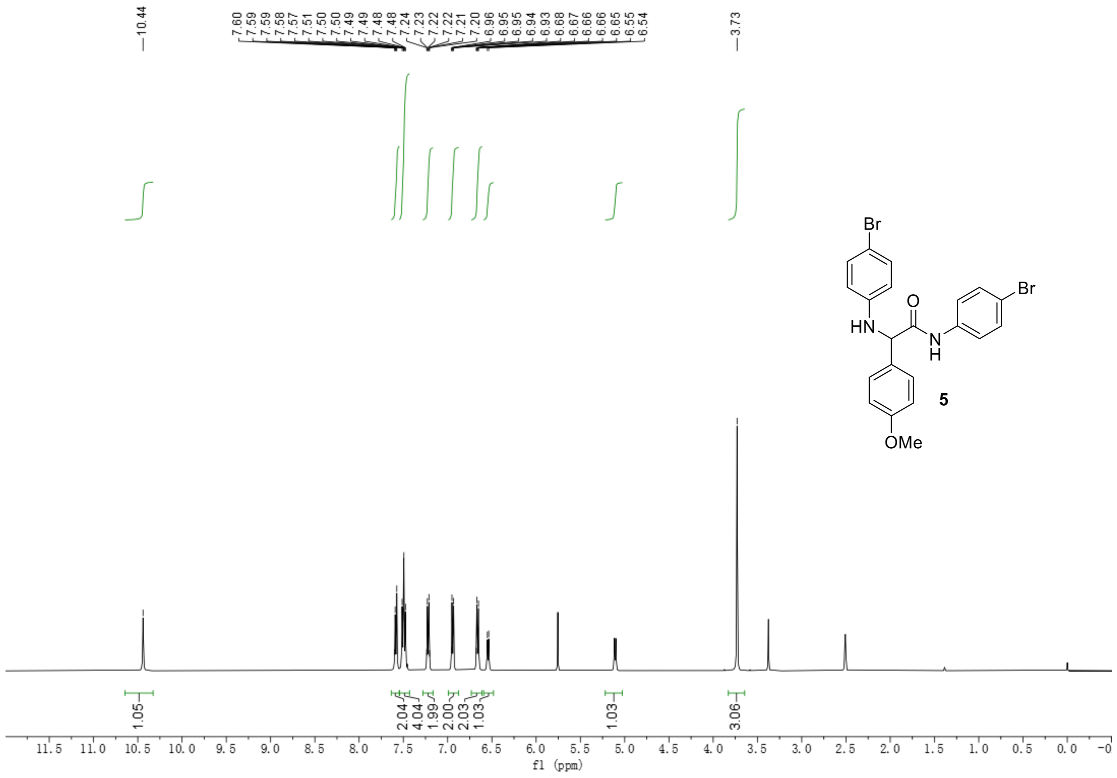

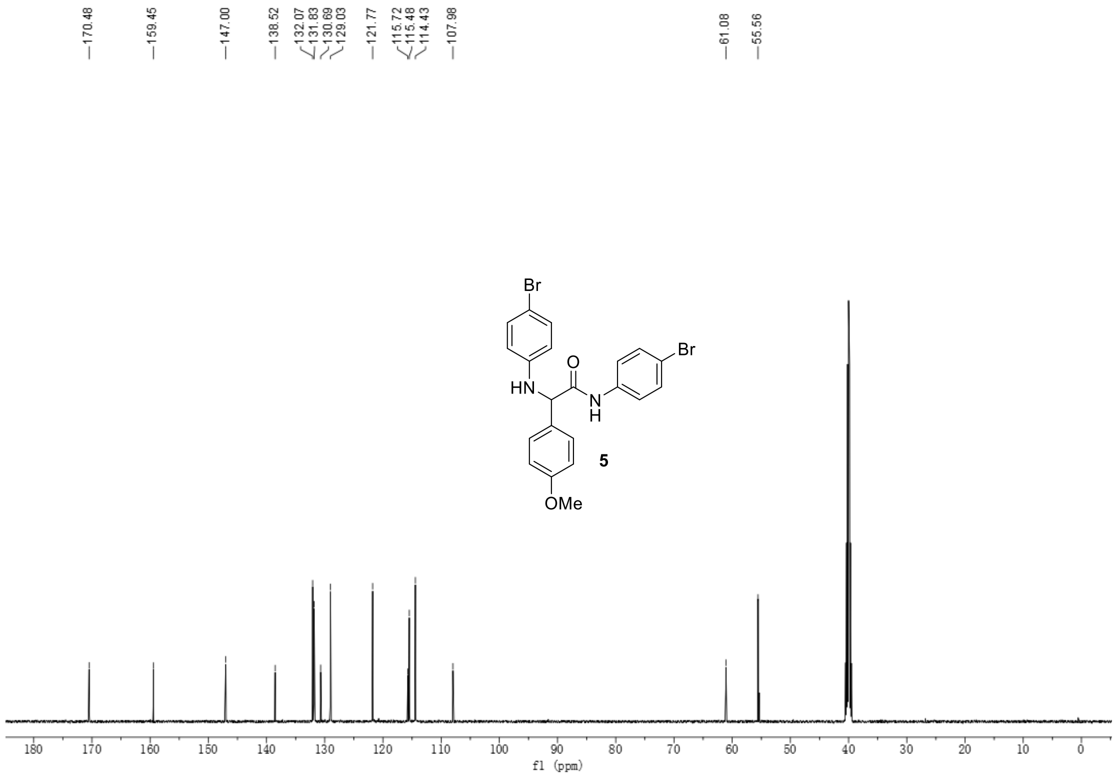

^{1}H NMR (500 MHz, DMSO) and ^{13}C NMR (125 MHz, DMSO) spectra for 6

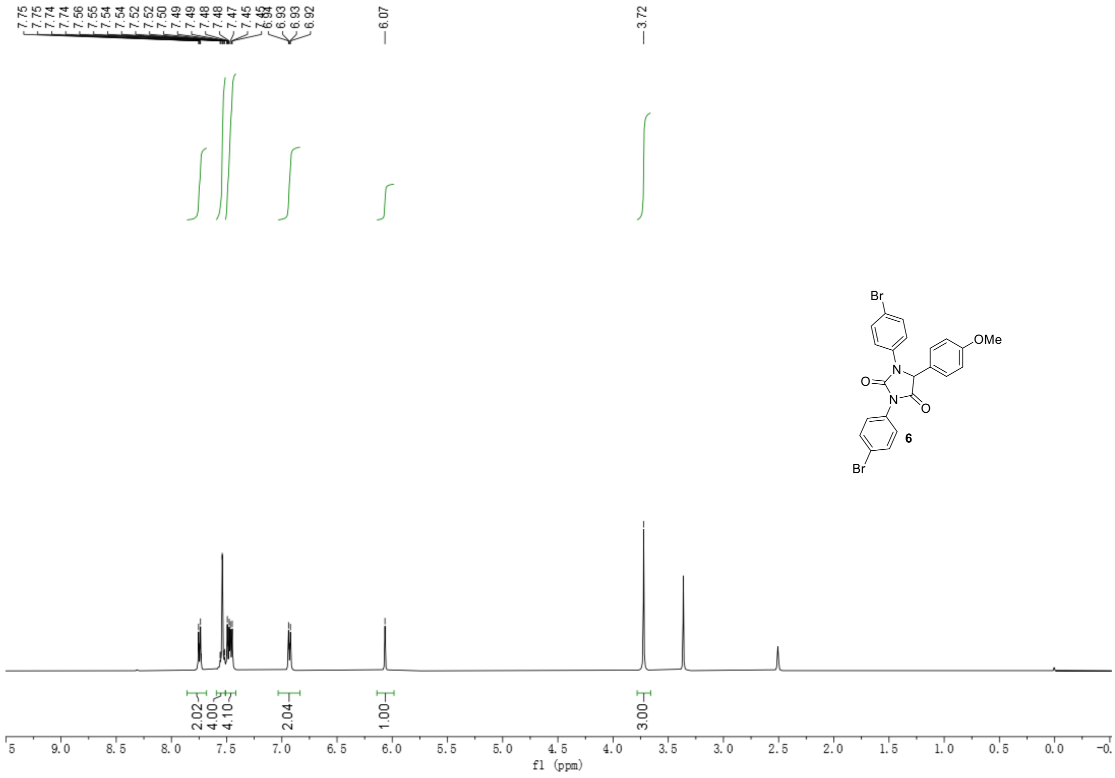

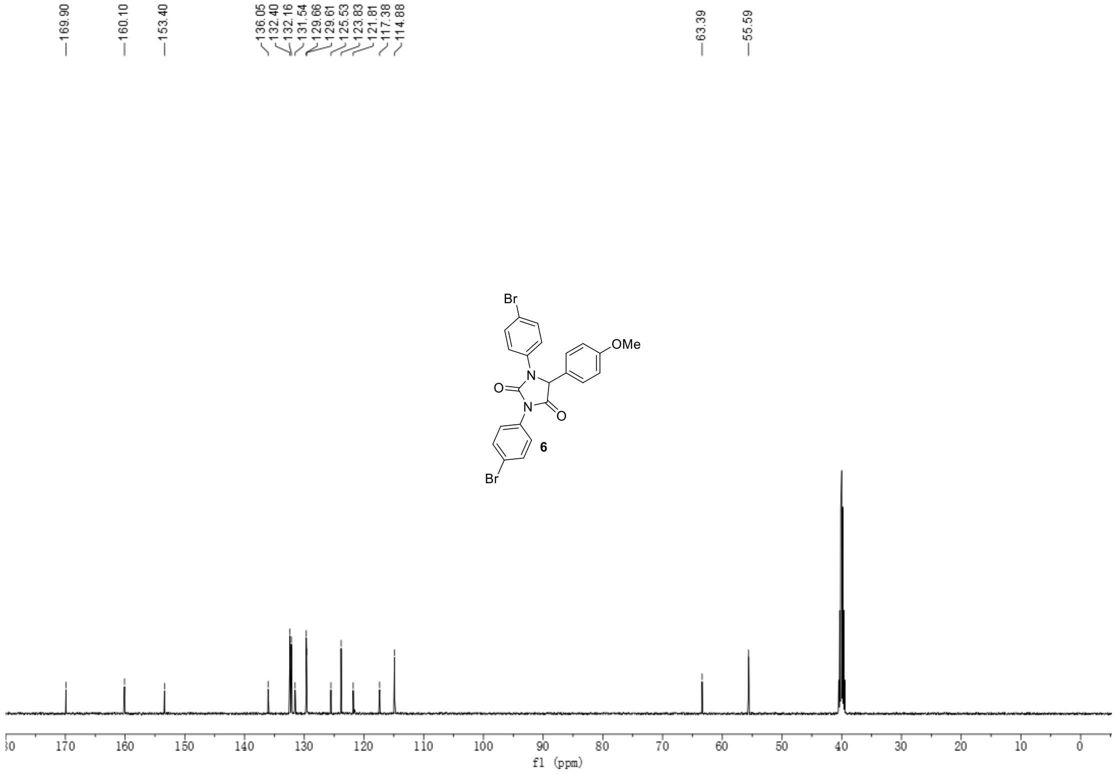

^{1}H NMR (500 MHz, CDCl3), ^{13}C NMR (125 MHz, CDCl3) and ^{19}F NMR (471 MHz, CDCl3) spectra for 7

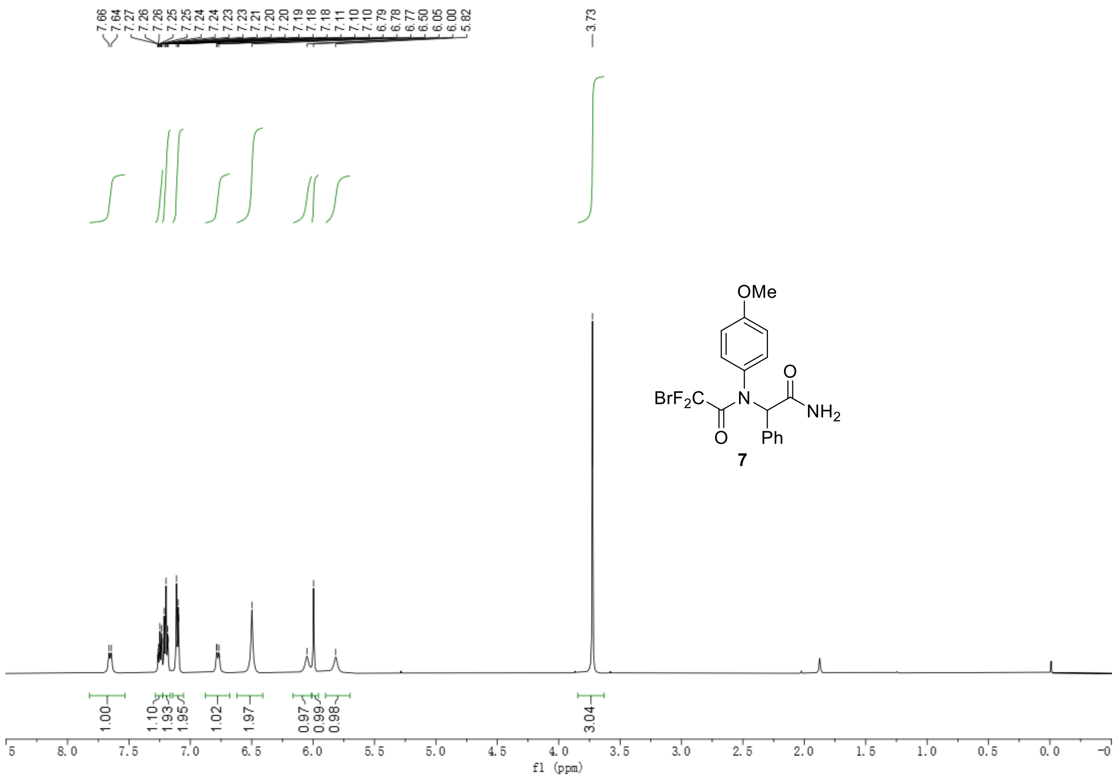

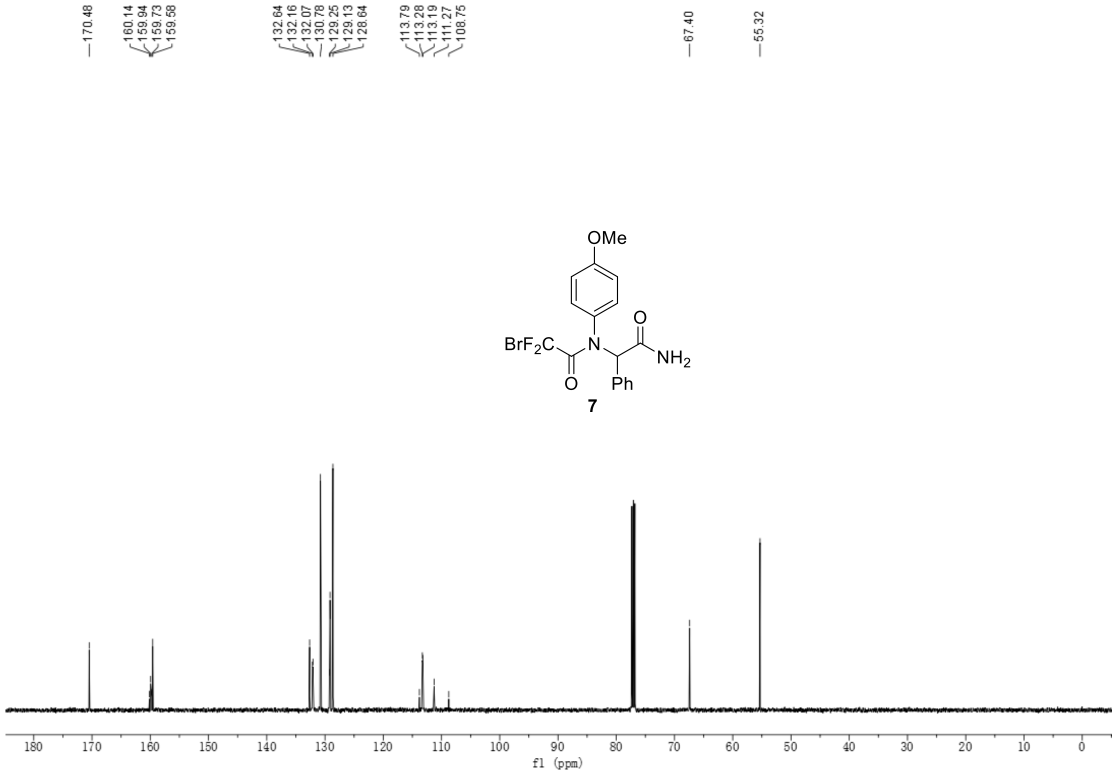

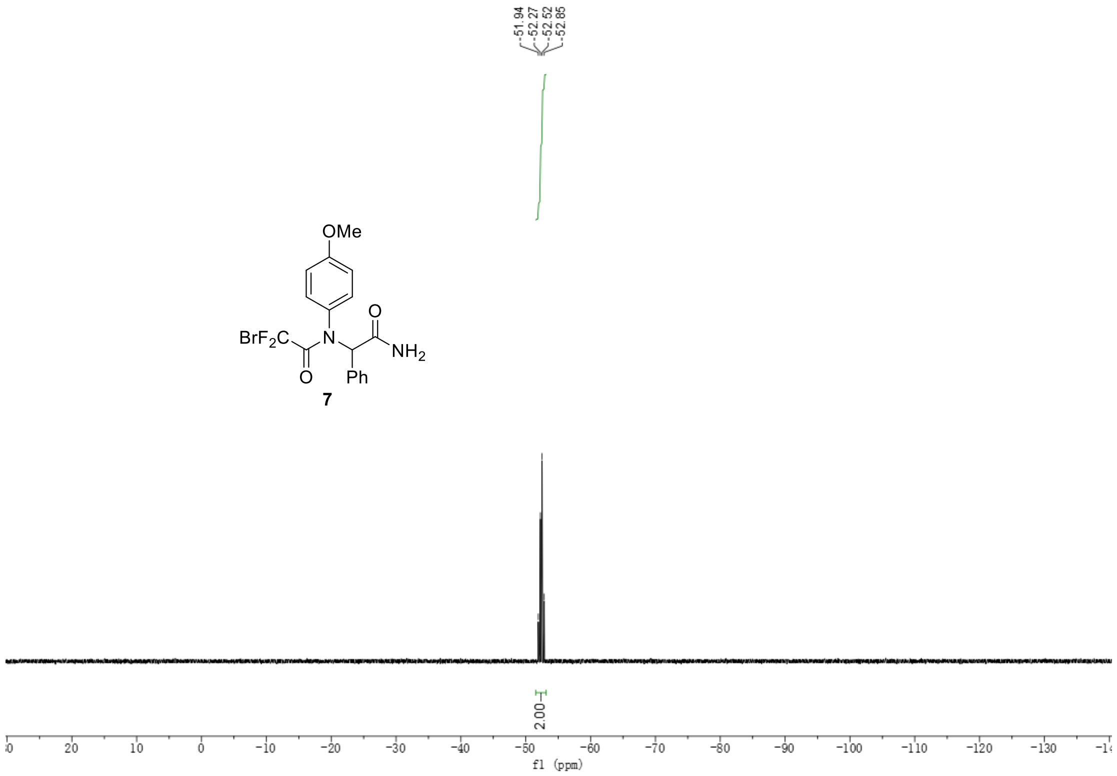

^{1}H NMR (500 MHz, DMSO) and ^{13}C NMR (125 MHz, DMSO) spectra for 9

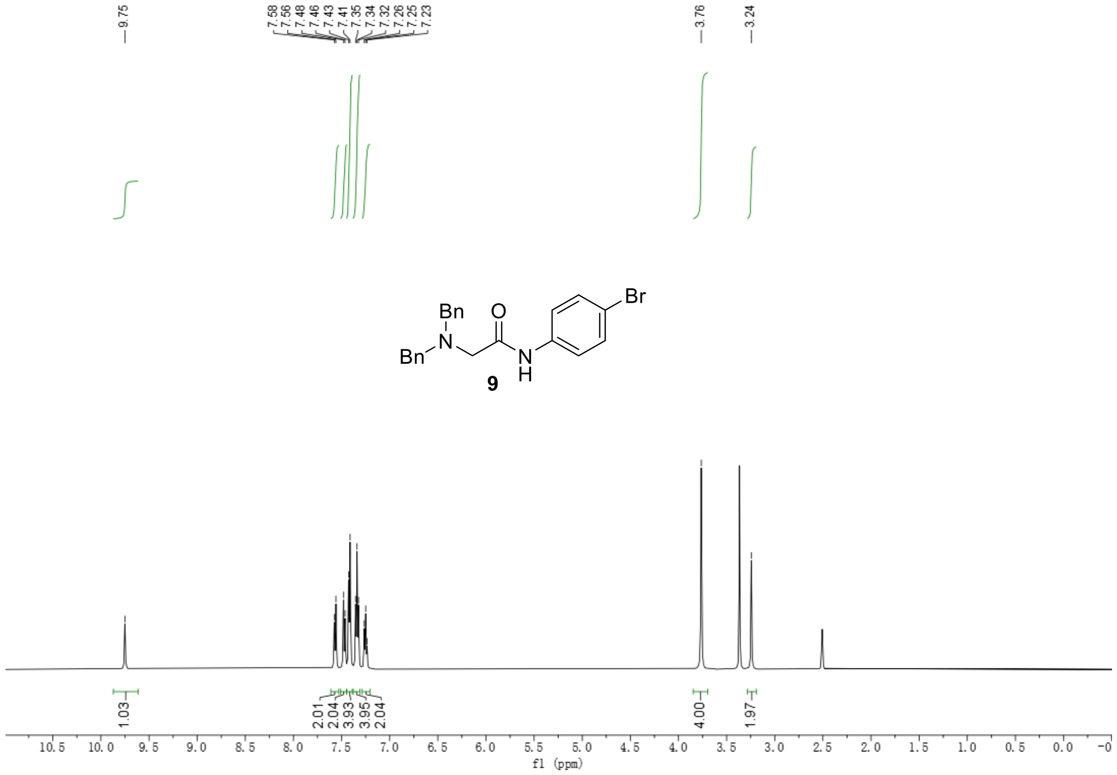

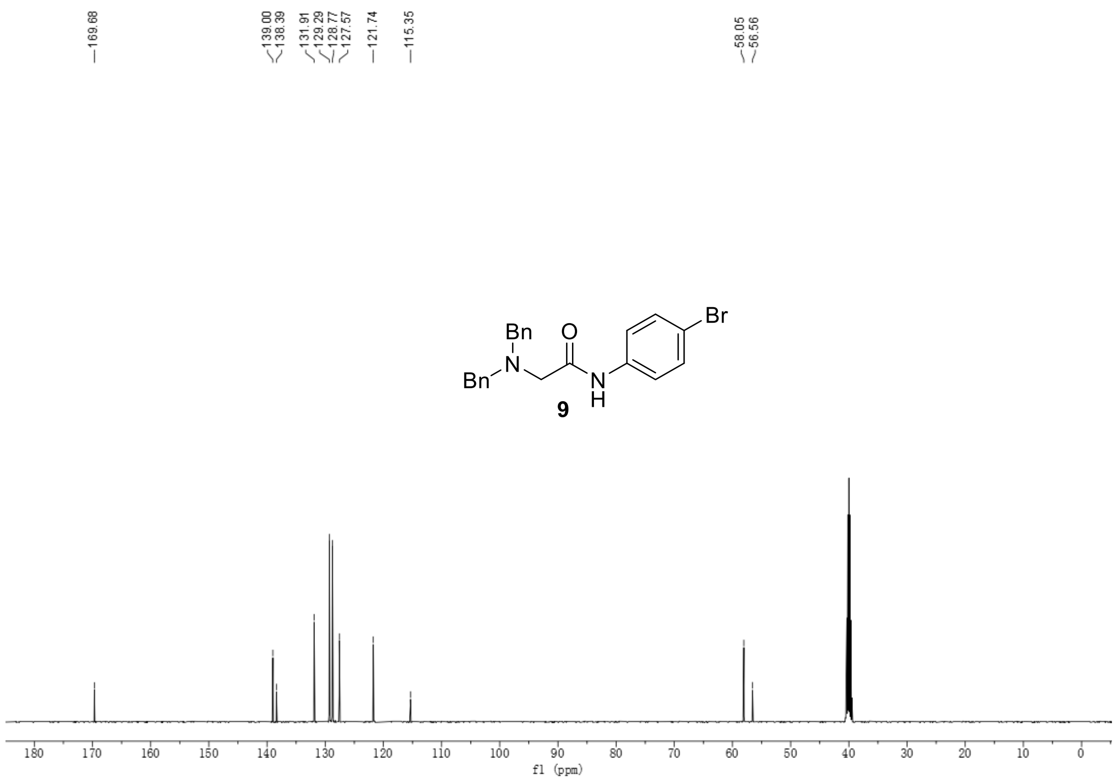

9. Reference

1. Huang, Y., Liu, Z., Liu, W. H. Deaminative addition of alkylpyridinium salt to aldehyde. *Org. Lett.* **2023**, *25*, 4934-4939.

2. Gaussian 16, Revision C.02; M. J. Frisch, G. W. Trucks, H. B. Schlegel, G. E. Scuseria, M. A. Robb, J. R. Cheeseman, G. Scalmani, V. Barone, G. A. Petersson, H. Nakatsuji, X. Li, M. Caricato, A. V. Marenich, J. Bloino, B. G. Janesko, R. Gomperts, B. Mennucci, H. P. Hratchian, J. V. Ortiz, A. F. Izmaylov, J. L. Sonnenberg, D. Williams-Young, F. Ding, F. Lipparini, F. Egidi, J. Goings, B. Peng, A. Petrone, T. Henderson, D. Ranasinghe, V. G. Zakrzewski, J. Gao, N. Rega, G. Zheng, W. Liang, M. Hada, M. Ehara, K. Toyota, R. Fukuda, J. Hasegawa, M. Ishida, T. Nakajima, Y. Honda, O. Kitao, H. Nakai, T. Vreven, K. Throssell, J. A. Montgomery, Jr., J. E. Peralta, F. Ogliaro, M. J. Bearpark, J. J. Heyd, E. N. Brothers, K. N. Kudin, V. N. Staroverov, T. A. Keith, R. Kobayashi, J. Normand, K. Raghavachari, A. P. Rendell, J. C. Burant, S. S. Iyengar, J. Tomasi, M. Cossi, J. M. Millam, M. Klene, C. Adamo, R. Cammi, J. W. Ochterski, R. L. Martin, K. Morokuma, O. Farkas, J. B. Foresman, and D. J. Fox, : Gaussian,Inc., Wallingford CT, 2016.

3. Becke, A. D., Density‐functional thermochemistry. III. The role of exact exchange. *J. Chern. Phys.* **1993**, *98 (7)*, 5648-5652.

4. Lee, C.; Yang, W.; Parr, R. G., Development of the Colle-Salvetti correlation-energy formula into

a functional of the electron density. *Physical Review B* **1988**, *37 (2)*, 785-789.

5. Becke, A. D., Density-functional exchange-energy approximation with correct asymptotic behavior. *Phys Rev A Gen Phys* **1988**, *38 (6)*, 3098-3100.

6. Weigend, F.; Ahlrichs, R., Balanced basis sets of split valence, triple zeta valence and quadruple zeta valence quality for H to Rn: Design and assessment of accuracy. *Phys. Chem. Chem. Phys.* **2005**, *7 (18)*, 3297-305.

7. Fukui, K., The path of chemical reactions-the IRC approach. *Acc. Chem. Res.* **1981**, **14 (12)**, 363-368.

8. Weigend, F., Accurate Coulomb-fitting basis sets for H to Rn. *Phys. Chem. Chem. Phys.* **2006**, *8 (9)*, 1057-65.

9. Marenich, A. V.; Cramer, C. J.; Truhlar, D. G., Universal Solvation Model Based on Solute Electron Density and on a Continuum Model of the Solvent Defined by the Bulk Dielectric Constant and Atomic Surface Tensions. *J. Phys. Chem. B* **2009**, *113 (18)*, 6378-6396.

10. Legault, C. Y., Université de Sherbrooke,´CYLView 2.0. 2020, http://www.cylview.org.

11. Zeng, X., Li, Y., Min, Q.-Q., Xue, X.-S. & Zhang, X. Copper catalyzed difluorocarbene transfer enables modular synthesis. *Nat. Chem.* **2023**, 15, 1064-1073.

12. Tan, T. D., Zhou, F., Quirion, K. P., Wang, Y. Q., Ng, D. Z. W., Luo, X., Chan, E. C. Y., Liu, P., Koh, M. J. Catalytic difluorocarbene insertion enables access to fluorinated oxetane isosteres. *Nat. Chem.* **2025**, 17, 719–726.
